# Supplementary material for: Effect of concussion on salary and employment: a population-based event time study using a quasi-experimental design
Source: BMJ Open. 2020 Oct 21;10(10):e038161. doi: 10.1136/bmjopen-2020-038161 (PMC7580067; doi:10.1136/bmjopen-2020-038161)
Supplement: Supplementary data [file bmjopen-2020-038161supp001.pdf]

SUPPLEMENTAL MATERIALS AND METHODS

Our quasi-experimental, difference-in-differences event time approach compares two groups of individuals from the same cohort, where both groups experience concussions, but at two different time points (t_c,$t_c+\Delta$). For the simple situation where we have three periods (t=0,1,2) and the exposure group (T) experiences their concussion at the start of period 1 ($t_c=1$), and the control group (C) at the start of period 2 ($t_c+\Delta=2$), the effect of concussion on salary (Y) is:

$$\Delta = \left(Y_1^T - Y_1^C\right) - \left(Y_0^T - Y_0^C\right)$$

The effect of concussion on salary in t=1 is estimated by comparing the average difference in salary between exposure and control groups for the post-concussion period t=1 $\left(Y_1^T - Y_1^C\right)$ to the average difference in salary for the pre-concussion, or baseline, interval t=0 $\left(Y_0^T - Y_0^C\right)$. Assuming the exact timing of a concussion is random for small enough sizes of Δ, and under the additional assumption that the exposure group would have had parallel trends in salary as the control group absent suffering concussion at t_c, δ captures the causal effect of concussion among those who suffer concussions – also known as the average effect on the treated (AT). The AT does not capture how concussions would affect a random person. The AT captures how concussions causally affect those who suffer concussions.

For our study, the parallel trends assumption states that exposure and control groups have parallel developments in salary leading up to the exposure group's concussion and the exposure and control groups would have further exhibited parallel salary trajectories if the concussion had not occurred. To test the parallel trends assumption, we estimate a dynamic version of the model specification (shown in supplementary table S1), which explicitly allows us to test whether the parallel trend assumption for our sample is probable.

To validate that the timing of concussion is random with our study period, we present estimates for effect of exposure across different periods between exposure and control incident (Δ). Most recorded concussions outside contact sports and military engagements stem from unforeseen events, such as falls or striking/being struck by an object[25,26], so assuming random timing is likely valid. People who regular engage in activities that result in high risk of multiple concussions may be different than the average concussion patient and would be more likely to end up in the exposure sample than in the control sample, which could induce bias. To avoid such potential bias, we restrict our sample to individuals without prior diagnoses for intracranial injuries ten years prior to exposure.

At t=-1, i.e. one year before the exposure group suffered a concussion, the control groups were slightly smaller than the exposure group, and two control groups (Δ=4 and 5) differed slightly but significantly in terms of average patient age ($p <. 001$; supplementary table S2), male to female ratio ($p <. 001$), and for control group Δ=5, in the frequency of individuals with at least a high school degree ($p < .001$). However, the differences are numerically small. To test that composition differences between exposure and control do not drive our results, we provide separate results for individuals with and without high school degree, for males and females, and for different age groups across all different values of Δ.

Further, our design inherently leads to the possibility of timing issues—our exposure group always suffers their concussion earlier (in terms of calendar time and age) than the control groups do. If the labor market is constantly improving or worsening during the period we consider, this could substantially influence our results. Therefore, we also estimate separate models across exposure incident year and control group. Estimating separate models allow us the added benefit of being able to examine whether the business cycle influences the effect of concussions on salary.

Statistical model

To estimate the impact of concussion on salary, we define the following variables: Exposure or control group *g*, which includes individuals *i*, at times to exposure-groups concussion incident *t*. First, we estimate a standard difference in differences model for each separate control group Δ={1, 2, 3, 4, and 5} using ordinary least squares:

$$Salary_{git} = \beta_0 + \gamma exposure_g + \theta post_t + \delta post \times exposure_{git} + \boldsymbol{X_i \beta} + \sum_{Age=26}^{48+\Delta} I(Age)\eta_{age} + \sum_{year=1999}^{2012} I(year)\,\eta_{year} + \epsilon_{git} \qquad \text{(S1)}$$

where $Salary_{git}$ measures annual salaried income deflated to 2015-level, $exposure_g$ indicates whether the observation belongs to the exposure or control group, $post_t$ captures the period after the exposure group's concussion occurred, and $post_t \times exposure_{git}$ captures the effect concussion, measured as share of year $t \geq 0$ affected by concussion. In this way, someone who suffers a concussion July 1 has $post_t \times exposure_{git} = 0.5$ for $t = 0$ and $post_t \times exposure_{git} = 1$ for $t > 0$. $\boldsymbol{X_i}$ is a set of covariates that includes a high school indicator and a gender dummy, ϵ_{git} is the error-term, and the two last sets of indicator variables $I(Age)$ and $I(Year)$ capture age and incident year levels (control group indexed against incident year). Under the parallel trends assumption, δ then captures the annual effect of concussion on salary. In eq. 1, $exposure_g$ normalizes any pre-exposure differences between the exposure and control group, thereby creating a joint baseline pre-exposure.

We estimate robust individual-level clustered standard errors to account for the possibility that individuals enter the data twice both as control (0) and exposure (1) individuals (g={0,1}), and that they are observed for multiple periods (t={-4,…, Δ-1}). To calculate the relative salary decrease after concussion, we exploit the parallel trends assumption to generate the expected counterfactual salary level, i.e. had the concussion not occurred, and calculate the decline expressed in percentage as: $\%\text{ change} = \delta\ /\ E(\widehat{Salary}_{git}|g = 1, post_t = 1, post_t \times exposure_{git} = 0)$. In this way, we provide both absolute estimates measured in 1K Euro, as well as percentage change.

We expect δ from eq. (1) to likely be negative. Yet, a decrease in annual salary can arrive through two different channels. Concussions may affect salary through either decreasing income among those employed or by reducing the number of individuals who are employed and earning any salary at. To parse out which of the two channels is driving the results, we examine how concussion affects the salary distribution among the exposure group following. Following Chernozhukov et al.[27] we estimate a series of regressions across the whole salary distribution, where, for a finite set of points, we predict how concussion affects the likelihood of having earnings on the left side of each finite point, as follows:

$$\sum_{j=0}^{\max(Salary)} p_j = \beta_{0j} + \delta_j post_t \times exposure_{git} + \theta post_t + \gamma_j exposure_g + \boldsymbol{X_i\beta} + \sum_{Age=26}^{48+\Delta} I(Age)\eta_{age,j} + \sum_{year=1999}^{2012} I(year)\,\eta_{year,j} + \epsilon_{git,j} \quad \text{(S2)}$$

where $p_j = \Pr(Salary_{git} \leq j)$ and j is the interval from 0 to $max(Salary)$. Across the salary distribution, we can now predict the probability of earning less than j for those with and without concussions. From equation 2, we predict $p_j^1 = E(p_j|post_t \times exposure_{git} = 1, exposure_g = 1, t \geq 0)$ and the counterfactual $p_j^0 = E\left(p_j\middle|post_t \times exposure_{git} = 0, exposure_g = 1, t \geq 0\right)$. Plotting p_j^1 and p_j^0 over each value of salary j, and assuming rank stability, gives the cumulative density function of salary for the treated (p_j^1) and the counterfactual observation of the treated had they not suffered concussions (p_j^0). The difference between p_j^1 and p_j^0 is simply δ_j. If the value of δ_j monotonically moves towards zero as j increases until $p_j^1 \approx p_j^0 \approx 1$ it indicates that exit from employment fully drives the effect of concussion on salary. If instead the value of δ_j is constant or increasing across parts of the distribution, it instead indicates that a decrease in salary among those still receiving salary drives at least part of the effect.

Eq. 1 and eq. 2 are based on the parallel trends assumption. The assumption states that exposure and control groups follow parallel salary trajectories until individuals in the exposure group experiences a concussion, and that the parallel trends would have continued had the concussion not occurred. Whereas we cannot verify the counterfactual situation of parallel trends after exposure, we can use a dynamic model to test for systematic differences in salary trends between exposure and control group in the years leading up to the exposure group's concussion event. To do so, we estimate the following dynamic model:

$$Salary_{git} = \beta_0 + \sum_{t\neq-1,t=-4}^{\Delta-1} \delta_t \times I(t_g) \times exposure_g + \sum_{t=-4}^{\Delta-1} I(t_g)\eta_t + \gamma exposure_g + \boldsymbol{X_i\beta} + \sum_{Age=26}^{48+\Delta} I(Age)\eta_{age} + \sum_{year=1999}^{2012} I(year)\,\eta_{year} + \epsilon_{git} \quad \text{(S3)}$$

Where we interact exposure group status (*exposure*$_g$) with indicators $I(t_g)$ capturing time from concussion. If the parallel trends assumption holds, then it must be the case $\{\delta_{-4}, \delta_{-3}, \delta_{-2}\}=0$, whereas the size and sign of $\{\delta_{0}\ldots\delta_{\Delta-1}\}$ captures the dynamic effect of a concussion from the year of incidence and Δ-1 years onward. By estimating the effect of concussion on salary among different years of the study period, we are also able to capture how the impact of concussion on salary evolves year to year after the concussion has occurred. We further estimate eq. 3 for a series of related labor market outcomes (annual total income, annual amount of sickness benefits received, annual probability of being employed), to generate a more thorough understanding on how concussions affect labor market outcomes—i.e., if people experience a decrease in salary due to a concussion, are they then compensated through different types of welfare state services.

25 Faul M, Coronado V. Epidemiology of traumatic brain injury. *Handbook of clinical neurology* **127**:3-13, 2015

26 Taylor CA, Bell JM, Breiding MJ, Xu L. Traumatic Brain Injury–Related Emergency Department Visits, Hospitalizations, and Deaths — United States, 2007 and 2013. *MMWR Surveillance Summaries* **66**:1-16, 2017

27 Chernozhukov V, Fernández-Val I, Melly B. Inference on Counterfactual Distributions. *Econometrica* **81**:2205-2268, 2013
