# Supplementary material for: Effect of concussion on salary and employment: a population-based event time study using a quasi-experimental design
Source: BMJ Open. 2020 Oct 21;10(10):e038161. doi: 10.1136/bmjopen-2020-038161 (PMC7580067; doi:10.1136/bmjopen-2020-038161)
Supplement: Supplementary data [file bmjopen-2020-038161supp002.pdf]

```
********************************************************************
*************************************************
********************************************************************
*************************************************
********************************************************************
*************************************************
*****
***** This study relies on restricted individual level
administrative data obtained
***** from Statistics Denmark.
*****
***** Only Danish research environments are granted authorization to
access data from Statistics Denmark. ***** Foreign
***** researchers can, however, get access to micro data through an
affiliation to a Danish authorized
***** environment.
***** Access is given to anonymized micro data, i.e. data at an
individual personal or corporate level. ***** Access takes
***** place through researcherís own pc over the Internet.
*****
***** See https://www.dst.dk/en/TilSalg/Forskningsservice for detail
*****
***** For the replication of present study, contact the ROCKWOOL
Foundation for access.
***** http://www.rockwoolfonden.dk/en/
*****
********************************************************************
*************************************************
********************************************************************
*************************************************
********************************************************************
*************************************************



********************************************************************
*************************************************
********************************************************************
*************************************************
********************************************************************
*************************************************
*****
***** This is the master data do-file for the full package of do-
files that generates the results for
***** Fallesen and Campos (2020). Execute files in the order they
are listed. For each file, specify a ***** home registry in place of
[home]. Further, generate subfolders [home]/data, [home]/tables, and
***** [home]/highdef to capture auxiliary data sets, tables, and
figures.
*****
********************************************************************
*************************************************
********************************************************************
*************************************************
```

```
********************************************************************
*************************************************

***Install user-written reghdfe command for faster computation of
regressions
ssc install reghdfe

***Generate concussion samples and merges on covariates and outcome
do 01generate_sample.do

***Generates auxilary data sets the includes social benefit
recipiency indicator and levels of benefits received
do 02generate_benefits.do

***Generates auxilary data sets the includes social benefit
recipiency indicator and levels of benefits received
do 02generate_benefits.do

***Generate results
do 03generate_figures_and_results

exit, clear
```

```
clear all

********************************************************************
***********
**
**                This program builds data for Fallesen & Campos
(2020)
**                study of concussion's impact on productivity
measured
**                through annual salary
**
********************************************************************
***********


**Global for path to registry data
global dorg "E:/data/rawdata/706630"

*Global for processed data
global data "[home]/data"

/*globals for price index to calculate income at 2015-level across
years*/
/*Price index obtained from www.dst.dk/en/statistik/emner/priser-og-
forbrug/forbrugeriser/nettoprisindeks */

{
global price1980 = .358
global price1981 = .398
global price1982 = .439
global price1983 = .466
global price1984 = .494
global price1985 = .517
global price1986 = .521
global price1987 = .537
global price1988 = .564
global price1989 = .594
global price1990 = .612
global price1991 = .628
global price1992 = .642
global price1993 = .651
global price1994 = .662
global price1995 = .674
global price1996 = .688
global price1997 = .703
global price1998 = .713
global price1999 = .728
global price2000 = .751
global price2001 = .769
global price2002 = .788
global price2003 = .806
global price2004 = .817
global price2005 = .833
global price2006 = .850
```

```
global price2007 = .867
global price2008 = .899
global price2009 = .917
global price2010 = .936
global price2011 = .960
global price2012 = .978
global price2013 = .986
global price2014 = .994
global price2015 =1.00
global price2016 =1.005
global price2017 =1.017
}


/*Locate concussions and other TBIs from the Danish National Patient
Registry */
/**/
forvalue t = 1977/2017{
        if `t' < 1994 use $dorg/lpr_diag`t'.dta /// **uses ICD-8
codes until Dec. 31, 1993
                if substr(c_diag,1,2)=="85"
        if `t' > 1993 use $dorg/lpr_diag`t'.dta /// **uses ICD-10
codes from Jan. 1, 1994
                if substr(c_diag,1,4)=="DS06"
        **recovers encrypted social security number and admittance
date
        merge m:m recnum using $dorg/lpr_adm`t', keepus(pnr d_ind*)
keep(3)
        drop _merge recnum


        **Keeps diagnosis, diagnosis type, and individual id (pnr)
        keep pnr c_diag  c_diagtype pnr d_ind

        **generate year variable
        gen year = year(d_ind)

        **geenrate share of year with concussion
         gen time_from_incident = 1-((d_ind-mdy(1,1,year(d_ind)))/
365)
        drop d_ind

        *save as one dataset
        if `t' >  1977 append using $data/concussion.dta
        if `t' == 2017 sort pnr year
        save $data/concussion.dta, replace


}
*/
/********************************************
Sets up datasets for treatment group (x = 0)
and the control groups who suffer concussion
1, 2, 3, 4, 5 years later (x = 1 2 3 4 5).
```

```
Treatment group are not allowed to have suffered
any type of TBI the last 10 years before concussion
, control group are not allowed to have suffered any TBI
concussion the last 10 + x years.

*****************************************/
/**/
forvalue x = 0/5{
        use $data/concussion.dta, clear
        sort pnr year

        **generate measure of length between registered TBIs
        by pnr: gen help = year-year[_n-1]

        drop if help < 10+`x'

        **Keep only concussion, and only when it was primary
diagnosis
        keep if c_diagtype== "A" & ///    Primary daignosis
                (c_diag == "85099" | /// ICD-8 code for concussion
                substr(c_diag,1,5) == "DS060") // ICD-10 code for
concussion

        *generate treatment and control datasets
        save $data/concussion_`x'.dta, replace

}



*/
/***************************************************************
Generate datasets for analysis. First incident year is allowed
to be 1992, because it is the first year where we have full record
for the five year plus control group (1977+10+5 = 1992).

We generate seperate datasets for each incident year for treatment
group and different control groups.

***************************************************************/

forvalue time = 1/5{            //for different control groups

        local post_period = 5    // local for number of years
observed post
                                                          //
concussion for treatment group

        local endtime = 2017-`post_period'        /*last year where
we allow for treatment event
                                                          to
occur, in order to have long enough control
                                                          period.
```

```
Defined by latest year available data*/

        forvalue count = 1992(1)`endtime'{
                local t = `count'                       // for
ease of coding
                local n = `t'-4                         // first
pre-treatment event period
                local c = `t'+`post_period'     //last post-event
period
                local w = `t'+`time'            //time of
concussion for control

                use pnr alder using $dorg/bef`t'         ///
Bring in all 30-49 yr olds
                        if inrange(alder`t',20,59), clear // from
the population register

                **year variable
                gen year = `t'

                **limit sample to those who suffer a concussion in
`t'
                merge 1:1 pnr year using $data/
concussion_0.dta, ///
                        keep(3) nogen

                forvalue x=`n'/`c'{     //add longitudinal data
                        merge 1:1 pnr using $dorg/bef`x', ///
                                keep(1 3) keepus(efalle alder
koen)   //add information on spouse,

                                        //age, and gender
                        rename _merge merge`x' //indicator for
whether in DK that year

                        **Add salary information and ses
information
                        if `x' < 2017{
                                merge 1:m pnr using $dorg/
ind`x', ///      m:1 to account for duplicates
                                        nogen keep(1 3)
keepus(erhvervsindk_13 pre_socio personindk dispon_13
aekvivadisp_13)  // in data on non-important variables

                                bysort _all: keep if _n ==1
                                        //drop perfect duplicates
                                **Align variable names and account
for inflation

                                rename erhvervsindk_13 loenmv
                                rename pre_socio pre_socio`x'

                                foreach kk in personindk dispon_13
aekvivadisp_13 loenmv{
```

```
                                                  rename `kk'
`kk'`x'
                                  }

                                  foreach kk in personindk dispon_13
aekvivadisp_13 loenmv{
                                                  replace `kk'`x' =
        `kk'`x'/${price`x'}
                                  }

                                  **Bring in educational information
                                  merge 1:1 pnr using $dorg/
uddany`x', ///
                                  nogen keep(1 3) keepus(hffsp)
                          }
                          if `x' == 2017{
                                  merge 1:m pnr using $dorg/
ind`x', ///      m:1 to account for duplicates
                                          nogen keep(1 3)
keepus(erhvervsindk_13 pre_socio personindk)      // in data on
non-important variables

                                  bysort _all: keep if _n ==1
                                          //drop perfect duplicates
                                  **Align variable names and account
for inflation

                                  rename erhvervsindk_13 loenmv
                                  rename pre_socio pre_socio`x'

                                  foreach kk in personindk loenmv{
                                                  rename `kk'
`kk'`x'
                                  }

                                  foreach kk in personindk loenmv{

                                                  replace `kk'`x' =
        `kk'`x'/${price`x'}
                                  }

                                  **Bring in educational information
                                  merge 1:1 pnr using $dorg/
udda`x', ///
                                          nogen keep(1 3)
keepus(hfaudd)
                                  rename hfaudd hfaudd`x'
                          }
                  }

                  **Reshape data to panel structure
                  if `count'>= 2012 reshape long efalle alder koen
loenmv pre_socio personindk dispon_13 aekvivadisp_13 hffsp merge
hfaudd , i(pnr) j(t)
```

```
                 if `count' < 2012 reshape long efalle alder koen
loenmv pre_socio personindk dispon_13 aekvivadisp_13 hffsp merge  ,
i(pnr) j(t)

                 gen count = t-year       //variable for time to
concussion
                 gen treatment =1 //treatment group indicator

                 save $data/sample_temp.dta, replace //temporary
dataset

                 /
*********************************************************
                 Now build control sample for time `time' and year
`count'

*********************************************************/



                 use pnr alder using $dorg/bef`t'          ///
Bring in all 30-49 yr olds
                          if inrange(alder`t',20,59), clear // from
the population register

                 **year variable
                 gen year = `w'   //time for concussion for control
group `time'

                 **limit sample to those who suffer a concussion in
`t'
                 merge 1:1 pnr year using $data/
concussion_`time'.dta, ///
                          keep(3) nogen

                          forvalue x=`n'/`c'{      //add
longitudinal data
                                   merge 1:1 pnr using $dorg/
bef`x', ///
                                            keep(1 3) keepus(efalle
alder koen)      //add information on spouse,

                                                     //age, and gender
                                   rename _merge merge`x' //indicator
for whether in DK that year
                                   if `x' < 2017{
                                            **Add salary and SES
information
                                            merge 1:m pnr using
$dorg/ind`x', ///        1:m to account for duplicates
                                                     nogen keep(1 3)
keepus(erhvervsindk_13 pre_socio personindk dispon_13
aekvivadisp_13) // in data on non-important variables
```

```
                                        bysort _all: keep if _n
==1                                             //drop perfect
duplicates

                                        **Align variable names
and account for inflation

                                        rename erhvervsindk_13
loenmv
                                        rename pre_socio
pre_socio`x'

                                        foreach kk in personindk
dispon_13 aekvivadisp_13 loenmv{
                                                rename `kk'
`kk'`x'
                                        }

                                        foreach kk in personindk
dispon_13 aekvivadisp_13 loenmv{
                                                replace `kk'`x' =
        `kk'`x'/${price`x'}
                                        }

                                        **Bring in educational
information
                                        merge 1:1 pnr using
$dorg/uddany`x', ///
                                        nogen keep(1 3)
keepus(hffsp)
                                }
                        if `x' == 2017{
                                merge 1:m pnr using $dorg/
ind`x', ///      m:1 to account for duplicates
                                        nogen keep(1 3)
keepus(erhvervsindk_13 pre_socio personindk)      // in data on
non-important variables

                                bysort _all: keep if _n ==1
                                        //drop perfect duplicates
                                **Align variable names and account
for inflation

                                rename erhvervsindk_13 loenmv
                                rename pre_socio pre_socio`x'

                                foreach kk in personindk loenmv{
                                                rename `kk'
`kk'`x'
                                }

                                foreach kk in personindk loenmv{

                                                replace `kk'`x' =
```

```
        `kk'`x'/${price`x'}
                                }

                                **Bring in educational information
                                merge 1:1 pnr using $dorg/
udda`x', ///
                                        nogen keep(1 3)
keepus(hfaudd)
                                rename hfaudd hfaudd`x'
                        }
                }

                **Reshape data to panel structure
                if `count'>= 2012 reshape long efalle alder koen
loenmv pre_socio personindk dispon_13 aekvivadisp_13 hffsp merge
hfaudd , i(pnr) j(t)
                if `count' < 2012 reshape long efalle alder koen
loenmv pre_socio personindk dispon_13 aekvivadisp_13 hffsp merge  ,
i(pnr) j(t)
                gen count = t-`t'                //variable for
time to concussion for treatment
                gen control`time' =1     //control indicator
                save $data/control_temp, replace

                **Build sample with treatment and control `time'
for year `count'
                use $data/sample_temp
                append using $data/control_temp

                **fixes control and treatment indicators
                replace control`time' = 0 if control`time'==.
                replace treatment = 0 if treatment==.

                **Picks up changes to education variable
                if `count' >=2012{
                        tostring hfaudd, replace
                        rename hfaudd start
                        merge m:1 start using "\
\srvfsenas1\data\Formater\SAS formater i Danmarks
Statistik\STATA_datasaet\Disced\c_udd_niveau_l1l2_k.dta" , nogen
keep(1 3)
                        destring UDD, replace force
                        **Replace all with high scho0l degree or
higher in HFAUDD to have HFFSP = 40000001
                        replace hffsp = 40000001 if t == 2017 &
inrange(UDD,30,80)
                        replace hffsp = 0 if t == 2017 & !
inrange(UDD,30,80)
                        drop UDD start
                }

                sort pnr t
```

```
                save $data/sample_control_`count'_`time'.dta,
replace

        }
}


forvalue time = 1/5{
        forvalue count =2003/2012{
                if `time' ==1 & `count' ==2003 use $data/
sample_control_`count'_`time'.dta, clear
                else append using $data/
sample_control_`count'_`time'.dta

                if `time' ==5 & `count' ==2012 bysort pnr: keep if
_n ==1
                if `time' ==5 & `count' ==2012  count
        }
}
```

```
clear

********************************************************************
************
********************************************************************
************
**
**               Calculate share of year on public benefits and size
of benefit
**               payments for Fallesen and Campos (2020)
**
**
********************************************************************
************
********************************************************************
************
/*globals for price index to calculate income at 2015-level across
years*/
/*Price index obtained from www.dst.dk/en/statistik/emner/priser-og-
forbrug/forbrugeriser/nettoprisindeks */

global price1980 = .358
global price1981 = .398
global price1982 = .439
global price1983 = .466
global price1984 = .494
global price1985 = .517
global price1986 = .521
global price1987 = .537
global price1988 = .564
global price1989 = .594
global price1990 = .612
global price1991 = .628
global price1992 = .642
global price1993 = .651
global price1994 = .662
global price1995 = .674
global price1996 = .688
global price1997 = .703
global price1998 = .713
global price1999 = .728
global price2000 = .751
global price2001 = .769
global price2002 = .788
global price2003 = .806
global price2004 = .817
global price2005 = .833
global price2006 = .850
global price2007 = .867
global price2008 = .899
global price2009 = .917
global price2010 = .936
global price2011 = .960
global price2012 = .978
```

```
global price2013 = .986
global price2014 = .994
global price2015 =1.00
global price2016 =1.005
global price2017 =1.017


**Global for path to registry data
global dorg "e:/data/rawdata/706630"
*Global for processed data
global data "E:/data/workdata/706630/pf/FallesenCampos/data"

forvalue t=1996/2017{

        ** Read in data on social benefits recipiency share of
weeks
        ** from the DREAM database
        use $dorg/dream`t'
        gen share =0
        forvalue y = 1/52{
                        if `y' < 10 replace share = share+1 if
y_0`y' !=.
                        if `y' >  9 replace share = share+1 if
y_`y' !=.
                        if `y' < 10 drop y_0`y'
                        if `y' >  9 drop y_`y'

        }
        **Generate annual measure of share of year receiving social
benefits
        replace share = share/52
        keep pnr share
        gen t = `t'
        if `t' > 1996 append using $data/temp.dta
        save $data/temp.dta, replace

}


forvalue t=1998/2017{

        **Read in information on size of different types of social
benefits

        if `t' < 2002{
                use pnr syg_barsel_13 konthj arblhum pre_socio
using $dorg/ind`t'.dta, clear
                replace syg_barsel_13 = syg_barsel_13/${price`t'}
                replace konthj  = konthj /${price`t'}
                replace arblhum = arblhum/${price`t'}
                gen kont_dag = konthj+arblhum
                drop konthj arblhum
        }
        if `t' >= 2002 & `t' < 2013{
```

```
                use pnr syg_barsel_13 adagpagn konthj arblhum
pre_socio using $dorg/ind`t'.dta, clear
                replace syg_barsel_13 = syg_barsel_13/${price`t'}
                replace adagpagn = adagpagn/${price`t'}
                replace konthj  = konthj /${price`t'}
                replace arblhum = arblhum/${price`t'}
                gen kont_dag = konthj+arblhum
                drop konthj arblhum
        }
        if `t' >= 2013{
                use pnr syg_barsel_13 adagpagn dagpenge_kontant_13
pre_socio using $dorg/ind`t'.dta, clear
                replace syg_barsel_13 = syg_barsel_13/${price`t'}
                replace adagpagn = adagpagn/${price`t'}
                replace dagpenge_kontant_13  =
dagpenge_kontant_13 /${price`t'}
                gen kont_dag = dagpenge_kontant_13-syg_barsel_13
                drop dagpenge_kontant_13
        }
        gen t = `t'
        compress
        bysort pnr: keep if _n ==1
        if `t' > 1998 append using $data/temp2.dta
        if `t' == 2017{
                sort pnr t
        }
        save $data/temp2.dta, replace
}
```

```
clear all

*******************************************************************
***********
**
**              This program geenrates figerus and results for
Fallesen & Campos (2020)
**              study of concussion's impact on productivity
measured through
**              annual salary
**
*******************************************************************
***********

**Global for path to registry data
global dorg "E:/data/rawdata/706630"

*Global for processed data
global data "[home]/data"

*Global on figures
global highdef "[home]\highdef"



forvalue time = 1/5{             //for different control groups

        local post_period = 5    // local for number of years
observed post
                                                          //
concussion for exposure group

        local endtime = 2017-`post_period'        /*last year where
we allow for exposure event
                                                          to
occur, in order to have long enough control
                                                          period.
Defined by latest year available data*/

        **Matrixes to capture estimates
        matrix results   = J(15,6,.)      // For salary estimates
        matrix results_p = J(15,6,.)      // For Pr(salary=0)
estimates
        matrix t         = J(15,6,.)      // For time indicators

        forvalue count = 2003(1)`endtime'{
                use $data/sample_control_`count'_`time', clear

                gen female = koen==2

                qui{
                        gen edu =0
                        replace edu = 1 if inrange(hffsp,
20000000,39000000) | ///
```

```
(hffsp >40000000 & hffsp!=.)
                 }

                 **exclude individuals in years where they do not
appear in data,
                 **due to either death or migration, as well as
periods from when
                 **the control group sufer their concussion
                 drop if merge ==1 | count > `time'-1

                 **generate concussion variable
                 gen treat = inrange(count,0,`time'-1) & treatment
==1
                 replace treat = time_from_incident if count ==0 &
treatment ==1

                 **Generate pre-concussion income difference
for
                 **use in calculating marginal effects
                 sum loenmv if count <0 & treatment ==0
                 local control =r(mean)
                 sum loenmv if count <0 & treatment ==1
                 local treat =r(mean)
                 sum loenmv if count>=0 & treatment ==0
                 local control_post =r(mean)
                 gen post = count >=0

                 **estimate DiD model on salary
                 reghdfe loenmv treat, abs(alder female post
treatment edu year) cl(pnr)
                 matrix b =e(b)   //regression coefficient
                 matrix V = e(V)  // standard error^2
                 local n = `count'-2002   //time

                 matrix results[`n',1]    =       b[1,1]
                 matrix results[`n',2]    =       V[1,1]^.5
                 matrix results[`n',3]    =       b[1,1]/
(`control_post'-(`control'-`treat'))
                 matrix results[`n',4]    =       `n'


        }
        svmat results

        rename results1 est
        rename results2 se
        rename results3 marg
        rename results4 time

        replace time = time+(`time'-3)*.1 //jitter estimates for
graph
```

```
          keep est* se* marg* time
          keep if est !=.

          replace est = est/7446    //estimate measured as 1000 Euro
          replace se = se/7446      //S.E. measured as 1000 Euro

          gen upper = est+se*1.96 // Upper CI
          gen lower = est-se*1.96 // Lower CI


          gen control = `time'              //indicate control group

          if `time' >1 append using $data/results.dta
          save $data/results.dta, replace
}

use $data/results.dta, clear

replace time = 2002+time


*reads in unemployment statistcis obtained from statistikbanken.dk/
en/

gen unemp = 5.8 if time ==2003
replace unemp = 5.8 if time ==2004
replace unemp = 5.1 if time ==2005
replace unemp = 3.9 if time ==2006
replace unemp = 2.7 if time ==2007
replace unemp = 1.9 if time ==2008
replace unemp = 3.6 if time ==2009
replace unemp = 4.2 if time ==2010
replace unemp = 4.0 if time ==2011
replace unemp = 4.5 if time ==2012

scatter est time if control ==1, mcolor(navy) yaxis(1) ysc(range(-4
3) axis(1)) ylab(-4(1)3)  || ///
scatter est time if control ==2, mcolor(blue) || ///
scatter est time if control ==3, mcolor(midblue) || ///
scatter est time if control ==4, mcolor(gray) || ///
scatter est time if control ==5, mcolor(ltblue) || ///
rspike upper lower time if control ==1, lcolor(navy) || ///
rspike upper lower time if control ==2, lcolor(blue) || ///
rspike upper lower time if control ==3, lcolor(midblue) || ///
rspike upper lower time if control ==4, lcolor(gray) || ///
rspike upper lower time if control ==5, lcolor(ltblue) || ///
line unemp time , lcolor(black) yaxis(2) ysc(range(0 6) axis(2))
ylab(0(1)6, axis(2))  ///
xsc(range(2002.5 2012.5)) xlab(2003(2)2012) ///
yline(0) ysize(10) xsize(12) graphr(c(white)) ///
xti("Year of concussion for exposure group") scale(.95) ///
legend(label(1 " Control," "{&Delta}=1 yr") ///
label(2 " Control," "{&Delta}=2 yr") ///
label(3 " Control," "{&Delta}=3 yr") ///
```

```
label(4 " Control," "{&Delta}=4 yr") ///
label(5 " Control," "{&Delta}=5 yr") ///
label(6 "% Unemp." "of LF") ///
c(1) order(1 2 3 4 5 6) pos(3) size(small) ///
c(1) symx(4) region(lc(white))) ///
yti("Effect on Salary (in EUR 1K)", height(7) axis(1)) ///
yti("Percent of Full Time Uemployed among LF", height(7) axis(2))


graph export $highdef/marg_est.png, replace width(3900)


forvalue control_time=1/5{
        local end = 2012 // last incident year in data
        if `control_time' ==1    eststo clear

        **build dataset for joint estimate across years
        forvalue count=2003/`end'{
                if `count'==2003{
                        use $data/
sample_control_`count'_`control_time'.dta, clear
                        gen time = `count' //incident year
indicator
                }
                else append using $data/
sample_control_`count'_`control_time'.dta
                replace time = `count' if time ==.

                **exclude individuals in years where they do not
appear in data,
                **due to either death or migration, as well as
periods from when
                **the control group sufer their concussion
                drop if merge ==1 | count > `control_time'-1
        }

        gen female = koen==2

        //build ident, so we can multivariate cluster for
individuals
        //who occur both as control and exposure during the period
(id)

        bysort pnr time: gen helpx = _n ==1
        gen id= sum(helpx)
        drop helpx

        **Generate educational groups
        qui{
                gen edu =0
                replace edu = 1 if inrange(hffsp,20000000,39000000)
| ///
                                                            (hffsp
>40000000 & hffsp!=.)
```

```
	}

	**Calculate number of observations for exposure and control
	count if count==0 & treatment ==1
	local Ntreated = r(N)
	count if count==0 & treatment ==0
	local Ncontrol = r(N)

	**generate concussion variable
	gen treat = inrange(count,0,`control_time'-1) & treatment 
==1
	replace treat = time_from_incident if count ==0 & treatment 
==1

	**Generate pre-concussion income difference	for
	**use in calculating marginal effects
	sum loenmv if count <0 & treatment ==0
	local control =r(mean)
	sum loenmv if count <0 & treatment ==1
	local treat =r(mean)
	sum loenmv if count>=0 & treatment ==0
	local control_post =r(mean)

	forvalue t=-4/4{
		local n = `t'*-1
		if `t' < -1 gen T_`n' = treatment ==1 & count ==`t'
		if `t' > -1 gen T`t' = treatment ==1 & count ==`t'

	}

	**estimate DiD model on salary
	reghdfe loenmv T*, abs(alder female count time treatment 
edu) cl(pnr  id)
	eststo est1_`control_time'
	if `control_time'==1 matrix results = J(5,5,.) // matrix to 
capture results
	if `control_time'==1 matrix results_p = J(5,5,.) // matrix 
to capture results
	if `control_time'==1 matrix results_pre = J(5,5,.) // 
matrix to capture results

	matrix b = e(b)
	matrix V = e(V)
	local n = `control_time'

	matrix results[`n',1]	= b[1,1] / 7466  // capture beta 
results as 1K Euro
	matrix results[`n',2]	= (V[1,1]^.5)/7466	// 
capture standard error as 1K Euro
	matrix results[`n',3]	=	b[1,1]/(`control_post'-
(`control'-`treat'))
	matrix results[`n',4]	=	`n'

	gen no_lon = loenmv<1 //dummy for no salary
```

```
	**estimate DiD LP-model for pre-trends
	xi: reghdfe loenmv T*, abs(alder female count time 
treatment edu) cl(pnr  id), if count <0
	eststo est3_`control_time'
	matrix b =e(b)   //regression coefficient
	matrix V = e(V)  // standard error^2

	matrix results_pre[`n',1] =	b[1,1]
	matrix results_pre[`n',2] =	V[1,1]^.5
	matrix results_pre[`n',3] =	b[1,1]/(`control_post'-
(`control'-`treat'))
	matrix results_pre[`n',4] =	`n'

}

esttab est1_* using [home]/tables/dynamic1.rtf, ///
	replace se(1) b(1) compress nogap star(+ .1 * .05 ** .01 
*** .001) ///
	keep(T*)

esttab est2_* using [home]/tables/dynamic2.rtf, ///
	replace se(3) b(3) compress nogap star(+ .1 * .05 ** .01 
*** .001) ///
	keep(T*)

esttab est3_* using [home]/tables/pre_trends.rtf, ///
	replace se(3) b(3) compress nogap star(+ .1 * .05 ** .01 
*** .001) ///
	keep(T*)

forvalue control_time=1/5{
	local end = 2012 // last incident year in data
	if `control_time' ==1	eststo clear
	qui{
		**build dataset for joint estimate across years
		forvalue count=2003/`end'{
			if `count'==2003{
				use $data/
sample_control_`count'_`control_time'.dta, clear
				gen time = `count' //incident year 
indicator
			}
			else append using $data/
sample_control_`count'_`control_time'.dta
			replace time = `count' if time ==.

			**exclude individuals in years where they 
do not appear in data,
			**due to either death or migration, as 
well as periods from when
			**the control group sufer their concussion
```

```
        drop if merge ==1 | count >
`control_time'-1
                }

                gen female = koen==2

                        **Generate educational groups
                gen edu =0
                replace edu = 1 if inrange(hffsp,20000000,39000000)
| ///

(hffsp >40000000 & hffsp!=.)


                //build ident, so we can multivariate cluster for
individuals
                //who occur both as control and exposure during the
period (id)

                bysort pnr time: gen helpx = _n ==1
                gen id= sum(helpx)
                drop helpx

                **Calculate number of observations for exposure and
control
                count if count==0 & treatment ==1
                local Ntreated = r(N)
                count if count==0 & treatment ==0
                local Ncontrol = r(N)

                **generate concussion variable
                gen treat = inrange(count,0,`control_time'-1) &
treatment ==1
                replace treat = time_from_incident if count ==0 &
treatment ==1

                **Generate pre-concussion income difference
for
                **use in calculating marginal effects
                sum loenmv if count <0 & treatment ==0
                local control =r(mean)
                sum loenmv if count <0 & treatment ==1
                local treat =r(mean)
                sum loenmv if count>=0 & treatment ==0
                local control_post =r(mean)

                forvalue t=-4/4{
                        local n = `t'*-1
                        if `t' < -1 gen T_`n' = treatment ==1 &
count ==`t'
                        if `t' > -1 gen T`t' = treatment ==1 &
count ==`t'

                }
```

```
                gen post = count > -1

                **estimate DiD model on salary
                reghdfe loenmv treat, abs(alder female post time
treatment edu) cl(pnr id)
                eststo est1_`control_time'
                if `control_time'==1 matrix results = J(5,5,.) //
matrix to capture results
                if `control_time'==1 matrix results_p = J(5,5,.) //
matrix to capture results


                matrix b = e(b)
                matrix V = e(V)
                local n = `control_time'

                matrix results[`n',1]    = b[1,1] / 7466  //
capture beta results as 1K Euro
                matrix results[`n',2]    = (V[1,1]^.5)/
7466    // capture standard error as 1K Euro
                matrix results[`n',3]    =       b[1,1]/
(`control_post'-(`control'-`treat'))
                matrix results[`n',4]    =       `n'


        }
        *examining balance of samples
        di in ye `control_time'
        bysort treatment: sum female alder edu if count ==0

}



svmat results

gen upper = results1+results2*1.96
gen lower = results1-results2*1.96

scatter results1 results4 if results4 ==1, mcolor(navy) || ///
scatter results1 results4 if results4 ==2, mcolor(navy) || ///
scatter results1 results4 if results4 ==3, mcolor(navy) || ///
scatter results1 results4 if results4 ==4, mcolor(navy) || ///
scatter results1 results4 if results4 ==5, mcolor(navy) || ///
rspike upper lower results4 if results4 ==1, lcolor(navy) || ///
rspike upper lower results4 if results4 ==2, lcolor(navy) || ///
rspike upper lower results4 if results4 ==3, lcolor(navy) || ///
rspike upper lower results4 if results4 ==4, lcolor(navy) || ///
rspike upper lower results4 if results4 ==5, lcolor(navy)  ///
ysc(range(-2 1)) ylab(-2(.5)1) ///
xsc(range(.5 5.5)) xlab(1(1)5) ///
yline(0) ysize(10) xsize(12) graphr(c(white)) ///
xti("Years between exposure and control incident") scale(.95) ///
```

```
legend(label(1 " Control," "{&Delta}=1 yr") ///
label(2 " Control," "{&Delta}=2 yr") ///
label(3 " Control," "{&Delta}=3 yr") ///
label(4 " Control," "{&Delta}=4 yr") ///
label(5 " Control," "{&Delta}=5 yr") ///
c(1) order(1 2 3 4 5) pos(3) size(small) ///
c(1) symx(4) region(lc(white))) ///
yti("Effect in 1K Euro ({&delta}{subscript: Salary})",
height(7)) ///
legend(off)

graph export $highdef/est2003_2011.png, replace width(3900)


**Reports marginal effects for period 2003-2011 in percent

gen upper2 = (results3+results2/(results1/results3)*1.96)*100
gen lower2 = (results3-results2/(results1/results3)*1.96)*100
replace results3 = results3*100

scatter results3 results4 , mcolor(navy) || ///
rspike upper2 lower2 results4, lcolor(navy) ///
ysc(range(-5 0)) ylab(-5(.5)0) ///
xsc(range(.5 5.5)) xlab(1(1)5) ///
yline(0) ysize(10) xsize(12) graphr(c(white)) ///
/*title("Percentage change in salary, 2003-10")*/ ///
yti("Salary change (in %)", height(7)) ///
xti("Years between exposure and control incident") scale(.95) ///
legend(label(1 " Control," "{&Delta}=1 yr") ///
label(2 " Control," "{&Delta}=2 yr") ///
label(3 " Control," "{&Delta}=3 yr") ///
label(4 " Control," "{&Delta}=4 yr") ///
label(5 " Control," "{&Delta}=5 yr") ///
c(1) order(1 2 3 4 5) pos(3) size(small) ///
c(1) symx(4) region(lc(white)))  legend(off)
/*
note("Marginal effects for exposure dummy across spacing of control
groups. Decrease " ///
"calculated by dividing {&delta} with the normalized control groups'
average salary " ///
"post-concussion. Control groups suffer concussions 1, 2, 3, 4, and
5 years (&Delta) after" ///
"the exposure group. Both control and exposure group are 30-49 years
of age when" ///
"exposure group suffers concussion. 95% confidence intervals.")*/

graph export $highdef/marginal2003_2011.png, replace width(3900)




*********************************************************************
***********
**
```

```
**               Results for individuals with at least highschool
**
**
*********************************************************************
***********


forvalue control_time=1/5{
        local end = 2012 // last incident year in data

        **build dataset for joint estimate across years
        forvalue count=2003/`end'{
                if `count'==2003{
                        use $data/
sample_control_`count'_`control_time'.dta, clear
                        gen time = `count' //incident year
indicator
                }
                else append using $data/
sample_control_`count'_`control_time'.dta
                replace time = `count' if time ==.

                **exclude individuals in years where they do not
appear in data,
                **due to either death or migration, as well as
periods from when
                **the control group sufer their concussion
                drop if merge ==1 | count > `control_time'-1


        }

        gen female = koen==2

        **Generate educational groups
        qui{
                gen edu =0
                replace edu = 1 if inrange(hffsp,20000000,39000000)
| ///
                                                            (hffsp
>40000000 & hffsp!=.)
        }

        keep if edu==1

        //build ident, so we can multivariate cluster for
individuals
        //who occur both as control and exposure during the period
(id)

        bysort pnr time: gen helpx = _n ==1
        gen id= sum(helpx)
        drop helpx help
```

```
        **Calculate number of observations for exposure and control
        count if count==0 & treatment ==1
        local Ntreated = r(N)
        count if count==0 & treatment ==0
        local Ncontrol = r(N)

        **generate concussion variable
        gen treat = inrange(count,0,`control_time'-1) & treatment
==1
        replace treat = time_from_incident if count ==0 & treatment
==1

        **Generate pre-concussion income difference        for
        **use in calculating marginal effects
        sum loenmv if count <0 & treatment ==0
        local control =r(mean)
        sum loenmv if count <0 & treatment ==1
        local treat =r(mean)
        sum loenmv if count>=0 & treatment ==0
        local control_post =r(mean)

        gen post = count >=0

        **estimate DiD model on salary
        xi: reghdfe loenmv treat, abs(alder female post time
treatment) cl(id pnr)

        if `control_time'==1 matrix results_edu = J(5,5,.) //
matrix to capture results
        if `control_time'==1 matrix results_p_edu = J(5,5,.) //
matrix to capture results


        matrix b = e(b)
        matrix V = e(V)
        local n = `control_time'

        matrix results_edu[`n',1]         = b[1,1] / 7466  //
capture beta results as 1K Euro
        matrix results_edu[`n',2]         = (V[1,1]^.5)/
7466    // capture standard error as 1K Euro
        matrix results_edu[`n',3] =       b[1,1]/(`control_post'-
(`control'-`treat'))
        matrix results_edu[`n',4] =       `n'


}

*********************************************************************
***********
**
**               Results for individuals with no high school+
**
**
```

```
*********************************************************************
***********


forvalue control_time=1/5{
        local end = 2012 // last incident year in data

        **build dataset for joint estimate across years
        forvalue count=2003/`end'{
                if `count'==2003{
                        use $data/
sample_control_`count'_`control_time'.dta, clear
                        gen time = `count' //incident year
indicator
                }
                else append using $data/
sample_control_`count'_`control_time'.dta
                replace time = `count' if time ==.

                **exclude individuals in years where they do not
appear in data,
                **due to either death or migration, as well as
periods from when
                **the control group sufer their concussion
                drop if merge ==1 | count > `control_time'-1


        }

        gen female = koen==2

        //build ident, so we can multivariate cluster for
individuals
        //who occur both as control and exposure during the period
(id)

        bysort pnr time: gen helpx = _n ==1
        gen id= sum(helpx)
        drop helpx help

        **Generate educational groups
        qui{
                gen edu =0
                replace edu = 1 if inrange(hffsp,20000000,39000000)
| ///
                                                                (hffsp
>40000000 & hffsp!=.)
        }

        keep if edu==0



        **Calculate number of observations for exposure and control
```

```
        count if count==0 & treatment ==1
        local Ntreated = r(N)
        count if count==0 & treatment ==0
        local Ncontrol = r(N)

        **generate concussion variable
        gen treat = inrange(count,0,`control_time'-1) & treatment
==1
        replace treat = time_from_incident if count ==0 & treatment
==1

        **Generate pre-concussion income difference        for
        **use in calculating marginal effects
        sum loenmv if count <0 & treatment ==0
        local control =r(mean)
        sum loenmv if count <0 & treatment ==1
        local treat =r(mean)
        sum loenmv if count>=0 & treatment ==0
        local control_post =r(mean)
        gen post = count >=0

        **estimate DiD model on salary
        xi: reghdfe loenmv treat, abs(alder female post time
treatment) cl(id pnr)

        if `control_time'==1 matrix results_noedu = J(5,5,.) //
matrix to capture results
        if `control_time'==1 matrix results_p_noedu = J(5,5,.) //
matrix to capture results


        matrix b = e(b)
        matrix V = e(V)
        local n = `control_time'

        matrix results_noedu[`n',1]       = b[1,1] / 7466  //
capture beta results as 1K Euro
        matrix results_noedu[`n',2]       = (V[1,1]^.5)/
7466    // capture standard error as 1K Euro
        matrix results_noedu[`n',3]       =       b[1,1]/
(`control_post'-(`control'-`treat'))
        matrix results_noedu[`n',4]       =       `n'



}
********************************************************************
***********
**
**      Draw figure for subgroups
**
**
********************************************************************
***********
```

```
local t= -.15

foreach x in noedu edu{
	svmat results_`x'
	replace results_`x'4= results_`x'4+`t'
	svmat results_p_`x'
	replace results_p_`x'4= results_p_`x'4+`t'

	gen upper`x' = results_`x'1+results_`x'2*1.96
	gen lower`x' = results_`x'1-results_`x'2*1.96

	gen upper2`x' = (results_`x'3+results_`x'2/(results_`x'1/
results_`x'3)*1.96)*100
	gen lower2`x' = (results_`x'3-results_`x'2/(results_`x'1/
results_`x'3)*1.96)*100
	replace results_`x'3 = results_`x'3*100

	gen upper_p_`x' = results_p_`x'1+results_p_`x'2*1.96
	gen lower_p_`x' = results_p_`x'1-results_p_`x'2*1.96

	local t = `t'+.1

}


keep results* upper* lower*
keep if _n <=5

**generate locals for figure

foreach x in noedu edu{
	if "`x'" == "nopay" local color = "navy"
	if "`x'" == "pay"	local color = "red"
	if "`x'" == "noedu" local color = "green"
	if "`x'" == "edu"	local color = "purple"

	local figure_`x' "scatter results_`x'1 results_`x'4,
mcolor(`color') || rspike upper`x' lower`x' results_`x'4,
lcolor(`color') vertical"
	if "`x'" == "nopay" local figure2_`x' "scatter results_`x'3
results_`x'4, mcolor(`color') || rspike upper2`x' lower2`x'
results_`x'4, lcolor(`color' )  vertical "
	else local figure2_`x' "scatter results_`x'3 results_`x'4,
mcolor(`color') || rspike upper2`x' lower2`x' results_`x'4,
lcolor(`color')  vertical "
	local figure_p_`x' "scatter results_p_`x'1 results_p_`x'4,
mcolor(`color') || rspike upper_p_`x' lower_p_`x' results_p_`x'4,
lcolor(`color')  vertical "
}


`figure_noedu' || `figure_edu'  ///
legend( ///
```

```
label(1 "Less than" "high school") ///
label(3 "At least" "high school") ///
c(1) order(1 3 ) pos(3) size(small) ///
c(1) symx(4) region(lc(white))) ///
ysc(range(-4 2)) ylab(-4(1)2) ///
xsc(range(.5 5.5)) xlab(1(1)5) ///
yline(0) ysize(10) xsize(12) graphr(c(white)) ///
///title("Parameter estimates across control group, 2003-10") ///
xti("Years between exposure and control incident") scale(.95) ///
yti("Effect in 1K Euro ({&delta}{subscript: Salary})",
height(7)) ///
/*note("Parameter estimates for exposure dummy across spacing of
control groups." ///
"Control groups suffer concussions 1, 2, 3, 4, and 5 years (&Delta)
after the exposure group." ///
"Both control and exposure group are 30-49 years of age when
exposure group suffers " ///
"concussion. 95% confidence intervals.")
*/

graph export $highdef/grouped_est2003_2011.png, replace width(3900)

preserve
```

```
`figure2_noedu' || `figure2_edu'  ///
legend( ///
label(1 "Less than" "high school") ///
label(3 "At least" "high school") ///
c(1) order(1 3 5) pos(3) size(small) ///
c(1) symx(4) region(lc(white))) ///
ysc(range(-12 3)) ylab(-12(3)3) ///
xsc(range(.5 5.5)) xlab(1(1)5) ///
yline(0) ysize(10) xsize(12) graphr(c(white)) ///
/// title("Percentage change in salary, 2003-10") ///
yti("Salary change (in %)", height(7)) ///
xti("Years between exposure and control incident") scale(.95) ///
/*note("Marginal effects for exposure dummy across spacing of
control groups." ///
"change calculated by {&delta} with the normalized control groups'
average" ///
"salary post-concussion. Control groups suffer concussions 1, 2, 3,
4, and 5 years (&Delta) after" ///
"the exposure group. Both control and exposure group are 30-49 years
of age when" ///
"exposure group suffers concussion.  95% confidence intervals.")*/

graph export $highdef/grouped_marginal2003_2011.png, replace
width(3900)

restore
```

```
*********************************************************************
***********
**
**                Results different age-groups
**
**
*********************************************************************
***********

forvalue y=20(5)55{
        forvalue control_time=1/5{
                local end = 2012 // last incident year in data

                **build dataset for joint estimate across years
                forvalue count=2003/`end'{
                        if `count'==2003{
                                use $data/
sample_control_`count'_`control_time'.dta, clear
                                gen time = `count' //incident year
indicator
                        }
                        else append using $data/
sample_control_`count'_`control_time'.dta
                        replace time = `count' if time ==.

                        **exclude individuals in years where they
do not appear in data,
                        **due to either death or migration, as
well as periods from when
                        **the control group sufer their concussion
                        drop if merge ==1 | count >
`control_time'-1


                }

                gen female = koen==2

                //build ident, so we can multivariate cluster for
individuals
                //who occur both as control and exposure during the
period (id)

                bysort pnr time: gen helpx = _n ==1
                gen id= sum(helpx)
                drop helpx help

                gen nopay = loenmv <1

                **Generate age group
                local z = `y'+4
                gen help = count == 0 & inrange(alder,`y',`z')
                bysort id: egen helpx =max(help)
```

```
                keep if helpx == 1
                drop helpx help



                **Calculate number of observations for exposure and
control
                count if count==0 & treatment ==1
                local Ntreated = r(N)
                count if count==0 & treatment ==0
                local Ncontrol = r(N)

                **generate concussion variable
                gen treat = inrange(count,0,`control_time'-1) &
treatment ==1
                replace treat = time_from_incident if count ==0 &
treatment ==1

                **Generate pre-concussion income difference
for
                **use in calculating marginal effects
                sum loenmv if count <0 & treatment ==0
                local control =r(mean)
                sum loenmv if count <0 & treatment ==1
                local treat =r(mean)
                sum loenmv if count>=0 & treatment ==0
                local control_post =r(mean)
                gen post = count >=0

                **estimate DiD model on salary
                xi: reghdfe loenmv treat, abs(alder female post
time treatment) cl(id pnr)

                if `control_time'==1 matrix results_`y' =
J(5,5,.) // matrix to capture results
                if `control_time'==1 matrix results_p_`y' =
J(5,5,.) // matrix to capture results


                matrix b = e(b)
                matrix V = e(V)
                local n = `control_time'

                matrix results_`y'[`n',1]       = b[1,1] /
7466    // capture beta results as 1K Euro
                matrix results_`y'[`n',2]       = (V[1,1]^.5)/
7466    // capture standard error as 1K Euro
                matrix results_`y'[`n',3] =     b[1,1]/
(`control_post'-(`control'-`treat'))
                matrix results_`y'[`n',4] =     `n'


        }
```

```
}



local t = -.15   //Jitter estimates along x-axis
forvalue x =20(5)55{
        svmat results_`x'
        replace results_`x'4= results_`x'4+`t'
        svmat results_p_`x'
        replace results_p_`x'4= results_p_`x'4+`t'

        gen upper`x' = results_`x'1+results_`x'2*1.96
        gen lower`x' = results_`x'1-results_`x'2*1.96

        gen upper2`x' = (results_`x'3+results_`x'2/(results_`x'1/
results_`x'3)*1.96)*100
        gen lower2`x' = (results_`x'3-results_`x'2/(results_`x'1/
results_`x'3)*1.96)*100
        replace results_`x'3 = results_`x'3*100

        gen upper_p_`x' = results_p_`x'1+results_p_`x'2*1.96
        gen lower_p_`x' = results_p_`x'1-results_p_`x'2*1.96

        local t = `t'+.1

}

keep results* upper* lower*
keep if _n <=5

**generate locals for figure

forvalue x =20(5)55{
        if `x' == 20 local color = "black"
        if `x' == 25 local color = "orange"
        if `x' == 30 local color = "navy"
        if `x' == 35 local color = "red"
        if `x' == 40 local color = "green"
        if `x' == 45 local color = "purple"
        if `x' == 50 local color = "sienna"
        if `x' == 55 local color = "teal"

        local figure_`x' "scatter results_`x'1 results_`x'4,
mcolor(`color') || rspike upper`x' lower`x' results_`x'4,
lcolor(`color') vertical"
        local figure2_`x' "scatter results_`x'3 results_`x'4,
mcolor(`color') || rspike upper2`x' lower2`x' results_`x'4,
lcolor(`color')  vertical "
        local figure_p_`x' "scatter results_p_`x'1 results_p_`x'4,
mcolor(`color') || rspike upper_p_`x' lower_p_`x' results_p_`x'4,
lcolor(`color')  vertical "
}

/**/
```

```
`figure_20' || `figure_25' || `figure_30' || `figure_35' ///
        || `figure_40' || `figure_45' || `figure_50' ||
`figure_55'  ///
legend( ///
label(1 "Age 20-24") ///
label(3 "Age 25-29") ///
label(5 "Age 30-34") ///
label(7 "Age 35-39") ///
label(9 "Age 40-44") ///
label(11 "Age 45-49") ///
label(13 "Age 50-54") ///
label(15 "Age 55-59") ///
c(1) order(1 3 5 7 9 11 13 15) pos(3) size(small) ///
c(1) symx(4) region(lc(white))) ///
ysc(range(-4 2)) ylab(-4(1)2) ///
xsc(range(.5 5.5)) xlab(1(1)5) ///
yline(0) ysize(10) xsize(12) graphr(c(white)) ///
/// title("Parameter estimates across control group, 2003-10") ///
xti("Years between exposure and control incident") scale(.95) ///
yti("Effect in 1K Euro ({&delta}{subscript: Salary})",
height(7)) ///
/*note("Parameter estimates for exposure dummy across spacing of
control groups." ///
"Control groups suffer concussions 1, 2, 3, 4, and 5 years (&Delta)
after the exposure group." ///
"Age group described age at time of exposure incident. 95%
confidence intervals.")*/

graph export $highdef/age_est2003_2011.png, replace width(3900)
*/

`figure2_20' || `figure2_25' || `figure2_30' || `figure2_35' ///
        || `figure2_40' || `figure2_45' || `figure2_50' ||
`figure2_55'  ///
legend( ///
label(1 "Age 20-24") ///
label(3 "Age 25-29") ///
label(5 "Age 30-34") ///
label(7 "Age 35-39") ///
label(9 "Age 40-44") ///
label(11 "Age 45-49") ///
label(13 "Age 50-54") ///
label(15 "Age 55-59") ///
c(1) order(1 3 5 7 9 11 13 15) pos(3) size(small) ///
c(1) symx(4) region(lc(white))) ///
ysc(range(-12 3)) ylab(-12(3)3) ///
xsc(range(.5 5.5)) xlab(1(1)5) ///
yline(0) ysize(10) xsize(12) graphr(c(white)) ///
yti("Salary change (in %)", height(7)) ///
xti("Years between exposure and control incident") scale(.95)


graph export $highdef/age_marginal2003_2011.png, replace width(3900)
```

```
`figure_p_30' || `figure_p_35' || `figure_p_40' ||
`figure_p_45'  ///  ///
legend(label(1 "Age 30-34") ///
label(3 "Age 35-39") ///
label(5 "Age 40-44") ///
label(7 "Age 45-49") ///
c(1) order(1 3 5 7) pos(3) size(small) ///
c(1) symx(4) region(lc(white))) ///
ysc(range(-.02 .050)) ylab(-0.02(.01)0.05) ///
xsc(range(.5 5.5)) xlab(1(1)5) ///
yline(0, lcolor(black)) ysize(10) xsize(12) graphr(c(white)) ///
title("Parameter estimates across control group, 2003-10") ///
yti("Effect on Pr(Salary=0)", height(7)) ///
xti("Years between exposure and control incident ({&Delta})")
scale(.95) ///
note("Parameter estimates for exposure dummy across spacing of
control groups." ///
"Control groups suffer concussions 1, 2, 3, 4, and 5 (&Delta) years
after the exposure group." ///
"Age group described age at time of exposure incident. 95%
confidence intervals.")

graph export $highdef/age_nopay2003_2011.png, replace width(3900)


*********************************************************************
***********
**
**                Results accross gender
**
**
*********************************************************************
***********

forvalue y=0/1{
        forvalue control_time=1/5{
                local end = 2012 // last incident year in data

                **build dataset for joint estimate across years
                forvalue count=2003/`end'{
                        if `count'==2003{
                                use $data/
sample_control_`count'_`control_time'.dta, clear
                                gen time = `count' //incident year
indicator
                        }
                        else append using $data/
sample_control_`count'_`control_time'.dta
                        replace time = `count' if time ==.

                        **exclude individuals in years where they
do not appear in data,
                        **due to either death or migration, as
well as periods from when
```

```
                        **the control group sufer their concussion
                        drop if merge ==1 | count >
`control_time'-1


                }

                gen female = koen==2

                //build ident, so we can multivariate cluster for
individuals
                //who occur both as control and exposure during the
period (id)

                bysort pnr time: gen helpx = _n ==1
                gen id= sum(helpx)
                drop helpx help

                gen nopay = loenmv <1

                **Generate age group
                local z = `y'+4
                gen help = count == 0 & female==`y'
                bysort id: egen helpx =max(help)
                keep if helpx == 1
                drop helpx help



                **Calculate number of observations for exposure and
control
                count if count==0 & treatment ==1
                local Ntreated = r(N)
                count if count==0 & treatment ==0
                local Ncontrol = r(N)

                **generate concussion variable
                gen treat = inrange(count,0,`control_time'-1) &
treatment ==1
                replace treat = time_from_incident if count ==0 &
treatment ==1

                **Generate pre-concussion income difference
for
                **use in calculating marginal effects
                sum loenmv if count <0 & treatment ==0
                local control =r(mean)
                sum loenmv if count <0 & treatment ==1
                local treat =r(mean)
                sum loenmv if count>=0 & treatment ==0
                local control_post =r(mean)
                gen post = count >=0
```

```
                **estimate DiD model on salary
                xi: reghdfe loenmv treat, abs(alder post time 
treatment) cl(id pnr)

                if `control_time'==1 matrix results_`y' = 
J(5,5,.) // matrix to capture results
                if `control_time'==1 matrix results_p_`y' = 
J(5,5,.) // matrix to capture results


                matrix b = e(b)
                matrix V = e(V)
                local n = `control_time'

                matrix results_`y'[`n',1]         = b[1,1] / 
7466    // capture beta results as 1K Euro
                matrix results_`y'[`n',2]         = (V[1,1]^.5)/
7466    // capture standard error as 1K Euro
                matrix results_`y'[`n',3] =       b[1,1]/
(`control_post'-(`control'-`treat'))
                matrix results_`y'[`n',4] =       `n'


        }
}



local t = -.05   //Jitter estimates along x-axis
foreach x in 0 1{
        svmat results_`x'
        replace results_`x'4= results_`x'4+`t'
        svmat results_p_`x'
        replace results_p_`x'4= results_p_`x'4+`t'

        gen upper`x' = results_`x'1+results_`x'2*1.96
        gen lower`x' = results_`x'1-results_`x'2*1.96

        gen upper2`x' = (results_`x'3+results_`x'2/(results_`x'1/
results_`x'3)*1.96)*100
        gen lower2`x' = (results_`x'3-results_`x'2/(results_`x'1/
results_`x'3)*1.96)*100
        replace results_`x'3 = results_`x'3*100

        gen upper_p_`x' = results_p_`x'1+results_p_`x'2*1.96
        gen lower_p_`x' = results_p_`x'1-results_p_`x'2*1.96

        local t = `t'+.1

}

keep results* upper* lower*
keep if _n <=5
```

```
**generate locals for figure

foreach x in 0 1{
        if `x' == 0 local color = "red"
        if `x' == 1 local color = "green"

        local figure_`x' "scatter results_`x'1 results_`x'4,
mcolor(`color') || rspike upper`x' lower`x' results_`x'4,
lcolor(`color') vertical"
        local figure2_`x' "scatter results_`x'3 results_`x'4,
mcolor(`color') || rspike upper2`x' lower2`x' results_`x'4,
lcolor(`color')  vertical "
        local figure_p_`x' "scatter results_p_`x'1 results_p_`x'4,
mcolor(`color') || rspike upper_p_`x' lower_p_`x' results_p_`x'4,
lcolor(`color')  vertical "
}


`figure_0' || `figure_1'  ///
legend(label(1 "Men") ///
label(3 "Women") ///
c(1) order(1 3) pos(3) size(small) ///
c(1) symx(4) region(lc(white))) ///
ysc(range(-4 2)) ylab(-4(1)2) ///
xsc(range(.5 5.5)) xlab(1(1)5) ///
yline(0) ysize(10) xsize(12) graphr(c(white)) ///
/// title("Parameter estimates across control group, 2003-10") ///
xti("Years between exposure and control incident") scale(.95) ///
yti("Effect in 1K Euro ({&delta}{subscript: Salary})",
height(7)) ///
/*note("Parameter estimates for exposure dummy across spacing of
control groups." ///
"Control groups suffer concussions 1, 2, 3, 4, and 5 years (&Delta)
after the exposure group." ///
"95% confidence intervals.")*/

graph export $highdef/gender_est2003_2011.png, replace width(3900)

`figure2_0' || `figure2_1'   ///
legend( ///
label(1 "Men") ///
label(3 "Women") ///
c(1) order(1 3 5 7) pos(3) size(small) ///
c(1) symx(4) region(lc(white))) ///
ysc(range(-12 3)) ylab(-12(3)3) ///
xsc(range(.5 5.5)) xlab(1(1)5) ///
yline(0) ysize(10) xsize(12) graphr(c(white)) ///
/// title("Percentage change in salary, 2003-10") ///
yti("Salary change (in %)", height(7)) ///
xti("Years between exposure and control incident") scale(.95) ///
/* note("Parameter estimates for exposure dummy across spacing of
control groups." ///
"Control groups suffer concussions 1, 2, 3, 4, and 5 years (&Delta)
after the exposure group." ///
```

```
"95% confidence intervals.")*/

graph export $highdef/gender_marginal2003_2011.png, replace
width(3900)

`figure_p_0' || `figure_p_1'   ///
legend( ///
label(1 "Men") ///
label(3 "Women") ///
c(1) order(1 3 5 7) pos(3) size(small) ///
c(1) symx(4) region(lc(white))) ///
ysc(range(-.02 .050)) ylab(-0.02(.01)0.05) ///
xsc(range(.5 5.5)) xlab(1(1)5) ///
yline(0, lcolor(black)) ysize(10) xsize(12) graphr(c(white)) ///
/// title("Parameter estimates across control group, 2003-10") ///
yti("Effect on Pr(Salary=0)", height(7)) ///
xti("Years between exposure and control incident ({&Delta})")
scale(.95) ///
/*note("Parameter estimates for exposure dummy across spacing of
control groups." ///
"Control groups suffer concussions 1, 2, 3, 4, and 5 years (&Delta)
after the exposure group." ///
"95% confidence intervals.")*/

graph export $highdef/gender_nopay2003_2011.png, replace width(3900)


********************************************************************
************
**
**                       Generate descriptive figures of wage
development for exposure
**                        and control group
**
**
********************************************************************
************


 forvalue control_time=1/5{
        local end = 2012 // last incident year in data

        **build dataset for joint estimate across years
        forvalue count=2003/`end'{
                if `control_time' ==1 & `count'==2003{
                        use $data/
sample_control_`count'_`control_time'.dta, clear
                        gen time = `count' //incident year
indicator
                }
                else append using $data/
sample_control_`count'_`control_time'.dta

                **Drops exposure-group already in the data
```

```
                 if `control_time' > 1 drop if time ==. & treatment
==1
                 replace time = `count' if time ==.
                 replace control1 = `control_time' if
control`control_time'==1
                 if `control_time' > 1 drop control`control_time'

                 **exclude individuals in years where they do not
appear in data,
                 **due to either death or migration, as well as
periods from when
                 **the control group suffer their concussion

        }

}

**Generate mean salary for each period relative to exposure groups
concussion
**separately for exposure and each control group

bysort control1 count: egen mean_loen = mean(loenmv)

**Generate mean Pr(sal= for each period relative to exposure groups
concussion
**separately for exposure and each control group

gen no_lon = loenmv < 1

bysort control1 count: egen mean_no_lon = mean(no_lon)

**generate group size
bysort control1 count: gen Ncount=_N if count ==0

**generate pre-exposure mean levels for normalization
gen pre = count <0
bysort control1 pre: egen pre_mean_loen = mean(loenmv) if pre==1
bysort control1 pre: egen pre_mean_no_lon = mean(no_lon) if pre==1


**reduce data set size
bysort control1 count: keep if _n ==1

keep count mean* pre_* control1 Ncount

**standardize to 1k euro
replace mean_loen = mean_loen/7466
replace pre_mean_loen = pre_mean_loen/7466

sort control1 count


**Pre-tratment normalization of salary
```

```
gen norm_mean_lon =mean_loen
gen norm_mean_no_lon =mean_no_lon

forvalue t = 1/5{
        qui sum pre_mean_loen if control == 0
        local treat = r(mean)
        qui sum pre_mean_loen if control == `t'
        local control = r(mean)

        **normalize with pre-concussion difference
        qui replace norm_mean_lon = mean_loen - (`control'-`treat')
if control ==`t'

        qui sum pre_mean_no_lon if control == 0
        local treat = r(mean)
        qui sum pre_mean_no_lon if control == `t'
        local control = r(mean)

        **normalize with pre-concussion difference
        qui replace norm_mean_no_lon = mean_no_lon - (`control'-
`treat') if control ==`t'

}

**local indicators of group sizes
forvalue t=0/5{
        qui sum Ncount if control == `t'
        local C`t' = r(mean)
}


graph twoway ///
        connect mean_l count if control1== 0, ///
        lcolor(black) mcolor(black) || ///
        connect mean_l count if control1== 1, ///
        lcolor(blue)  mcolor(blue) || ///
        connect mean_l count if control1== 2, ///
        lcolor(green) mcolor(green) || ///
        connect mean_l count if control1== 3, ///
        lcolor(purple) mcolor(purple) || ///
        connect mean_l count if control1== 4, ///
        lcolor(red) mcolor(red) || ///
        connect mean_l count if control1== 5, ///
        lcolor(orange) mcolor(orange) ///
        legend( ///
                label(1 "Exposure" ///
                                 "N=`C0'") ///
                label(2 "Control {&Delta}=1" ///
                                 "N=`C1'") ///
                label(3 "Control {&Delta}=2" ///
                                 "N=`C2'") ///
                label(4 "Control {&Delta}=3" ///
                                 "N=`C3'") ///
                label(5 "Control {&Delta}=4" ///
```

```
                                 "N=`C4'") ///
                label(6 "Control {&Delta}=5" ///
                                 "N=`C5'") ///
                c(1) order(1 2 3 4 5 6) pos(3) size(small) ///
                c(1) symx(4) region(lc(white))) ///
        ysc(range(26 33)) ylab(26(1)33) ///
        xsc(range(-5 5)) xlab(-5(1)5) ///
        xline(0, lcolor(red)) ysize(10) xsize(12)
graphr(c(white)) ///
///     title("Impact of Concussion on Salary") ///
        yti("Deflated Salaried Income in 1K EUR", height(7)) ///
        xti("Years since exposure group concussion") scale(.95) ///
/*      note("Control groups suffer concussion 1, 2, 3, 4, and 5
years (&Delta) after exposure group." ///
                 "Both control and exposure group at 30-49 years of
age at beginning of year =0." ///
                 "Vertical line indicates time of exposure group
concussion.")*/

graph export $highdef/FigureS2.png, replace width(3900)


graph twoway ///
        connect norm_mean_l count if control1== 1, ///
        lcolor(blue)  mcolor(blue) || ///
        connect norm_mean_l count if control1== 2, ///
        lcolor(green) mcolor(green) || ///
        connect norm_mean_l count if control1== 3, ///
        lcolor(purple) mcolor(purple) || ///
        connect norm_mean_l count if control1== 4, ///
        lcolor(red) mcolor(red) || ///
        connect norm_mean_l count if control1== 5, ///
        lcolor(orange) mcolor(orange) || ///
        connect mean_l count if control1== 0, ///
        lcolor(black) mcolor(black)  ///
        legend( ///
                label(1 "Control {&Delta}=1" ///
                                 "N=`C1'") ///
                label(2 "Control {&Delta}=2" ///
                                 "N=`C2'") ///
                label(3 "Control {&Delta}=3" ///
                                 "N=`C3'") ///
                label(4 "Control {&Delta}=4" ///
                                 "N=`C4'") ///
                label(5 "Control {&Delta}=5" ///
                                 "N=`C5'") ///
                label(6 "Exposure" ///
                                 "N=`C0'") ///
                c(1) order(6 1 2 3 4 5) pos(3) size(small) ///
                c(1) symx(4) region(lc(white))) ///
        ysc(range(26 31)) ylab(26(1)31) ///
        xsc(range(-5 5)) xlab(-5(1)5) ///
        xline(0, lcolor(red)) ysize(10) xsize(12)
graphr(c(white)) ///
```

```
///     title("Impact of Concussion on Salary") ///
        yti("Deflated Norm. Salaried Income in 1K EUR",
height(7)) ///
        xti("Years since exposure group's concussion") scale(.95)
        /*///
        note("Control groups suffer concussion 1, 2, 3, 4, and 5
years (&Delta) after exposure group." ///
                "Both control and exposure group at 30-49 years of
age at beginning of year =0." ///
                "Vertical line indicates time of exposure group
concussion." ///
                "Salary levels normalized with pre-concussion level
difference between" ///
                "each control group and the exposure group")*/

graph export $highdef/Figure2.png, replace width(3900)


graph twoway ///
        connect mean_no_l count if control1== 0, ///
        lcolor(black) mcolor(black) || ///
        connect mean_no_l count if control1== 1, ///
        lcolor(blue)  mcolor(blue) || ///
        connect mean_no_l count if control1== 2, ///
        lcolor(green) mcolor(green) || ///
        connect mean_no_l count if control1== 3, ///
        lcolor(purple) mcolor(purple) || ///
        connect mean_no_l count if control1== 4, ///
        lcolor(red) mcolor(red) || ///
        connect mean_no_l count if control1== 5, ///
        lcolor(orange) mcolor(orange) ///
        legend( ///
                label(1 "Exposure" ///
                                "N=`C0'") ///
                label(2 "Control {&Delta}=1" ///
                                "N=`C1'") ///
                label(3 "Control {&Delta}=2" ///
                                "N=`C2'") ///
                label(4 "Control {&Delta}=3" ///
                                "N=`C3'") ///
                label(5 "Control {&Delta}=4" ///
                                "N=`C4'") ///
                label(6 "Control {&Delta}=5" ///
                                "N=`C5'") ///
                c(1) order(1 2 3 4 5 6) pos(3) size(small) ///
                c(1) symx(4) region(lc(white))) ///
        ysc(range(.2 .325)) ylab(.2(.025).325) ///
        xsc(range(-5 5)) xlab(-5(1)5) ///
        xline(0, lcolor(red)) ysize(10) xsize(12)
graphr(c(white)) ///
///     title("Impact of Concussion on Prob(Salary=0)") ///
        yti("Prob(Salary=0)", height(7)) ///
        xti("Years since exposure group concussion") scale(.95) ///
/*      note("Control groups suffer concussion 1, 2, 3, 4, and 5
```

```
years (&Delta) after exposure group." ///
                  "Both control and exposure group at 30-49 years of
age at beginning of year =0." ///
                  "Vertical line indicates time of exposure group
concussion.")*/

graph export $highdef/FigureS3.png, replace width(3900)

graph twoway ///
        connect norm_mean_no_l count if control1== 1, ///
        lcolor(blue)  mcolor(blue) || ///
        connect norm_mean_no_l count if control1== 2, ///
        lcolor(green) mcolor(green) || ///
        connect norm_mean_no_l count if control1== 3, ///
        lcolor(purple) mcolor(purple) || ///
        connect norm_mean_no_l count if control1== 4, ///
        lcolor(red) mcolor(red) || ///
        connect norm_mean_no_l count if control1== 5, ///
        lcolor(orange) mcolor(orange) || ///
        connect mean_no_l count if control1== 0, ///
        lcolor(black) mcolor(black) ///
        legend( ///
                label(1 "Control {&Delta}=1" ///
                                "N=`C1'") ///
                label(2 "Control {&Delta}=2" ///
                                "N=`C2'") ///
                label(3 "Control {&Delta}=3" ///
                                "N=`C3'") ///
                label(4 "Control {&Delta}=4" ///
                                "N=`C4'") ///
                label(5 "Control {&Delta}=5" ///
                                "N=`C5'") ///
                label(6 "Exposure" ///
                                "N=`C0'") ///
                c(1) order(6 1 2 3 4 5) pos(3) size(small) ///
                c(1) symx(4) region(lc(white))) ///
        ysc(range(.2 .325)) ylab(.2(.025).325) ///
        xsc(range(-5 5)) xlab(-5(1)5) ///
        xline(0, lcolor(red)) ysize(10) xsize(12)
graphr(c(white)) ///
///     title("Impact of Concussion on Prob(Salary=0)") ///
        yti("Norm. Prob(Salary=0)", height(7)) ///
        xti("Years since exposure group concussion") scale(.95) ///
/*      note("Control groups suffer concussion 1, 2, 3, 4, and 5
years (&Delta) after exposure group." ///
                  "Both control and exposure group at 30-49 years of
age at beginning of year =0." ///
                  "Vertical line indicates time of exposure group
concussion." ///
                  "Probability levels normalized with pre-concussion
level difference between" ///
                  "each control group and the exposure group")*/

graph export $highdef/Figure2A.png, replace width(3900)
```

```
********************************************************************
************
**
**      Effects across the salary distribution
**
**
**
********************************************************************
************


forvalue control_time=5/5{
        local end = 2012 // last incident year in data

        **build dataset for joint estimate across years
        forvalue count=2003/`end'{
                if `count'==2003{
                        use $data/
sample_control_`count'_`control_time'.dta, clear
                        gen time = `count' //incident year
indicator
                }
                else append using $data/
sample_control_`count'_`control_time'.dta
                replace time = `count' if time ==.

                **exclude individuals in years where they do not
appear in data,
                **due to either death or migration, as well as
periods from when
                **the control group sufer their concussion
                drop if merge ==1 | count > `control_time'-1
        }

        gen female = koen==2
        qui{
                gen edu =0
                replace edu = 1 if inrange(hffsp,20000000,39000000)
| ///
                                                            (hffsp
>40000000 & hffsp!=.)
        }

        //build ident, so we can multivariate cluster for
individuals
        //who occur both as control and exposure during the period
(id)
```

```
        bysort pnr time: gen helpx = _n ==1
        gen id= sum(helpx)
        drop helpx


        **generate concussion variable
        gen treat = inrange(count,0,`control_time'-1) & treatment
==1
        replace treat = time_from_incident if count ==0 & treatment
==1

        **effect across salary distribution
        if `control_time'==5 matrix results = J(81,5,.) // matrix
to capture results

        local n = `control_time'
        gen post = count >=0
        replace count = count+6
        local v =0

        qui{
                forvalue t= 0(14932)1045240{
                        local top = 42
                        local v =1+`v'
                        if `n' ==5 matrix results[`v',1]= `t'
                        gen D = personindk <=`t'
                        reg D treat i.treatment i.post i.edu
i.alder i.female i.time, cl(pnr)
                        matrix V = e(V)
                        matrix b= e(b)
                        matrix results[`v',2]    = b[1,1]
                        margins, at(treat= (0 1) treatment=1
post=1)
                        matrix results[`v',5]    = V[1,1]^.5
                        matrix M = r(b)
                        matrix results[`v',4]    = M[1,2] //
                        matrix results[`v',3]    = M[1,1] //
                        drop D
                }
        }
}

svmat results

gen l5 = results2-1.96*results5
gen u5 = results2+1.96*results5


replace results1 = results1/7466

gen dif6 = results4-results3
gen udif6 = u5
gen ldif6 = l5
```

```
gr two rline udif6 ldif6 results1, ///
        yaxis(2) color(gray) lp(dash) ylab(0(.01).04, axis(2))
ysc(range(-.006 .04) axis(2))     || ///
        line dif6 results1, yaxis(2) yline(0, axis(2)) ///
        lcolor(black)  ylab(0(.01).04)  ysc(range(-.006 .04)
axis(2)) || ///
        line results4 results1, yaxis(1) lcolor(red) || ///
        line results3 results1, yaxis(1) lcolor(blue) ///
         ysc(range(0 1)) ylab(0(.1)1, nogrid) xlab(0(10)140)
xsc(range(0 140)) ///
        ysize(10) xsize(12) graphr(c(white)) ///
        xti("Total Income in 1K Euro") scale(1) ///
        yti("Effect of Concussion on Pr(Total Income < X)",
axis(2)) ///
        yti("{&Phi}(Total Income)",  axis(1)) ///
        legend( ///
        label(2 "Effect of concussion (left axis)") ///
        label(3 "Concussion income distribution (right axis)") ///
        label(4 "Counterfactual income distribution (right
axis)") ///
        c(1) order(2 3 4) pos(6) size(small) ///
                         symx(4) region(lc(white)))



graph export [home]\highdef\l5_income.png,  replace width(3900)

cap graph drop g1 g2

gr two ///
        line results4 results1, yaxis(1) lcolor(red) || ///
        line results3 results1, yaxis(1) lcolor(blue) ///
         ysc(range(.2 1)) ylab(0(.1)1) xlab(0(20)140,
labs(small)) ///
         xsc(range(0 140)) ///
        ysize(10) xsize(10) graphr(c(white)) ///
        xti("Total Income in 1K Euro") scale(1) ///
        yti("Cumulative Distribution of Total Income",
axis(1)) ///
        legend( ///
        label(1 "Observed Post-Concussion Total Income
Distribution") ///
        label(2 "Counterfactual No-Concussion Total Income
Distribution") ///
        c(1) order(1 2 4) pos(6) size(small) ///
                         symx(4) region(lc(white))) , name(g1)

gr two rline udif6 ldif6 results1, ///
        color(gray) lp(dash) ysc(range(-.002 .035)) || ///
        line dif6 results1,  yline(0) ///
        lcolor(black)  ylab(0(.01).0325)  ysc(range(-.002 .
035)) ///
        xlab(0(20)140, labs(small)) xsc(range(0 140))      ///
        ysize(10) xsize(10) graphr(c(white)) ///
```

```
        xti("Total Income in 1K Euro (X)") scale(1) ///
        yti("Effect of Concussion on Pr(Total Income < X)") ///
                legend( label(1 "95% CI") ///
        label(2 "Effect of concussion") ///
        c(1) order(2 1) pos(6) size(small) ///
                         symx(4) region(lc(white))) , name(g2)

graph combine g1 g2      ,graphr(c(white))

graph export "[home]\highdef\figure s2.tif",  replace width(1000)



********************************************************************
************
**
**      Effects across the salary distribution
**
**
**
********************************************************************
************


forvalue control_time=5/5{
        local end = 2012 // last incident year in data

        **build dataset for joint estimate across years
        forvalue count=2003/`end'{
                if `count'==2003{
                        use $data/
sample_control_`count'_`control_time'.dta, clear
                        gen time = `count' //incident year
indicator
                }
                else append using $data/
sample_control_`count'_`control_time'.dta
                replace time = `count' if time ==.

                **exclude individuals in years where they do not
appear in data,
                **due to either death or migration, as well as
periods from when
                **the control group sufer their concussion
                drop if merge ==1 | count > `control_time'-1
        }

        gen female = koen==2
        qui{
                gen edu =0
                replace edu = 1 if inrange(hffsp,20000000,39000000)
| ///
                                                            (hffsp
>40000000 & hffsp!=.)
```

```
	}

	//build ident, so we can multivariate cluster for
individuals
	//who occur both as control and exposure during the period
(id)

	bysort pnr time: gen helpx = _n ==1
	gen id= sum(helpx)
	drop helpx


	**generate concussion variable
	gen treat = inrange(count,0,`control_time'-1) & treatment
==1
	replace treat = time_from_incident if count ==0 & treatment
==1

	**effect across salary distribution
	if `control_time'==5 matrix results = J(81,5,.) // matrix
to capture results

	local n = `control_time'
	gen post = count >=0
	replace count = count+6
	local v =0

	**Estimate Pr(salary < X) across income distribution
	qui{
		forvalue t= 0(14932)895950{
			local top = 42
			local v =1+`v'
			if `n' ==5 matrix results[`v',1]= `t'
			gen D = loenmv <=`t'
			reg D treat i.treatment i.post i.edu
i.alder i.female i.time, cl(pnr)
			matrix V = e(V)
			matrix b= e(b)
			matrix results[`v',2]    = b[1,1]
			margins, at(treat= (0 1) treatment=1
post=1)
			matrix results[`v',5]    = V[1,1]^.5
			matrix M = r(b)
			matrix results[`v',4]    = M[1,2] //
			matrix results[`v',3]    = M[1,1] //
			drop D
		}
	}
}

svmat results

*Generate 95% confidence intervals
gen l5 = results2-1.96*results5
```

```
gen u5 = results2+1.96*results5

*Correct to €
replace results1 = results1/7466

*Obtain difference between observed and counterfactual wage
distribution
gen dif6 = results4-results3
gen udif6 = u5
gen ldif6 = l5

gr two rline udif6 ldif6 results1, ///
        yaxis(2) color(gray) lp(dash) ysc(range(-.006 .04) axis(2))
|| ///
        line dif6 results1, yaxis(2) yline(0, axis(2)) ///
        lcolor(black)  ylab(0(.01).0325)  ysc(range(-.006 .04)
axis(2)) || ///
        line results4 results1, yaxis(1) lcolor(red) || ///
        line results3 results1, yaxis(1) lcolor(blue) ///
         ysc(range(0 1)) ylab(0(.1)1, nogrid) xlab(0(10)120)
xsc(range(0 120)) ///
        ysize(10) xsize(12) graphr(c(white)) ///
        xti("Salary in 1K Euro") scale(1) ///
        yti("Effect of Concussion on Pr(Salary < X)", axis(2)) ///
        yti("{&Phi}(Salary)",  axis(1)) ///
        legend( ///
        label(2 "Effect of concussion (left axis)") ///
        label(3 "Concussion salary distribution (right axis)") ///
        label(4 "Counterfactual salary distribution (right
axis)") ///
        c(1) order(2 3 4) pos(6) size(small) ///
                          symx(4) region(lc(white)))

cap graph drop g1 g2

gr two ///
        line results4 results1, yaxis(1) lcolor(red) || ///
        line results3 results1, yaxis(1) lcolor(blue) ///
         ysc(range(.2 1)) ylab(0.2(.1)1) xlab(0(10)120,
labs(small)) ///
         xsc(range(0 120)) ///
        ysize(10) xsize(10) graphr(c(white)) ///
        xti("Salary in 1K Euro") scale(1) ///
        yti("Cumulative Distribution of Salary",  axis(1)) ///
        legend( ///
        label(1 "Observed Post-Concussion Salary Distribution") ///
        label(2 "Counterfactual No-Concussion Salary
Distribution") ///
        c(1) order(1 2 4) pos(6) size(small) ///
                          symx(4) region(lc(white))) , name(g1)

gr two rline udif6 ldif6 results1, ///
        color(gray) lp(dash) ysc(range(-.002 .035)) || ///
        line dif6 results1,  yline(0) ///
```

```
        lcolor(black)  ylab(0(.01).0325)  ysc(range(-.002 .
035)) ///
        xlab(0(10)120, labs(small)) xsc(range(0 120))      ///
        ysize(10) xsize(10) graphr(c(white)) ///
        xti("Salary in 1K Euro (X)") scale(1) ///
        yti("Effect of Concussion on Pr(Salary < X)") ///
                legend( label(1 "95% CI") ///
        label(2 "Effect of concussion") ///
        c(1) order(2 1) pos(6) size(small) ///
                         symx(4) region(lc(white))) , name(g2)

graph combine g1 g2      ,graphr(c(white))

graph export "[home]\highdef\figure 3.tif",  replace width(1000)




**ESTIMATE SICK LEAVE

forvalue control_time=1/5{
        local end = 2012 // last incident year in data
        if `control_time' ==1    eststo clear

        **build dataset for joint estimate across years
        forvalue count=2003/`end'{
                if `count'==2003{
                        use $data/
sample_control_`count'_`control_time'.dta, clear
                        gen time = `count' //incident year
indicator
                }
                else append using $data/
sample_control_`count'_`control_time'.dta
                replace time = `count' if time ==.

                **exclude individuals in years where they do not
appear in data,
                **due to either death or migration, as well as
periods from when
                **the control group sufer their concussion
                drop if merge ==1 | count > `control_time'-1
        }

        merge m:1 pnr t using $data/temp.dta, keep(1 3) nogen
        replace share = 0 if share==.

        gen female = koen==2

        //build ident, so we can multivariate cluster for
individuals
        //who occur both as control and exposure during the period
(id)
```

```
        bysort pnr time: gen helpx = _n ==1
        gen id= sum(helpx)
        drop helpx


        **Generate educational groups
        qui{
                gen edu =0
                replace edu = 1 if inrange(hffsp,20000000,39000000)
| ///
                                                                        (hffsp
>40000000 & hffsp!=.)
        }


        **Calculate number of observations for exposure and control
        count if count==0 & treatment ==1
        local Ntreated = r(N)
        count if count==0 & treatment ==0
        local Ncontrol = r(N)

        **generate concussion variable
        gen treat = inrange(count,0,`control_time'-1) & treatment
==1
        replace treat = time_from_incident if count ==0 & treatment
==1

        **Generate pre-concussion income difference        for
        **use in calculating marginal effects
        sum loenmv if count <0 & treatment ==0
        local control =r(mean)
        sum loenmv if count <0 & treatment ==1
        local treat =r(mean)
        sum loenmv if count>=0 & treatment ==0
        local control_post =r(mean)

        forvalue t=-4/4{
                local n = `t'*-1
                if `t' < -1 gen T_`n' = treatment ==1 & count ==`t'
                if `t' > -1 gen T`t' = treatment ==1 & count ==`t'

        }

        **estimate DiD model on salary
        reghdfe share T*, abs(alder female count time treatment
edu) cl(pnr  id)
        eststo est1_`control_time'
        if `control_time'==1 matrix results = J(5,5,.) // matrix to
capture results
        if `control_time'==1 matrix results_p = J(5,5,.) // matrix
to capture results
```

```
        matrix b = e(b)
        matrix V = e(V)
        local n = `control_time'

        matrix results[`n',1]    = b[1,1] / 7466  // capture beta 
results as 1K Euro
        matrix results[`n',2]    = (V[1,1]^.5)/7466       // 
capture standard error as 1K Euro
        matrix results[`n',3]    =       b[1,1]/(`control_post'-
(`control'-`treat'))
        matrix results[`n',4]    =       `n'

        gen no_share = share >0 //dummy for no salary


        **Generate pre-concussion probability difference   for 
        **use in calculating marginal effects
        sum no_share if count <0 & treatment ==0
        local control =r(mean)
        sum no_share if count <0 & treatment ==1
        local treat =r(mean)
        sum no_share if count>=0 & treatment ==0
        local control_post =r(mean)

        **estimate DiD LP-model on P(salary=0)
        xi: reghdfe no_share T*, abs(alder female count time 
treatment edu) cl(pnr  id)
        eststo est2_`control_time'
        matrix b =e(b)   //regression coefficient
        matrix V = e(V)  // standard error^2

        matrix results_p[`n',1]  =       b[1,1]
        matrix results_p[`n',2]  =       V[1,1]^.5
        matrix results_p[`n',3]  =       b[1,1]/(`control_post'-
(`control'-`treat'))
        matrix results_p[`n',4]  =       `n'

}

esttab est1_* using [home]/tables/dynamic_share1.rtf, ///
        replace se(3) b(3) compress nogap star(+ .1 * .05 ** .01 
*** .001) ///
        keep(T*)

esttab est2_* using [home]/tables/dynamic_share2.rtf, ///
        replace se(3) b(3) compress nogap star(+ .1 * .05 ** .01 
*** .001) ///
        keep(T*)

**ESTIMATE SICK LEAVE

forvalue control_time=1/5{
        local end = 2012 // last incident year in data
        if `control_time' ==1    eststo clear
```

```
        **build dataset for joint estimate across years
        forvalue count=2003/`end'{
                if `count'==2003{
                        use $data/
sample_control_`count'_`control_time'.dta, clear
                        gen time = `count' //incident year
indicator
                }
                else append using $data/
sample_control_`count'_`control_time'.dta
                replace time = `count' if time ==.

                **exclude individuals in years where they do not
appear in data,
                **due to either death or migration, as well as
periods from when
                **the control group sufer their concussion
                drop if merge ==1 | count > `control_time'-1
        }

        merge m:1 pnr t using $data/temp2.dta, keep(1 3) nogen
        replace syg_barsel_13= 0 if syg_barsel_13==.

        gen female = koen==2

        //build ident, so we can multivariate cluster for
individuals
        //who occur both as control and exposure during the period
(id)

        bysort pnr time: gen helpx = _n ==1
        gen id= sum(helpx)
        drop helpx


        **Generate educational groups
        qui{
                gen edu =0
                replace edu = 1 if inrange(hffsp,20000000,39000000)
| ///
                                                                (hffsp
>40000000 & hffsp!=.)
        }


        **Calculate number of observations for exposure and control
        count if count==0 & treatment ==1
        local Ntreated = r(N)
        count if count==0 & treatment ==0
        local Ncontrol = r(N)

        **generate concussion variable
        gen treat = inrange(count,0,`control_time'-1) & treatment
```

```
==1
        replace treat = time_from_incident if count ==0 & treatment
==1

        **Generate pre-concussion income difference       for
        **use in calculating marginal effects
        sum loenmv if count <0 & treatment ==0
        local control =r(mean)
        sum loenmv if count <0 & treatment ==1
        local treat =r(mean)
        sum loenmv if count>=0 & treatment ==0
        local control_post =r(mean)

        forvalue t=-4/4{
                local n = `t'*-1
                if `t' < -1 gen T_`n' = treatment ==1 & count ==`t'
                if `t' > -1 gen T`t' = treatment ==1 & count ==`t'

        }

        **estimate DiD model on salary
        reghdfe syg_barsel_13 T*, abs(alder female count time
treatment edu) cl(pnr  id)
        eststo est1_`control_time'
        if `control_time'==1 matrix results = J(5,5,.) // matrix to
capture results
        if `control_time'==1 matrix results_p = J(5,5,.) // matrix
to capture results


        matrix b = e(b)
        matrix V = e(V)
        local n = `control_time'

        matrix results[`n',1]    = b[1,1] / 7466  // capture beta
results as 1K Euro
        matrix results[`n',2]    = (V[1,1]^.5)/7466       //
capture standard error as 1K Euro
        matrix results[`n',3]    =       b[1,1]/(`control_post'-
(`control'-`treat'))
        matrix results[`n',4]    =       `n'




        **Generate pre-concussion probability difference   for
        **use in calculating marginal effects

        **estimate DiD LP-model on P(salary=0)
        xi: reghdfe kont_dag T*, abs(alder female count time
treatment edu) cl(pnr  id)
        eststo est2_`control_time'
        matrix b =e(b)   //regression coefficient
        matrix V = e(V)  // standard error^2
```

```
        matrix results_p[`n',1]  =       b[1,1]
        matrix results_p[`n',2]  =       V[1,1]^.5
        *matrix results_p[`n',3] =       b[1,1]/(`control_post'-
(`control'-`treat'))
        matrix results_p[`n',4]  =       `n'

}

esttab est1_* using [home]/tables/dynamic_sickpay1.rtf, ///
        replace se(3) b(3) compress nogap star(+ .1 * .05 ** .01
*** .001) ///
        keep(T*)

esttab est2_* using [home]/tables/dynamic_welfare2.rtf, ///
        replace se(3) b(3) compress nogap star(+ .1 * .05 ** .01
*** .001) ///
        keep(T*)
```
