# Supplementary material for: Effect of concussion on salary and employment: a population-based event time study using a quasi-experimental design
Source: BMJ Open. 2020 Oct 21;10(10):e038161. doi: 10.1136/bmjopen-2020-038161 (PMC7580067; doi:10.1136/bmjopen-2020-038161)
Supplement: Supplementary data [file bmjopen-2020-038161supp003.pdf]

BMJ Publishing Group Limited (BMJ) disclaims all liability and responsibility arising from any reliance placed on this supplemental material which has been supplied by the author(s)

Supplemental results

Fallesen P, Campos B. *BMJ Open* 2020; 10:e038161. doi: 10.1136/bmjopen-2020-038161

Table S1. Test of parallel trends assumption pre-exposure incident against each control group separately using eq. S3 in supplementary methods. Separate exposure dummies for all time periods (except the year prior to exposure, which serves as reference period).

Time to exposure group's concussion (exposure)	Δ=1<br>Est<br>(S.E.)<br>p-value	Δ=2<br>Est<br>(S.E.)<br>p-value	Δ=3<br>Est<br>(S.E.)<br>p-value	Δ=4<br>Est<br>(S.E.)<br>p-value	Δ=5<br>Est<br>(S.E.)<br>p-value
**Exposure-4y**	-0.368<br>(0.226)<br>p=.104	0.046<br>(0.363)<br>p=.900	0.120<br>(0.362)<br>p=.741	0.159<br>(0.313)<br>p=.612	0.042<br>(0.329)<br>p=.899
**Exposure-3y**	-0.094<br>(0.317)<br>p=.768	0.227<br>(0.510)<br>p=.656	0.167<br>(0.354)<br>p=.637	0.537<br>(0.372)<br>p=.148	0.113<br>(0.393)<br>p=.774
**Exposure-2y**	-0.548<br>(0.312)<br>p=.079	-0.082<br>(0.347)<br>p=.812	-0.163<br>(0.236)<br>p=.491	-0.124<br>(0.247)<br>p=.617	0.082<br>(0.250)<br>p=.744
**Exposure-1y**	Ref.	Ref.	Ref.	Ref.	Ref.
**N*T**	284115	273725	266120	260647	256337

Note: The table shows test for differences in pre-exposure trends between exposure and control group model using interactions between pre-exposure time dummies and the exposure indicator. There is no indication of substantial or significant pre-exposure differences in salary trajectories between exposure group and any of the control groups.

Fallesen P, Campos B. *BMJ Open* 2020; 10:e038161. doi: 10.1136/bmjopen-2020-038161

Table S2. Effect of concussion on different labor market outcome parameters using separate exposure dummies for all time periods (except the year prior to exposure, which serves as reference period): In this exploratory analysis, the exposure group is compared to the control group Δ=5, which suffers a concussion five years after the exposure group. Outcomes include annual salaried income (annual salary), total annual income (total income), annual sick leave benefits received (sick leave benefits) as well as a binary indicator of employment (probability of employment). Monetary outcomes were measured at 2015-level in € 1,000.

**Time to exposure group's concussion (exposure)**	**Annual Salary Est. (S.E.) p-value**	**Total income Est. (S.E.) p-value**	**Sick leave benefits Est. (S.E.) p-value**	**Probability of employment Est. (S.E.) p-value**
**Exposure-4y**	0.012	0.164	0.035	0.001
	(0.212)	(0.173)	(0.036)	(0.004)
	p=.954	p=.343	p=.320	p=.803
**Exposure-3y**	0.059	0.305	0.022	-0.001
	(0.252)	(0.233)	(0.034)	(0.003)
	p=.814	p=.190	p=.529	p=.739
**Exposure-2y**	0.043	0.122	0.002	0.001
	(0.160)	(0.147)	(0.029)	(0.003)
	p=.788	p=.405	p=.946	p=.739
**Exposure-1y**				
**Exposure**	-0.611	-0.338	0.166	-0.003
	(0.168)	(0.140)	(0.030)	(0.003)
	p<.001	0.016	p<.001	p=.317
**Exposure+1y**	-1.389	-0.608	0.288	-0.020
	(0.209)	(0.162)	(0.039)	(0.003)
	p<.001	p<.001	p<.001	p<.001
**Exposure+2y**	-1.568	-0.847	0.132	-0.023
	(0.261)	(0.231)	(0.039)	(0.004)
	p<.001	p<.001	p=.001	p<.001
**Exposure+3y**	-1.393	-0.497	0.031	-0.022
	(0.246)	(0.219)	(0.040)	(0.004)
	p<.001	p=.023	p=.432	p<.001
**Exposure+4y**	-1.319	-0.499	-0.076	-0.018
	(0.253)	(0.218)	(0.042)	(0.004)
	p<.001	p=.022	p=.075	p<.001
**N*T**	577762	577758	577872	577872

Note: Annual salary include all income from salary and employee fringe benefits, employee stock options, employer paid sick leave, net gains (including interests and capital gains) from own companies. Total income includes all income absent wealth. Sick leave includes only public health benefits (sick leave and paternity leave). Employment is a binary indicator measured last week of November for each year. Results obtained from estimations following Eq. (1). Models include controls for high school diploma, gender, age, and observation year. Results obtained using reghdfe in Stata. Total number of observations (N*T) differ slightly between outcomes because all income information is not available for all observation all years.

Source: Own calculations on data from Statistics Denmark.

Fallesen P, Campos B. *BMJ Open* 2020; 10:e038161. doi: 10.1136/bmjopen-2020-038161

Table S3. Demographic factors for exposure group and control groups (Δ=1, 2, 3, 4, 5) averaged over the 5 years leading up to the concussion event in each of the groups. Factors include patient age (in years), share of sample female (1=100% female), and share of individuals with at least a high school degree (1=100%).

		Exposure	Control, Δ=1	Control, Δ=2	Control, Δ=3	Control, Δ=4	Control, Δ=5
**Pr(Female=1)**	Mean	.430	.438	.447	.458	.464	.473
	S.D.	(.495)	(.496)	(.497)	(.498)	(.499)	(.499)
	p-value		.030	<.001	<.001	<.001	<.001
**Age**	Mean	36.899	37.354	37.754	38.065	38.343	38.592
	S.D.	(11.856)	(11.857)	(11.718)	(11.630)	(11.584)	(11.491)
	p-value		<.001	<.001	<.001	<.001	<.001
**Pr(High school=1)**	Mean	.624	.632	.640	.646	.653	.660
	S.D.	(.484)	(.482)	(.480)	(.478)	(.476)	(.474)
	p-value		.026	<.001	<.001	<.001	<.001
**Total individuals**		37848	34551	31851	29922	28580	27484

Note: S.D.: Standard deviation. P-values calculated using two-sided t-tests. All test performed between exposure group and each control group separately.

Fallesen P, Campos B. *BMJ Open* 2020; 10:e038161. doi: 10.1136/bmjopen-2020-038161

Figure S1. Unnormalized Average Salary for Treatment and Control Groups Measured in 1K € at 2015-levels

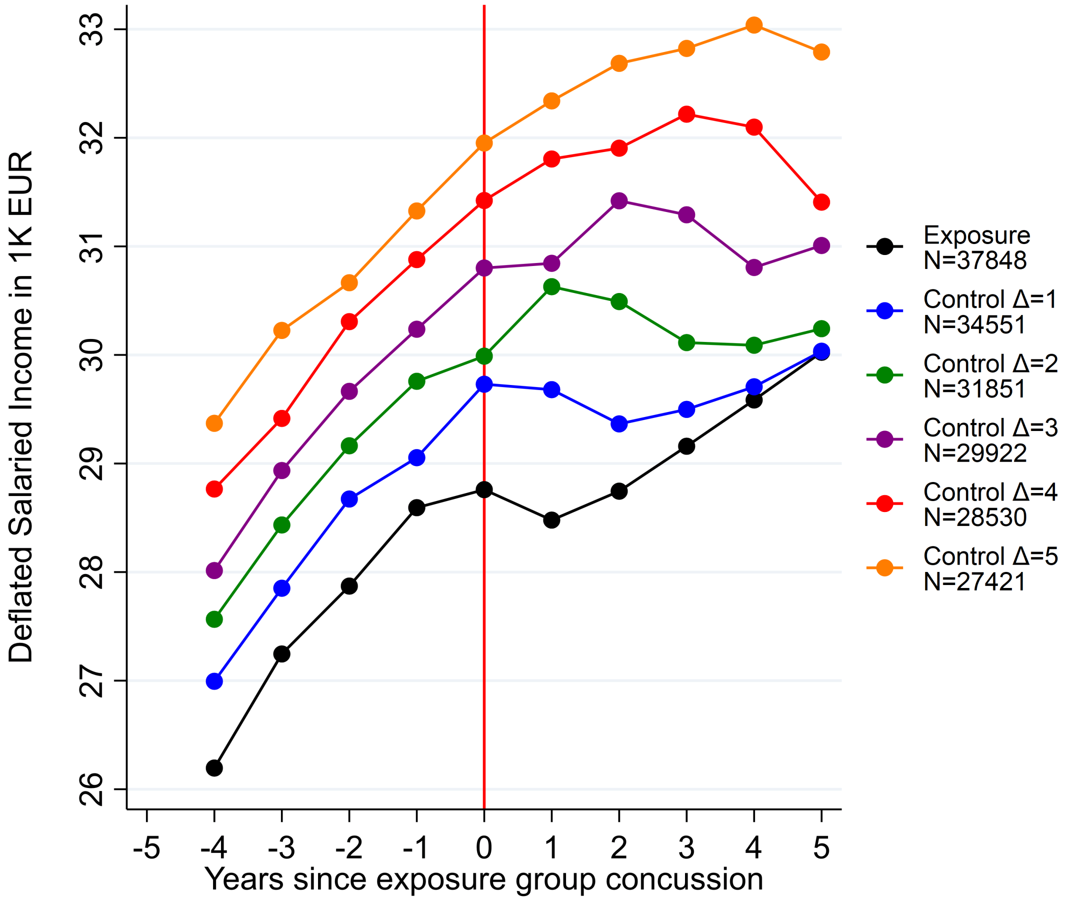

Note: Salary of the exposure group compared to salary of the 5 control groups, who experienced their concussions Δ={1, 2, 3, 4, and 5} years later than the exposure group. Salary progression is shown for the 5 years before and the 5 years after the exposure group suffered a concussion event. Table S1 demonstrates that the trends for salary progression pre-exposure incident are parallel between exposure group and each control group.

Fallesen P, Campos B. *BMJ Open* 2020; 10:e038161. doi: 10.1136/bmjopen-2020-038161

Figure S2. (Left Panel)The Cumulative Distribution for Total Income Post-Treatment among the Treatment Group and Their Counterfactual, and (Right Panel) the Difference between the Two CDFs Expressed as the Effect of Concussion on the Probability of Total Income Below that Income-Level Expressed on the X-Axis following Exposure Event.

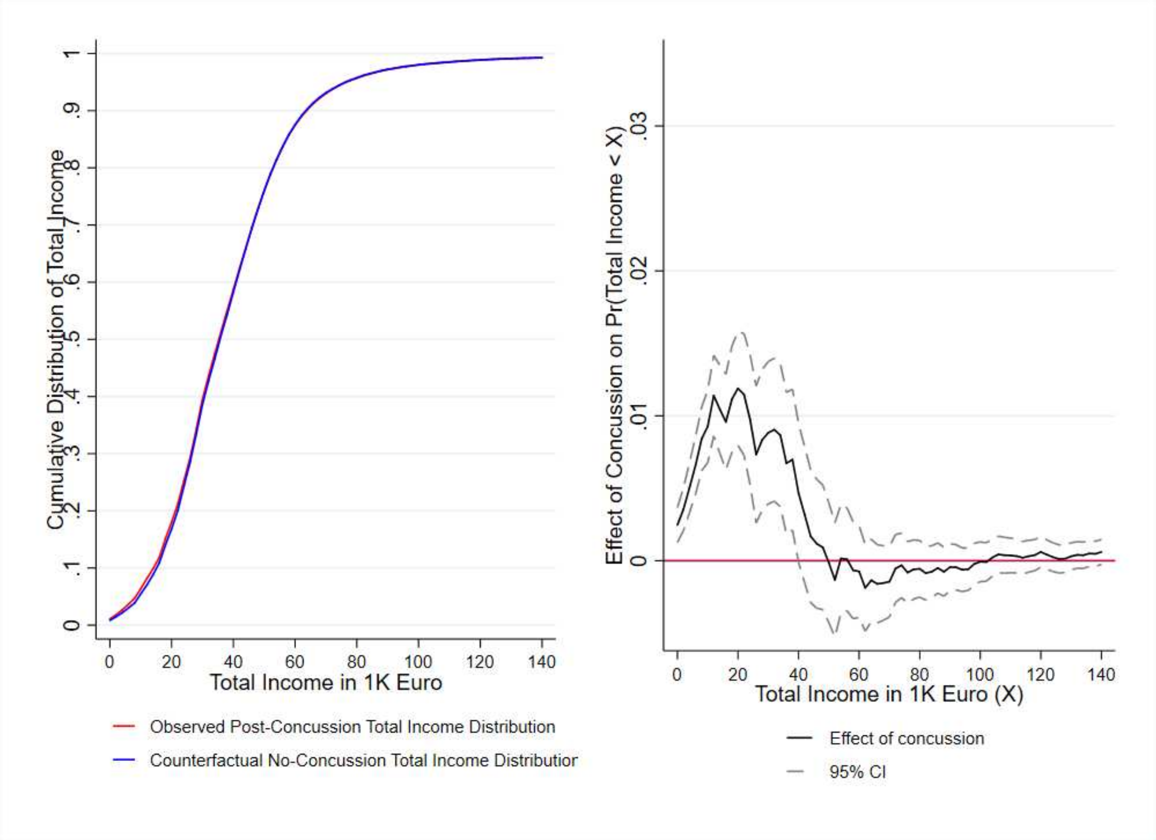

Note: The figure shows the observed cumulative salary distribution following a concussion (red) and the expected counterfactual salary distribution absent the concussion (blue). The black line shows the difference between the observed and the counterfactual distribution, and the grey dash lines show the 95 % confidence interval. The bell-shape of the difference between the two distributions as the total income increase from 0 to 40,000 € indicates that the main part of the effect of concussions on total incomes is driven by low-income people shifting total income downwards following concussion, but not going to total income equal to zero.

Figure S3. Percentage Effect of Concussion on Relative Salary Across Age Groups.

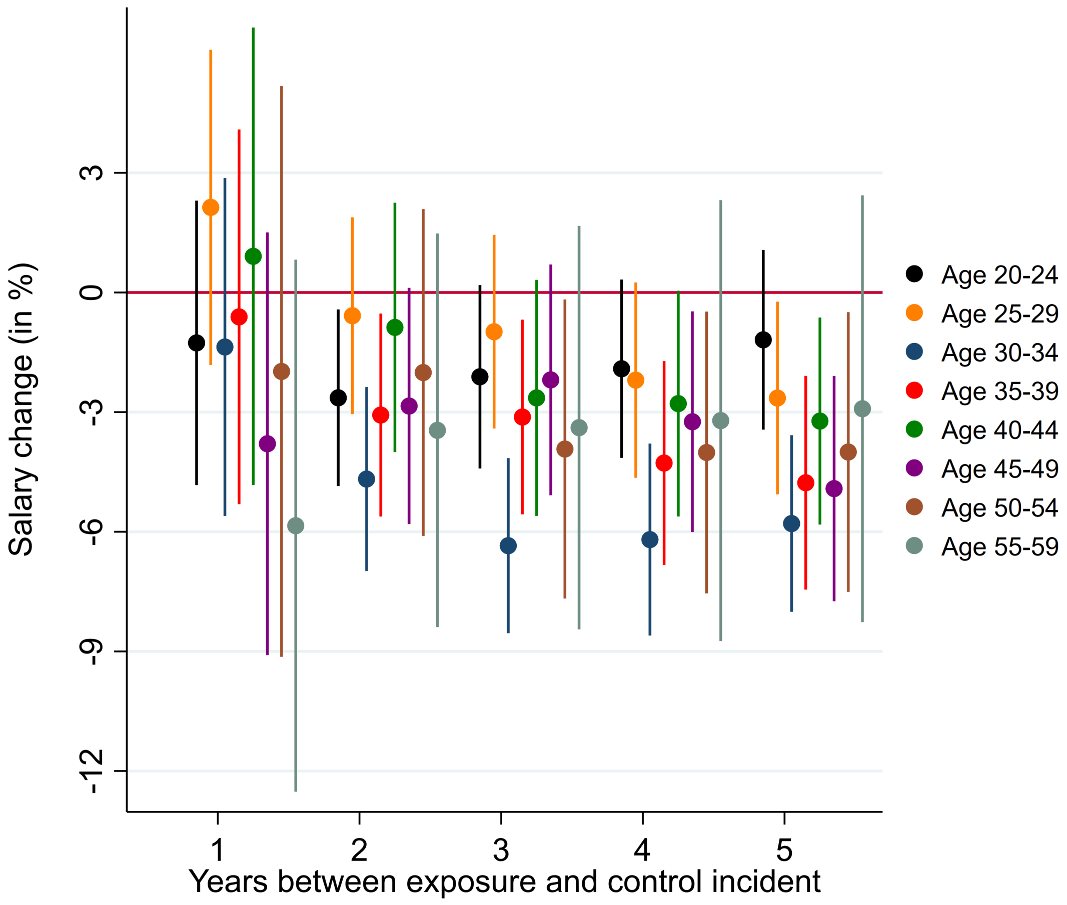

Note: Relative salary drop of the exposure group compared to the control groups, who suffer a concussion 1, 2, 3, 4, and 5 years after the exposure group. Relative salary changes are shown across different age groups. Graph shows parameter estimates and 95% CI.

Fallesen P, Campos B. *BMJ Open* 2020; 10:e038161. doi: 10.1136/bmjopen-2020-038161

Figure S4. Percentage Effect of Concussion on Relative Salary Across High School Completion.

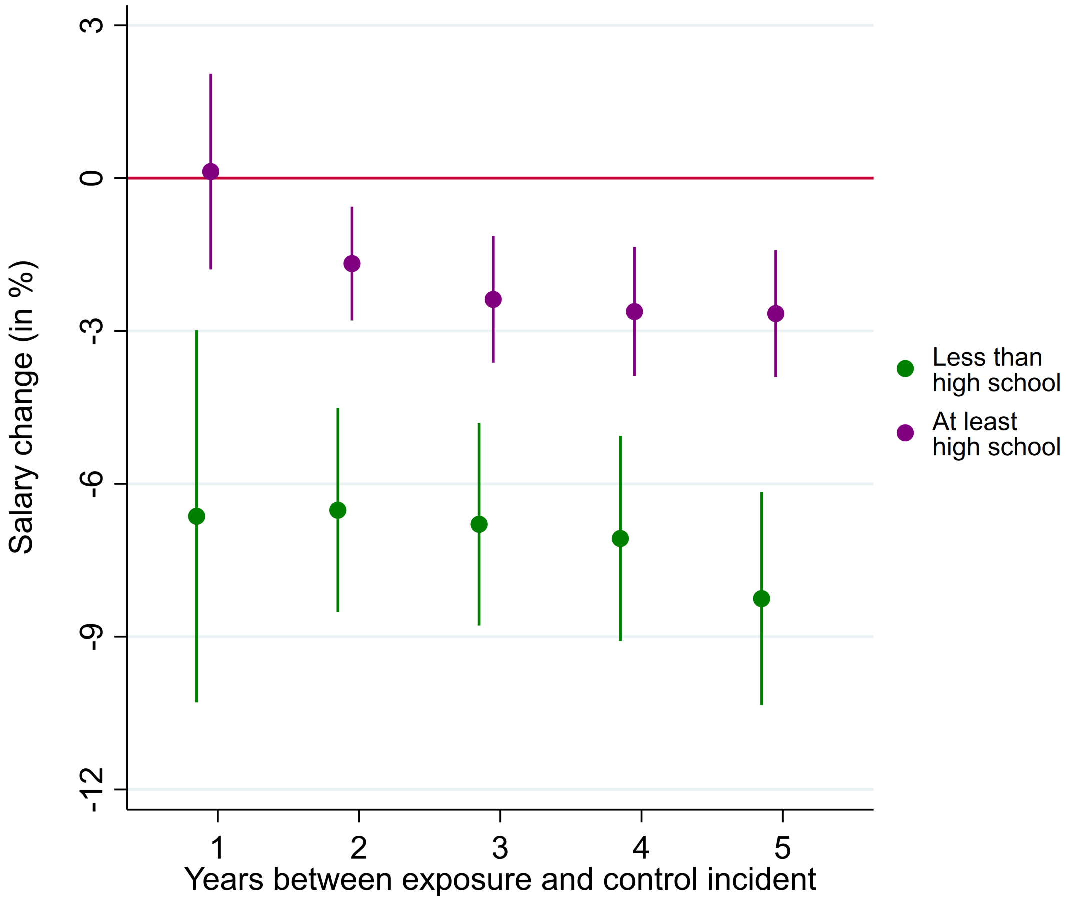

Note: Relative salary drop of the exposure group compared to the control groups, who suffer a concussion 1, 2, 3, 4, and 5 years after the exposure group. Relative salary changes are shown across whether individuals had obtained at least a high school diploma (ISCED > 2). Graph shows parameter estimates and 95% CI.

Fallesen P, Campos B. *BMJ Open* 2020; 10:e038161. doi: 10.1136/bmjopen-2020-038161

Figure S5. **Percentage Effect of Concussion on Relative Salary Across Gender.**

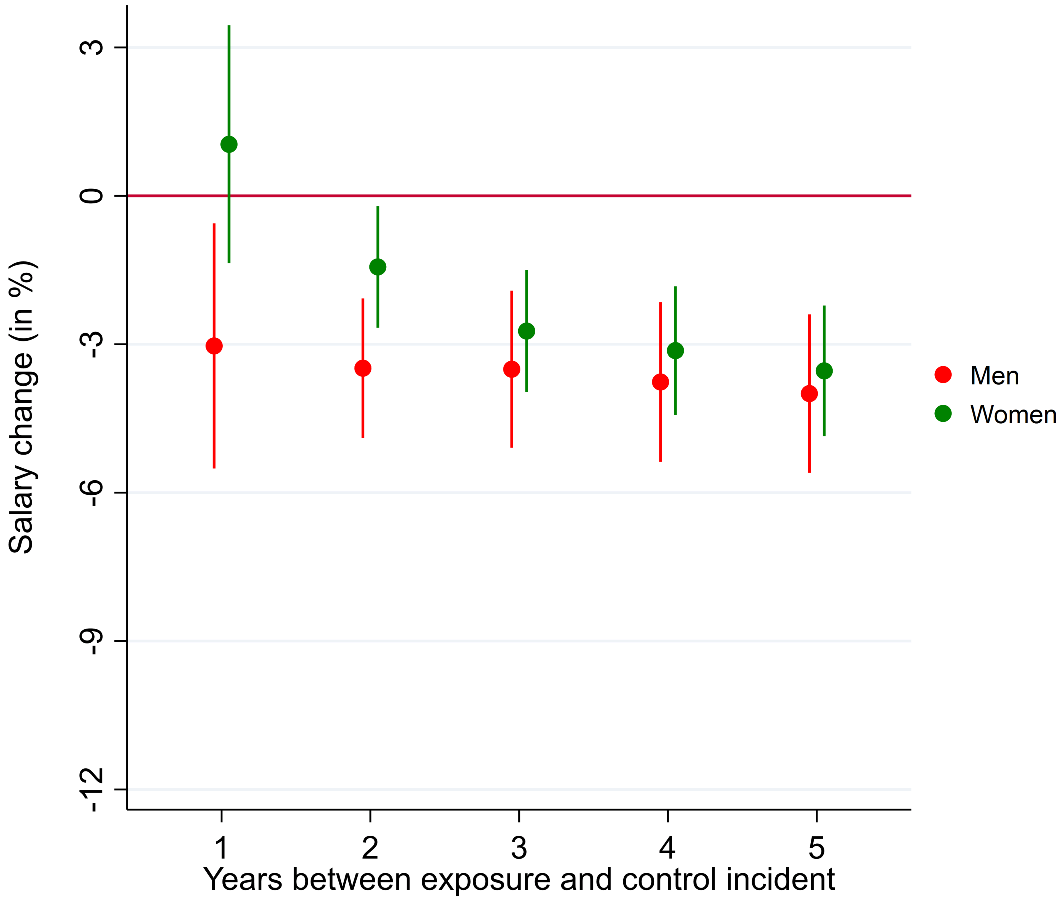

Note: Relative salary drop of the exposure group compared to the control groups, who suffer a concussion 1, 2, 3, 4, and 5 years after the exposure group. Relative salary changes are shown across gender. Graph shows parameter estimates and 95% CI.

Figure S6. Effect of Concussion on Absolute Salary in 1K Euro Across Age groups.

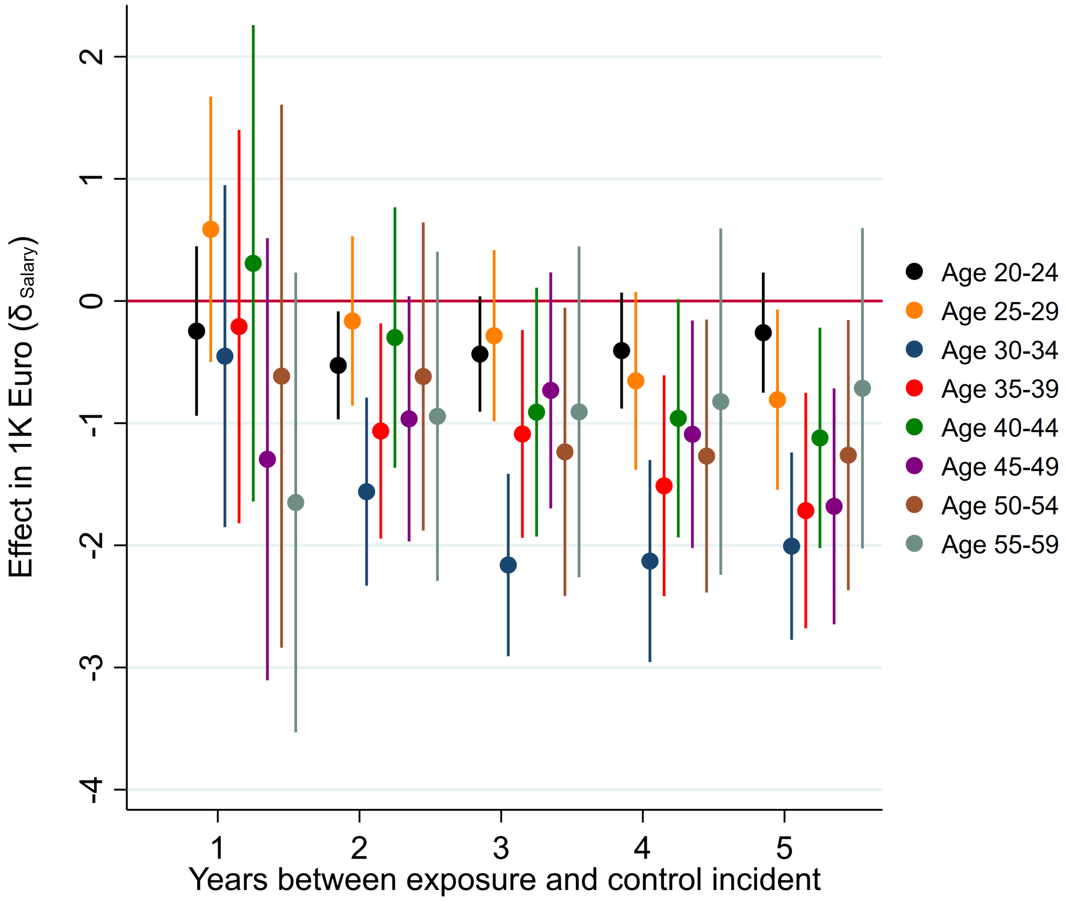

Note: Absolute salary drop of the exposure group compared to the control groups, who suffer a concussion 1, 2, 3, 4, and 5 years after the exposure group. Absolute salary changes are shown across different age groups. Graph shows parameter estimates and 95% CI.

Fallesen P, Campos B. *BMJ Open* 2020; 10:e038161. doi: 10.1136/bmjopen-2020-038161

Figure S7. **Effect of Concussion on Absolute Salary in 1K Euro Across Education.**

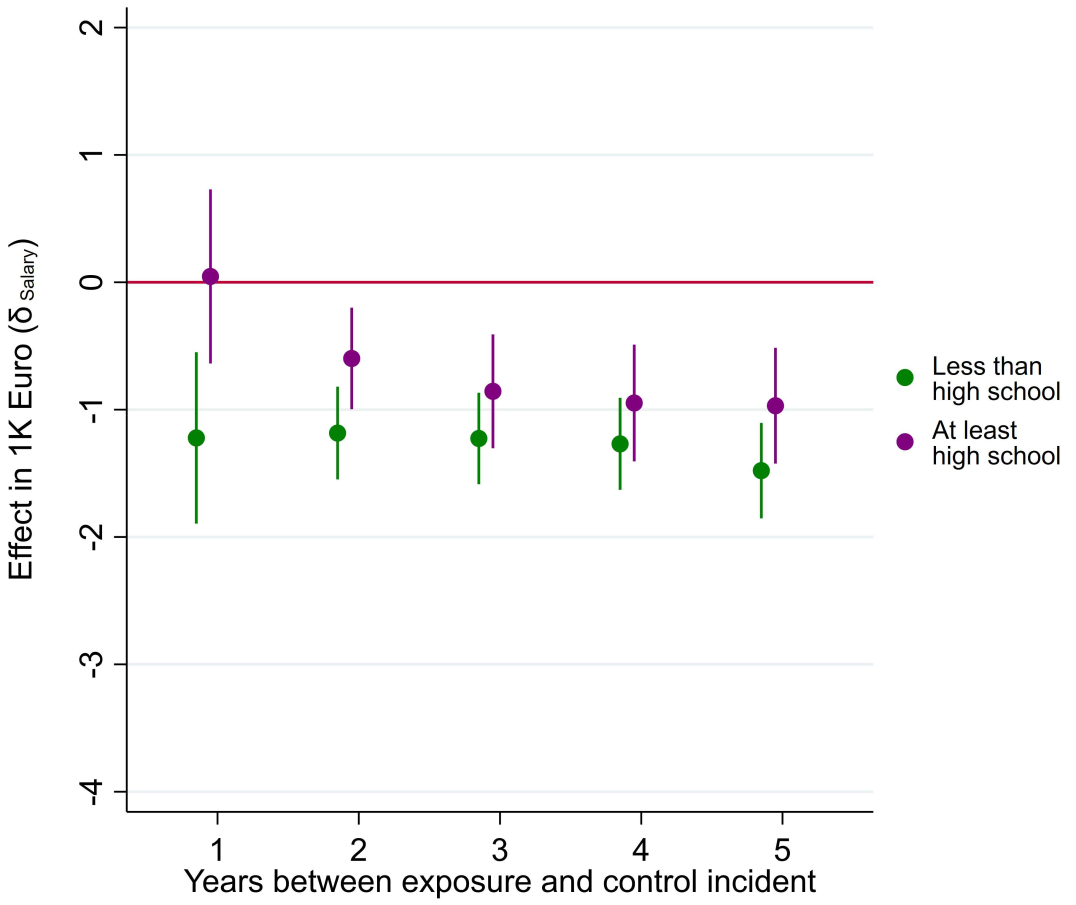

Note: Absolute salary drop of the exposure group compared to the control groups, who suffer a concussion 1, 2, 3, 4, and 5 years after the exposure group. Absolute changes in salary are shown across whether individuals had obtained at least a high school diploma (ISCED > 2). Graph shows parameter estimates and 95% CI.

Fallesen P, Campos B. *BMJ Open* 2020; 10:e038161. doi: 10.1136/bmjopen-2020-038161

Figure S8. **Effect of Concussion on Absolute Salary in 1K Euro Across Gender.**

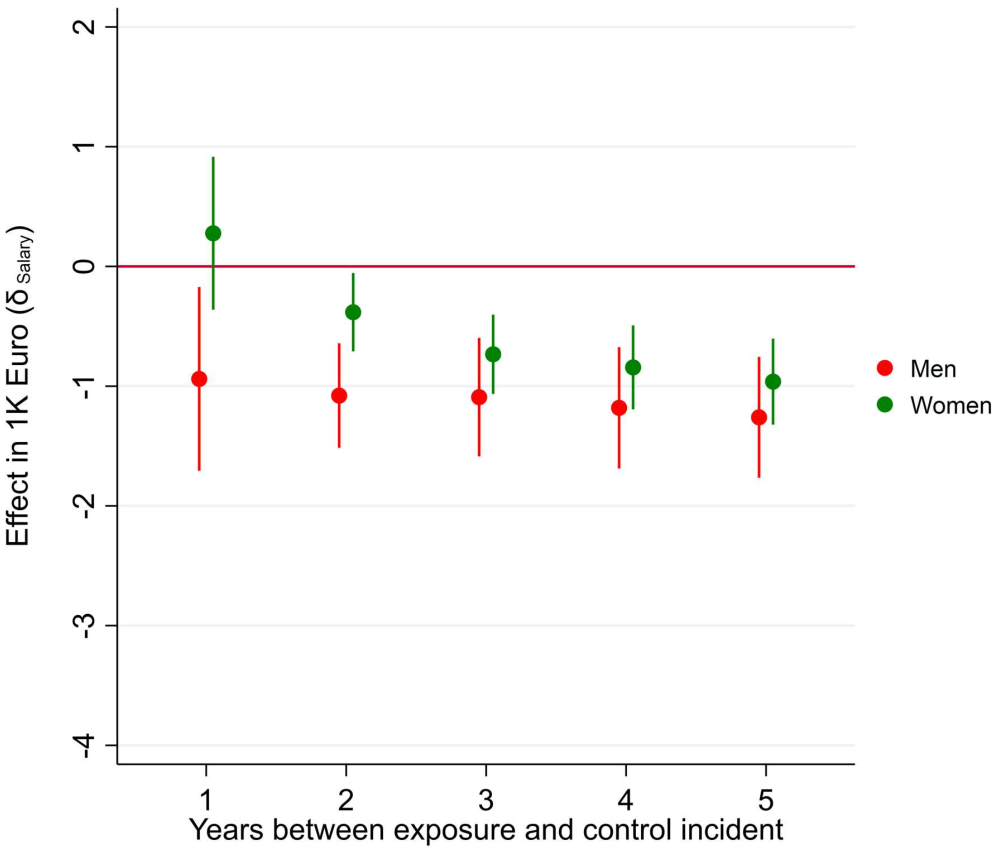

Note: Absolute salary drop of the exposure group compared to the control groups, who suffer a concussion 1, 2, 3, 4, and 5 years after the exposure group. Absolute changes in salary are shown across gender. Graph shows parameter estimates and 95% CI.
