# Supplementary material for: Effect of concussion on salary and employment: a population-based event time study using a quasi-experimental design
Source: BMJ Open. 2020 Oct 21;10(10):e038161. doi: 10.1136/bmjopen-2020-038161 (PMC7580067; doi:10.1136/bmjopen-2020-038161)
Supplement: Author's manuscript [file bmjopen-2020-038161.draft_revisions.pdf]

BMJ Open is committed to open peer review. As part of this commitment we make the peer review history of every article we publish publicly available.

When an article is published we post the peer reviewers' comments and the authors' responses online. We also post the versions of the paper that were used during peer review. These are the versions that the peer review comments apply to.

The versions of the paper that follow are the versions that were submitted during the peer review process. They are not the versions of record or the final published versions. They should not be cited or distributed as the published version of this manuscript.

BMJ Open is an open access journal and the full, final, typeset and author-corrected version of record of the manuscript is available on our site with no access controls, subscription charges or pay-per-view fees (http://bmjopen.bmj.com).

If you have any questions on BMJ Open's open peer review process please email info.bmjopen@bmj.com

The Effect of Concussion on Salary and Employment-A Population-Based Event Time Study using a Quasi-Experimental Design

Journal:	*BMJ Open*
Manuscript ID	bmjopen-2020-038161
Article Type:	Original research
Date Submitted by the Author:	02-Mar-2020
Complete List of Authors:	Fallesen, Peter; Stockholm University, Swedish Institute for Social Research<br>Campos, Benito; Heidelberg University Hospital Department of Neurosurgery, ;
Keywords:	Trauma management < ORTHOPAEDIC & TRAUMA SURGERY, HEALTH ECONOMICS, Neurosurgery < SURGERY

SCHOLARONE™
Manuscripts

I, the Submitting Author has the right to grant and does grant on behalf of all authors of the Work (as defined in the below author licence), an exclusive licence and/or a non-exclusive licence for contributions from authors who are: i) UK Crown employees; ii) where BMJ has agreed a CC-BY licence shall apply, and/or iii) in accordance with the terms applicable for US Federal Government officers or employees acting as part of their official duties; on a worldwide, perpetual, irrevocable, royalty-free basis to BMJ Publishing Group Ltd ("BMJ") its licensees and where the relevant Journal is co-owned by BMJ to the co-owners of the Journal, to publish the Work in this journal and any other BMJ products and to exploit all rights, as set out in our licence.

The Submitting Author accepts and understands that any supply made under these terms is made by BMJ to the Submitting Author unless you are acting as an employee on behalf of your employer or a postgraduate student of an affiliated institution which is paying any applicable article publishing charge ("APC") for Open Access articles. Where the Submitting Author wishes to make the Work available on an Open Access basis (and intends to pay the relevant APC), the terms of reuse of such Open Access shall be governed by a Creative Commons licence – details of these licences and which Creative Commons licence will apply to this Work are set out in our licence referred to above.

Other than as permitted in any relevant BMJ Author's Self Archiving Policies, I confirm this Work has not been accepted for publication elsewhere, is not being considered for publication elsewhere and does not duplicate material already published. I confirm all authors consent to publication of this Work and authorise the granting of this licence.

For peer review only

The Effect of Concussion on Salary and Employment-A Population-Based Event Time Study using a Quasi-Experimental Design

[1,2]Peter Fallesen, PhD, [3,4]Benito Campos, M.D.

[1]Swedish Institute for Social Research, Stockholm University, SE-106 91 Stockholm, Sweden
[2]ROCKWOOL Foundation, Ny Kongensgade 6, DK-1472 Copenhagen C, Denmark
[3]Department of Neurosurgery, University of Heidelberg, INF400, 69120 Heidelberg, Germany
[4]Department of Health Tech, Technical University of Denmark, DK-2800 Kongens Lyngby, Denmark

Address of Correspondence: Peter Fallesen, Swedish Institute for Social Research, Stockholm University, SE-106 91 Stockholm, Sweden, peter.fallesen@sofi.su.se, +4561333184

Keywords: administrative data; concussion; income; productivity

Abstract word count: 196

Text word count: 3025 (excluding references and figure/table legends)

Number of references: 20

Number of tables and/or figures: 2 tables and 4 figures

Number of videos: 0

For peer review only

Abstract

Objective: Concussions are the most frequent traumatic brain injuries. Yet, the socio-economic impact of concussions remains unclear. We study the socio-economic effect of concussions on working age adults on a population scale.

Design: Our population-based, event time study uses administrative data as well as hospital and emergency room records for the population of Denmark.

Setting: We study all Danish patients, aged 20-59 y, who were treated at a public hospital or emergency room between 2003-2017 after suffering a concussion without other intracranial or extracranial injuries (n=55,424 unique individuals) with no prior diagnosis of intra- or extracranial injury within the past ten years leading up to the incident.

Primary and Secondary Outcome Measures: As primary endpoint, we investigate the mean effect of concussion on annual salaried income within a five-year period after trauma. In an exploratory analysis, we study whether the potential impact of concussion on annual salaried income is driven by patient age, education or economic cycle.

Results: Concussion was associated with an average change in annual salary income of -1,223€ (95% CI, -1,540€; -905€, p<.001) corresponding to a salary change of -4.1 % (95% CI, -5.2 %; -3.1 %). People between 30-39 y and those without high school degrees suffered the largest salary decreases. Affected individuals leaving the workforce drove the main part of the decrease. Absolute annual effect sizes were countercyclical to the unemployment rate.

Conclusions: Concussions have a large and long-lasting impact on salary and employment of working-age adults on a nationwide scale.

For peer review only

Strengths and limitations of this study

- We use natural experiments to obtain plausible causal effects between concussion and salary/employment.
- We study a large, population-based sample with multiple data layers.
- We study how economic cycles affect our outcome measures.

For peer review only

For peer review only - http://bmjopen.bmj.com/site/about/guidelines.xhtml

Introduction

Concussions are by far the most frequently occurring intracranial injuries affecting approximately 1 in 219 Danes every year.[10] Immediate symptoms may last for days or weeks, and 10-15% of patients diagnosed with concussion suffer from long-term symptoms such as headache, fatigue, and intolerance to stress. Clinical practice has encouraged patients to restrict social, mental, and physical activity in the weeks following a concussion (see[14] for review), although prolonged inactivity may prolong symptoms.[4,8,12] Thus, symptoms, comorbidities, and suggested treatment are associated with short- to long-term absence from work and lower productivity.

Yet, the causal effect of concussion on economic burdens for individuals and society through decreased labor market activity has not been identified. First, concussion is a sudden incidence and thus not amenable to prospective study nor randomization. Cohort and case-control studies[1,3,7,16,17] provide some valuable evidence on employment and labor market outcomes among those who suffered concussions but are prone to selection bias. Individuals at high risk of concussions may differ on unobserved characteristics (e.g., risk aversion, routine activities) from those at low risk. People who are more likely to suffer concussions may also, on average, have more precarious or unstable employment trajectories prior to the incident, which may further bias prospective studies. Given the high incidence of concussion, even small losses of productivity and discrete drops in employment would have a significant socioeconomic impact and thus, it would require large patient cohorts with suitable controls to grasp the full socioeconomic impact of concussions. Thus, absent the possibility of randomization, using a natural or quasi-experimental design is the only likely option to parse out the *causal* effect of

For peer review only

concussions on labor market activity.[9]

We examine how concussions affect salary and employment of working age individuals in Denmark, a representative north-European industrial nation with a strong welfare state and a flexible labor market. We use administrative longitudinal data linked to hospital and emergency room diagnostic data on all Danes, who received a primary diagnosis of concussion between 2003 and 2017. To address the problem of unmeasured bias between those that do and do not experience a concussion, we use a quasi-experimental event-study approach[5,6] where we compare similar individuals, who experienced their concussions at different time points. Under mild assumptions of parallel trends in wage progression prior to concussion and random timing of concussion event within a five-year time frame, the approach recovers a robust estimation of the effect of concussion on annual salary and employment status.

For peer review only - http://bmjopen.bmj.com/site/about/guidelines.xhtml

Material & Methods

Data Sources and Sample Construction

Our concussion data originates from the Danish National Patient Registry (DNPR) (see[11] for description). DNPR is published annually and holds information on all hospitalizations at public hospitals in Denmark since 1977, and on all emergency room visits and outpatient treatments at public hospitals since 1994. Our combined exposure and control cohort includes all Danes aged 20-59 y, who received a primary diagnosis of concussion (ICD-10 code S06.0, ICD-8 code N850) between 2003 and 2017 and did not sustain any kind of additional intracranial or extracranial injury. Individuals who regularly engage in activities associated with a high risk of sustaining multiple concussions may differ from the average concussion patient and would likely be over-represented in the exposure sample. To avoid such potential bias, we exclude all individuals, who were diagnosed with any kind of brain trauma during a ten-year period prior to the concussion event. Altogether, we study a cohort of 55,424 individuals. Only attrition is through mortality and out-migration, and out-migrated or deceased patients with missing spells in the follow up period is excluded in those periods.

As a measure of productivity, we use price-index deflated annual salaried income. Salary information comes from Statistics Denmark's Income Statistics database. The database includes all declared annual incomes including income from self-employment. The Danish Tax Authorities supply the data to Statistics Denmark. Overall accuracy is considered very good.[15] Table 1 reports number of observations for the samples and number of observations with missing salary information. As evident, only between 0.01 to 0.02 percent of observations across exposure and control groups have missing salary information. We disregard these

For peer review only - http://bmjopen.bmj.com/site/about/guidelines.xhtml

observations in the main analysis. Through social security numbers we link information on salaried income to records on diagnosed concussions. Further, we obtain information on high school or equivalent level degree at time of concussion using the Danish Education Database. From the Danish Population Database, we obtain demographic information on age and gender for all respondents. Since the data used in the study come from de-identified administrative registers that Statistics Denmark makes available for research purposes for approved institutions, no approval from an ethics committee was needed to carry out the study. The research was carried out as part of project no. 706630 approved by Statistics Denmark.

Quasi-experimental design

We use a quasi-experimental, difference-in-differences event time approach previously described in a health setting by Dobkin et al.[5] We compare two groups of individuals from the same cohort, where both groups experience concussions, but at two different time points (t_c,t_c+Δ). Specifically, we sample all 55,496 individuals into six different subgroups: i) The *exposure group*, which includes all patients, who suffered their concussion during the period 2003-2012 (n=37,848) and ii) five *control groups*, which comprise patients who experienced their concussions Δ={1 (n=34,551), 2 (n=31,851), 3 (n=29,922), 4 (n=28,530), and 5 (n=27,421)} years later than the exposure group and did not experience any kind of brain injury in the 10+Δ years before the concussion event (note that the design allows individuals to both be part of the exposure and control group). Our model is built on the assumption that the exact timing of a concussion is random for small enough sizes of Δ, and on the additional assumption that the exposure and the control groups would have displayed parallel trends in salary if the

For peer review only - http://bmjopen.bmj.com/site/about/guidelines.xhtml

control group had not suffered a concussion at t_c. Table 1 show the number of patients in the exposure group and the five control groups for each year relative to exposure group's concussion incident. Using multiple comparison groups allow us the gage the validity of the assumption that the exact timing of a concussion is random for small enough sizes of Δ

To estimate the impact of concussion on labor market outcomes, we focus on the change in annual salary as our primary outcome, and, in further exploratory analyses, study additional outcomes such as income from health-related benefits, income from welfare benefits, and employment rates. Our data is nested within a three-level structure: Exposure or control group *g*, which includes individuals *I*, at times to exposure-groups concussion incident *t*. First, we estimate a standard difference in differences model for each separate control group Δ={1, 2, 3, 4, and 5} using ordinary least squares:

$$Salary_{git} = \beta_0 + \gamma Exposure_g + \theta Post_t + \delta Post \times Exposure_{git} + \boldsymbol{X_i \beta} + \sum_{Age=20}^{59+\Delta} I(Age)\eta_{Age} + \sum_{Year=1999}^{2012} I(Year)\eta_{Year} + \epsilon_{git} \quad (1)$$

where $Salary_{git}$ measures annual salaried income deflated to 2015-level; $Exposure_g$ indicates whether the observation belongs to the exposure or control group; $Post_t$ captures the period after the exposure group's concussion occurred; $Post_t \times Exposure_{git}$ captures the effect concussion, measured as share of year t ≥ 0 affected by concussion; $\boldsymbol{X_i}$ is a set of covariates that includes a high school indicator and a gender dummy; ϵ_{git} is the error-term; and the two last sets of indicator variables $I(Age)$ and $I(Year)$ capture age and incident year (for control group, the year indexed against). Under an assumption of parallel trends in salaried earnings between exposure and control groups had the exposure concussion not occurred, δ then captures the annual causal effect of concussion on salary for people exposed to concussions.

For peer review only

For additional exploratory analyses, we also estimate separate models across gender, educational level, and age, as well as across the salary distribution (see Supplemental Methods, Supplemental Digital Content 1, for further details).

Standard Protocol Approvals, Registrations, and Patient Consents

Since the data used in the study come from de-identified administrative registers that Statistics Denmark makes available for research purposes for approved institutions, no approval from an ethics committee was needed to carry out the study. The research was carried out as part of project no. 706630 approved by Statistics Denmark.

Patient and Public Involvement

There was no involvement from patients or members of the public in the design, or conduct, or reporting, or dissemination plans of the research.

For peer review only - http://bmjopen.bmj.com/site/about/guidelines.xhtml

Results

Concussion leads to long-term loss in salaried income

Compared to people who experienced their concussion one to five years after the exposure group, concussions had a sizeable effect on salaried income. Comparing to people who experienced a concussion one year after the exposure group, we estimate the loss in salaried income to be 423€ (95% CI: -9129€; 73€, p=.095; Table 2), corresponding to a salary decrease of 1.5 % (95% CI: -.3.0 %; 3.2 %; Figure 1). Comparing to the control group, who suffered their concussions five years after the exposure group, annual salary of the exposure group was 1,243€ (95% CI: 1,564€; 922€, p<.001) lower than annual salary of the control group Δ=5, corresponding to a salary decrease of 4.2 % (95% CI: 3.1 %; 5.3 %; Figure 1). Normalized wage progression for the control groups, who suffered a concussion 1, 2, 3, 4, and 5 years after the exposure group, showed similar trends and similar levels pre-exposure, indicating that the parallel wage trends assumption was met (Figure 2 and table S1, Figures S1 in Supplemental Digital Content 2). Thus, there is a sizeable effect of concussion on salary, and the effect only fully reveal itself after the first year since exposure incident.

We hypothesized that the salary decrease caused by concussion resulted from a combination of lower salary and exit from the labor market, either through short- or long-term absence/unemployment. In an exploratory analysis, we tested whether labor force exit drove the full effect of concussion on salary (Figure 3). Compared to the control group Δ=5, which suffers a concussion five years after the exposure group, a concussion was associated with 2.6% (95% CI: 3.0 %; 2.2 %, p <.001) increase in the risk of receiving € 0 in annual salary, or, in other words, effectively exiting employment. From the cumulative density function in Figure

For peer review only

3 we infer that exit from employment among the bottom half of the salary distribution drove the effect of concussion on salary.

Long-term loss in salaried income stems from exit from the labor market

To further examine whether exit from the labor market was caused either through short- or long-term absence/unemployment, we estimated a dynamic model using the control group Δ=5, which suffers a concussion five years after the exposure group. Following the concussion incidence, sick leave benefits payments were higher in the exposure group for the first two years following the incident. Concussed individuals were to some extent compensated for their salaried income loss through sick leave benefits in the first few years following a concussion, but the compensation ended while the salary drop persisted. Further, employment in the exposure group remained lower than in the control group Δ=5 and remained so for the entire post-exposure period (see table S2, Supplemental Digital Content 2 for further details). Total income decline was lower than the salary decline through the five years, which indicates that some form of public benefits covered part of the salary loss (see Figure S2, Supplemental Digital Content 2 for further details).

These results suggest that the impact of concussions on salary largely stems from affected individuals leaving the labor force completely, likely instead sustaining themselves through early retirement, disability pensions, self-sufficiency, or other income sources.

Younger patients without high school degree drove the effect of concussion on loss income

The exposure group and all control groups differed slightly in terms of average patient age,

For peer review only - http://bmjopen.bmj.com/site/about/guidelines.xhtml

For peer review only

male to female ratio, and for control group Δ=5, in the frequency of individuals with at least a high school degree (see Table S3, Supplemental Digital Content 2 for further details). To ensure that differences in gender, education, or age did not influence our results, we subdivided our exposure group into subgroups based on gender, education status, and age at time of concussion. We then estimated the impact of concussion on salary and employment across all values of Δ and for all subgroups (see, Figures S3-S8, Supplemental Digital Content 2 for further details). Patients between age 30-39 and those without a high school degree experienced the largest absolute and relative declines in salary.

Finally, we addressed the role of timing of concussion across different years. Given that per design our exposure group always suffered their concussion earlier than the control groups do, changing labor market conditions could moderate effects. Part of our sample suffered their concussion during or just prior to the Great Recession in 2009-2010, which arguably presented the largest shock to both the global and local economy since the Great Depression in the 1930s. In Denmark, the great recession was preceded by a series of years of economic growth, low unemployment, and increasing salaries (see Figure S8, Supplemental Digital Content 2 for salary development from 1994 to 2017). We estimated the impact of concussion on salary separately for each year from 2003-2012 and plotted the estimate against the percent of full-time unemployment in the Danish labor force (Figure 4). Suffering a concussion during an economic boom had a substantially higher impact on salary than doing so during a recession when comparing to control groups who suffered concussions two to five years later than exposure group.

For peer review only - http://bmjopen.bmj.com/site/about/guidelines.xhtml

Discussion

The impact of concussion on employment and salary remains understudied. In a systematic review of four studies on the association between mild TBI and return to work, Cancelliere and colleagues[1] found that most workers return to work within 3-6 months of suffering a mild TBI, but that the long-term impact (more than one year after concussion) was not studied. In addition, studies included small to medium sample sizes, varied measures of return to work, and employed both case-control and cohort designs. Using an inception cohort study design, Theadom and colleagues[16] collected follow up data four years after mild TBI incidents on 245 New Zealanders who were employed prior to incident. They found a 3.6 percent productivity decline among those who suffered a concussion, compared to a 2.3 population average decline. The group suffering mild TBI also reported more difficulties carrying out work-related tasks. In a related study,[16] Theadom and colleagues further found that the mild TBI group had persisting cognitive symptoms four years after suffering their concussion compared to an age-sex matched control group. Also using a case-control design and data from Taiwan, Chu and colleagues[3] found that one month after incident, 26 percent of patients had still not managed to return to work, and a large share of those who did return scored below full-time employment on a work quality index. Only one other study by Graff and colleagues[7] include a large patient cohort (n=19,732). Using case-control they found an association between concussion and failing to return to work of 1.54 odds ratio, but also found that exposed individuals had lower labor market attachment and was more likely to receive health related benefits pre-incident compared to the control group.

In the present study, we overcame some of the obstacles faced by previous work on the impact

For peer review only - http://bmjopen.bmj.com/site/about/guidelines.xhtml

254 of concussions on labor market outcomes by including a vast cohort of patients and exploiting a quasi-experimental design that allow us to plausibly account for unobserved difference between exposure and control group. In addition, salary and employment data reported here were compiled routinely through third-party reporting and were mandatory for all subjects, thus giving a complete and comprehensive picture of the economic impact of concussion on a nationwide scale.

260 Altogether, we showed that Danes between 20-59 year of age, who suffered a concussion during the period 2003-2012 experienced average salary losses of 4.1%. The impact of concussions on salary already materialized one year after the incident and remained sizeable for at least five years. This result is in line with an implementation period in which the impact of concussion on wages fully develops. First, concussions occur at some point during the year, thereby not affecting already earned salary that year. Second, in Denmark, most employees are entitled to receive their salary during sickness absence for an open ended, but not indefinite period.

268 If we assume that people return to their expected salary levels after a five-year recovery period (a very conservative assumption that is not supported by our data), the mere net annual salary loss in our sample would be approximately €23,000,000 measured in 2015-value. That would neither include hospital charges, medical costs for the treatment of concussion, the foregone tax from income, and the increased need for welfare spending, nor would it account for the large group of individuals who never seeks treatment[13] or receive their diagnosis from their general practitioner rather than in a hospital or emergency room, and thus escape our study. Thus, total public costs are likely substantially higher.

For peer review only - http://bmjopen.bmj.com/site/about/guidelines.xhtml

In addition, both in absolute and relative terms, the early peak-working aged individuals (30-39 y) and the less-educated individuals in our cohort seemed to be most affected after suffering a concussion. These findings might have and additional and yet unmeasured social impact, especially if our results are transferrable to other nations with a less established welfare state and a less flexible labor market. In such countries, the impact on the young and less-educated individuals suffering a concussion and thus on society might be accentuated.

Comparing our hospital incidence rates to more complete canvases of incidences[2], it seems likely that the actual cost in the population is more than twice as large as what we estimate. If we were to consider the average concussion incidence rates for six other advanced European countries that are somewhat comparable to Denmark (Norway, Finland, Germany, Netherlands, England and France) and under the assumption that concussion have a similar impact on earnings in these countries, the net annual salary loss would be approximately €1,099,400,000 measured in 2015-value. While our study likely underestimates the total socioeconomic impact of concussion, it suggests that concussions has a large economic impact on a nationwide scale and on productivity and income at the patient level.

Conclusion

Using timing of concussion as a natural experiment, we provide first plausible causal estimates of the effect of concussion on salary and employment. Our results show that concussion has a large and long-term negative causal impact on salary and employment. People between 30-39 y and those without high school degrees suffered the largest salary decreases.

For peer review only

For peer review only - http://bmjopen.bmj.com/site/about/guidelines.xhtml

Disclosures:

The authors report no conflict of interest.

Acknowledgements:

NA

Funding:

This work was supported by the Swedish Research Council for Health (PF), Working Life and Welfare (Grant no. 2016-07099) with additional funding from the ROCKWOOL Foundation (PF). The research was carried out independently of the funders.

Authors contributions: P.F. and B.C. conceived of the presented idea, P.F. performed the computations. P.F. and B.C. verified the statistical methods. P.F. and B.C. discussed the results and wrote the manuscript. The corresponding author confirms that he had full access to all the data in the study and had final responsibility for the decision to submit for publication.

Data Availability Statement

The data used in this study has been made available through a trusted third party, Statistics Denmark. Due to privacy concerns the data cannot be made available outside the hosted research servers at Statistics Denmark. University-based and private Danish scientific organizations can be authorized to work with data within Statistics Denmark. Such organization can provide access to individual scientists inside and outside of Denmark. Requests for data

For peer review only

For peer review only - http://bmjopen.bmj.com/site/about/guidelines.xhtml

may be sent to Statistics Denmark: http://www.dst.dk/en/OmDS/organisation/TelefonbogOrg.aspx?kontor=13&tlfbogsort=sektion or the Danish Data Protection Agency: https://www.datatilsynet.dk/english/the-danish-data-protection-agency/contact/. The authors document and make available all code needed to reproduce the findings in the study.

For peer review only

For peer review only - http://bmjopen.bmj.com/site/about/guidelines.xhtml

References

1. Cancelliere C, Kristman VL, Cassidy JD, Hincapié CA, Côté P, Boyle E, et al: Systematic Review of Return to Work After Mild Traumatic Brain Injury: Results of the International Collaboration on Mild Traumatic Brain Injury Prognosis. **Archives of Physical Medicine and Rehabilitation 95**:S201–S209, 2014
2. Cassidy JD, Carroll LJ, Peloso PM, Borg J, Von Holst H, Holm L, et al: Incidence, risk factors and prevention of mild traumatic brain injury: Results of the WHO Collaborating Centre Task Force on mild traumatic brain injury. **Journal of Rehabilitation Medicine 36**:28–60, 2004
3. Chu SY, Tsai YH, Xiao SH, Huang SJ, Yang CC: Quality of return to work in patients with mild traumatic brain injury: a prospective investigation of associations among post-concussion symptoms, neuropsychological functions, working status and stability. **Brain Injury 31**:1674–1682, 2017
4. DiFazio M, Silverberg ND, Kirkwood MW, Bernier R, Iverson GL: Prolonged Activity Restriction After Concussion: Are We Worsening Outcomes? **Clinical pediatrics 55**:443–51, 2016
5. Dobkin C, Finkelstein A, Kluender R, Notowidigdo MJ: The Economic Consequences of Hospital Admissions. **American Economic Review 108**:308–352, 2018
6. Fadlon I, Nielsen TH: **Household Responses to Severe Health Shocks and the Design of Social Insurance**. Cambridge, MA, 2015
7. Graff HJ, Siersma V, Møller A, Kragstrup J, Andersen LL, Egerod I, et al: Labour market attachment after mild traumatic brain injury: nationwide cohort study with 5-year register follow-up in Denmark. **BMJ open 9**:e026104, 2019
8. Lal A, Kolakowsky-Hayner SA, Ghajar J, Balamane M: The Effect of Physical Exercise After a Concussion: A Systematic Review and Meta-analysis. **The American journal of sports medicine 46**:743–752, 2018
9. Ogilvie D, Adams J, Bauman A, Gregg EW, Panter J, Siegel KR, et al: Using natural experimental studies to guide public health action: turning the evidence-based medicine paradigm on its head. **J Epidemiol Community Health**:2019
10. Pinner M, Jensen R, Birket-Smith M, Gade A, Riis JO, others: **Konsensusrapport Om Commotio Cerebri (Hjernerystelse)**. Videnscenter for Hjerneskade, 2003
11. Schmidt M, Schmidt SAJ, Sandegaard JL, Ehrenstein V, Pedersen L, Sørensen HT: The Danish National Patient Registry: a review of content, data quality, and research potential. **Clinical epidemiology 7**:449–90, 2015
12. Schneider KJ, Leddy JJ, Guskiewicz KM, Seifert T, McCrea M, Silverberg ND, et al: Rest and treatment/rehabilitation following sport-related concussion: a systematic review. **British journal of sports medicine 51**:930–934, 2017
13. Setnik L, Bazarian JJ: The characteristics of patients who do not seek medical treatment for traumatic brain injury. **Brain Injury 21**:1–9, 2007
14. Silverberg ND, Iverson GL: Is rest after concussion "the best medicine?": recommendations for activity resumption following concussion in athletes, civilians, and military service members. **The Journal of head trauma rehabilitation 28**:250–9, 2013
15. Statistics Denmark: Documentation of statistics: Income Statistics - Statistics Denmark.

For peer review only

For peer review only - http://bmjopen.bmj.com/site/about/guidelines.xhtml

2015 Available: https://www.dst.dk/en/Statistik/dokumentation/documentationofstatistics/income-statistics. Accessed 20 August 2018

16. Theadom A, Barker-Collo S, Jones K, Kahan M, Te Ao B, McPherson K, et al: Work Limitations 4 Years After Mild Traumatic Brain Injury: A Cohort Study. **Archives of Physical Medicine and Rehabilitation 98**:1560–1566, 2017
17. Theadom A, Starkey N, Barker-Collo S, Jones K, Ameratunga S, Feigin V, et al: Population-based cohort study of the impacts of mild traumatic brain injury in adults four years post-injury. **PLOS ONE 13**:e0191655, 2018
18. Faul M, Coronado V. Epidemiology of traumatic brain injury. **Handbook of clinical neurology 127**:3-13, 2015
19. Taylor CA, Bell JM, Breiding MJ, Xu L. Traumatic Brain Injury–Related Emergency Department Visits, Hospitalizations, and Deaths — United States, 2007 and 2013. **MMWR Surveillance Summaries 66**:1-16, 2017
20. Chernozhukov V, Fernández-Val I, Melly B. Inference on Counterfactual Distributions. **Econometrica 81**:2205-2268, 2013

For peer review only

Figure Legends

Figure 1. Estimated effect of concussions in percentage on salary for the exposure group measured against each control group

Figure shows the percentage change in salary experienced by the exposure group following their concussions compared to the expected trajectory absent the concussion (calculated from the control groups) with 95 % confidence intervals. See table 1 for separate p-values for each estimate.

Figure 2. Salary development for exposure and control groups across time of exposure

Figure shows the salary trajectories for the exposure group (black) who suffers concussion at year zero against normalized wage trajectories for the control groups who suffer their concussions one to five years later. Δ indicates the number of years between exposure and control incident. Table shows that there are no significant differences in the normalized salary levels for exposure and control group prior to exposure incident (see Figure S1, Supplemental Digital Content 2 for unnormalized salary trajectories).

Figure 3. The cumulative density function (cdf) for salary post-treatment among the treatment group and their counterfactual, and the difference between the two cdfs expressed as the effect of concussion on the probability of earning below that salary-level following exposure event.

The figure shows the observed cumulative salary distribution following a concussion (red) and

For peer review only - http://bmjopen.bmj.com/site/about/guidelines.xhtml

407 the expected counterfactual salary distribution absent the concussion (blue). The black line
408 shows the difference between the observed and the counterfactual distribution, and the grey
409 dash lines show the 95 % confidence interval. The closed to constant decline of the difference
410 between the two distributions as the salary increase indicates that the main part of the effect of
411 concussions on salary are driven by people having a salary equal to zero.

412 **Figure 4. Effect of concussion on salary across incident years and control groups**
413 **together with the percentage fulltime unemployed of the labor force.**

414 Figure shows annual estimates of concussion against each control group separately mapped
415 against the share of the labor force that is full time unemployed. 95 % confidence intervals. The
416 estimates for the effect of concussion on salary almost uniformly increase in absolute
417 magnitude when unemployment decreases, and decrease when unemployment increase,
418 indicating that the effect of concussion on salary is countercyclical to the economic cycle.

419

420 **Supplemental Digital Content titles & legends**

421 **Supplemental Digital Content [#1].** Text file. Supplemental materials and methods. This file
422 contains further details on our quasi-experimental, difference-in-differences event time
423 approach.

424

425 **Supplemental Digital Content [#2].** Table. Supplemental results Table S1: Test of parallel
426 trends assumption pre-exposure incident against each control group separately using eq. S3 in
427 supplementary methods. Separate exposure dummies for all time periods (except the year prior

For peer review only - http://bmjopen.bmj.com/site/about/guidelines.xhtml

For peer review only

to exposure, which serves as reference period).

Supplemental Digital Content [#2]. Table. Supplemental results Table S2: Effect of concussion on different labor market outcome parameters using separate exposure dummies for all time periods (except the year prior to exposure, which serves as reference period): In this exploratory analysis, the exposure group is compared to the control group Δ=5, which suffers a concussion five years after the exposure group. Outcomes include annual salaried income (annual salary), total annual income (total income), annual sick leave benefits received (sick leave benefits) as well as a binary indicator of employment (probability of employment). Monetary outcomes were measured at 2015-level in € 1,000.

Supplemental Digital Content [#2]. Table. Supplemental results Table S3: Demographic factors for exposure group and control groups (Δ=1, 2, 3, 4, 5) averaged over the 5 years leading up to the concussion event in each of the groups. Factors include patient age (in years), share of sample female (1=100% female), and share of individuals with at least a high school degree (1=100%).

Supplemental Digital Content [#2]. Figure. Supplemental results Figure S1: Unnormalized Average Salary for Treatment and Control Groups Measured in 1K € at 2015-levels

Supplemental Digital Content [#2]. Figure. Supplemental results Figure S2: The Cumulative Density Function (CDF) for Total Income Post-Treatment among the Treatment Group and

For peer review only - http://bmjopen.bmj.com/site/about/guidelines.xhtml

450 Their Counterfactual, and the Difference between the Two CDFs Expressed as the Effect of Concussion on the Probability of Total Income Below that Income-Level following Exposure Event.

454 **Supplemental Digital Content [#2].** Figure. Supplemental results Figure S3: Percentage Effect of Concussion on Relative Salary Across Age Groups.

457 **Supplemental Digital Content [#2].** Figure. Supplemental results Figure S4: Percentage Effect of Concussion on Relative Salary Across High School Completion.

460 **Supplemental Digital Content [#2].** Figure. Supplemental results Figure S5: Percentage Effect of Concussion on Relative Salary Across Gender.

463 **Supplemental Digital Content [#2].** Figure. Supplemental results Figure S6: Effect of Concussion on Absolute Salary in 1K Euro Across Age groups.

466 **Supplemental Digital Content [#2].** Figure. Supplemental results Figure S7: Effect of Concussion on Absolute Salary in 1K Euro Across Education.

469 **Supplemental Digital Content [#2].** Figure. Supplemental results Figure S8: Effect of Concussion on Absolute Salary in 1K Euro Across Gender.

For peer review only

Table 1. Number of observations for exposure and control groups across time since exposure and number of observations with missing salary information

Years until exposure	Exposure group	Control Δ=1	Control Δ=2	Control Δ=3	Control Δ=4	Control Δ=5
-4	36,804	33,681	31,112	29,190	27,859	26,794
-3	36,978	33,834	31,245	29,366	27,973	26,907
-2	37,195	34,003	31,407	29,501	28,146	27,031
-1	37,449	34,224	31,582	29,687	28,288	27,220
0	37,848	34,551	31,851	29,922	28,530	27,421
1	37,467		31,755	29,832	28,433	27,337
2	36,940			29,807	28,421	27,295
3	36,484				28,421	27,304
4	36,084					27,314
Total observations	333,249	170,293	188,952	207,305	226,071	244,623
Observations with missing salary	81	32	31	44	35	29

Control groups have not suffered a concussion in 10+Δ years before incident, exposure group has not suffered concussion the 10 years before exposure incident.

For peer review only

Table 2. Estimated effect of concussion on salary of exposure group compared to control groups that suffered their concussion Δ = 1, 2, 3, 4, 5 y after the exposure group's concussion event, measured at 2015-level. $N_{Exposure}$: 37,848

	Estimated salary effect (δ)	95 % CI	*p*	$N_{Control}$
Δ = 1 y	-423€	(-919€;73€)	.095	34,551
Δ = 2 y	-825€	(-1,108€; -543€)	<.001	31,851
Δ = 3 y	-1,019€	(-1,331€; -707€)	<.001	29,922
Δ = 4 y	-1,126€	(-1,446€; -805€)	<.001	28,530
Δ = 5 y	-1,243€	(-1,564€; -922€)	<.001	27,421

Results obtained from estimations following Eq. (1). Models include controls for high school diploma, gender, age, and observation year. Results obtained using reghdfe in Stata.

For peer review only

For peer review only - http://bmjopen.bmj.com/site/about/guidelines.xhtml

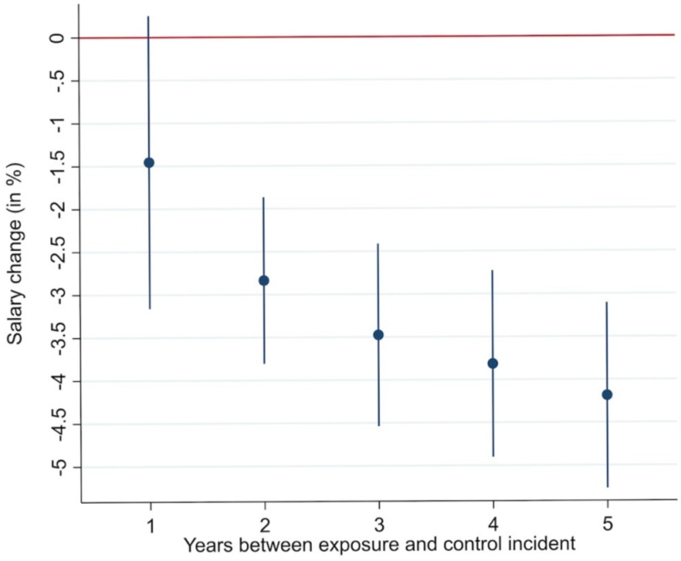

Figure 1

338x190mm (108 x 108 DPI)

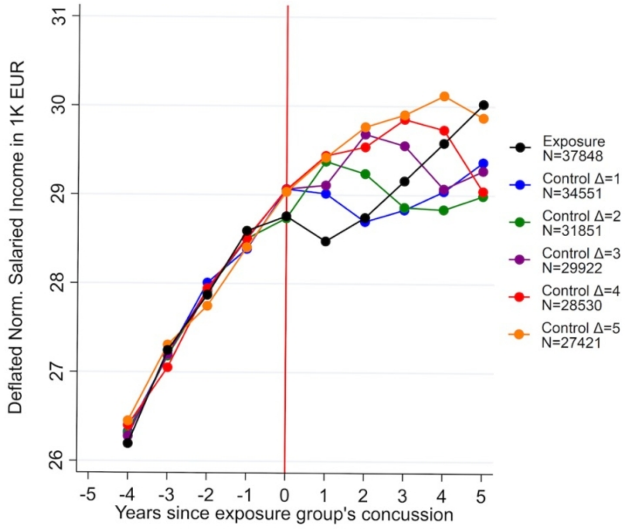

Figure 2

338x190mm (108 x 108 DPI)

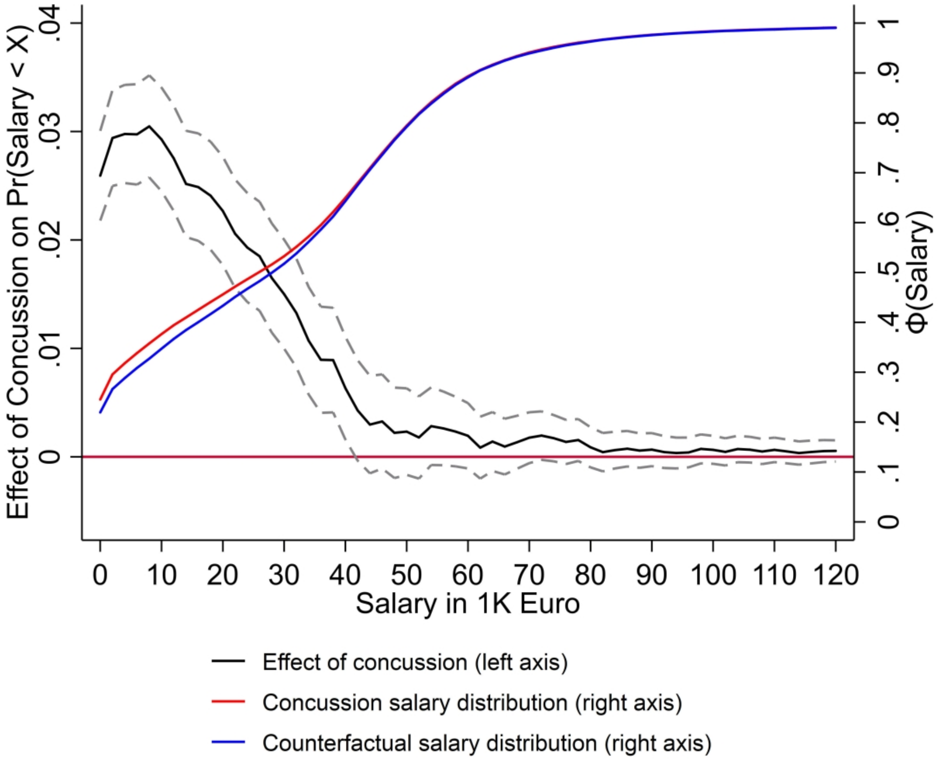
Effect of Concussion on Pr(Salary < X)
.04
.03
.02
.01
0
Φ(Salary)
1
.9
.8
.7
.6
.5
.4
.3
.2
.1
0
0 10 20 30 40 50 60 70 80 90 100 110 120
Salary in 1K Euro
Effect of concussion (left axis)
Concussion salary distribution (right axis)
Counterfactual salary distribution (right axis)

Figure 3

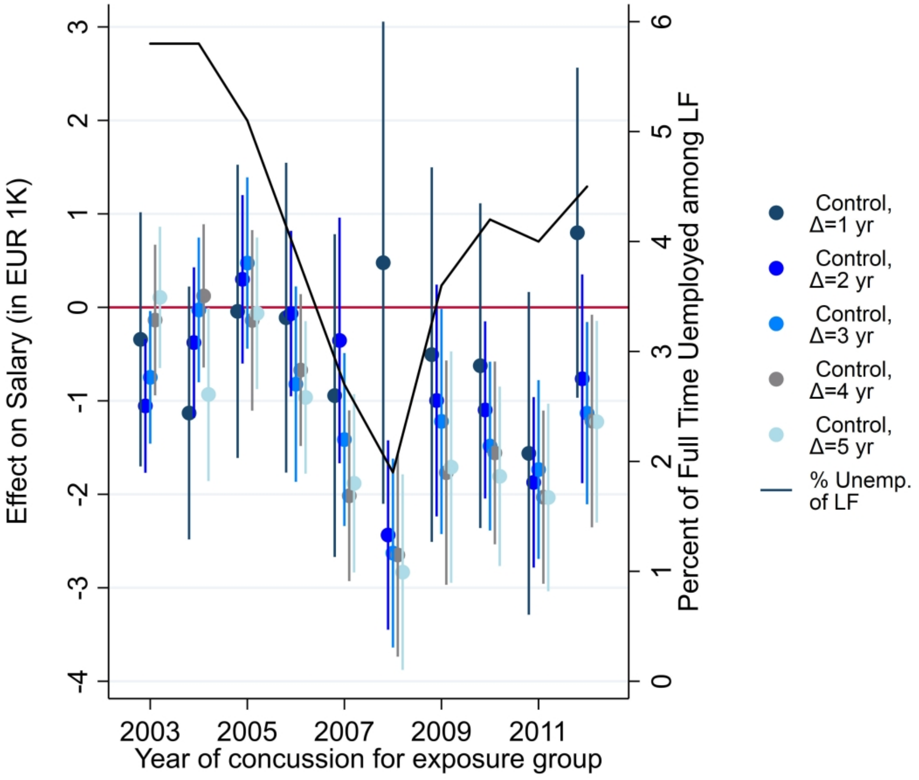
Effect on Salary (in EUR 1K)
Percent of Full Time Uemployed among LF
Year of concussion for exposure group
3
2
1
0
-1
-2
-3
-4
6
5
4
3
2
1
0
2003
2005
2007
2009
2011
Control, Δ=1 yr
Control, Δ=2 yr
Control, Δ=3 yr
Control, Δ=4 yr
Control, Δ=5 yr
% Unemp. of LF

Figure 4

SUPPLEMENTAL MATERIALS AND METHODS

Our quasi-experimental, difference-in-differences event time approach compares two groups of individuals from the same cohort, where both groups experience concussions, but at two different time points (t_c,t_c+Δ). For the simple situation where we have three periods (t=0,1,2) and the exposure group (T) experiences their concussion at the start of period 1 (t_c=1), and the control group (C) at the start of period 2 (t_c+Δ=2), the effect of concussion on salary (Y) is:

$$\Delta = \left(Y_1^T - Y_1^C\right) - \left(Y_0^T - Y_0^C\right)$$

The effect of concussion on salary in t=1 is estimated by comparing the average difference in salary between exposure and control groups for the post-concussion period t=1 $\left(Y_1^T - Y_1^C\right)$ to the average difference in salary for the pre-concussion, or baseline, interval t=0 $(Y_0^T - Y_0^C)$. Assuming the exact timing of a concussion is random for small enough sizes of Δ, and under the additional assumption that the exposure group would have had parallel trends in salary as the control group absent suffering concussion at t_c, δ captures the causal effect of concussion among those who suffer concussions – also known as the average effect on the treated (AT). The AT does not capture how concussions would affect a random person. The AT captures how concussions causally affect those who suffer concussions.

For our study, the parallel trends assumption states that exposure and control groups have parallel developments in salary leading up to the exposure group's concussion and the exposure and control groups would have further exhibited parallel salary trajectories if the concussion had not occurred. To test the parallel trends assumption, we estimate a dynamic version of the model specification (shown in supplementary table S1), which explicitly allows us to test whether the parallel trend assumption for our sample is probable.

To validate that the timing of concussion is random with our study period, we present estimates for effect of exposure across different periods between exposure and control incident (Δ). Most recorded concussions outside contact sports and military engagements stem from unforeseen events, such as falls or striking/being struck by an object[18,19], so assuming random timing is likely valid. People who regular engage in activities that result in high risk of multiple concussions may be different than the average concussion patient and would be more likely to end up in the exposure sample than in the control sample, which could induce bias. To avoid such potential bias, we restrict our sample to individuals without prior diagnoses for intracranial injuries ten years prior to exposure.

At t=-1, i.e. one year before the exposure group suffered a concussion, the control groups were slightly smaller than the exposure group, and two control groups (Δ=4 and 5) differed slightly but significantly in terms of average patient age (p <. 001; supplementary table S2), male to female ratio (p <. 001), and for control group Δ=5, in the frequency of individuals with at least a high school degree (p < .001). However, the differences are numerically small. To test that composition differences between exposure and control do not drive our results, we provide separate results for individuals with and without high school degree, for males and females, and for different age groups across all different values of Δ.

Further, our design inherently leads to the possibility of timing issues—our exposure group always suffers their concussion earlier (in terms of calendar time and age) than the control groups do. If the labor market is constantly improving or worsening during the period we consider, this could substantially influence our results. Therefore, we also estimate separate models across exposure incident year and control group. Estimating separate models allow us the added benefit of being able to examine whether the business cycle influences the effect of concussions on salary.

Statistical model

To estimate the impact of concussion on salary, we define the following variables: Exposure or control group g, which includes individuals i, at times to exposure-groups concussion incident t. First, we estimate a standard difference in differences model for each separate control group Δ={1, 2, 3, 4, and 5} using ordinary least squares:

$$Salary_{git} = \beta_0 + \gamma exposure_g + \theta post_t + \delta post \times exposure_{git} + \boldsymbol{X_i \beta} + \sum_{Age=26}^{48+\Delta} I(Age)\eta_{age} + \sum_{year=1999}^{2012} I(year)\,\eta_{year} + \epsilon_{git} \qquad \text{(S1)}$$

where $Salary_{git}$ measures annual salaried income deflated to 2015-level, $exposure_g$ indicates whether the observation belongs to the exposure or control group, $post_t$ captures the period after the exposure group's concussion occurred, and $post_t \times exposure_{git}$ captures the effect concussion, measured as share of year t ≥ 0 affected by concussion. In this way, someone who suffers a concussion July 1 has $post_t \times exposure_{git}$ = 0.5 for t = 0 and $post_t \times exposure_{git}$ = 1 for t > 0. $\boldsymbol{X_i}$ is a set of covariates that includes a high school indicator and a gender dummy, ϵ_{git} is the error-term, and the two last sets of indicator variables $I(Age)$ and $I(Year)$ capture age and incident year levels (control group indexed against incident year). Under the parallel trends assumption, δ then captures the annual effect of concussion on salary. In eq. 1, $exposure_g$ normalizes any pre-exposure differences between the exposure and control group, thereby creating a joint baseline pre-exposure.

We estimate robust individual-level clustered standard errors to account for the possibility that individuals enter the data twice both as control (0) and exposure (1) individuals (g={0,1}), and that they are observed for multiple periods (t={-4,…, Δ-1}). To calculate the relative salary decrease after concussion, we exploit the parallel trends assumption to generate the expected counterfactual salary level, i.e. had the concussion not occurred, and calculate the decline expressed in percentage as: $\%\,\text{change} = \delta \,/\, E(\widehat{Salary}_{git} | g = 1, post_t = 1, post_t \times exposure_{git} = 0)$. In this way, we provide both absolute estimates measured in 1K Euro, as well as percentage change.

We expect δ from eq. (1) to likely be negative. Yet, a decrease in annual salary can arrive through two different channels. Concussions may affect salary through either decreasing income among those employed or by reducing the number of individuals who are employed and earning any salary at. To parse out which of the two channels is driving the results, we examine how concussion affects the salary distribution among the exposure group following. Following Chernozhukov et al.[20] we estimate a series of regressions across the whole salary distribution, where, for a finite set of points, we predict how concussion affects the likelihood of having earnings on the left side of each finite point, as follows:

$$\sum_{j=0}^{\max(Salary)} p_j = \beta_{0j} + \delta_j post_t \times exposure_{git} + \theta post_t + \gamma_j exposure_g + \boldsymbol{X_i \beta} + \sum_{Age=26}^{48+\Delta} I(Age)\eta_{age,j} + \sum_{year=1999}^{2012} I(year)\,\eta_{year,j} + \epsilon_{git,j} \qquad \text{(S2)}$$

where $p_j = \Pr\,(Salary_{git} \leq j)$ and j is the interval from 0 to $max(Salary)$. Across the salary distribution, we can now predict the probability of earning less than j for those with and without concussions. From equation 2, we predict $p_j^1 = E(p_j | post_t \times exposure_{git} = 1, exposure_g = 1, t \geq 0)$ and the counterfactual $p_j^0 = E(p_j | post_t \times exposure_{git} = 0, exposure_g = 1, t \geq 0)$. Plotting p_j^1 and p_j^0 over each value of salary j, and assuming rank stability, gives the cumulative density function of salary for the treated (p_j^1) and the counterfactual observation of the treated had they not suffered concussions (p_j^0). The difference between p_j^1 and p_j^0 is simply δ_j. If the value of δ_j monotonically moves towards zero as j increases until $p_j^1 \approx p_j^0 \approx 1$ it indicates that exit from employment fully drives the effect of concussion on salary. If instead the value of δ_j is constant or increasing across parts of the distribution, it instead indicates that a decrease in salary among those still receiving salary drives at least part of the effect.

Eq. 1 and eq. 2 are based on the parallel trends assumption. The assumption states that exposure and control groups follow parallel salary trajectories until individuals in the exposure group experiences a concussion, and that the parallel trends would have continued had the concussion not occurred. Whereas we cannot verify the counterfactual situation of parallel trends after exposure, we can use a dynamic model to test for systematic differences in salary trends between exposure and control group in the years leading up to the exposure group's concussion event. To do so, we estimate the following dynamic model:

$$Salary_{git} = \beta_0 + \sum_{t \neq -1, t=-4}^{\Delta-1} \delta_t \times I(t_g) \times exposure_g + \sum_{t=-4}^{\Delta-1} I(t_g)\eta_t + \gamma exposure_g + \boldsymbol{X_i \beta} + \sum_{Age=26}^{48+\Delta} I(Age)\eta_{age} + \sum_{year=1999}^{2012} I(year)\,\eta_{year} + \epsilon_{git} \qquad \text{(S3)}$$

Where we interact exposure group status ($exposure_g$) with indicators $I(t_g)$ capturing time from concussion. If the parallel trends assumption holds, then it must be the case {δ_{-4}, δ_{-3}, δ_{-2}}=0, whereas the size and sign of {$\delta_0 \ldots \delta_{\Delta-1}$} captures the dynamic effect of a concussion from the year of incidence and Δ-1 years onward. By estimating the effect of concussion on salary among different years of the study period, we are also able to capture how the impact of concussion on salary evolves year to year after the concussion has occurred. We further estimate eq. 3 for a series of related labor market outcomes (annual total income, annual amount of sickness benefits received, annual probability of being employed), to generate a more thorough understanding on how concussions affect labor market outcomes—i.e., if people experience a decrease in salary due to a concussion, are they then compensated through different types of welfare state services.

For peer review only

Supplemental results

For peer review only

For peer review only - http://bmjopen.bmj.com/site/about/guidelines.xhtml

Table S1. Test of parallel trends assumption pre-exposure incident against each control group separately using eq. S3 in supplementary methods. Separate exposure dummies for all time periods (except the year prior to exposure, which serves as reference period).

Time to exposure group's concussion (exposure)	Δ=1 Est (S.E.) p-value	Δ=2 Est (S.E.) p-value	Δ=3 Est (S.E.) p-value	Δ=4 Est (S.E.) p-value	Δ=5 Est (S.E.) p-value
**Exposure-4y**	-0.368 (0.226) p=.104	0.046 (0.363) p=.900	0.120 (0.362) p=.741	0.159 (0.313) p=.612	0.042 (0.329) p=.899
**Exposure-3y**	-0.094 (0.317) p=.768	0.227 (0.510) p=.656	0.167 (0.354) p=.637	0.537 (0.372) p=.148	0.113 (0.393) p=.774
**Exposure-2y**	-0.548 (0.312) p=.079	-0.082 (0.347) p=.812	-0.163 (0.236) p=.491	-0.124 (0.247) p=.617	0.082 (0.250) p=.744
**Exposure-1y**	Ref.	Ref.	Ref.	Ref.	Ref.
**N*T**	284115	273725	266120	260647	256337

Note: The table shows test for differences in pre-exposure trends between exposure and control group model using interactions between pre-exposure time dummies and the exposure indicator. There is no indication of substantial or significant pre-exposure differences in salary trajectories between exposure group and any of the control groups.

For peer review only

Table S2. **Effect of concussion on different labor market outcome parameters using separate exposure dummies for all time periods (except the year prior to exposure, which serves as reference period): In this exploratory analysis, the exposure group is compared to the control group Δ=5, which suffers a concussion five years after the exposure group. Outcomes include annual salaried income (annual salary), total annual income (total income), annual sick leave benefits received (sick leave benefits) as well as a binary indicator of employment (probability of employment). Monetary outcomes were measured at 2015-level in € 1,000.**

Time to exposure group's concussion (exposure)	Annual Salary Est. (S.E.) p-value	Total income Est. (S.E.) p-value	Sick leave benefits Est. (S.E.) p-value	Probability of employment Est. (S.E.) p-value
**Exposure-4y**	0.012<br>(0.212)<br>p=.954	0.164<br>(0.173)<br>p=.343	0.035<br>(0.036)<br>p=.320	0.001<br>(0.004)<br>p=.803
**Exposure-3y**	0.059<br>(0.252)<br>p=.814	0.305<br>(0.233)<br>p=.190	0.022<br>(0.034)<br>p=.529	-0.001<br>(0.003)<br>p=.739
**Exposure-2y**	0.043<br>(0.160)<br>p=.788	0.122<br>(0.147)<br>p=.405	0.002<br>(0.029)<br>p=.946	0.001<br>(0.003)<br>p=.739
**Exposure-1y**				
**Exposure**	-0.611<br>(0.168)<br>p<.001	-0.338<br>(0.140)<br>0.016	0.166<br>(0.030)<br>p<.001	-0.003<br>(0.003)<br>p=.317
**Exposure+1y**	-1.389<br>(0.209)<br>p<.001	-0.608<br>(0.162)<br>p<.001	0.288<br>(0.039)<br>p<.001	-0.020<br>(0.003)<br>p<.001
**Exposure+2y**	-1.568<br>(0.261)<br>p<.001	-0.847<br>(0.231)<br>p<.001	0.132<br>(0.039)<br>p=.001	-0.023<br>(0.004)<br>p<.001
**Exposure+3y**	-1.393<br>(0.246)<br>p<.001	-0.497<br>(0.219)<br>p=.023	0.031<br>(0.040)<br>p=.432	-0.022<br>(0.004)<br>p<.001
**Exposure+4y**	-1.319<br>(0.253)<br>p<.001	-0.499<br>(0.218)<br>p=.022	-0.076<br>(0.042)<br>p=.075	-0.018<br>(0.004)<br>p<.001
**N*T**	577762	577758	577872	577872

Note: Annual salary include all income from salary and employee fringe benefits, employee stock options, employer paid sick leave, net gains (including interests and capital gains) from own companies. Total income includes all income absent wealth. Sick leave includes only public health benefits (sick leave and paternity leave). Employment is a binary indicator measured last week of November for each year. Results obtained from estimations following Eq. (1). Models include controls for high school diploma, gender, age, and observation year. Results obtained using reghdfe in Stata. Total number of observations (N*T) differ slightly between outcomes because all income information is not available for all observation all years.

Source: Own calculations on data from Statistics Denmark.

For peer review only

Table S3. **Demographic factors for exposure group and control groups (Δ=1, 2, 3, 4, 5) averaged over the 5 years leading up to the concussion event in each of the groups. Factors include patient age (in years), share of sample female (1=100% female), and share of individuals with at least a high school degree (1=100%).**

		Exposure	Control, Δ=1	Control, Δ=2	Control, Δ=3	Control, Δ=4	Control, Δ=5
**Pr(Female=1)**	Mean	.430	.438	.447	.458	.464	.473
	S.D.	(.495)	(.496)	(.497)	(.498)	(.499)	(.499)
	p-value		.030	<.001	<.001	<.001	<.001
**Age**	Mean	36.899	37.354	37.754	38.065	38.343	38.592
	S.D.	(11.856)	(11.857)	(11.718)	(11.630)	(11.584)	(11.491)
	p-value		<.001	<.001	<.001	<.001	<.001
**Pr(High school=1)**	Mean	.624	.632	.640	.646	.653	.660
	S.D.	(.484)	(.482)	(.480)	(.478)	(.476)	(.474)
	p-value		.026	<.001	<.001	<.001	<.001
**Total individuals**		37848	34551	31851	29922	28580	27484

Note: S.D.: Standard deviation. P-values calculated using two-sided t-tests. All test performed between exposure group and each control group separately.

For peer review only

Figure S1. **Unnormalized Average Salary for Treatment and Control Groups Measured in 1K € at 2015-levels**

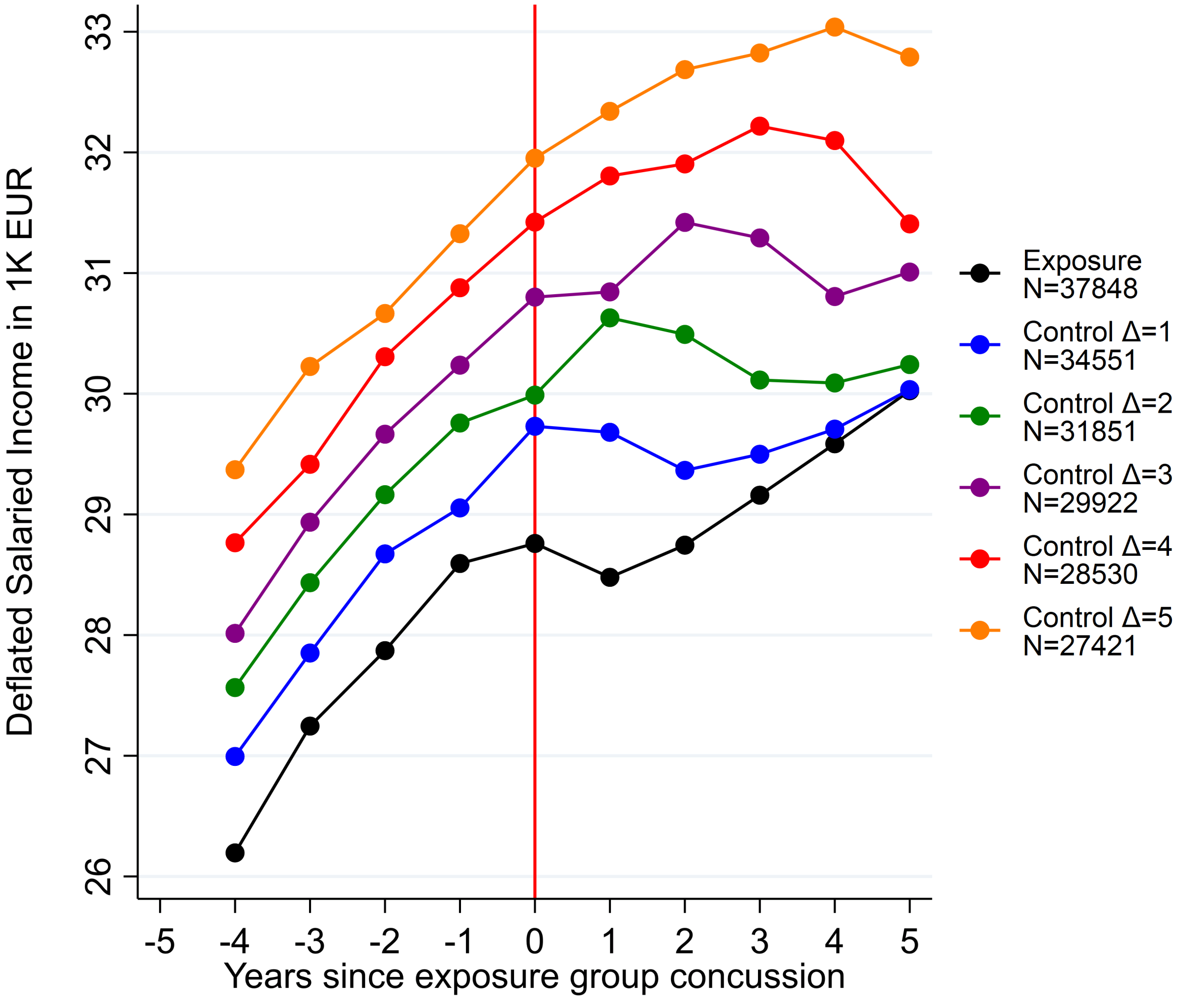

Note: Salary of the exposure group compared to salary of the 5 control groups, who experienced their concussions Δ={1, 2, 3, 4, and 5} years later than the exposure group. Salary progression is shown for the 5 years before and the 5 years after the exposure group suffered a concussion event. Table S1 demonstrates that the trends for salary progression pre-exposure incident are parallel between exposure group and each control group.

Figure S2. **The Cumulative Density Function (CDF) for Total Income Post-Treatment among the Treatment Group and Their Counterfactual, and the Difference between the Two CDFs Expressed as the Effect of Concussion on the Probability of Total Income Below that Income-Level following Exposure Event.**

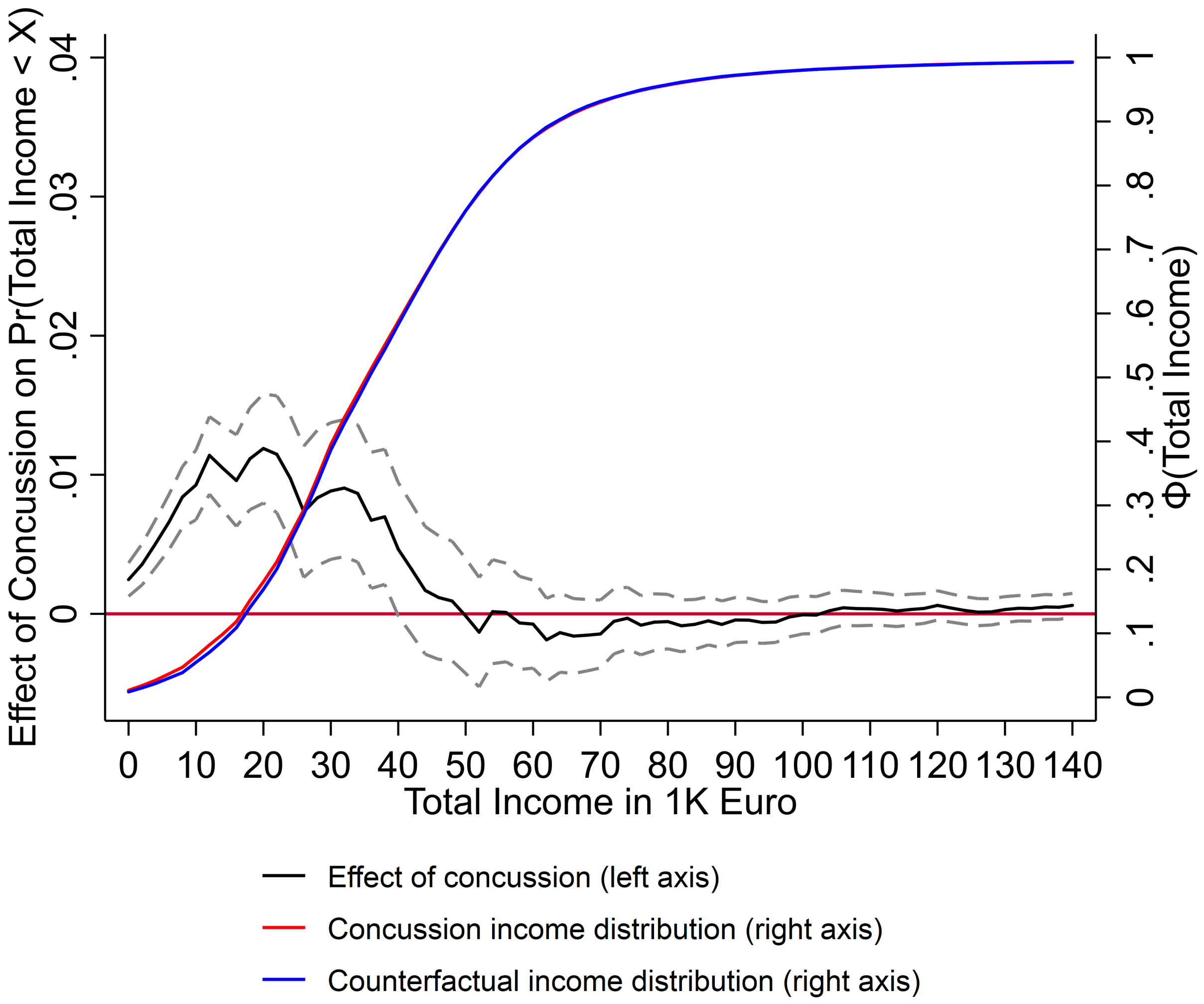

Note: The figure shows the observed cumulative salary distribution following a concussion (red) and the expected counterfactual salary distribution absent the concussion (blue). The black line shows the difference between the observed and the counterfactual distribution, and the grey dash lines show the 95 % confidence interval. The bell-shape of the difference between the two distributions as the total income increase from 0 to 40,000 € indicates that the main part of the effect of concussions on total incomes is driven by low-income people shifting total income downwards following concussion, but not going to total income equal to zero.

Figure S3. Percentage Effect of Concussion on Relative Salary Across Age Groups.

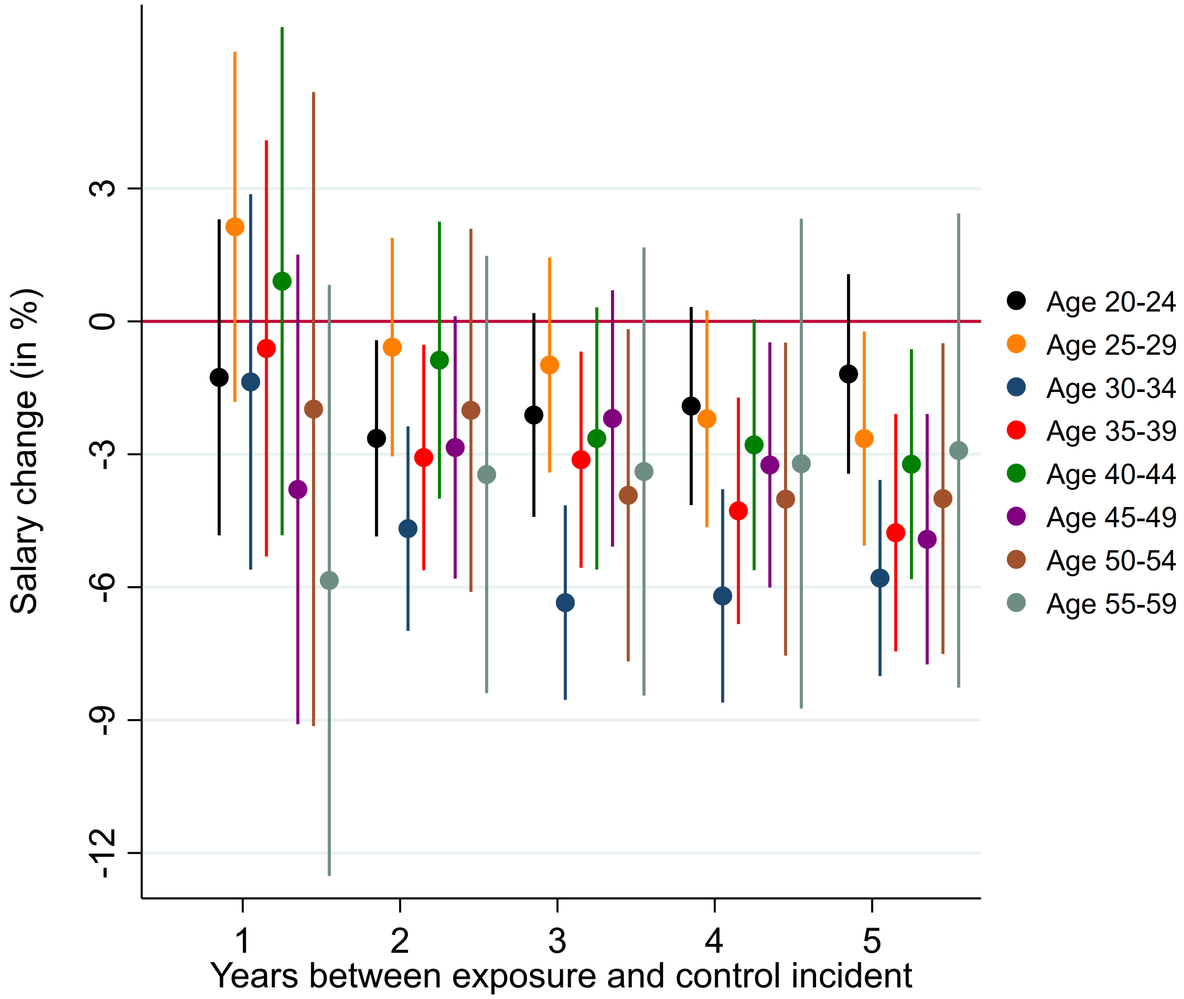

Note: Relative salary drop of the exposure group compared to the control groups, who suffer a concussion 1, 2, 3, 4, and 5 years after the exposure group. Relative salary changes are shown across different age groups. Graph shows parameter estimates and 95% CI.

Figure S4. **Percentage Effect of Concussion on Relative Salary Across High School Completion.**

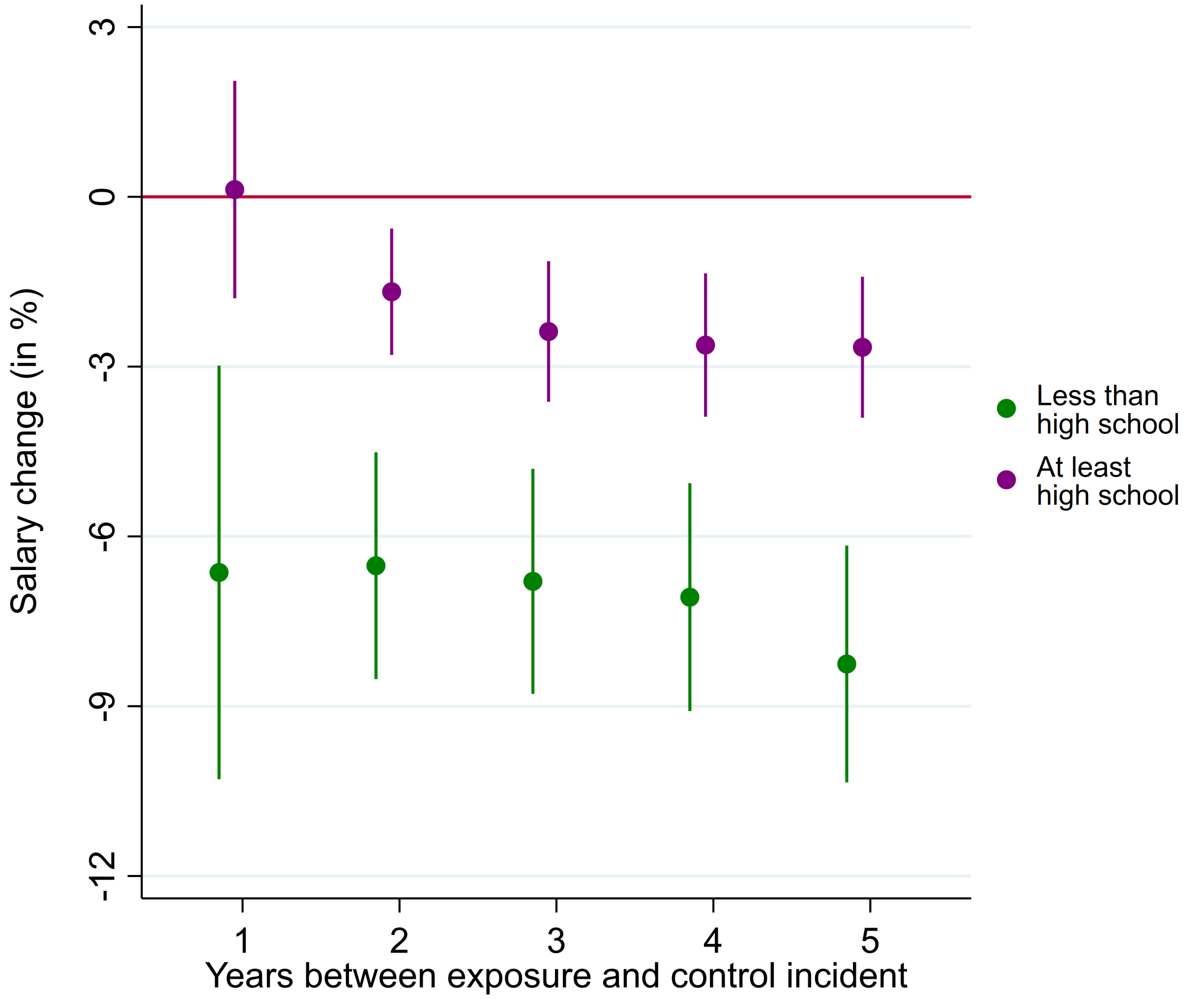

Note: Relative salary drop of the exposure group compared to the control groups, who suffer a concussion 1, 2, 3, 4, and 5 years after the exposure group. Relative salary changes are shown across whether individuals had obtained at least a high school diploma (ISCED > 2). Graph shows parameter estimates and 95% CI.

For peer review only - http://bmjopen.bmj.com/site/about/guidelines.xhtml

Figure S5. **Percentage Effect of Concussion on Relative Salary Across Gender.**

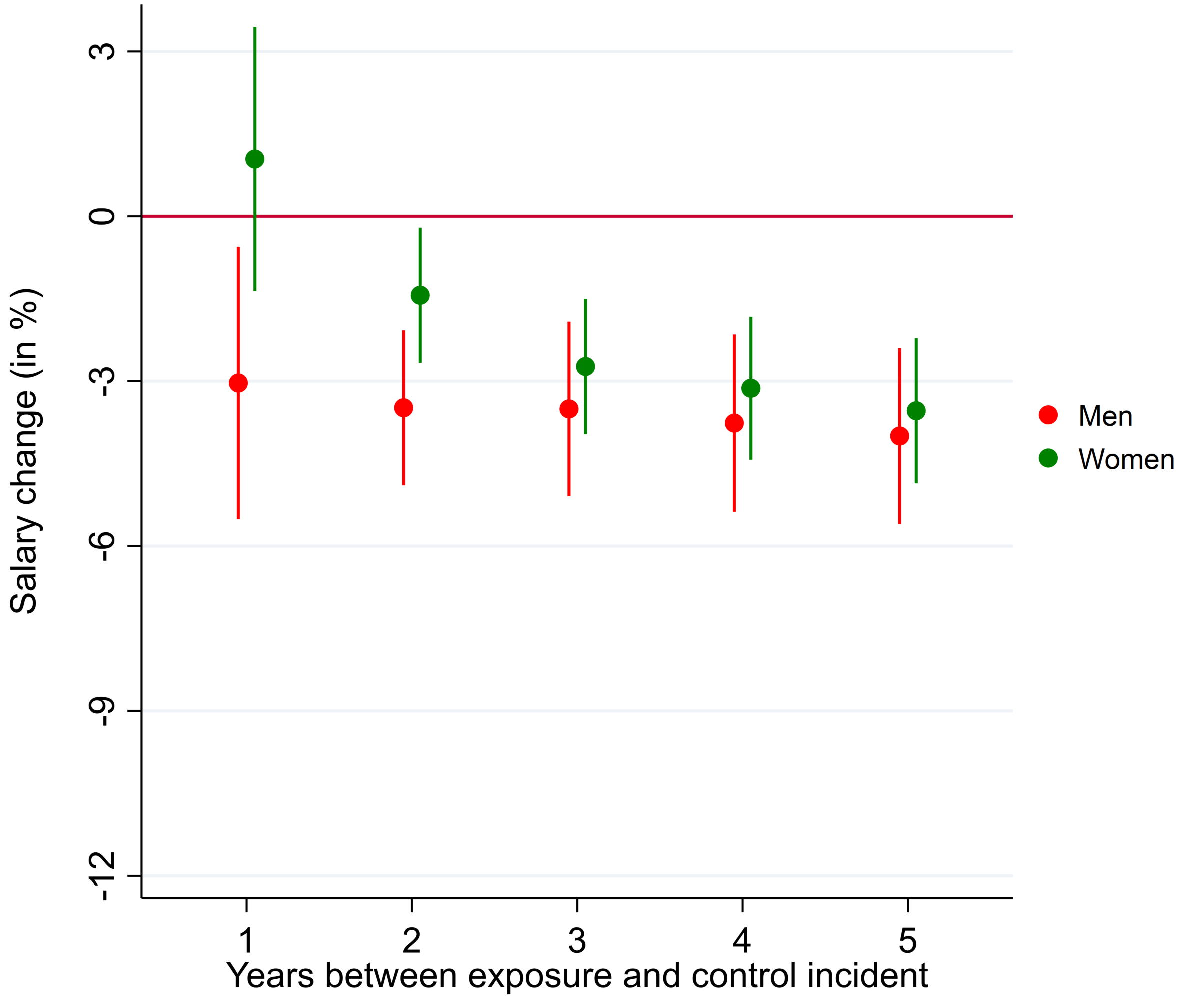

Note: Relative salary drop of the exposure group compared to the control groups, who suffer a concussion 1, 2, 3, 4, and 5 years after the exposure group. Relative salary changes are shown across gender. Graph shows parameter estimates and 95% CI.

For peer review only - http://bmjopen.bmj.com/site/about/guidelines.xhtml

Figure S6. **Effect of Concussion on Absolute Salary in 1K Euro Across Age groups.**

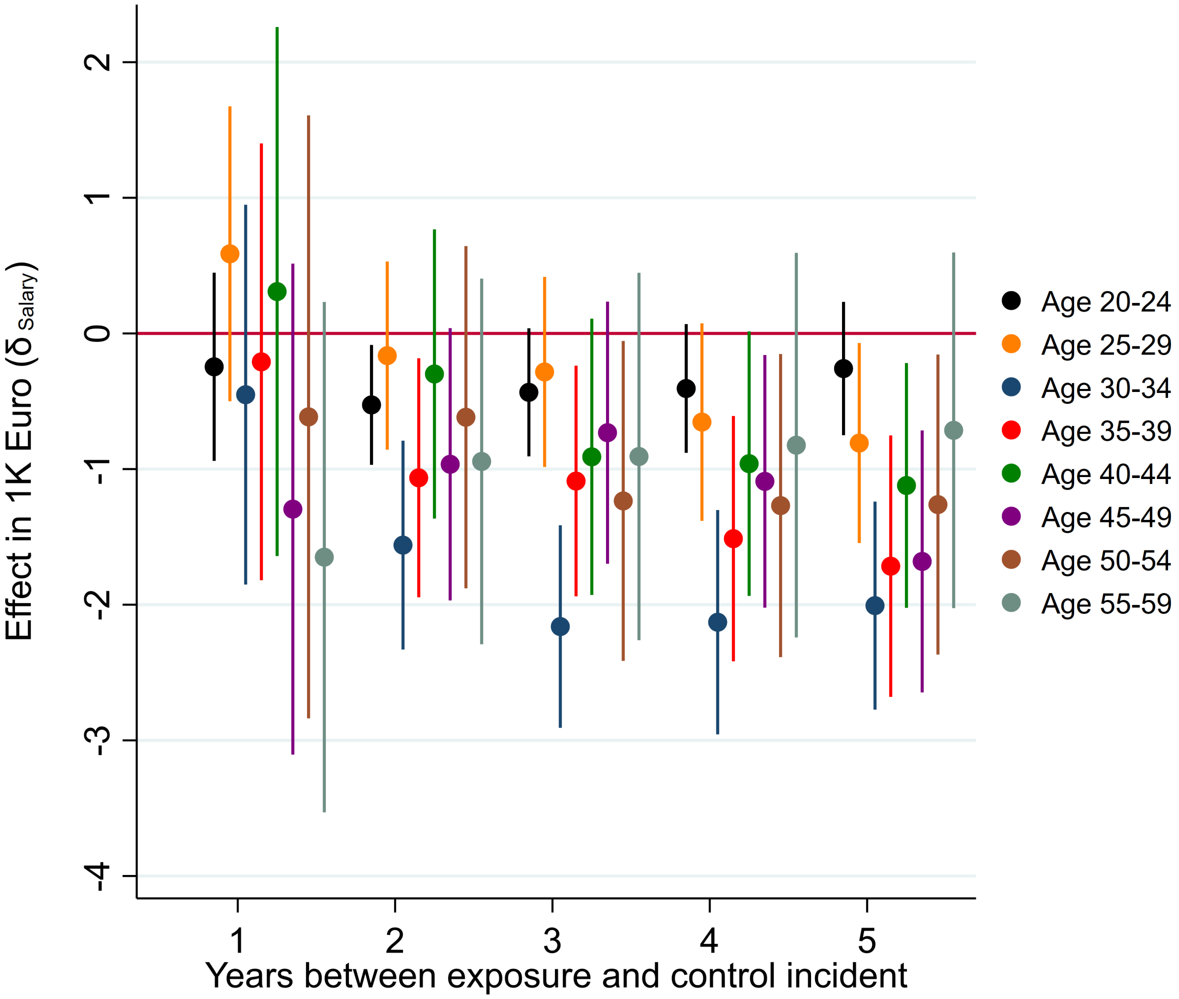

Note: Absolute salary drop of the exposure group compared to the control groups, who suffer a concussion 1, 2, 3, 4, and 5 years after the exposure group. Absolute salary changes are shown across different age groups. Graph shows parameter estimates and 95% CI.

only

Figure S7. **Effect of Concussion on Absolute Salary in 1K Euro Across Education.**

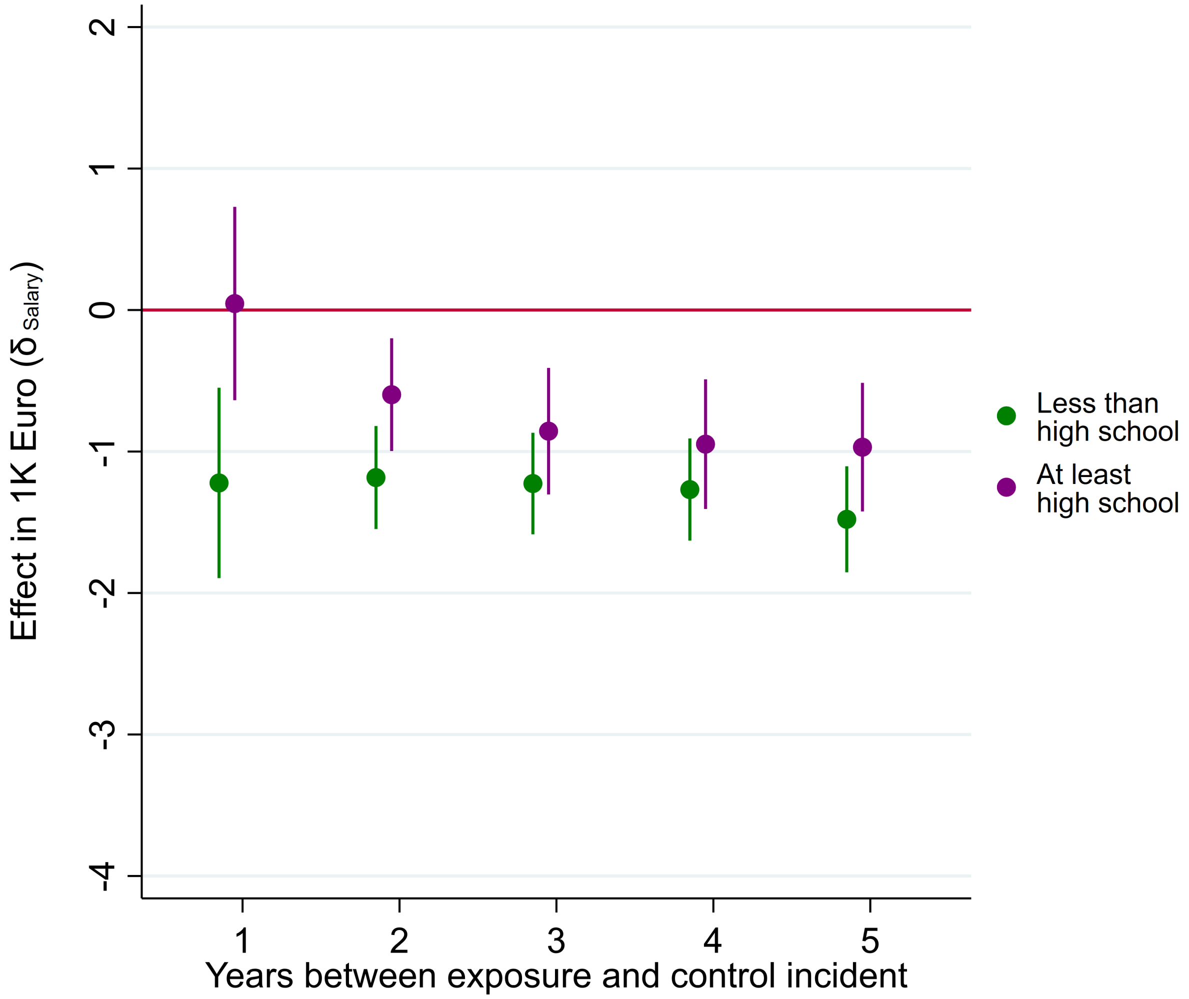

Note: Absolute salary drop of the exposure group compared to the control groups, who suffer a concussion 1, 2, 3, 4, and 5 years after the exposure group. Absolute changes in salary are shown across whether individuals had obtained at least a high school diploma (ISCED > 2). Graph shows parameter estimates and 95% CI.

only

Figure S8. **Effect of Concussion on Absolute Salary in 1K Euro Across Gender.**

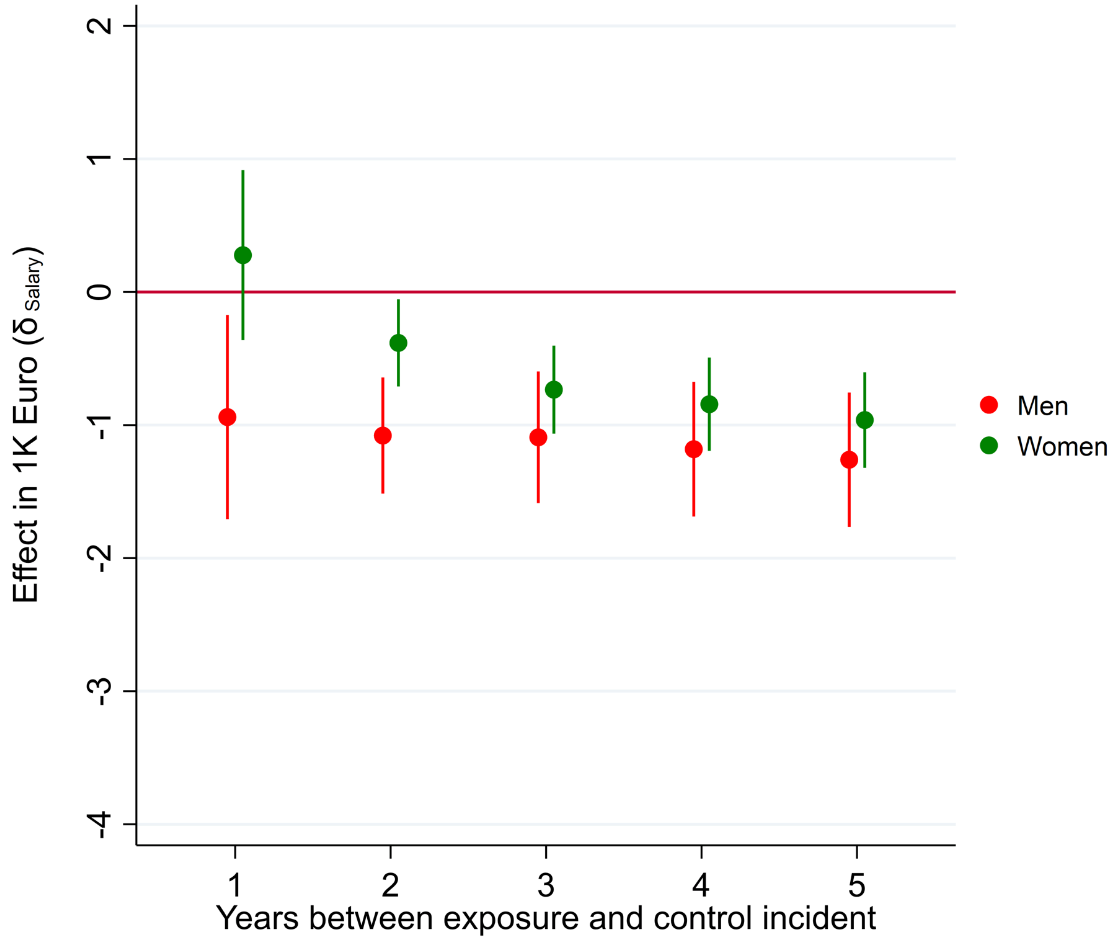

Note: Absolute salary drop of the exposure group compared to the control groups, who suffer a concussion 1, 2, 3, 4, and 5 years after the exposure group. Absolute changes in salary are shown across gender. Graph shows parameter estimates and 95% CI.

For peer review only - http://bmjopen.bmj.com/site/about/guidelines.xhtml

The RECORD statement – checklist of items, extended from the STROBE statement, that should be reported in observational studies using routinely collected health data.

	Item No.	STROBE items	Location in manuscript where items are reported	RECORD items	Location in manuscript where items are reported
**Title and abstract**					
	1	(a) Indicate the study's design with a commonly used term in the title or the abstract (b) Provide in the abstract an informative and balanced summary of what was done and what was found	title<br>abstract	RECORD 1.1: The type of data used should be specified in the title or abstract. When possible, the name of the databases used should be included.<br><br>RECORD 1.2: If applicable, the geographic region and timeframe within which the study took place should be reported in the title or abstract.<br><br>RECORD 1.3: If linkage between databases was conducted for the study, this should be clearly stated in the title or abstract.	title<br>abstract<br><br>title<br>abstract<br><br>abstract
**Introduction**					
Background rationale	2	Explain the scientific background and rationale for the investigation being reported	abstract<br>introduction		
Objectives	3	State specific objectives, including any prespecified hypotheses	introduction		
**Methods**					
Study Design	4	Present key elements of study design early in the paper	introduction		
Setting	5	Describe the setting, locations, and relevant dates, including periods of recruitment, exposure, follow-up, and data collection	materials and methods		

For peer review only

Participants	6	*(a) Cohort study* - Give the eligibility criteria, and the sources and methods of selection of participants. Describe methods of follow-up<br>*Case-control study* - Give the eligibility criteria, and the sources and methods of case ascertainment and control selection. Give the rationale for the choice of cases and controls<br>*Cross-sectional study* - Give the eligibility criteria, and the sources and methods of selection of participants<br><br>*(b) Cohort study* - For matched studies, give matching criteria and number of exposed and unexposed<br>*Case-control study* - For matched studies, give matching criteria and the number of controls per case	materials and methods	RECORD 6.1: The methods of study population selection (such as codes or algorithms used to identify subjects) should be listed in detail. If this is not possible, an explanation should be provided.<br><br>RECORD 6.2: Any validation studies of the codes or algorithms used to select the population should be referenced. If validation was conducted for this study and not published elsewhere, detailed methods and results should be provided.<br><br>RECORD 6.3: If the study involved linkage of databases, consider use of a flow diagram or other graphical display to demonstrate the data linkage process, including the number of individuals with linked data at each stage.	materials and methods<br><br>materials and methods<br><br>not included
Variables	7	Clearly define all outcomes, exposures, predictors, potential confounders, and effect modifiers. Give diagnostic criteria, if applicable.	materials and methods<br>main text	RECORD 7.1: A complete list of codes and algorithms used to classify exposures, outcomes, confounders, and effect modifiers should be provided. If these cannot be reported, an explanation should be provided.	materials and methods
Data sources/ measurement	8	For each variable of interest, give sources of data and details of methods of assessment (measurement).<br>Describe comparability of assessment methods if there is more than one group	materials and methods		

For peer review only

Bias	9	Describe any efforts to address potential sources of bias	materials and methods and results		
Study size	10	Explain how the study size was arrived at	materials and methods		
Quantitative variables	11	Explain how quantitative variables were handled in the analyses. If applicable, describe which groupings were chosen, and why	materials and methods		
Statistical methods	12	(a) Describe all statistical methods, including those used to control for confounding<br>(b) Describe any methods used to examine subgroups and interactions<br>(c) Explain how missing data were addressed<br>(d) *Cohort study* - If applicable, explain how loss to follow-up was addressed<br>*Case-control study* - If applicable, explain how matching of cases and controls was addressed<br>*Cross-sectional study* - If applicable, describe analytical methods taking account of sampling strategy<br>(e) Describe any sensitivity analyses	a) materials and methods<br>b) materials and methods<br>c) materials and methods<br>d-e) NA		
Data access and cleaning methods		..		RECORD 12.1: Authors should describe the extent to which the investigators had access to the database population used to create the study population.	materials and methods<br><br>materials and methods

For peer review only

				RECORD 12.2: Authors should provide information on the data cleaning methods used in the study.	
Linkage		..		RECORD 12.3: State whether the study included person-level, institutional-level, or other data linkage across two or more databases. The methods of linkage and methods of linkage quality evaluation should be provided.	materials and methods
**Results**					
Participants	13	(a) Report the numbers of individuals at each stage of the study (*e.g.*, numbers potentially eligible, examined for eligibility, confirmed eligible, included in the study, completing follow-up, and analysed)<br>(b) Give reasons for non-participation at each stage.<br>(c) Consider use of a flow diagram	(a-c) materials and methods	RECORD 13.1: Describe in detail the selection of the persons included in the study (*i.e.,* study population selection) including filtering based on data quality, data availability and linkage. The selection of included persons can be described in the text and/or by means of the study flow diagram.	materials and methods
Descriptive data	14	(a) Give characteristics of study participants (*e.g.*, demographic, clinical, social) and information on exposures and potential confounders<br>(b) Indicate the number of participants with missing data for each variable of interest<br>(c) *Cohort study* - summarise follow-up time (*e.g.*, average and total amount)	a) materials and methods and Supplementary Table S3<br><br>b) materials and methods,Table 1<br><br>c) materials and methods		
Outcome data	15	*Cohort study* - Report numbers of outcome events or summary measures over time<br>*Case-control study* - Report numbers in each exposure	materials and methodsand Supplementary Table S3, results		

For peer review only

		category, or summary measures of exposure<br>*Cross-sectional study* - Report numbers of outcome events or summary measures			
Main results	16	(a) Give unadjusted estimates and, if applicable, confounder-adjusted estimates and their precision (e.g., 95% confidence interval). Make clear which confounders were adjusted for and why they were included<br>(b) Report category boundaries when continuous variables were categorized<br>(c) If relevant, consider translating estimates of relative risk into absolute risk for a meaningful time period	a) results<br>b) results<br>c) results		
Other analyses	17	Report other analyses done—e.g., analyses of subgroups and interactions, and sensitivity analyses	results		
**Discussion**					
Key results	18	Summarise key results with reference to study objectives	results and discussion		
Limitations	19	Discuss limitations of the study, taking into account sources of potential bias or imprecision. Discuss both direction and magnitude of any potential bias	results and discussion	RECORD 19.1: Discuss the implications of using data that were not created or collected to answer the specific research question(s). Include discussion of misclassification bias, unmeasured confounding, missing data, and changing eligibility over time, as they pertain to the study being reported.	NA
Interpretation	20	Give a cautious overall interpretation of results considering objectives,	discussion		

		limitations, multiplicity of analyses, results from similar studies, and other relevant evidence			
Generalisability	21	Discuss the generalisability (external validity) of the study results	discussion		
**Other Information**					
Funding	22	Give the source of funding and the role of the funders for the present study and, if applicable, for the original study on which the present article is based	Title page		
Accessibility of protocol, raw data, and programming code		..	Reference to supplementary data throughout the text	RECORD 22.1: Authors should provide information on how to access any supplemental information such as the study protocol, raw data, or programming code.	materials and methods

*Reference: Benchimol EI, Smeeth L, Guttmann A, Harron K, Moher D, Petersen I, Sørensen HT, von Elm E, Langan SM, the RECORD Working Committee. The REporting of studies Conducted using Observational Routinely-collected health Data (RECORD) Statement. *PLoS Medicine* 2015; in press.

*Checklist is protected under Creative Commons Attribution (CC BY) license.

For peer review only

The Effect of Concussion on Salary and Employment-A Population-Based Event Time Study using a Quasi-Experimental Design

Journal:	*BMJ Open*
Manuscript ID	bmjopen-2020-038161.R1
Article Type:	Original research
Date Submitted by the Author:	21-Jul-2020
Complete List of Authors:	Fallesen, Peter; Stockholm University, Swedish Institute for Social Research<br>Campos, Benito; Heidelberg University Hospital Department of Neurosurgery; Technical University of Denmark, Department of Health Tech
**Primary Subject Heading**:	Public health
Secondary Subject Heading:	Neurology
Keywords:	HEALTH ECONOMICS, Neurosurgery < SURGERY, Trauma management < ORTHOPAEDIC & TRAUMA SURGERY

SCHOLARONE™
Manuscripts

I, the Submitting Author has the right to grant and does grant on behalf of all authors of the Work (as defined in the below author licence), an exclusive licence and/or a non-exclusive licence for contributions from authors who are: i) UK Crown employees; ii) where BMJ has agreed a CC-BY licence shall apply, and/or iii) in accordance with the terms applicable for US Federal Government officers or employees acting as part of their official duties; on a worldwide, perpetual, irrevocable, royalty-free basis to BMJ Publishing Group Ltd ("BMJ") its licensees and where the relevant Journal is co-owned by BMJ to the co-owners of the Journal, to publish the Work in this journal and any other BMJ products and to exploit all rights, as set out in our licence.

The Submitting Author accepts and understands that any supply made under these terms is made by BMJ to the Submitting Author unless you are acting as an employee on behalf of your employer or a postgraduate student of an affiliated institution which is paying any applicable article publishing charge ("APC") for Open Access articles. Where the Submitting Author wishes to make the Work available on an Open Access basis (and intends to pay the relevant APC), the terms of reuse of such Open Access shall be governed by a Creative Commons licence – details of these licences and which Creative Commons licence will apply to this Work are set out in our licence referred to above.

Other than as permitted in any relevant BMJ Author's Self Archiving Policies, I confirm this Work has not been accepted for publication elsewhere, is not being considered for publication elsewhere and does not duplicate material already published. I confirm all authors consent to publication of this Work and authorise the granting of this licence.

For peer review only

The Effect of Concussion on Salary and Employment-A Population-Based Event Time Study using a Quasi-Experimental Design

[1,2]Peter Fallesen, PhD, [3,4]Benito Campos, M.D.

[1]Swedish Institute for Social Research, Stockholm University, SE-106 91 Stockholm, Sweden
[2]ROCKWOOL Foundation, Ny Kongensgade 6, DK-1472 Copenhagen C, Denmark
[3]Department of Neurosurgery, University of Heidelberg, INF400, 69120 Heidelberg, Germany
[4]Department of Health Tech, Technical University of Denmark, DK-2800 Kongens Lyngby, Denmark

Address of Correspondence: Peter Fallesen, Swedish Institute for Social Research, Stockholm University, SE-106 91 Stockholm, Sweden, peter.fallesen@sofi.su.se, +4561333184

Keywords: administrative data; concussion; income; productivity

Abstract word count: 245

Text word count: 3913 (excluding references and figure/table legends)

Number of references: 21

Number of tables and/or figures: 2 tables and 4 figures

Number of videos: 0

For peer review only

For peer review only - http://bmjopen.bmj.com/site/about/guidelines.xhtml

Abstract

Objective: Concussions are the most frequent traumatic brain injuries. Yet, the socio-economic impact of concussions remains unclear. Socio-economic effects of concussions on working age adults were studied on a population scale.

Design: This population-based, event time study uses administrative data as well as hospital and emergency room records for the population of Denmark.

Setting: We study all Danish patients, aged 20-59 y, who were treated at a public hospital or emergency room between 2003-2017 after suffering a concussion without other intracranial or extracranial injuries (n=55,424 unique individuals) with no prior diagnosis of intra- or extracranial injury within the past ten years leading up to the incident.

Primary and Secondary Outcome Measures: As primary endpoint, we investigate the mean effect of concussion on annual salaried income within a five-year period after trauma. In an exploratory analysis, we study whether the potential impact of concussion on annual salaried income is driven by patient age, education, or economic cycle.

Results: Concussion was associated with an average change in annual salary income of -1,223€ (95% CI, -1,540€; -905€, p<.001) corresponding to a salary change of -4.2 % (95% CI, -5.2 %; -3.1 %). People between 30-39 y and those without high school degrees suffered the largest salary decreases. Affected individuals leaving the workforce drove the main part of the decrease. Absolute annual effect sizes were countercyclical to the unemployment rate.

Conclusions: Concussions have a large and long-lasting impact on salary and employment of working-age adults on a nationwide scale.

For peer review only - http://bmjopen.bmj.com/site/about/guidelines.xhtml

Strengths and limitations of this study

- Natural experiments used to obtain plausible causal effects between concussion and salary/employment.
- Large, population-based sample with multiple data layers.
- Analysis includes how economic cycles affect outcome measures.
- Data only captures concussions registered in ERs and hospitals.
- Because concussions do not occur at random, causal estimate relies on stronger assumptions than for a randomized control trial.

For peer review only

For peer review only - http://bmjopen.bmj.com/site/about/guidelines.xhtml

Introduction

Concussions are by far the most frequently occurring intracranial injuries affecting approximately 450 to 600 per 100,000 people every year[1] . Immediate symptoms may last for days or weeks. Further, Danish cohort data[2] indicates that 10-15% of patients diagnosed with concussion suffer from long-term symptoms such as headache, fatigue, and intolerance to stress, whereas other studies place the upper bound as high as 30%[3,4]. Clinical practice has encouraged patients to restrict social, mental, and physical activity in the weeks following a concussion (see[5] for review), although prolonged inactivity may prolong symptoms. Thus, symptoms, comorbidities, and suggested treatment are associated with short- to long-term absence from work and lower productivity.

Yet, the causal effect of concussion on economic burdens for individuals and society through decreased labour market activity has not been identified. First, concussion is a sudden incident and thus not amenable to prospective study nor randomization. Cohort and case-control studies[6–10] provide some valuable evidence on employment and labour market outcomes among those who suffered concussions but are prone to selection bias. Individuals at high risk of concussions may differ on unobserved characteristics (e.g., risk aversion, routine activities) from those at low risk. People who are more likely to suffer concussions may also, on average, have more precarious or unstable employment trajectories prior to the incident, which may further bias prospective studies. Given the high incidence rate of concussion, even small losses of productivity and discrete drops in employment would have a significant socioeconomic impact and thus, it would require large patient cohorts with suitable controls to grasp the full socioeconomic impact of concussions. Thus, absent the possibility of randomization, using a

For peer review only

For peer review only - http://bmjopen.bmj.com/site/about/guidelines.xhtml

78 natural or quasi-experimental design is the only likely option to parse out the *causal* effect of
79 concussions on labour market activity.[11]

80 We examine how concussions affect salary and employment of working age individuals in
81 Denmark, a representative north-European industrial nation with a strong welfare state and a
82 flexible labour market. We use administrative longitudinal data linked to hospital and emergency
83 room diagnostic data on all Danes, who received a primary diagnosis of concussion between
84 2003 and 2017. To address the problem of unmeasured bias between those that do and do not
85 experience a concussion, we use a quasi-experimental event-study approach[12,13] where we
86 compare similar individuals, who experienced their concussions at different time points. Under
87 mild assumptions of parallel trends in wage progression prior to concussion and random timing
88 of concussion event within a five-year time frame, the approach recovers a robust estimation
89 of the effect of concussion on annual salary and employment status.

90

For peer review only - http://bmjopen.bmj.com/site/about/guidelines.xhtml

Material & Methods

Data Sources and Sample Construction

Concussion data originates from the Danish National Patient Registry (DNPR) (see[14] for description). DNPR is published annually and holds information on all hospitalizations at public hospitals in Denmark since 1977, on all emergency room visits and outpatient treatments at public hospitals since 1994, and almost all private hospital treatments since 2003. With one single, short-lived exception, private hospitals do not operate emergency rooms in Denmark. Since 2003, the data cover 95 % of all treatments at private hospitals[14], yet only 13 concussion were diagnosed in private hospital settings throughout the period covered by the data.

The combined exposure and control cohort includes all Danes aged 20-59 y, who received a primary diagnosis of concussion (ICD-10 code S06.0, ICD-8 code N850) between 2003 and 2017 and did not sustain any kind of additional intracranial or extracranial injury. Individuals who regularly engage in activities associated with a high risk of sustaining multiple concussions may differ from the average concussion patient and would likely be over-represented in the exposure sample. To avoid such potential bias, all individuals who were diagnosed with any kind of brain trauma during a ten-year period prior to the concussion event were excluded. Altogether, the study included a cohort of 55,424 individuals. Only attrition is through mortality and out-migration, and out-migrated or deceased patients with missing spells in the follow up period is excluded in those periods.

As a measure of productivity, a price-index deflated annual salaried income was used. Salary information comes from Statistics Denmark’s Income Statistics database. The database

For peer review only - http://bmjopen.bmj.com/site/about/guidelines.xhtml

includes all declared annual incomes including income from self-employment. The Danish Tax Authorities supply the data to Statistics Denmark. Overall accuracy is considered very good.[15] Table 1 reports number of observations for the samples and number of observations with missing salary information. As evident, only between 0.01 to 0.02 percent of observations across exposure and control groups have missing salary information. These observations were disregarded in the main analysis. Through social security numbers, information on salaried income were linked to records on diagnosed concussions. Further, information on high school or equivalent level degree at time of concussion was obtained using the Danish Education Database. The Danish Population Database provided demographic information on age and gender for all respondents. Since the data used in the study come from de-identified administrative registers that Statistics Denmark makes available for research purposes for approved institutions, no approval from an ethics committee was needed to carry out the study. The research was carried out as part of project no. 706630 approved by Statistics Denmark. Statistical analysis was carried out using Stata MP 15.1.

Quasi-experimental design

The study used a quasi-experimental, difference-in-differences event time approach previously described in a health setting by Dobkin et al.[12] The approach compare two groups of individuals from the same cohort, where both groups experience concussions, but at two different time points (t_c,t_c+Δ). Specifically, the sample of 55,496 individuals was divided into six different subgroups: i) The *exposure group*, which includes all patients, who suffered their concussion during the period 2003-2012 (n=37,848) and ii) five *control groups*, which comprise

For peer review only - http://bmjopen.bmj.com/site/about/guidelines.xhtml

patients who experienced their concussions Δ={1 (n=34,551), 2 (n=31,851), 3 (n=29,922), 4 (n=28,530), and 5 (n=27,421)} years later than the exposure group and did not experience any kind of brain injury in the 10+Δ years before the concussion event (note that the design allows individuals to both be part of the exposure and control group). The model is built on the assumption that the exact timing of a concussion is random for small enough values of Δ, and on the additional assumption that the exposure and the control groups would have displayed parallel trends in salary if the control group had not suffered a concussion at t_c (i.e., assuming that control and exposure group(s) would have continued to show similar trends in salaried earnings had the exposure group not experienced concussions). Table 1 show the number of patients in the exposure group and the five control groups for each year relative to exposure group's concussion incident. Using multiple comparison groups makes it possible to gage the validity of the assumption that the exact timing of a concussion is random for small enough sizes of Δ

To estimate the impact of concussion on labour market outcomes, the analysis focuses on the change in annual salary as the primary outcome, and, in further exploratory analyses, studies additional outcomes such as income from health-related benefits, income from welfare benefits, and employment rates. The data are nested within a three-level structure: Exposure or control group *g*, which includes individuals *I*, at times to exposure-groups concussion incident *t*. First, a standard difference in differences model for each separate control group Δ={1, 2, 3, 4, and 5} is estimated using ordinary least squares:

$$Salary_{git} = \beta_0 + \gamma Exposure_g + \theta Post_t + \delta Post \times Exposure_{git} + \boldsymbol{X_i\beta} + \sum_{Age=20}^{59+\Delta} I(Age)\eta_{Age} + \sum_{Year=1999}^{2012} I(Year)\eta_{Year} + \epsilon_{git} \qquad (1)$$

8

where $Salary_{git}$ measures annual salaried income deflated to 2015-level; $Exposure_g$ indicates whether the observation belongs to the exposure or control group; $Post_t$ captures the period after the exposure group's concussion occurred; $Post_t \times Exposure_{git}$ captures the effect concussion, measured as share of year t ≥ 0 affected by concussion (i.e., for year of incident exposure is expressed as share of year spent with post-exposure, for following years it is equal to 1); $\boldsymbol{X_i}$ is a set of covariates that includes a high school indicator and a gender dummy; ϵ_{git} is the error-term; and the two last sets of indicator variables $I(Age)$ and $I(Year)$ capture age and incident year (for control group, the year indexed against). Under an assumption of parallel trends in salaried earnings (i.e., assuming that control and exposure group(s) would have continued to show similar trends in salaried earnings had the exposure group not experienced concussions), δ then captures the annual causal effect of concussion on salary for people exposed to concussions (see Supplemental Methods for further details). For additional exploratory analyses, separate models across gender, educational level, and age, as well as across the salary distribution are also estimated (see Supplemental Methods, Supplemental Digital Content 1, for further details). The authors document and make available all code needed to reproduce the findings in the study (Supplemental Digital Content 2).

Standard Protocol Approvals, Registrations, and Patient Consents

Since the data used in the study come from de-identified administrative registers that Statistics Denmark makes available for research purposes for approved institutions, no approval from an ethics committee was needed to carry out the study. The research was carried out as part of project no. 706630 approved by Statistics Denmark.

For peer review only

Patient and Public Involvement

There was no involvement from patients or members of the public in the design, or conduct, or reporting, or dissemination plans of the research.

For peer review only

For peer review only - http://bmjopen.bmj.com/site/about/guidelines.xhtml

Results

Concussion leads to long-term loss in salaried income

Individuals who suffered a concussion (exposure group) had a lower salaried income compared to individuals who experienced their concussion 1, 2, 3, 4 and 5 years after the exposure group (control groups). Compared to patients who experienced a concussion one year after the exposure group, salaried income was €423/£380 (95% CI: -€9129/-£8208; €73/£66, p=.095; Table 2) lower, corresponding to a salary decrease of 1.5 % (95% CI: -0.3 %; 3.2 %; Figure 1). Compared to patients who experienced a concussion 5 years after the exposure group, however, salaried income in the exposure group was €1,243 (95% CI: -€1,564/-£1,406; -€922/-£829, p<.001) lower, corresponding to a salary decrease of 4.2 % (95% CI: 3.1 %; 5.3 %; Figure 1). Normalized wage progression for the control groups, who suffered a concussion 1, 2, 3, 4, and 5 years after the exposure group, showed similar trends and similar levels pre-exposure, indicating that the parallel wage trends assumption was met (Figure 2 and table S1, Figures S1 in Supplemental Digital Content 3).

We hypothesized that the salary decreases resulted from a combination of lower salary and exit from the labour market, either through short- or long-term absence/unemployment. In an exploratory analysis, we tested whether labour force exit drove the full effect of concussion on salary (Figure 3). By comparing the cumulative distribution of salary density for the exposure group with the cumulative distribution of salary density for the Δ=5 control group (Figure 3, left panel), we found that the impact of concussion on salary was significant for individuals in the lower quartile of the salary distribution (at a 95 % significance level). Specifically, below a threshold salaried income of 40,000€ (£36,000) the presumed impact of concussion on salary

For peer review only
For peer review only - http://bmjopen.bmj.com/site/about/guidelines.xhtml

increased towards the bottom of the earnings pyramid; Figure 3, right panel).

Comparing the exposure group to the control group Δ=5, which suffers a concussion five years after the exposure group, concussion was associated with a 2.6% (95% CI: 3.0 %; 2.2 %, p <.001) increase in the risk of receiving € 0 in annual salary.

Long-term loss in salaried income stems from exit from the labour market

To further examine whether exit from the labour market was caused either through short- or long-term absence/unemployment, we estimated a dynamic model using the control group Δ=5, which suffers a concussion five years after the exposure group. Sick leave benefits payments were higher in the exposure group compared to the control groups for the first two years following concussion. Sick leave benefits were no longer different from year 3 while the difference in annual salary between exposure and control groups persisted. Further, employment in the exposure group remained lower than in the control group Δ=5 and remained so for the entire post-exposure period (see table S2, Supplemental Digital Content 3 for further details). To assess whether some form of public benefits covered part of the salary loss, total income decline was compared to salary decline following concussion. Indeed, total income decline was lower than the salary decline through a five year period (see Figure S2, Supplemental Digital Content 3 for further details).

Younger patients without high school degree drove the effect of concussion on income loss

The exposure group and all control groups differed slightly in terms of average patient age, male to female ratio, and for control group Δ=5, in the frequency of individuals with at least a

For peer review only

high school degree (see Table S3, Supplemental Digital Content 3 for further details). To ensure that differences in gender, education, or age did not influence our results, we subdivided our exposure group into subgroups based on gender, education status, and age at time of concussion. We then estimated the impact of concussion on salary and employment across all values of Δ and for all subgroups (see, Figures S3-S8, Supplemental Digital Content 3 for further details). Patients between age 30-39 and those without a high school degree experienced the largest absolute and relative declines in salary.

Finally, we addressed the role of timing of concussion across different years. Given that per design our exposure group always suffered their concussion earlier than the control groups do, changing labour market conditions could moderate effects. Part of our sample suffered their concussion during or just prior to the Great Recession in 2009-2010, which arguably presented the largest shock to both the global and local economy since the Great Depression in the 1930s. In Denmark, the great recession was preceded by a series of years of economic growth, low unemployment, and increasing salaries (see Figure S8, Supplemental Digital Content 3 for salary development from 1994 to 2017). We estimated the impact of concussion on salary separately for each year from 2003-2012 and plotted the estimate against the percent of full-time unemployment in the Danish labour force (Figure 4). Suffering a concussion during an economic boom had a substantially higher impact on salary than doing so during a recession when comparing to control groups who suffered concussions two to five years later than exposure group.

For peer review only

Discussion

The impact of concussion on employment and salary remains understudied. In a systematic review of four studies on the association between mild TBI and return to work, Cancelliere and colleagues[9] found that most workers return to work within 3-6 months of suffering a mild TBI, but that the long-term impact (more than one year after concussion) was not studied. In addition, studies included small to medium sample sizes, varied measures of return to work, and employed both case-control and cohort designs. Using an inception cohort study design, Theadom and colleagues[7] collected follow up data four years after mild TBI incidents on 245 New Zealanders who were employed prior to incident. They found a 3.6 percent productivity decline among those who suffered a concussion, compared to a 2.3 population average decline. The group suffering mild TBI also reported more difficulties carrying out work-related tasks. In a related study,[7] Theadom and colleagues further found that the mild TBI group had persisting cognitive symptoms four years after suffering their concussion compared to an age-sex matched control group. Also using a case-control design and data from Taiwan, Chu and colleagues[6] found that one month after incident, 26 percent of patients had still not managed to return to work, and a large share of those who did return scored below full-time employment on a work quality index. Only one other study by Graff and colleagues[10] include a large patient cohort (n=19,732). Using case-control they found an association between concussion and failing to return to work of 1.54 odds ratio, but also found that exposed individuals had lower labour market attachment and was more likely to receive health related benefits pre-incident compared to the control group.

In the present study, we overcame some of the obstacles faced by previous work on the impact

For peer review only - http://bmjopen.bmj.com/site/about/guidelines.xhtml

of concussions on labour market outcomes by including a vast cohort of patients and exploiting a quasi-experimental design that allow us to plausibly account for unobserved difference between exposure and control group. In such a quasi-experimental setup exposure and control groups only differ in the timing of concussion. Since everyone in the control group experiences a concussion within five years after individuals in the exposure group, the groups are likely to be balanced on unobservable characteristics. This is particularly important given the number of potential factors that can influence employment after concussion[16,17]. Data from Donker-Cools et al.[17], for instance, suggests larger employers are more able to keep those who have sustained brain injuries in work compared to smaller employers. However, studying the effect of concussion on salaried income for individuals with employer of different size lies beyond the granularity of our data.

In addition, salary and employment data reported here were compiled routinely through third-party reporting and were mandatory for all subjects, thus giving a complete and comprehensive picture of the economic impact of concussion on a nationwide scale. It should be mentioned that our study also included data from individuals diagnosed in private hospitals. However, given the setup of the Danish health care system, i.e. private hospitals predominantly do selective and overflow surgery and have no ERs[18], only 13 patients were diagnosed at a private hospital from 2003 onwards.

Altogether, we showed that Danes between 20-59 year of age, who suffered a concussion during the period 2003-2012 experienced average salary losses of 4.2%. The impact of concussions on salary already materialized one year after the incident and remained sizeable for at least five years. This result is in line with a "burn-in" period in which the impact of

For peer review only - http://bmjopen.bmj.com/site/about/guidelines.xhtml

concussion on wages fully develops. First, concussions occur at some point during the year, thereby not affecting already earned salary that year. Second, in Denmark, most employees are entitled to receive their salary during sickness absence for an open ended, but not indefinite period. The results further showed that both in absolute and relative terms, people with an educational level at less than a high school degree saw substantially larger negative impact to salaried earnings than did those with at least a high school degree. Also, the group with less than a high school degree also saw an immediate impact on salary from their concussion (cf. Figure S4), indicating that the burn-in period present for workers with at least high school education likely expressed differences in types of employment and job protection.

In addition, total income decline was lower than the salary decline through a five year period (see Figure S2, Supplemental Digital Content 3 for further details), suggesting that the impact of concussions on salary largely stems from affected individuals leaving the labour force completely, likely sustaining themselves through early retirement, disability pensions, self-sufficiency, or other income sources instead.

It is important to mention that our study was restricted to individuals diagnosed in ER and hospital settings. Rowson et al., however, show that in concussed individuals, severity of the cranial injury is not strongly correlated with strength or length of subsequent symptoms[19]. Thus, individuals diagnosed by a GP might suffer concussion effects as much as individuals who initially sustained a more severe cranial injury and sought medical attention in an ER or hospital setting. If this holds true, our results may have validity beyond individuals diagnosed in an ER or hospital setting.

For peer review only - http://bmjopen.bmj.com/site/about/guidelines.xhtml

If we assume that people return to their expected salary levels after a five-year recovery period (a very conservative assumption that is not supported by our data), the mere net annual salary loss in our sample would be approximately €23,000,000 (£21,000,000) measured in 2015-value. That would neither include hospital charges, medical costs for the treatment of concussion, the foregone tax from income, and the increased need for welfare spending, nor would it account for the large group of individuals who never seeks treatment[20] or receive their diagnosis from their general practitioner rather than in a hospital or emergency room, and thus escape our study. Thus, total public costs are likely substantially higher.

In addition, both in absolute and relative terms, the early peak-working aged individuals (30-39 y) and the less-educated individuals in our cohort seemed to be most affected after suffering a concussion. These findings might have and additional and yet unmeasured social impact, especially if our results are transferrable to other nations with a less established welfare state and a less flexible labour market. In such countries, the impact on the young and less-educated individuals suffering a concussion and thus on society might be accentuated.

Comparing our hospital incidence rates to more complete canvases of incidences carried out by Cassidy et al.[21], it seems likely that the actual cost in the population is more than twice as large as what we estimate, assuming that individuals not diagnosed in a hospital setting on average suffer the same extent of concussion symptoms. If we were to consider the average concussion incidence rates for six other advanced European countries that are somewhat comparable to Denmark (Norway, Finland, Germany, Netherlands, England and France) and under the assumption that concussion have a similar impact on earnings in these countries, the net annual salary loss would be approximately €1,099,400,000 (£988,4780,000) measured in

For peer review only - http://bmjopen.bmj.com/site/about/guidelines.xhtml

2015-value. While our study likely underestimates the total socioeconomic impact of concussion, it suggests that concussions has a large economic impact on a nationwide scale and on productivity and income at the patient level.

Conclusion

Using timing of concussion as a natural experiment, we provide first plausible causal estimates of the effect of concussion on salary and employment among patients treated for concussion in an emergency room or hospital setting in Denmark, 2003-2017. Our results show that among this patient group concussion has a large and long-term negative causal impact on salary and employment. People between 30-39 y and those without high school degrees suffered the largest salary decreases.

For peer review only

For peer review only - http://bmjopen.bmj.com/site/about/guidelines.xhtml

Disclosures:

The authors report no conflict of interest.

Acknowledgements:

NA

Funding:

This work was supported by the Swedish Research Council for Health (PF), Working Life and Welfare (Grant no. 2016-07099) with additional funding from the ROCKWOOL Foundation (PF). The research was carried out independently of the funders.

Authors contributions: P.F. and B.C. conceived of the presented idea, P.F. performed the computations. P.F. and B.C. verified the statistical methods. P.F. and B.C. discussed the results and wrote the manuscript. The corresponding author confirms that he had full access to all the data in the study and had final responsibility for the decision to submit for publication.

Data Availability Statement

The data used in this study has been made available through a trusted third party, Statistics Denmark. Due to privacy concerns the data cannot be made available outside the hosted research servers at Statistics Denmark. University-based and private Danish scientific organizations can be authorized to work with data within Statistics Denmark. Such organization can provide access to individual scientists inside and outside of Denmark. Requests for data

For peer review only

For peer review only - http://bmjopen.bmj.com/site/about/guidelines.xhtml

may be sent to Statistics Denmark: http://www.dst.dk/en/OmDS/organisation/TelefonbogOrg.aspx?kontor=13&tlfbogsort=sektion or the Danish Data Protection Agency: https://www.datatilsynet.dk/english/the-danish-data-protection-agency/contact/. The authors document and make available all code needed to reproduce the findings in the study.

For peer review only

For peer review only - http://bmjopen.bmj.com/site/about/guidelines.xhtml

References

1 Cassidy JD, Carroll LJ, Peloso PM, *et al.* Incidence, risk factors and prevention of mild traumatic brain injury: results of the WHO Collaborating Centre Task Force on Mild Traumatic Brain Injury. *J Rehabil Med* 2004;:28–60. doi:10.1080/16501960410023732

2 Pinner M, Jensen R, Birket-Smith M, *et al.* Konsensusrapport om commotio cerebri (hjernerystelse). Videnscenter for Hjerneskade 2003.

3 Hou R, Moss-Morris R, Peveler R, *et al.* When a minor head injury results in enduring symptoms: a prospective investigation of risk factors for postconcussional syndrome after mild traumatic brain injury. *J Neurol Neurosurg Psychiatry* 2012;**83**:217–23. doi:10.1136/jnnp-2011-300767

4 Ruff R. Two decades of advances in understanding of mild traumatic brain injury. *J Head Trauma Rehabil* 2005;**20**:5–18. doi:10.1097/00001199-200501000-00003

5 Silverberg ND, Iverson GL. Is rest after concussion "the best medicine?": recommendations for activity resumption following concussion in athletes, civilians, and military service members. *The Journal of head trauma rehabilitation* 2013;**28**:250–9. doi:10.1097/HTR.0b013e31825ad658

6 Chu SY, Tsai YH, Xiao SH, *et al.* Quality of return to work in patients with mild traumatic brain injury: a prospective investigation of associations among post-concussion symptoms, neuropsychological functions, working status and stability. *Brain Injury* 2017;**31**:1674–82. doi:10.1080/02699052.2017.1332783

7 Theadom A, Barker-Collo S, Jones K, *et al.* Work Limitations 4 Years After Mild Traumatic Brain Injury: A Cohort Study. *Archives of Physical Medicine and Rehabilitation*

For peer review only

For peer review only - http://bmjopen.bmj.com/site/about/guidelines.xhtml

2017;**98**:1560–6. doi:10.1016/j.apmr.2017.01.010

8 Theadom A, Starkey N, Barker-Collo S, *et al.* Population-based cohort study of the impacts of mild traumatic brain injury in adults four years post-injury. *PLOS ONE* 2018;**13**:e0191655. doi:10.1371/journal.pone.0191655

9 Cancelliere C, Kristman VL, Cassidy JD, *et al.* Systematic Review of Return to Work After Mild Traumatic Brain Injury: Results of the International Collaboration on Mild Traumatic Brain Injury Prognosis. *Archives of Physical Medicine and Rehabilitation* 2014;**95**:S201–9. doi:10.1016/j.apmr.2013.10.010

10 Graff HJ, Siersma V, Møller A, *et al.* Labour market attachment after mild traumatic brain injury: nationwide cohort study with 5-year register follow-up in Denmark. *BMJ open* 2019;**9**:e026104. doi:10.1136/bmjopen-2018-026104

11 Ogilvie D, Adams J, Bauman A, *et al.* Using natural experimental studies to guide public health action: turning the evidence-based medicine paradigm on its head. *J Epidemiol Community Health* Published Online First: 19 November 2019. doi:10.1136/jech-2019-213085

12 Dobkin C, Finkelstein A, Kluender R, *et al.* The Economic Consequences of Hospital Admissions. *American Economic Review* 2018;**108**:308–52. doi:10.1257/aer.20161038

13 Fadlon I, Nielsen TH. Household Responses to Severe Health Shocks and the Design of Social Insurance. Cambridge, MA: 2015. doi:10.3386/w21352

14 Schmidt M, Schmidt SAJ, Sandegaard JL, *et al.* The Danish National Patient Registry: a review of content, data quality, and research potential. *Clinical epidemiology* 2015;**7**:449–90. doi:10.2147/CLEP.S91125

For peer review only

15 Statistics Denmark. Documentation of statistics: Income Statistics - Statistics Denmark. 2015.https://www.dst.dk/en/Statistik/dokumentation/documentationofstatistics/income-statistics (accessed 20 Aug 2018).

16 Buck PW, Laster RG, Sagrati JS, *et al.* Working with mild traumatic brain injury: voices from the field. *Rehabil Res Pract* 2012;**2012**:625621. doi:10.1155/2012/625621

17 Donker-Cools BHPM, Wind H, Frings-Dresen MHW. Prognostic factors of return to work after traumatic or non-traumatic acquired brain injury. *Disabil Rehabil* 2016;**38**:733–41. doi:10.3109/09638288.2015.1061608

18 Vrangbaek K, Østergren K, Birk HO, *et al.* Patient reactions to hospital choice in Norway, Denmark, and Sweden. *Health Econ Policy Law* 2007;**2**:125–52. doi:10.1017/S174413310700401X

19 Rowson S, Duma SM, Stemper BD, *et al.* Correlation of Concussion Symptom Profile with Head Impact Biomechanics: A Case for Individual-Specific Injury Tolerance. *J Neurotrauma* 2018;**35**:681–90. doi:10.1089/neu.2017.5169

20 Setnik L, Bazarian JJ. The characteristics of patients who do not seek medical treatment for traumatic brain injury. *Brain Injury* 2007;**21**:1–9. doi:10.1080/02699050601111419

21 Cassidy JD, Carroll LJ, Peloso PM, *et al.* Incidence, risk factors and prevention of mild traumatic brain injury: Results of the WHO Collaborating Centre Task Force on mild traumatic brain injury. *Journal of Rehabilitation Medicine* 2004;**36**:28–60. doi:10.1080/16501960410023732

For peer review only

For peer review only

Figure Legends

Figure 1. Estimated effect of concussions in percentage on salary for the exposure group measured against each control group

Note: Figure shows the percentage change in salary experienced by the exposure group following their concussions compared to the expected trajectory absent the concussion (calculated from the control groups) with 95 % confidence intervals. See table 1 for separate p-values for each estimate.

Figure 2. Salary development for exposure and control groups across time of exposure

Note: Figure shows the salary trajectories for the exposure group (black) who suffers concussion at year zero against normalized wage trajectories for the control groups who suffer their concussions one to five years later. Δ indicates the number of years between exposure and control incident. Table shows that there are no significant differences in the normalized salary levels for exposure and control group prior to exposure incident (see Figure S1, Supplemental Digital Content 3 for unnormalized salary trajectories).

Figure 3. (Left panel) The cumulative density function (cdf) for salary post-treatment among the treatment group and their counterfactual outcome had they not experienced their concussions, and (Right panel) the change in salary density for the exposure group compared to their counterfactual baseline expressed as the effect of concussion on the probability of earning below the salary-level expressed on the x-axis following exposure

For peer review only - http://bmjopen.bmj.com/site/about/guidelines.xhtml

event.

Note: The figure shows the observed cumulative salary distribution following concussion for the exposure group (red) and the expected counterfactual salary distribution absent suffering concussion in the exposure group (blue), when using the Δ=5 control group. The black line shows the difference between the observed and the counterfactual distribution, and the grey dash lines show the 95 % confidence interval. The close to constant decline of the difference between the two distributions as the salary increase indicates that the main part of the effect of concussions on salary are driven by people having a salary equal to zero.

Figure 4. Effect of concussion on salary across incident years and control groups together with the percentage fulltime unemployed of the labor force.

Note: Figure shows annual estimates of concussion against each control group separately mapped against the share of the labor force that is full time unemployed. 95 % confidence intervals. The estimates for the effect of concussion on salary almost uniformly increase in absolute magnitude when unemployment decreases, and decrease when unemployment increase, indicating that the effect of concussion on salary is countercyclical to the economic cycle.

Supplemental Digital Content titles & legends

Supplemental Digital Content [#1]. Text file. Supplemental materials and methods. This file contains further details on our quasi-experimental, difference-in-differences event time approach.

For peer review only

Supplemental Digital Content [#2]. File. Code used for the analyses.

Supplemental Digital Content [#3]. Table. Supplemental results Table S1: Test of parallel trends assumption pre-exposure incident against each control group separately using eq. S3 in supplementary methods. Separate exposure dummies for all time periods (except the year prior to exposure, which serves as reference period).

Supplemental Digital Content [#3]. Table. Supplemental results Table S2: Effect of concussion on different labor market outcome parameters using separate exposure dummies for all time periods (except the year prior to exposure, which serves as reference period): In this exploratory analysis, the exposure group is compared to the control group Δ=5, which suffers a concussion five years after the exposure group. Outcomes include annual salaried income (annual salary), total annual income (total income), annual sick leave benefits received (sick leave benefits) as well as a binary indicator of employment (probability of employment). Monetary outcomes were measured at 2015-level in € 1,000.

Supplemental Digital Content [#3]. Table. Supplemental results Table S3: Demographic factors for exposure group and control groups (Δ=1, 2, 3, 4, 5) averaged over the 5 years leading up to the concussion event in each of the groups. Factors include patient age (in years), share of sample female (1=100% female), and share of individuals with at least a high school degree (1=100%).

For peer review only

For peer review only - http://bmjopen.bmj.com/site/about/guidelines.xhtml

Supplemental Digital Content [#3]. Figure. Supplemental results Figure S1: Unnormalized Average Salary for Treatment and Control Groups Measured in 1K € at 2015-levels

Supplemental Digital Content [#3]. Figure. Supplemental results Figure S2: The Cumulative Density Function (CDF) for Total Income Post-Treatment among the Treatment Group and Their Counterfactual, and the Difference between the Two CDFs Expressed as the Effect of Concussion on the Probability of Total Income Below that Income-Level following Exposure Event.

Supplemental Digital Content [#3]. Figure. Supplemental results Figure S3: Percentage Effect of Concussion on Relative Salary Across Age Groups.

Supplemental Digital Content [#3]. Figure. Supplemental results Figure S4: Percentage Effect of Concussion on Relative Salary Across High School Completion.

Supplemental Digital Content [#3]. Figure. Supplemental results Figure S5: Percentage Effect of Concussion on Relative Salary Across Gender.

Supplemental Digital Content [#3]. Figure. Supplemental results Figure S6: Effect of Concussion on Absolute Salary in 1K Euro Across Age groups.

For peer review only

Supplemental Digital Content [#3]. Figure. Supplemental results Figure S7: Effect of Concussion on Absolute Salary in 1K Euro Across Education.

Supplemental Digital Content [#3]. Figure. Supplemental results Figure S8: Effect of Concussion on Absolute Salary in 1K Euro Across Gender.

Table 1. Number of observations for exposure and control groups across time since exposure and number of observations with missing salary information

Years until exposure	Exposure group	Control Δ=1	Control Δ=2	Control Δ=3	Control Δ=4	Control Δ=5
-4	36,804	33,681	31,112	29,190	27,859	26,794
-3	36,978	33,834	31,245	29,366	27,973	26,907
-2	37,195	34,003	31,407	29,501	28,146	27,031
-1	37,449	34,224	31,582	29,687	28,288	27,220
0	37,848	34,551	31,851	29,922	28,530	27,421
1	37,467		31,755	29,832	28,433	27,337
2	36,940			29,807	28,421	27,295
3	36,484				28,421	27,304
4	36,084					27,314
Total observations	333,249	170,293	188,952	207,305	226,071	244,623
Observations with missing salary	81	32	31	44	35	29

Control groups have not suffered a concussion in 10+Δ years before incident, exposure group has not suffered concussion the 10 years before exposure incident.

For peer review only

For peer review only - http://bmjopen.bmj.com/site/about/guidelines.xhtml

542 **Table 2. Estimated effect of concussion on salary of exposure group compared to**
543 **control groups that suffered their concussion Δ = 1, 2, 3, 4, 5 y after the exposure**
544 **group's concussion event, measured at 2015-level. $N_{Exposure}$: 37,848**

545

	Estimated salary effect (δ)	95 % CI	*p*	$N_{Control}$
Δ = 1 y	-423€	(-919€;73€)	.095	34,551
Δ = 2 y	-825€	(-1,108€; -543€)	<.001	31,851
Δ = 3 y	-1,019€	(-1,331€; -707€)	<.001	29,922
Δ = 4 y	-1,126€	(-1,446€; -805€)	<.001	28,530
Δ = 5 y	-1,243€	(-1,564€; -922€)	<.001	27,421

546

547 Results obtained from estimations following Eq. (1). Models include controls for high school
548 diploma, gender, age, and observation year. Results obtained using reghdfe in Stata.

549

For peer review only

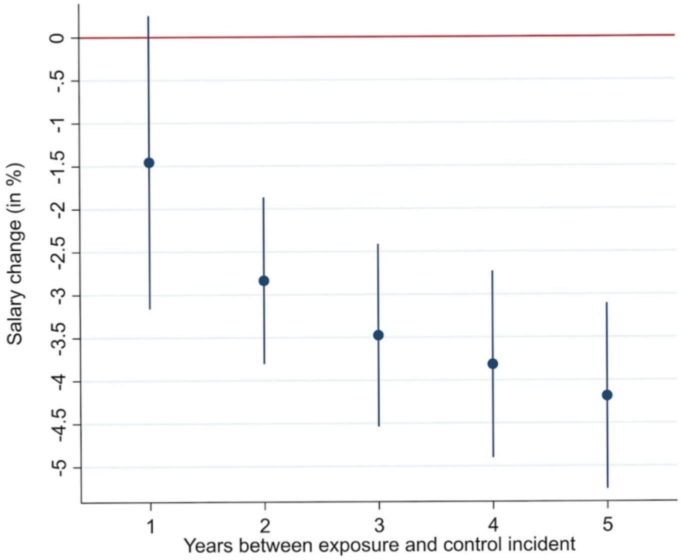

Figure 1

338x190mm (108 x 108 DPI)

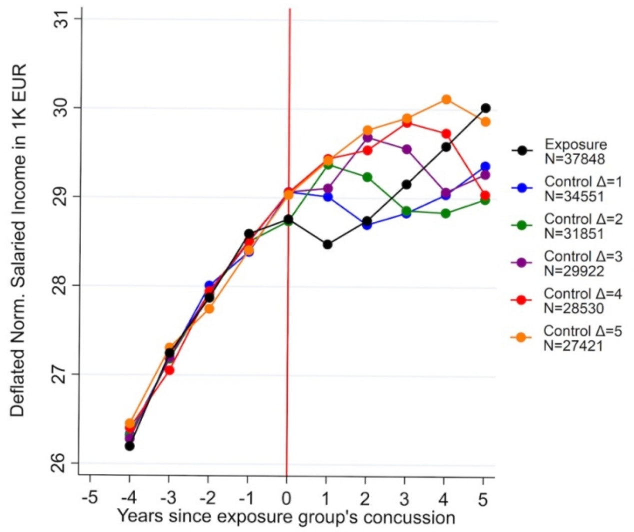

Figure 2

338x190mm (108 x 108 DPI)

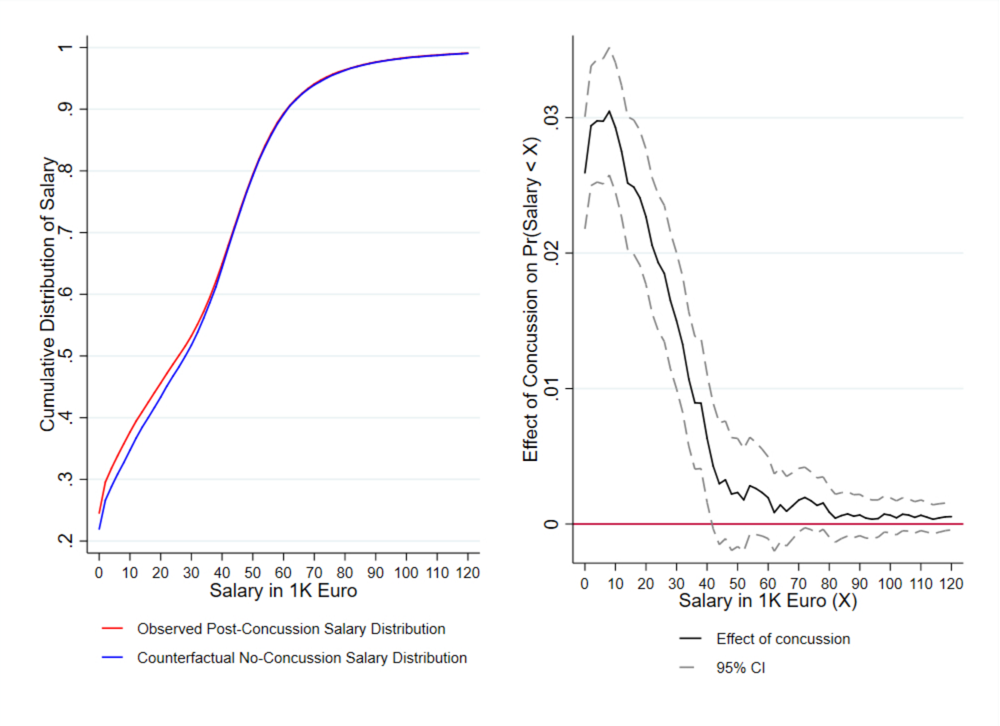

Figure 3

352x256mm (72 x 72 DPI)

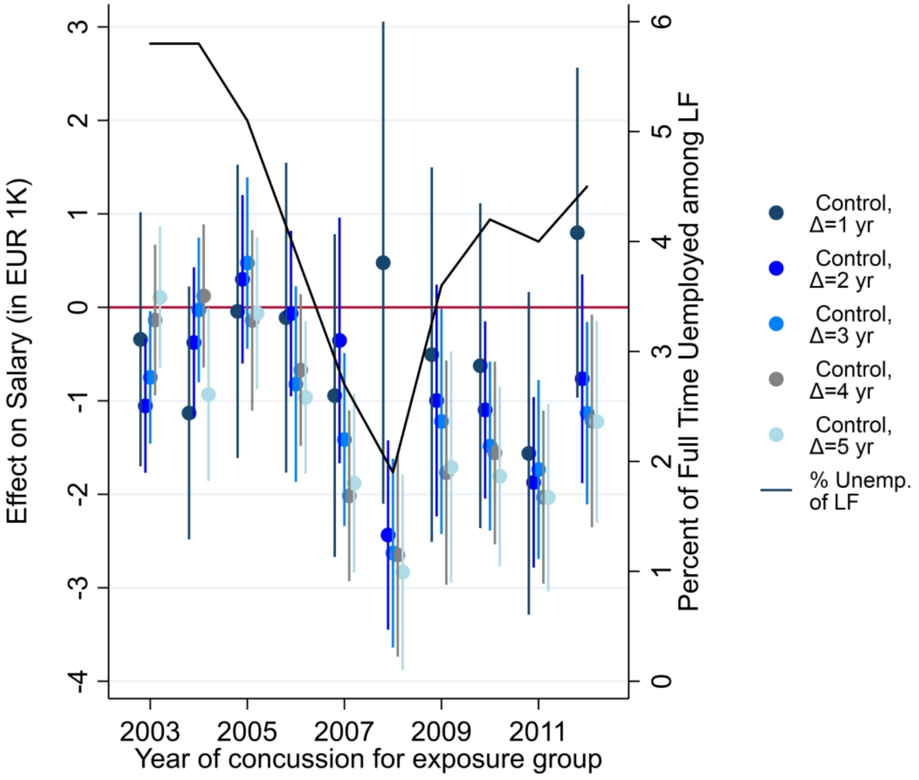
Effect on Salary (in EUR 1K)
Percent of Full Time Uemployed among LF
Year of concussion for exposure group
3
2
1
0
-1
-2
-3
-4
6
5
4
3
2
1
0
2003
2005
2007
2009
2011
Control, Δ=1 yr
Control, Δ=2 yr
Control, Δ=3 yr
Control, Δ=4 yr
Control, Δ=5 yr
% Unemp. of LF

Figure 4

SUPPLEMENTAL MATERIALS AND METHODS

Our quasi-experimental, difference-in-differences event time approach compares two groups of individuals from the same cohort, where both groups experience concussions, but at two different time points (t_c,$t_c+\Delta$). For the simple situation where we have three periods (t=0,1,2) and the exposure group (T) experiences their concussion at the start of period 1 (t_c=1), and the control group (C) at the start of period 2 ($t_c+\Delta=2$), the effect of concussion on salary (Y) is:

$$\Delta = \left(Y_1^T - Y_1^C\right) - \left(Y_0^T - Y_0^C\right)$$

The effect of concussion on salary in t=1 is estimated by comparing the average difference in salary between exposure and control groups for the post-concussion period t=1 $\left(Y_1^T - Y_1^C\right)$ to the average difference in salary for the pre-concussion, or baseline, interval t=0 $(Y_0^T - Y_0^C)$. Assuming the exact timing of a concussion is random for small enough sizes of Δ, and under the additional assumption that the exposure group would have had parallel trends in salary as the control group absent suffering concussion at t_c, δ captures the causal effect of concussion among those who suffer concussions – also known as the average effect on the treated (AT). The AT does not capture how concussions would affect a random person. The AT captures how concussions causally affect those who suffer concussions.

For our study, the parallel trends assumption states that exposure and control groups have parallel developments in salary leading up to the exposure group's concussion and the exposure and control groups would have further exhibited parallel salary trajectories if the concussion had not occurred. To test the parallel trends assumption, we estimate a dynamic version of the model specification (shown in supplementary table S1), which explicitly allows us to test whether the parallel trend assumption for our sample is probable.

To validate that the timing of concussion is random with our study period, we present estimates for effect of exposure across different periods between exposure and control incident (Δ). Most recorded concussions outside contact sports and military engagements stem from unforeseen events, such as falls or striking/being struck by an object[25,26], so assuming random timing is likely valid. People who regular engage in activities that result in high risk of multiple concussions may be different than the average concussion patient and would be more likely to end up in the exposure sample than in the control sample, which could induce bias. To avoid such potential bias, we restrict our sample to individuals without prior diagnoses for intracranial injuries ten years prior to exposure.

At t=-1, i.e. one year before the exposure group suffered a concussion, the control groups were slightly smaller than the exposure group, and two control groups (Δ=4 and 5) differed slightly but significantly in terms of average patient age (p <. 001; supplementary table S2), male to female ratio (p <. 001), and for control group Δ=5, in the frequency of individuals with at least a high school degree (p < .001). However, the differences are numerically small. To test that composition differences between exposure and control do not drive our results, we provide separate results for individuals with and without high school degree, for males and females, and for different age groups across all different values of Δ.

Further, our design inherently leads to the possibility of timing issues—our exposure group always suffers their concussion earlier (in terms of calendar time and age) than the control groups do. If the labor market is constantly improving or worsening during the period we consider, this could substantially influence our results. Therefore, we also estimate separate models across exposure incident year and control group. Estimating separate models allow us the added benefit of being able to examine whether the business cycle influences the effect of concussions on salary.

Statistical model

To estimate the impact of concussion on salary, we define the following variables: Exposure or control group *g*, which includes individuals *i*, at times to exposure-groups concussion incident *t*. First, we estimate a standard difference in differences model for each separate control group Δ={1, 2, 3, 4, and 5} using ordinary least squares:

$$Salary_{git} = \beta_0 + \gamma exposure_g + \theta post_t + \delta post \times exposure_{git} + \boldsymbol{X_i \beta} + \sum_{Age=26}^{48+\Delta} I(Age)\eta_{age} + \sum_{year=1999}^{2012} I(year)\,\eta_{year} + \epsilon_{git} \qquad \text{(S1)}$$

where $Salary_{git}$ measures annual salaried income deflated to 2015-level, $exposure_g$ indicates whether the observation belongs to the exposure or control group, $post_t$ captures the period after the exposure group's concussion occurred, and $post_t \times exposure_{git}$ captures the effect concussion, measured as share of year t ≥ 0 affected by concussion. In this way, someone who suffers a concussion July 1 has $post_t \times exposure_{git} = 0.5$ for $t = 0$ and $post_t \times exposure_{git} = 1$ for $t > 0$. $\boldsymbol{X_i}$ is a set of covariates that includes a high school indicator and a gender dummy, ϵ_{git} is the error-term, and the two last sets of indicator variables $I(Age)$ and $I(Year)$ capture age and incident year levels (control group indexed against incident year). Under the parallel trends assumption, δ then captures the annual effect of concussion on salary. In eq. 1, $exposure_g$ normalizes any pre-exposure differences between the exposure and control group, thereby creating a joint baseline pre-exposure.

For peer review only - http://bmjopen.bmj.com/site/about/guidelines.xhtml

We estimate robust individual-level clustered standard errors to account for the possibility that individuals enter the data twice both as control (0) and exposure (1) individuals (g={0,1}), and that they are observed for multiple periods (t={-4,…, Δ-1}). To calculate the relative salary decrease after concussion, we exploit the parallel trends assumption to generate the expected counterfactual salary level, i.e. had the concussion not occurred, and calculate the decline expressed in percentage as: $\%\,\text{change} = \delta \,/\, E(\widehat{Salary}_{git} | g = 1, post_t = 1, post_t \times exposure_{git} = 0)$. In this way, we provide both absolute estimates measured in 1K Euro, as well as percentage change.

We expect δ from eq. (1) to likely be negative. Yet, a decrease in annual salary can arrive through two different channels. Concussions may affect salary through either decreasing income among those employed or by reducing the number of individuals who are employed and earning any salary at. To parse out which of the two channels is driving the results, we examine how concussion affects the salary distribution among the exposure group following. Following Chernozhukov et al.[27] we estimate a series of regressions across the whole salary distribution, where, for a finite set of points, we predict how concussion affects the likelihood of having earnings on the left side of each finite point, as follows:

$$\sum_{j=0}^{\max(Salary)} p_j = \beta_{0j} + \delta_j post_t \times exposure_{git} + \theta post_t + \gamma_j exposure_g + \boldsymbol{X_i \beta} + \sum_{Age=26}^{48+\Delta} I(Age)\eta_{age,j} + \sum_{year=1999}^{2012} I(year)\,\eta_{year,j} + \epsilon_{git,j} \quad \text{(S2)}$$

where $p_j = \Pr(Salary_{git} \leq j)$ and j is the interval from 0 to $max(Salary)$. Across the salary distribution, we can now predict the probability of earning less than j for those with and without concussions. From equation 2, we predict $p_j^1 = E(p_j | post_t \times exposure_{git} = 1, exposure_g = 1, t \geq 0)$ and the counterfactual $p_j^0 = E(p_j | post_t \times exposure_{git} = 0, exposure_g = 1, t \geq 0)$. Plotting p_j^1 and p_j^0 over each value of salary j, and assuming rank stability, gives the cumulative density function of salary for the treated (p_j^1) and the counterfactual observation of the treated had they not suffered concussions (p_j^0). The difference between p_j^1 and p_j^0 is simply δ_j. If the value of δ_j monotonically moves towards zero as j increases until $p_j^1 \approx p_j^0 \approx 1$ it indicates that exit from employment fully drives the effect of concussion on salary. If instead the value of δ_j is constant or increasing across parts of the distribution, it instead indicates that a decrease in salary among those still receiving salary drives at least part of the effect.

Eq. 1 and eq. 2 are based on the parallel trends assumption. The assumption states that exposure and control groups follow parallel salary trajectories until individuals in the exposure group experiences a concussion, and that the parallel trends would have continued had the concussion not occurred. Whereas we cannot verify the counterfactual situation of parallel trends after exposure, we can use a dynamic model to test for systematic differences in salary trends between exposure and control group in the years leading up to the exposure group's concussion event. To do so, we estimate the following dynamic model:

$$Salary_{git} = \beta_0 + \sum_{t\neq -1, t=-4}^{\Delta-1} \delta_t \times I(t_g) \times exposure_g + \sum_{t=-4}^{\Delta-1} I(t_g)\eta_t + \gamma exposure_g + \boldsymbol{X_i \beta} + \sum_{Age=26}^{48+\Delta} I(Age)\eta_{age} + \sum_{year=1999}^{2012} I(year)\,\eta_{year} + \epsilon_{git} \quad \text{(S3)}$$

Where we interact exposure group status (*exposure*$_g$) with indicators $I(t_g)$ capturing time from concussion. If the parallel trends assumption holds, then it must be the case {δ_{-4}, δ_{-3}, δ_{-2}}=0, whereas the size and sign of {$\delta_0 \ldots \delta_{\Delta-1}$} captures the dynamic effect of a concussion from the year of incidence and Δ-1 years onward. By estimating the effect of concussion on salary among different years of the study period, we are also able to capture how the impact of concussion on salary evolves year to year after the concussion has occurred. We further estimate eq. 3 for a series of related labor market outcomes (annual total income, annual amount of sickness benefits received, annual probability of being employed), to generate a more thorough understanding on how concussions affect labor market outcomes—i.e., if people experience a decrease in salary due to a concussion, are they then compensated through different types of welfare state services.

25 Faul M, Coronado V. Epidemiology of traumatic brain injury. *Handbook of clinical neurology* **127**:3-13, 2015

26 Taylor CA, Bell JM, Breiding MJ, Xu L. Traumatic Brain Injury–Related Emergency Department Visits, Hospitalizations, and Deaths — United States, 2007 and 2013. *MMWR Surveillance Summaries* **66**:1-16, 2017

27 Chernozhukov V, Fernández-Val I, Melly B. Inference on Counterfactual Distributions. *Econometrica* **81**:2205-2268, 2013

```
********************************************************************
*************************************************
********************************************************************
*************************************************
********************************************************************
*************************************************
*****
***** This study relies on restricted individual level
administrative data obtained
***** from Statistics Denmark.
*****
***** Only Danish research environments are granted authorization to
access data from Statistics Denmark. ***** Foreign
***** researchers can, however, get access to micro data through an
affiliation to a Danish authorized
***** environment.
***** Access is given to anonymized micro data, i.e. data at an
individual personal or corporate level. ***** Access takes
***** place through researcherís own pc over the Internet.
*****
***** See https://www.dst.dk/en/TilSalg/Forskningsservice for detail
*****
***** For the replication of present study, contact the ROCKWOOL
Foundation for access.
***** http://www.rockwoolfonden.dk/en/
*****
********************************************************************
*************************************************
********************************************************************
*************************************************
********************************************************************
*************************************************


********************************************************************
*************************************************
********************************************************************
*************************************************
********************************************************************
*************************************************
*****
***** This is the master data do-file for the full package of do-
files that generates the results for
***** Fallesen and Campos (2020). Execute files in the order they
are listed. For each file, specify a ***** home registry in place of
[home]. Further, generate subfolders [home]/data, [home]/tables, and
***** [home]/highdef to capture auxiliary data sets, tables, and
figures.
*****
********************************************************************
*************************************************
********************************************************************
*************************************************
```

```
***********************************************************************
**************************************************

***Install user-written reghdfe command for faster computation of
regressions
ssc install reghdfe

***Generate concussion samples and merges on covariates and outcome
do 01generate_sample.do

***Generates auxilary data sets the includes social benefit
recipiency indicator and levels of benefits received
do 02generate_benefits.do

***Generates auxilary data sets the includes social benefit
recipiency indicator and levels of benefits received
do 02generate_benefits.do

***Generate results
do 03generate_figures_and_results

exit, clear
```

```
clear all

*****************************************************************
***********
**
**              This program builds data for Fallesen & Campos
(2020)
**              study of concussion's impact on productivity
measured
**              through annual salary
**
*****************************************************************
***********


**Global for path to registry data
global dorg "E:/data/rawdata/706630"

*Global for processed data
global data "[home]/data"

/*globals for price index to calculate income at 2015-level across
years*/
/*Price index obtained from www.dst.dk/en/statistik/emner/priser-og-
forbrug/forbrugeriser/nettoprisindeks */

{
global price1980 = .358
global price1981 = .398
global price1982 = .439
global price1983 = .466
global price1984 = .494
global price1985 = .517
global price1986 = .521
global price1987 = .537
global price1988 = .564
global price1989 = .594
global price1990 = .612
global price1991 = .628
global price1992 = .642
global price1993 = .651
global price1994 = .662
global price1995 = .674
global price1996 = .688
global price1997 = .703
global price1998 = .713
global price1999 = .728
global price2000 = .751
global price2001 = .769
global price2002 = .788
global price2003 = .806
global price2004 = .817
global price2005 = .833
global price2006 = .850
```

```
global price2007 = .867
global price2008 = .899
global price2009 = .917
global price2010 = .936
global price2011 = .960
global price2012 = .978
global price2013 = .986
global price2014 = .994
global price2015 =1.00
global price2016 =1.005
global price2017 =1.017
}


/*Locate concussions and other TBIs from the Danish National Patient
Registry */
/**/
forvalue t = 1977/2017{
        if `t' < 1994 use $dorg/lpr_diag`t'.dta /// **uses ICD-8
codes until Dec. 31, 1993
                if substr(c_diag,1,2)=="85"
        if `t' > 1993 use $dorg/lpr_diag`t'.dta /// **uses ICD-10
codes from Jan. 1, 1994
                if substr(c_diag,1,4)=="DS06"
        **recovers encrypted social security number and admittance
date
        merge m:m recnum using $dorg/lpr_adm`t', keepus(pnr d_ind*)
keep(3)
        drop _merge recnum


        **Keeps diagnosis, diagnosis type, and individual id (pnr)
        keep pnr c_diag  c_diagtype pnr d_ind

        **generate year variable
        gen year = year(d_ind)

        **geenrate share of year with concussion
         gen time_from_incident = 1-((d_ind-mdy(1,1,year(d_ind)))/
365)
        drop d_ind

        *save as one dataset
        if `t' >  1977 append using $data/concussion.dta
        if `t' == 2017 sort pnr year
        save $data/concussion.dta, replace


}
*/
/*******************************************
Sets up datasets for treatment group (x = 0)
and the control groups who suffer concussion
1, 2, 3, 4, 5 years later (x = 1 2 3 4 5).
```

```
Treatment group are not allowed to have suffered
any type of TBI the last 10 years before concussion
, control group are not allowed to have suffered any TBI
concussion the last 10 + x years.

******************************************/
/**/
forvalue x = 0/5{
        use $data/concussion.dta, clear
        sort pnr year

        **generate measure of length between registered TBIs
        by pnr: gen help = year-year[_n-1]

        drop if help < 10+`x'

        **Keep only concussion, and only when it was primary
diagnosis
        keep if c_diagtype== "A" & ///    Primary daignosis
                (c_diag == "85099" | ///  ICD-8 code for concussion
                substr(c_diag,1,5) == "DS060") // ICD-10 code for
concussion

        *generate treatment and control datasets
        save $data/concussion_`x'.dta, replace

}



*/
/*************************************************************
Generate datasets for analysis. First incident year is allowed
to be 1992, because it is the first year where we have full record
for the five year plus control group (1977+10+5 = 1992).

We generate seperate datasets for each incident year for treatment
group and different control groups.

*************************************************************/

forvalue time = 1/5{             //for different control groups

        local post_period = 5    // local for number of years
observed post
                                                           //
concussion for treatment group

        local endtime = 2017-`post_period'        /*last year where
we allow for treatment event
                                                           to
occur, in order to have long enough control
                                                           period.
```

```
Defined by latest year available data*/

        forvalue count = 1992(1)`endtime'{
                local t = `count'                        // for
ease of coding
                local n = `t'-4                          // first
pre-treatment event period
                local c = `t'+`post_period'      //last post-event
period
                local w = `t'+`time'             //time of
concussion for control

                use pnr alder using $dorg/bef`t'          ///
Bring in all 30-49 yr olds
                        if inrange(alder`t',20,59), clear // from
the population register

                **year variable
                gen year = `t'

                **limit sample to those who suffer a concussion in
`t'
                merge 1:1 pnr year using $data/
concussion_0.dta, ///
                        keep(3) nogen

                forvalue x=`n'/`c'{      //add longitudinal data
                        merge 1:1 pnr using $dorg/bef`x', ///
                                keep(1 3) keepus(efalle alder
koen)   //add information on spouse,

                                        //age, and gender
                        rename _merge merge`x' //indicator for
whether in DK that year

                        **Add salary information and ses
information
                        if `x' < 2017{
                                merge 1:m pnr using $dorg/
ind`x', ///     m:1 to account for duplicates
                                        nogen keep(1 3)
keepus(erhvervsindk_13 pre_socio personindk dispon_13
aekvivadisp_13)  // in data on non-important variables

                                bysort _all: keep if _n ==1
                                        //drop perfect duplicates
                                **Align variable names and account
for inflation

                                rename erhvervsindk_13 loenmv
                                rename pre_socio pre_socio`x'

                                foreach kk in personindk dispon_13
aekvivadisp_13 loenmv{
```

```
                                                rename `kk'
`kk'`x'
                                }

                                foreach kk in personindk dispon_13
aekvivadisp_13 loenmv{
                                                replace `kk'`x' =
        `kk'`x'/${price`x'}
                                }

                                **Bring in educational information
                                merge 1:1 pnr using $dorg/
uddany`x', ///
                                nogen keep(1 3) keepus(hffsp)
                        }
                        if `x' == 2017{
                                merge 1:m pnr using $dorg/
ind`x', ///      m:1 to account for duplicates
                                        nogen keep(1 3)
keepus(erhvervsindk_13 pre_socio personindk)      // in data on
non-important variables

                                bysort _all: keep if _n ==1
                                        //drop perfect duplicates
                                **Align variable names and account
for inflation

                                rename erhvervsindk_13 loenmv
                                rename pre_socio pre_socio`x'

                                foreach kk in personindk loenmv{
                                                rename `kk'
`kk'`x'
                                }

                                foreach kk in personindk loenmv{

                                                replace `kk'`x' =
        `kk'`x'/${price`x'}
                                }

                                **Bring in educational information
                                merge 1:1 pnr using $dorg/
udda`x', ///
                                        nogen keep(1 3)
keepus(hfaudd)
                                rename hfaudd hfaudd`x'
                        }
                }

                **Reshape data to panel structure
                if `count'>= 2012 reshape long efalle alder koen
loenmv pre_socio personindk dispon_13 aekvivadisp_13 hffsp merge
hfaudd , i(pnr) j(t)
```

```
                if `count' < 2012 reshape long efalle alder koen
loenmv pre_socio personindk dispon_13 aekvivadisp_13 hffsp merge  ,
i(pnr) j(t)

                gen count = t-year       //variable for time to
concussion
                gen treatment =1 //treatment group indicator

                save $data/sample_temp.dta, replace //temporary
dataset

                /
********************************************************
                Now build control sample for time `time' and year
`count'

*********************************************************/



                use pnr alder using $dorg/bef`t'          ///
Bring in all 30-49 yr olds
                        if inrange(alder`t',20,59), clear // from
the population register

                **year variable
                gen year = `w'   //time for concussion for control
group `time'

                **limit sample to those who suffer a concussion in
`t'
                merge 1:1 pnr year using $data/
concussion_`time'.dta, ///
                        keep(3) nogen

                        forvalue x=`n'/`c'{      //add
longitudinal data
                                merge 1:1 pnr using $dorg/
bef`x', ///
                                        keep(1 3) keepus(efalle
alder koen)      //add information on spouse,

                                                //age, and gender
                                rename _merge merge`x' //indicator
for whether in DK that year
                                if `x' < 2017{
                                        **Add salary and SES
information
                                        merge 1:m pnr using
$dorg/ind`x', ///        1:m to account for duplicates
                                                nogen keep(1 3)
keepus(erhvervsindk_13 pre_socio personindk dispon_13
aekvivadisp_13) // in data on non-important variables
```

```
                                        bysort _all: keep if _n
==1                                             //drop perfect
duplicates

                                        **Align variable names
and account for inflation

                                        rename erhvervsindk_13
loenmv
                                        rename pre_socio
pre_socio`x'

                                        foreach kk in personindk
dispon_13 aekvivadisp_13 loenmv{
                                                rename `kk'
`kk'`x'
                                        }

                                        foreach kk in personindk
dispon_13 aekvivadisp_13 loenmv{
                                                replace `kk'`x' =
        `kk'`x'/${price`x'}
                                        }

                                        **Bring in educational
information
                                        merge 1:1 pnr using
$dorg/uddany`x', ///
                                        nogen keep(1 3)
keepus(hffsp)
                                }
                        if `x' == 2017{
                                merge 1:m pnr using $dorg/
ind`x', ///      m:1 to account for duplicates
                                        nogen keep(1 3)
keepus(erhvervsindk_13 pre_socio personindk)      // in data on
non-important variables

                                bysort _all: keep if _n ==1
                                        //drop perfect duplicates
                                **Align variable names and account
for inflation

                                rename erhvervsindk_13 loenmv
                                rename pre_socio pre_socio`x'

                                foreach kk in personindk loenmv{
                                                rename `kk'
`kk'`x'
                                }

                                foreach kk in personindk loenmv{

                                                replace `kk'`x' =
```

```
          `kk'`x'/${price`x'}
                                }

                                **Bring in educational information
                                merge 1:1 pnr using $dorg/
udda`x', ///
                                        nogen keep(1 3)
keepus(hfaudd)
                                rename hfaudd hfaudd`x'
                        }
                }

                **Reshape data to panel structure
                if `count'>= 2012 reshape long efalle alder koen
loenmv pre_socio personindk dispon_13 aekvivadisp_13 hffsp merge
hfaudd , i(pnr) j(t)
                if `count' < 2012 reshape long efalle alder koen
loenmv pre_socio personindk dispon_13 aekvivadisp_13 hffsp merge  ,
i(pnr) j(t)
                gen count = t-`t'                //variable for
time to concussion for treatment
                gen control`time' =1     //control indicator
                save $data/control_temp, replace

                **Build sample with treatment and control `time'
for year `count'
                use $data/sample_temp
                append using $data/control_temp

                **fixes control and treatment indicators
                replace control`time' = 0 if control`time'==.
                replace treatment = 0 if treatment==.

                **Picks up changes to education variable
                if `count' >=2012{
                        tostring hfaudd, replace
                        rename hfaudd start
                        merge m:1 start using "\
\srvfsenas1\data\Formater\SAS formater i Danmarks
Statistik\STATA_datasaet\Disced\c_udd_niveau_l1l2_k.dta" , nogen
keep(1 3)
                        destring UDD, replace force
                        **Replace all with high scho0l degree or
higher in HFAUDD to have HFFSP = 40000001
                        replace hffsp = 40000001 if t == 2017 &
inrange(UDD,30,80)
                        replace hffsp = 0 if t == 2017 & !
inrange(UDD,30,80)
                        drop UDD start
                }

                sort pnr t
```

```
			save $data/sample_control_`count'_`time'.dta,
replace
		}
	}


	forvalue time = 1/5{
		forvalue count =2003/2012{
			if `time' ==1 & `count' ==2003 use $data/
sample_control_`count'_`time'.dta, clear
			else append using $data/
sample_control_`count'_`time'.dta

			if `time' ==5 & `count' ==2012 bysort pnr: keep if
_n ==1
			if `time' ==5 & `count' ==2012  count
		}
	}
```

For peer review only

```
clear

************************************************************************************
************************************************************************************
**
**              Calculate share of year on public benefits and size of benefit
**              payments for Fallesen and Campos (2020)
**
**
************************************************************************************
************************************************************************************
/*globals for price index to calculate income at 2015-level across years*/
/*Price index obtained from www.dst.dk/en/statistik/emner/priser-og-forbrug/forbrugeriser/nettoprisindeks */

global price1980 = .358
global price1981 = .398
global price1982 = .439
global price1983 = .466
global price1984 = .494
global price1985 = .517
global price1986 = .521
global price1987 = .537
global price1988 = .564
global price1989 = .594
global price1990 = .612
global price1991 = .628
global price1992 = .642
global price1993 = .651
global price1994 = .662
global price1995 = .674
global price1996 = .688
global price1997 = .703
global price1998 = .713
global price1999 = .728
global price2000 = .751
global price2001 = .769
global price2002 = .788
global price2003 = .806
global price2004 = .817
global price2005 = .833
global price2006 = .850
global price2007 = .867
global price2008 = .899
global price2009 = .917
global price2010 = .936
global price2011 = .960
global price2012 = .978
```

```
global price2013 = .986
global price2014 = .994
global price2015 =1.00
global price2016 =1.005
global price2017 =1.017


**Global for path to registry data
global dorg "e:/data/rawdata/706630"
*Global for processed data
global data "E:/data/workdata/706630/pf/FallesenCampos/data"

forvalue t=1996/2017{

        ** Read in data on social benefits recipiency share of
weeks
        ** from the DREAM database
        use $dorg/dream`t'
        gen share =0
        forvalue y = 1/52{
                        if `y' < 10 replace share = share+1 if
y_0`y' !=.
                        if `y' >  9 replace share = share+1 if
y_`y' !=.
                        if `y' < 10 drop y_0`y'
                        if `y' >  9 drop y_`y'

        }
        **Generate annual measure of share of year receiving social
benefits
        replace share = share/52
        keep pnr share
        gen t = `t'
        if `t' > 1996 append using $data/temp.dta
        save $data/temp.dta, replace

}


forvalue t=1998/2017{

        **Read in information on size of different types of social
benefits

        if `t' < 2002{
                use pnr syg_barsel_13 konthj arblhum pre_socio
using $dorg/ind`t'.dta, clear
                replace syg_barsel_13 = syg_barsel_13/${price`t'}
                replace konthj  = konthj /${price`t'}
                replace arblhum = arblhum/${price`t'}
                gen kont_dag = konthj+arblhum
                drop konthj arblhum
        }
        if `t' >= 2002 & `t' < 2013{
```

```
			use pnr syg_barsel_13 adagpagn konthj arblhum 
	pre_socio using $dorg/ind`t'.dta, clear
			replace syg_barsel_13 = syg_barsel_13/${price`t'}
			replace adagpagn = adagpagn/${price`t'}
			replace konthj  = konthj /${price`t'}
			replace arblhum = arblhum/${price`t'}
			gen kont_dag = konthj+arblhum
			drop konthj arblhum
		}
		if `t' >= 2013{
			use pnr syg_barsel_13 adagpagn dagpenge_kontant_13 
	pre_socio using $dorg/ind`t'.dta, clear
			replace syg_barsel_13 = syg_barsel_13/${price`t'}
			replace adagpagn = adagpagn/${price`t'}
			replace dagpenge_kontant_13  = 
	dagpenge_kontant_13 /${price`t'}
			gen kont_dag = dagpenge_kontant_13-syg_barsel_13
			drop dagpenge_kontant_13
		}
		gen t = `t'
		compress
		bysort pnr: keep if _n ==1
		if `t' > 1998 append using $data/temp2.dta
		if `t' == 2017{
			sort pnr t
		}
		save $data/temp2.dta, replace
	}
```

iew only

```
clear all

*********************************************************************
***********
**
**              This program geenrates figerus and results for
Fallesen & Campos (2020)
**              study of concussion's impact on productivity
measured through
**              annual salary
**
*********************************************************************
***********

**Global for path to registry data
global dorg "E:/data/rawdata/706630"

*Global for processed data
global data "[home]/data"

*Global on figures
global highdef "[home]\highdef"



forvalue time = 1/5{              //for different control groups

        local post_period = 5     // local for number of years
observed post
                                                            //
concussion for exposure group

        local endtime = 2017-`post_period'        /*last year where
we allow for exposure event
                                                            to
occur, in order to have long enough control
                                                            period.
Defined by latest year available data*/

        **Matrixes to capture estimates
        matrix results   = J(15,6,.)      // For salary estimates
        matrix results_p = J(15,6,.)      // For Pr(salary=0)
estimates
        matrix t         = J(15,6,.)      // For time indicators

        forvalue count = 2003(1)`endtime'{
                use $data/sample_control_`count'_`time', clear

                gen female = koen==2

                qui{
                        gen edu =0
                        replace edu = 1 if inrange(hffsp,
20000000,39000000) | ///
```

```
(hffsp >40000000 & hffsp!=.)
                    }

                    **exclude individuals in years where they do not
appear in data,
                    **due to either death or migration, as well as
periods from when
                    **the control group sufer their concussion
                    drop if merge ==1 | count > `time'-1

                    **generate concussion variable
                    gen treat = inrange(count,0,`time'-1) & treatment
==1
                    replace treat = time_from_incident if count ==0 &
treatment ==1

                    **Generate pre-concussion income difference
for
                    **use in calculating marginal effects
                    sum loenmv if count <0 & treatment ==0
                    local control =r(mean)
                    sum loenmv if count <0 & treatment ==1
                    local treat =r(mean)
                    sum loenmv if count>=0 & treatment ==0
                    local control_post =r(mean)
                    gen post = count >=0

                    **estimate DiD model on salary
                    reghdfe loenmv treat, abs(alder female post
treatment edu year) cl(pnr)
                    matrix b =e(b)   //regression coefficient
                    matrix V = e(V)  // standard error^2
                    local n = `count'-2002   //time

                    matrix results[`n',1]    =       b[1,1]
                    matrix results[`n',2]    =       V[1,1]^.5
                    matrix results[`n',3]    =       b[1,1]/
(`control_post'-(`control'-`treat'))
                    matrix results[`n',4]    =       `n'


          }
          svmat results

          rename results1 est
          rename results2 se
          rename results3 marg
          rename results4 time

          replace time = time+(`time'-3)*.1 //jitter estimates for
graph
```

```
        keep est* se* marg* time
        keep if est !=.

        replace est = est/7446    //estimate measured as 1000 Euro
        replace se = se/7446      //S.E. measured as 1000 Euro

        gen upper = est+se*1.96 // Upper CI
        gen lower = est-se*1.96 // Lower CI


        gen control = `time'              //indicate control group

        if `time' >1 append using $data/results.dta
        save $data/results.dta, replace
}

use $data/results.dta, clear

replace time = 2002+time


*reads in unemployment statistcis obtained from statistikbanken.dk/
en/

gen unemp = 5.8 if time ==2003
replace unemp = 5.8 if time ==2004
replace unemp = 5.1 if time ==2005
replace unemp = 3.9 if time ==2006
replace unemp = 2.7 if time ==2007
replace unemp = 1.9 if time ==2008
replace unemp = 3.6 if time ==2009
replace unemp = 4.2 if time ==2010
replace unemp = 4.0 if time ==2011
replace unemp = 4.5 if time ==2012

scatter est time if control ==1, mcolor(navy) yaxis(1) ysc(range(-4
3) axis(1)) ylab(-4(1)3)  || ///
scatter est time if control ==2, mcolor(blue) || ///
scatter est time if control ==3, mcolor(midblue) || ///
scatter est time if control ==4, mcolor(gray) || ///
scatter est time if control ==5, mcolor(ltblue) || ///
rspike upper lower time if control ==1, lcolor(navy) || ///
rspike upper lower time if control ==2, lcolor(blue) || ///
rspike upper lower time if control ==3, lcolor(midblue) || ///
rspike upper lower time if control ==4, lcolor(gray) || ///
rspike upper lower time if control ==5, lcolor(ltblue) || ///
line unemp time , lcolor(black) yaxis(2) ysc(range(0 6) axis(2))
ylab(0(1)6, axis(2))  ///
xsc(range(2002.5 2012.5)) xlab(2003(2)2012) ///
yline(0) ysize(10) xsize(12) graphr(c(white)) ///
xti("Year of concussion for exposure group") scale(.95) ///
legend(label(1 " Control," "{&Delta}=1 yr") ///
label(2 " Control," "{&Delta}=2 yr") ///
label(3 " Control," "{&Delta}=3 yr") ///
```

```
label(4 " Control," "{&Delta}=4 yr") ///
label(5 " Control," "{&Delta}=5 yr") ///
label(6 "% Unemp." "of LF") ///
c(1) order(1 2 3 4 5 6) pos(3) size(small) ///
c(1) symx(4) region(lc(white))) ///
yti("Effect on Salary (in EUR 1K)", height(7) axis(1)) ///
yti("Percent of Full Time Uemployed among LF", height(7) axis(2))


graph export $highdef/marg_est.png, replace width(3900)


forvalue control_time=1/5{
        local end = 2012 // last incident year in data
        if `control_time' ==1    eststo clear

        **build dataset for joint estimate across years
        forvalue count=2003/`end'{
                if `count'==2003{
                        use $data/
sample_control_`count'_`control_time'.dta, clear
                        gen time = `count' //incident year
indicator
                }
                else append using $data/
sample_control_`count'_`control_time'.dta
                replace time = `count' if time ==.

                **exclude individuals in years where they do not
appear in data,
                **due to either death or migration, as well as
periods from when
                **the control group sufer their concussion
                drop if merge ==1 | count > `control_time'-1
        }

        gen female = koen==2

        //build ident, so we can multivariate cluster for
individuals
        //who occur both as control and exposure during the period
(id)

        bysort pnr time: gen helpx = _n ==1
        gen id= sum(helpx)
        drop helpx

        **Generate educational groups
        qui{
                gen edu =0
                replace edu = 1 if inrange(hffsp,20000000,39000000)
| ///
                                                                  (hffsp
>40000000 & hffsp!=.)
```

```
	}

	**Calculate number of observations for exposure and control
	count if count==0 & treatment ==1
	local Ntreated = r(N)
	count if count==0 & treatment ==0
	local Ncontrol = r(N)

	**generate concussion variable
	gen treat = inrange(count,0,`control_time'-1) & treatment
==1
	replace treat = time_from_incident if count ==0 & treatment
==1

	**Generate pre-concussion income difference	for
	**use in calculating marginal effects
	sum loenmv if count <0 & treatment ==0
	local control =r(mean)
	sum loenmv if count <0 & treatment ==1
	local treat =r(mean)
	sum loenmv if count>=0 & treatment ==0
	local control_post =r(mean)

	forvalue t=-4/4{
		local n = `t'*-1
		if `t' < -1 gen T_`n' = treatment ==1 & count ==`t'
		if `t' > -1 gen T`t' = treatment ==1 & count ==`t'

	}

	**estimate DiD model on salary
	reghdfe loenmv T*, abs(alder female count time treatment
edu) cl(pnr  id)
	eststo est1_`control_time'
	if `control_time'==1 matrix results = J(5,5,.) // matrix to
capture results
	if `control_time'==1 matrix results_p = J(5,5,.) // matrix
to capture results
	if `control_time'==1 matrix results_pre = J(5,5,.) //
matrix to capture results

	matrix b = e(b)
	matrix V = e(V)
	local n = `control_time'

	matrix results[`n',1]	= b[1,1] / 7466  // capture beta
results as 1K Euro
	matrix results[`n',2]	= (V[1,1]^.5)/7466	//
capture standard error as 1K Euro
	matrix results[`n',3]	=	b[1,1]/(`control_post'-
(`control'-`treat'))
	matrix results[`n',4]	=	`n'

	gen no_lon = loenmv<1 //dummy for no salary
```

```
        **estimate DiD LP-model for pre-trends
        xi: reghdfe loenmv T*, abs(alder female count time
treatment edu) cl(pnr  id), if count <0
        eststo est3_`control_time'
        matrix b =e(b)   //regression coefficient
        matrix V = e(V)  // standard error^2

        matrix results_pre[`n',1] =       b[1,1]
        matrix results_pre[`n',2] =       V[1,1]^.5
        matrix results_pre[`n',3] =       b[1,1]/(`control_post'-
(`control'-`treat'))
        matrix results_pre[`n',4] =       `n'

}

esttab est1_* using [home]/tables/dynamic1.rtf, ///
        replace se(1) b(1) compress nogap star(+ .1 * .05 ** .01
*** .001) ///
        keep(T*)

esttab est2_* using [home]/tables/dynamic2.rtf, ///
        replace se(3) b(3) compress nogap star(+ .1 * .05 ** .01
*** .001) ///
        keep(T*)

esttab est3_* using [home]/tables/pre_trends.rtf, ///
        replace se(3) b(3) compress nogap star(+ .1 * .05 ** .01
*** .001) ///
        keep(T*)

forvalue control_time=1/5{
        local end = 2012 // last incident year in data
        if `control_time' ==1    eststo clear
        qui{
                **build dataset for joint estimate across years
                forvalue count=2003/`end'{
                        if `count'==2003{
                                use $data/
sample_control_`count'_`control_time'.dta, clear
                                gen time = `count' //incident year
indicator
                        }
                        else append using $data/
sample_control_`count'_`control_time'.dta
                        replace time = `count' if time ==.

                        **exclude individuals in years where they
do not appear in data,
                        **due to either death or migration, as
well as periods from when
                        **the control group sufer their concussion
```

```
                drop if merge ==1 | count >
`control_time'-1
        }

        gen female = koen==2

                **Generate educational groups
        gen edu =0
        replace edu = 1 if inrange(hffsp,20000000,39000000)
| ///

(hffsp >40000000 & hffsp!=.)


        //build ident, so we can multivariate cluster for
individuals
        //who occur both as control and exposure during the
period (id)

        bysort pnr time: gen helpx = _n ==1
        gen id= sum(helpx)
        drop helpx

        **Calculate number of observations for exposure and
control
        count if count==0 & treatment ==1
        local Ntreated = r(N)
        count if count==0 & treatment ==0
        local Ncontrol = r(N)

        **generate concussion variable
        gen treat = inrange(count,0,`control_time'-1) &
treatment ==1
        replace treat = time_from_incident if count ==0 &
treatment ==1

        **Generate pre-concussion income difference
for
        **use in calculating marginal effects
        sum loenmv if count <0 & treatment ==0
        local control =r(mean)
        sum loenmv if count <0 & treatment ==1
        local treat =r(mean)
        sum loenmv if count>=0 & treatment ==0
        local control_post =r(mean)

        forvalue t=-4/4{
                local n = `t'*-1
                if `t' < -1 gen T_`n' = treatment ==1 &
count ==`t'
                if `t' > -1 gen T`t' = treatment ==1 &
count ==`t'

        }
```

```
                gen post = count > -1

                **estimate DiD model on salary
                reghdfe loenmv treat, abs(alder female post time
treatment edu) cl(pnr id)
                eststo est1_`control_time'
                if `control_time'==1 matrix results = J(5,5,.) //
matrix to capture results
                if `control_time'==1 matrix results_p = J(5,5,.) //
matrix to capture results


                matrix b = e(b)
                matrix V = e(V)
                local n = `control_time'

                matrix results[`n',1]    = b[1,1] / 7466  //
capture beta results as 1K Euro
                matrix results[`n',2]    = (V[1,1]^.5)/
7466    // capture standard error as 1K Euro
                matrix results[`n',3]    =       b[1,1]/
(`control_post'-(`control'-`treat'))
                matrix results[`n',4]    =       `n'


        }
        *examining balance of samples
        di in ye `control_time'
        bysort treatment: sum female alder edu if count ==0

}



svmat results

gen upper = results1+results2*1.96
gen lower = results1-results2*1.96

scatter results1 results4 if results4 ==1, mcolor(navy) || ///
scatter results1 results4 if results4 ==2, mcolor(navy) || ///
scatter results1 results4 if results4 ==3, mcolor(navy) || ///
scatter results1 results4 if results4 ==4, mcolor(navy) || ///
scatter results1 results4 if results4 ==5, mcolor(navy) || ///
rspike upper lower results4 if results4 ==1, lcolor(navy) || ///
rspike upper lower results4 if results4 ==2, lcolor(navy) || ///
rspike upper lower results4 if results4 ==3, lcolor(navy) || ///
rspike upper lower results4 if results4 ==4, lcolor(navy) || ///
rspike upper lower results4 if results4 ==5, lcolor(navy)  ///
ysc(range(-2 1)) ylab(-2(.5)1) ///
xsc(range(.5 5.5)) xlab(1(1)5) ///
yline(0) ysize(10) xsize(12) graphr(c(white)) ///
xti("Years between exposure and control incident") scale(.95) ///
```

```
legend(label(1 " Control," "{&Delta}=1 yr") ///
label(2 " Control," "{&Delta}=2 yr") ///
label(3 " Control," "{&Delta}=3 yr") ///
label(4 " Control," "{&Delta}=4 yr") ///
label(5 " Control," "{&Delta}=5 yr") ///
c(1) order(1 2 3 4 5) pos(3) size(small) ///
c(1) symx(4) region(lc(white))) ///
yti("Effect in 1K Euro ({&delta}{subscript: Salary})",
height(7)) ///
legend(off)

graph export $highdef/est2003_2011.png, replace width(3900)


**Reports marginal effects for period 2003-2011 in percent

gen upper2 = (results3+results2/(results1/results3)*1.96)*100
gen lower2 = (results3-results2/(results1/results3)*1.96)*100
replace results3 = results3*100

scatter results3 results4 , mcolor(navy) || ///
rspike upper2 lower2 results4, lcolor(navy) ///
ysc(range(-5 0)) ylab(-5(.5)0) ///
xsc(range(.5 5.5)) xlab(1(1)5) ///
yline(0) ysize(10) xsize(12) graphr(c(white)) ///
/*title("Percentage change in salary, 2003-10")*/ ///
yti("Salary change (in %)", height(7)) ///
xti("Years between exposure and control incident") scale(.95) ///
legend(label(1 " Control," "{&Delta}=1 yr") ///
label(2 " Control," "{&Delta}=2 yr") ///
label(3 " Control," "{&Delta}=3 yr") ///
label(4 " Control," "{&Delta}=4 yr") ///
label(5 " Control," "{&Delta}=5 yr") ///
c(1) order(1 2 3 4 5) pos(3) size(small) ///
c(1) symx(4) region(lc(white)))  legend(off)
/*
note("Marginal effects for exposure dummy across spacing of control
groups. Decrease " ///
"calculated by dividing {&delta} with the normalized control groups'
average salary " ///
"post-concussion. Control groups suffer concussions 1, 2, 3, 4, and
5 years (&Delta) after" ///
"the exposure group. Both control and exposure group are 30-49 years
of age when" ///
"exposure group suffers concussion. 95% confidence intervals.")*/

graph export $highdef/marginal2003_2011.png, replace width(3900)




*******************************************************************
***********
**
```

```
**              Results for individuals with at least highschool
**
**
*******************************************************************
***********


forvalue control_time=1/5{
        local end = 2012 // last incident year in data

        **build dataset for joint estimate across years
        forvalue count=2003/`end'{
                if `count'==2003{
                        use $data/
sample_control_`count'_`control_time'.dta, clear
                        gen time = `count' //incident year
indicator
                }
                else append using $data/
sample_control_`count'_`control_time'.dta
                replace time = `count' if time ==.

                **exclude individuals in years where they do not
appear in data,
                **due to either death or migration, as well as
periods from when
                **the control group sufer their concussion
                drop if merge ==1 | count > `control_time'-1


        }

        gen female = koen==2

        **Generate educational groups
        qui{
                gen edu =0
                replace edu = 1 if inrange(hffsp,20000000,39000000)
| ///
                                                                 (hffsp
>40000000 & hffsp!=.)
        }

        keep if edu==1

        //build ident, so we can multivariate cluster for
individuals
        //who occur both as control and exposure during the period
(id)

        bysort pnr time: gen helpx = _n ==1
        gen id= sum(helpx)
        drop helpx help
```

```
        **Calculate number of observations for exposure and control
        count if count==0 & treatment ==1
        local Ntreated = r(N)
        count if count==0 & treatment ==0
        local Ncontrol = r(N)

        **generate concussion variable
        gen treat = inrange(count,0,`control_time'-1) & treatment 
==1
        replace treat = time_from_incident if count ==0 & treatment 
==1

        **Generate pre-concussion income difference        for
        **use in calculating marginal effects
        sum loenmv if count <0 & treatment ==0
        local control =r(mean)
        sum loenmv if count <0 & treatment ==1
        local treat =r(mean)
        sum loenmv if count>=0 & treatment ==0
        local control_post =r(mean)

        gen post = count >=0

        **estimate DiD model on salary
        xi: reghdfe loenmv treat, abs(alder female post time 
treatment) cl(id pnr)

        if `control_time'==1 matrix results_edu = J(5,5,.) // 
matrix to capture results
        if `control_time'==1 matrix results_p_edu = J(5,5,.) // 
matrix to capture results


        matrix b = e(b)
        matrix V = e(V)
        local n = `control_time'

        matrix results_edu[`n',1]         = b[1,1] / 7466  // 
capture beta results as 1K Euro
        matrix results_edu[`n',2]         = (V[1,1]^.5)/
7466    // capture standard error as 1K Euro
        matrix results_edu[`n',3] =       b[1,1]/(`control_post'-
(`control'-`treat'))
        matrix results_edu[`n',4] =       `n'


}

*********************************************************************
***********
**
**              Results for individuals with no high school+
**
**
```

```
*****************************************************************
***********


forvalue control_time=1/5{
        local end = 2012 // last incident year in data

        **build dataset for joint estimate across years
        forvalue count=2003/`end'{
                if `count'==2003{
                        use $data/
sample_control_`count'_`control_time'.dta, clear
                        gen time = `count' //incident year
indicator
                }
                else append using $data/
sample_control_`count'_`control_time'.dta
                replace time = `count' if time ==.

                **exclude individuals in years where they do not
appear in data,
                **due to either death or migration, as well as
periods from when
                **the control group sufer their concussion
                drop if merge ==1 | count > `control_time'-1


        }

        gen female = koen==2

        //build ident, so we can multivariate cluster for
individuals
        //who occur both as control and exposure during the period
(id)

        bysort pnr time: gen helpx = _n ==1
        gen id= sum(helpx)
        drop helpx help

        **Generate educational groups
        qui{
                gen edu =0
                replace edu = 1 if inrange(hffsp,20000000,39000000)
| ///
                                                                   (hffsp
>40000000 & hffsp!=.)
        }

        keep if edu==0



        **Calculate number of observations for exposure and control
```

```
        count if count==0 & treatment ==1
        local Ntreated = r(N)
        count if count==0 & treatment ==0
        local Ncontrol = r(N)

        **generate concussion variable
        gen treat = inrange(count,0,`control_time'-1) & treatment
==1
        replace treat = time_from_incident if count ==0 & treatment
==1

        **Generate pre-concussion income difference       for
        **use in calculating marginal effects
        sum loenmv if count <0 & treatment ==0
        local control =r(mean)
        sum loenmv if count <0 & treatment ==1
        local treat =r(mean)
        sum loenmv if count>=0 & treatment ==0
        local control_post =r(mean)
        gen post = count >=0

        **estimate DiD model on salary
        xi: reghdfe loenmv treat, abs(alder female post time
treatment) cl(id pnr)

        if `control_time'==1 matrix results_noedu = J(5,5,.) //
matrix to capture results
        if `control_time'==1 matrix results_p_noedu = J(5,5,.) //
matrix to capture results


        matrix b = e(b)
        matrix V = e(V)
        local n = `control_time'

        matrix results_noedu[`n',1]      = b[1,1] / 7466  //
capture beta results as 1K Euro
        matrix results_noedu[`n',2]      = (V[1,1]^.5)/
7466    // capture standard error as 1K Euro
        matrix results_noedu[`n',3]      =       b[1,1]/
(`control_post'-(`control'-`treat'))
        matrix results_noedu[`n',4]      =       `n'



}
*********************************************************************
***********
**
**      Draw figure for subgroups
**
**
*********************************************************************
***********
```

```
local t= -.15

foreach x in noedu edu{
        svmat results_`x'
        replace results_`x'4= results_`x'4+`t'
        svmat results_p_`x'
        replace results_p_`x'4= results_p_`x'4+`t'

        gen upper`x' = results_`x'1+results_`x'2*1.96
        gen lower`x' = results_`x'1-results_`x'2*1.96

        gen upper2`x' = (results_`x'3+results_`x'2/(results_`x'1/
results_`x'3)*1.96)*100
        gen lower2`x' = (results_`x'3-results_`x'2/(results_`x'1/
results_`x'3)*1.96)*100
        replace results_`x'3 = results_`x'3*100

        gen upper_p_`x' = results_p_`x'1+results_p_`x'2*1.96
        gen lower_p_`x' = results_p_`x'1-results_p_`x'2*1.96

        local t = `t'+.1

}


keep results* upper* lower*
keep if _n <=5

**generate locals for figure

foreach x in noedu edu{
        if "`x'" == "nopay" local color = "navy"
        if "`x'" == "pay"         local color = "red"
        if "`x'" == "noedu" local color = "green"
        if "`x'" == "edu"         local color = "purple"

        local figure_`x' "scatter results_`x'1 results_`x'4,
mcolor(`color') || rspike upper`x' lower`x' results_`x'4,
lcolor(`color') vertical"
        if "`x'" == "nopay" local figure2_`x' "scatter results_`x'3
results_`x'4, mcolor(`color') || rspike upper2`x' lower2`x'
results_`x'4, lcolor(`color' )  vertical "
        else local figure2_`x' "scatter results_`x'3 results_`x'4,
mcolor(`color') || rspike upper2`x' lower2`x' results_`x'4,
lcolor(`color')  vertical "
        local figure_p_`x' "scatter results_p_`x'1 results_p_`x'4,
mcolor(`color') || rspike upper_p_`x' lower_p_`x' results_p_`x'4,
lcolor(`color')  vertical "
}


`figure_noedu' || `figure_edu'  ///
legend( ///
```

```
label(1 "Less than" "high school") ///
label(3 "At least" "high school") ///
c(1) order(1 3 ) pos(3) size(small) ///
c(1) symx(4) region(lc(white))) ///
ysc(range(-4 2)) ylab(-4(1)2) ///
xsc(range(.5 5.5)) xlab(1(1)5) ///
yline(0) ysize(10) xsize(12) graphr(c(white)) ///
///title("Parameter estimates across control group, 2003-10") ///
xti("Years between exposure and control incident") scale(.95) ///
yti("Effect in 1K Euro ({&delta}{subscript: Salary})",
height(7)) ///
/*note("Parameter estimates for exposure dummy across spacing of
control groups." ///
"Control groups suffer concussions 1, 2, 3, 4, and 5 years (&Delta)
after the exposure group." ///
"Both control and exposure group are 30-49 years of age when
exposure group suffers " ///
"concussion. 95% confidence intervals.")
*/

graph export $highdef/grouped_est2003_2011.png, replace width(3900)

preserve



`figure2_noedu' || `figure2_edu'  ///
legend( ///
label(1 "Less than" "high school") ///
label(3 "At least" "high school") ///
c(1) order(1 3 5) pos(3) size(small) ///
c(1) symx(4) region(lc(white))) ///
ysc(range(-12 3)) ylab(-12(3)3) ///
xsc(range(.5 5.5)) xlab(1(1)5) ///
yline(0) ysize(10) xsize(12) graphr(c(white)) ///
/// title("Percentage change in salary, 2003-10") ///
yti("Salary change (in %)", height(7)) ///
xti("Years between exposure and control incident") scale(.95) ///
/*note("Marginal effects for exposure dummy across spacing of
control groups." ///
"change calculated by {&delta} with the normalized control groups'
average" ///
"salary post-concussion. Control groups suffer concussions 1, 2, 3,
4, and 5 years (&Delta) after" ///
"the exposure group. Both control and exposure group are 30-49 years
of age when" ///
"exposure group suffers concussion.  95% confidence intervals.")*/

graph export $highdef/grouped_marginal2003_2011.png, replace
width(3900)

restore
```

```
*********************************************************************
***********
**
**               Results different age-groups
**
**
*********************************************************************
***********

forvalue y=20(5)55{
        forvalue control_time=1/5{
                local end = 2012 // last incident year in data

                **build dataset for joint estimate across years
                forvalue count=2003/`end'{
                        if `count'==2003{
                                use $data/
sample_control_`count'_`control_time'.dta, clear
                                gen time = `count' //incident year
indicator
                        }
                        else append using $data/
sample_control_`count'_`control_time'.dta
                        replace time = `count' if time ==.

                        **exclude individuals in years where they
do not appear in data,
                        **due to either death or migration, as
well as periods from when
                        **the control group sufer their concussion
                        drop if merge ==1 | count >
`control_time'-1


                }

                gen female = koen==2

                //build ident, so we can multivariate cluster for
individuals
                //who occur both as control and exposure during the
period (id)

                bysort pnr time: gen helpx = _n ==1
                gen id= sum(helpx)
                drop helpx help

                gen nopay = loenmv <1

                **Generate age group
                local z = `y'+4
                gen help = count == 0 & inrange(alder,`y',`z')
                bysort id: egen helpx =max(help)
```

```
                keep if helpx == 1
                drop helpx help




                **Calculate number of observations for exposure and
control
                count if count==0 & treatment ==1
                local Ntreated = r(N)
                count if count==0 & treatment ==0
                local Ncontrol = r(N)

                **generate concussion variable
                gen treat = inrange(count,0,`control_time'-1) &
treatment ==1
                replace treat = time_from_incident if count ==0 &
treatment ==1

                **Generate pre-concussion income difference
for
                **use in calculating marginal effects
                sum loenmv if count <0 & treatment ==0
                local control =r(mean)
                sum loenmv if count <0 & treatment ==1
                local treat =r(mean)
                sum loenmv if count>=0 & treatment ==0
                local control_post =r(mean)
                gen post = count >=0

                **estimate DiD model on salary
                xi: reghdfe loenmv treat, abs(alder female post
time treatment) cl(id pnr)

                if `control_time'==1 matrix results_`y' =
J(5,5,.) // matrix to capture results
                if `control_time'==1 matrix results_p_`y' =
J(5,5,.) // matrix to capture results


                matrix b = e(b)
                matrix V = e(V)
                local n = `control_time'

                matrix results_`y'[`n',1]         = b[1,1] /
7466    // capture beta results as 1K Euro
                matrix results_`y'[`n',2]         = (V[1,1]^.5)/
7466    // capture standard error as 1K Euro
                matrix results_`y'[`n',3] =       b[1,1]/
(`control_post'-(`control'-`treat'))
                matrix results_`y'[`n',4] =       `n'


        }
```

```
}

local t = -.15   //Jitter estimates along x-axis
forvalue x =20(5)55{
        svmat results_`x'
        replace results_`x'4= results_`x'4+`t'
        svmat results_p_`x'
        replace results_p_`x'4= results_p_`x'4+`t'

        gen upper`x' = results_`x'1+results_`x'2*1.96
        gen lower`x' = results_`x'1-results_`x'2*1.96

        gen upper2`x' = (results_`x'3+results_`x'2/(results_`x'1/
results_`x'3)*1.96)*100
        gen lower2`x' = (results_`x'3-results_`x'2/(results_`x'1/
results_`x'3)*1.96)*100
        replace results_`x'3 = results_`x'3*100

        gen upper_p_`x' = results_p_`x'1+results_p_`x'2*1.96
        gen lower_p_`x' = results_p_`x'1-results_p_`x'2*1.96

        local t = `t'+.1

}

keep results* upper* lower*
keep if _n <=5

**generate locals for figure

forvalue x =20(5)55{
        if `x' == 20 local color = "black"
        if `x' == 25 local color = "orange"
        if `x' == 30 local color = "navy"
        if `x' == 35 local color = "red"
        if `x' == 40 local color = "green"
        if `x' == 45 local color = "purple"
        if `x' == 50 local color = "sienna"
        if `x' == 55 local color = "teal"

        local figure_`x' "scatter results_`x'1 results_`x'4,
mcolor(`color') || rspike upper`x' lower`x' results_`x'4,
lcolor(`color') vertical"
        local figure2_`x' "scatter results_`x'3 results_`x'4,
mcolor(`color') || rspike upper2`x' lower2`x' results_`x'4,
lcolor(`color')  vertical "
        local figure_p_`x' "scatter results_p_`x'1 results_p_`x'4,
mcolor(`color') || rspike upper_p_`x' lower_p_`x' results_p_`x'4,
lcolor(`color')  vertical "
}

/**/
```

```
`figure_20' || `figure_25' || `figure_30' || `figure_35' ///
        || `figure_40' || `figure_45' || `figure_50' ||
`figure_55'  ///
legend( ///
label(1 "Age 20-24") ///
label(3 "Age 25-29") ///
label(5 "Age 30-34") ///
label(7 "Age 35-39") ///
label(9 "Age 40-44") ///
label(11 "Age 45-49") ///
label(13 "Age 50-54") ///
label(15 "Age 55-59") ///
c(1) order(1 3 5 7 9 11 13 15) pos(3) size(small) ///
c(1) symx(4) region(lc(white))) ///
ysc(range(-4 2)) ylab(-4(1)2) ///
xsc(range(.5 5.5)) xlab(1(1)5) ///
yline(0) ysize(10) xsize(12) graphr(c(white)) ///
/// title("Parameter estimates across control group, 2003-10") ///
xti("Years between exposure and control incident") scale(.95) ///
yti("Effect in 1K Euro ({&delta}{subscript: Salary})",
height(7)) ///
/*note("Parameter estimates for exposure dummy across spacing of
control groups." ///
"Control groups suffer concussions 1, 2, 3, 4, and 5 years (&Delta)
after the exposure group." ///
"Age group described age at time of exposure incident. 95%
confidence intervals.")*/

graph export $highdef/age_est2003_2011.png, replace width(3900)
*/

`figure2_20' || `figure2_25' || `figure2_30' || `figure2_35' ///
        || `figure2_40' || `figure2_45' || `figure2_50' ||
`figure2_55'  ///
legend( ///
label(1 "Age 20-24") ///
label(3 "Age 25-29") ///
label(5 "Age 30-34") ///
label(7 "Age 35-39") ///
label(9 "Age 40-44") ///
label(11 "Age 45-49") ///
label(13 "Age 50-54") ///
label(15 "Age 55-59") ///
c(1) order(1 3 5 7 9 11 13 15) pos(3) size(small) ///
c(1) symx(4) region(lc(white))) ///
ysc(range(-12 3)) ylab(-12(3)3) ///
xsc(range(.5 5.5)) xlab(1(1)5) ///
yline(0) ysize(10) xsize(12) graphr(c(white)) ///
yti("Salary change (in %)", height(7)) ///
xti("Years between exposure and control incident") scale(.95)


graph export $highdef/age_marginal2003_2011.png, replace width(3900)
```

```
`figure_p_30' || `figure_p_35' || `figure_p_40' ||
`figure_p_45'  ///  ///
legend(label(1 "Age 30-34") ///
label(3 "Age 35-39") ///
label(5 "Age 40-44") ///
label(7 "Age 45-49") ///
c(1) order(1 3 5 7) pos(3) size(small) ///
c(1) symx(4) region(lc(white))) ///
ysc(range(-.02 .050)) ylab(-0.02(.01)0.05) ///
xsc(range(.5 5.5)) xlab(1(1)5) ///
yline(0, lcolor(black)) ysize(10) xsize(12) graphr(c(white)) ///
title("Parameter estimates across control group, 2003-10") ///
yti("Effect on Pr(Salary=0)", height(7)) ///
xti("Years between exposure and control incident ({&Delta})")
scale(.95) ///
note("Parameter estimates for exposure dummy across spacing of
control groups." ///
"Control groups suffer concussions 1, 2, 3, 4, and 5 (&Delta) years
after the exposure group." ///
"Age group described age at time of exposure incident. 95%
confidence intervals.")

graph export $highdef/age_nopay2003_2011.png, replace width(3900)


*********************************************************************
***********
**
**               Results accross gender
**
**
*********************************************************************
***********

forvalue y=0/1{
        forvalue control_time=1/5{
                local end = 2012 // last incident year in data

                **build dataset for joint estimate across years
                forvalue count=2003/`end'{
                        if `count'==2003{
                                use $data/
sample_control_`count'_`control_time'.dta, clear
                                gen time = `count' //incident year
indicator
                        }
                        else append using $data/
sample_control_`count'_`control_time'.dta
                        replace time = `count' if time ==.

                        **exclude individuals in years where they
do not appear in data,
                        **due to either death or migration, as
well as periods from when
```

```
                    **the control group sufer their concussion
                    drop if merge ==1 | count > 
`control_time'-1


            }

            gen female = koen==2

            //build ident, so we can multivariate cluster for 
individuals
            //who occur both as control and exposure during the 
period (id)

            bysort pnr time: gen helpx = _n ==1
            gen id= sum(helpx)
            drop helpx help

            gen nopay = loenmv <1

            **Generate age group
            local z = `y'+4
            gen help = count == 0 & female==`y'
            bysort id: egen helpx =max(help)
            keep if helpx == 1
            drop helpx help




            **Calculate number of observations for exposure and 
control
            count if count==0 & treatment ==1
            local Ntreated = r(N)
            count if count==0 & treatment ==0
            local Ncontrol = r(N)

            **generate concussion variable
            gen treat = inrange(count,0,`control_time'-1) & 
treatment ==1
            replace treat = time_from_incident if count ==0 & 
treatment ==1

            **Generate pre-concussion income difference 
for
            **use in calculating marginal effects
            sum loenmv if count <0 & treatment ==0
            local control =r(mean)
            sum loenmv if count <0 & treatment ==1
            local treat =r(mean)
            sum loenmv if count>=0 & treatment ==0
            local control_post =r(mean)
            gen post = count >=0
```

```
                  **estimate DiD model on salary
                  xi: reghdfe loenmv treat, abs(alder post time
treatment) cl(id pnr)

                  if `control_time'==1 matrix results_`y' =
J(5,5,.) // matrix to capture results
                  if `control_time'==1 matrix results_p_`y' =
J(5,5,.) // matrix to capture results


                  matrix b = e(b)
                  matrix V = e(V)
                  local n = `control_time'

                  matrix results_`y'[`n',1]        = b[1,1] /
7466    // capture beta results as 1K Euro
                  matrix results_`y'[`n',2]        = (V[1,1]^.5)/
7466    // capture standard error as 1K Euro
                  matrix results_`y'[`n',3] =      b[1,1]/
(`control_post'-(`control'-`treat'))
                  matrix results_`y'[`n',4] =      `n'


         }
}



local t = -.05   //Jitter estimates along x-axis
foreach x in 0 1{
         svmat results_`x'
         replace results_`x'4= results_`x'4+`t'
         svmat results_p_`x'
         replace results_p_`x'4= results_p_`x'4+`t'

         gen upper`x' = results_`x'1+results_`x'2*1.96
         gen lower`x' = results_`x'1-results_`x'2*1.96

         gen upper2`x' = (results_`x'3+results_`x'2/(results_`x'1/
results_`x'3)*1.96)*100
         gen lower2`x' = (results_`x'3-results_`x'2/(results_`x'1/
results_`x'3)*1.96)*100
         replace results_`x'3 = results_`x'3*100

         gen upper_p_`x' = results_p_`x'1+results_p_`x'2*1.96
         gen lower_p_`x' = results_p_`x'1-results_p_`x'2*1.96

         local t = `t'+.1

}

keep results* upper* lower*
keep if _n <=5
```

```
**generate locals for figure

foreach x in 0 1{
        if `x' == 0 local color = "red"
        if `x' == 1 local color = "green"

        local figure_`x' "scatter results_`x'1 results_`x'4,
mcolor(`color') || rspike upper`x' lower`x' results_`x'4,
lcolor(`color') vertical"
        local figure2_`x' "scatter results_`x'3 results_`x'4,
mcolor(`color') || rspike upper2`x' lower2`x' results_`x'4,
lcolor(`color')  vertical "
        local figure_p_`x' "scatter results_p_`x'1 results_p_`x'4,
mcolor(`color') || rspike upper_p_`x' lower_p_`x' results_p_`x'4,
lcolor(`color')  vertical "
}


`figure_0' || `figure_1'  ///
legend(label(1 "Men") ///
label(3 "Women") ///
c(1) order(1 3) pos(3) size(small) ///
c(1) symx(4) region(lc(white))) ///
ysc(range(-4 2)) ylab(-4(1)2) ///
xsc(range(.5 5.5)) xlab(1(1)5) ///
yline(0) ysize(10) xsize(12) graphr(c(white)) ///
/// title("Parameter estimates across control group, 2003-10") ///
xti("Years between exposure and control incident") scale(.95) ///
yti("Effect in 1K Euro ({&delta}{subscript: Salary})",
height(7)) ///
/*note("Parameter estimates for exposure dummy across spacing of
control groups." ///
"Control groups suffer concussions 1, 2, 3, 4, and 5 years (&Delta)
after the exposure group." ///
"95% confidence intervals.")*/

graph export $highdef/gender_est2003_2011.png, replace width(3900)

`figure2_0' || `figure2_1'   ///
legend( ///
label(1 "Men") ///
label(3 "Women") ///
c(1) order(1 3 5 7) pos(3) size(small) ///
c(1) symx(4) region(lc(white))) ///
ysc(range(-12 3)) ylab(-12(3)3) ///
xsc(range(.5 5.5)) xlab(1(1)5) ///
yline(0) ysize(10) xsize(12) graphr(c(white)) ///
/// title("Percentage change in salary, 2003-10") ///
yti("Salary change (in %)", height(7)) ///
xti("Years between exposure and control incident") scale(.95) ///
/* note("Parameter estimates for exposure dummy across spacing of
control groups." ///
"Control groups suffer concussions 1, 2, 3, 4, and 5 years (&Delta)
after the exposure group." ///
```

```
"95% confidence intervals.")*/

graph export $highdef/gender_marginal2003_2011.png, replace
width(3900)

`figure_p_0' || `figure_p_1'   ///
legend( ///
label(1 "Men") ///
label(3 "Women") ///
c(1) order(1 3 5 7) pos(3) size(small) ///
c(1) symx(4) region(lc(white))) ///
ysc(range(-.02 .050)) ylab(-0.02(.01)0.05) ///
xsc(range(.5 5.5)) xlab(1(1)5) ///
yline(0, lcolor(black)) ysize(10) xsize(12) graphr(c(white)) ///
/// title("Parameter estimates across control group, 2003-10") ///
yti("Effect on Pr(Salary=0)", height(7)) ///
xti("Years between exposure and control incident ({&Delta})")
scale(.95) ///
/*note("Parameter estimates for exposure dummy across spacing of
control groups." ///
"Control groups suffer concussions 1, 2, 3, 4, and 5 years (&Delta)
after the exposure group." ///
"95% confidence intervals.")*/

graph export $highdef/gender_nopay2003_2011.png, replace width(3900)


*********************************************************************
************
**
**                       Generate descriptive figures of wage
development for exposure
**                        and control group
**
**
*********************************************************************
************


 forvalue control_time=1/5{
        local end = 2012 // last incident year in data

        **build dataset for joint estimate across years
        forvalue count=2003/`end'{
                if `control_time' ==1 & `count'==2003{
                        use $data/
sample_control_`count'_`control_time'.dta, clear
                        gen time = `count' //incident year
indicator
                }
                else append using $data/
sample_control_`count'_`control_time'.dta

                **Drops exposure-group already in the data
```

```
                if `control_time' > 1 drop if time ==. & treatment
==1
                replace time = `count' if time ==.
                replace control1 = `control_time' if
control`control_time'==1
                if `control_time' > 1 drop control`control_time'

                **exclude individuals in years where they do not
appear in data,
                **due to either death or migration, as well as
periods from when
                **the control group suffer their concussion

        }

}

**Generate mean salary for each period relative to exposure groups
concussion
**separately for exposure and each control group

bysort control1 count: egen mean_loen = mean(loenmv)

**Generate mean Pr(sal= for each period relative to exposure groups
concussion
**separately for exposure and each control group

gen no_lon = loenmv < 1

bysort control1 count: egen mean_no_lon = mean(no_lon)

**generate group size
bysort control1 count: gen Ncount=_N if count ==0

**generate pre-exposure mean levels for normalization
gen pre = count <0
bysort control1 pre: egen pre_mean_loen = mean(loenmv) if pre==1
bysort control1 pre: egen pre_mean_no_lon = mean(no_lon) if pre==1


**reduce data set size
bysort control1 count: keep if _n ==1

keep count mean* pre_* control1 Ncount

**standardize to 1k euro
replace mean_loen = mean_loen/7466
replace pre_mean_loen = pre_mean_loen/7466

sort control1 count


**Pre-tratment normalization of salary
```

```
gen norm_mean_lon =mean_loen
gen norm_mean_no_lon =mean_no_lon

forvalue t = 1/5{
        qui sum pre_mean_loen if control == 0
        local treat = r(mean)
        qui sum pre_mean_loen if control == `t'
        local control = r(mean)

        **normalize with pre-concussion difference
        qui replace norm_mean_lon = mean_loen - (`control'-`treat')
if control ==`t'

        qui sum pre_mean_no_lon if control == 0
        local treat = r(mean)
        qui sum pre_mean_no_lon if control == `t'
        local control = r(mean)

        **normalize with pre-concussion difference
        qui replace norm_mean_no_lon = mean_no_lon - (`control'-
`treat') if control ==`t'

}

**local indicators of group sizes
forvalue t=0/5{
        qui sum Ncount if control == `t'
        local C`t' = r(mean)
}


graph twoway ///
        connect mean_l count if control1== 0, ///
        lcolor(black) mcolor(black) || ///
        connect mean_l count if control1== 1, ///
        lcolor(blue)  mcolor(blue) || ///
        connect mean_l count if control1== 2, ///
        lcolor(green) mcolor(green) || ///
        connect mean_l count if control1== 3, ///
        lcolor(purple) mcolor(purple) || ///
        connect mean_l count if control1== 4, ///
        lcolor(red) mcolor(red) || ///
        connect mean_l count if control1== 5, ///
        lcolor(orange) mcolor(orange) ///
        legend( ///
                label(1 "Exposure" ///
                                "N=`C0'") ///
                label(2 "Control {&Delta}=1" ///
                                "N=`C1'") ///
                label(3 "Control {&Delta}=2" ///
                                "N=`C2'") ///
                label(4 "Control {&Delta}=3" ///
                                "N=`C3'") ///
                label(5 "Control {&Delta}=4" ///
```

```
                                "N=`C4'") ///
                    label(6 "Control {&Delta}=5" ///
                                "N=`C5'") ///
                    c(1) order(1 2 3 4 5 6) pos(3) size(small) ///
                    c(1) symx(4) region(lc(white))) ///
        ysc(range(26 33)) ylab(26(1)33) ///
        xsc(range(-5 5)) xlab(-5(1)5) ///
        xline(0, lcolor(red)) ysize(10) xsize(12)
graphr(c(white)) ///
///     title("Impact of Concussion on Salary") ///
        yti("Deflated Salaried Income in 1K EUR", height(7)) ///
        xti("Years since exposure group concussion") scale(.95) ///
/*      note("Control groups suffer concussion 1, 2, 3, 4, and 5
years (&Delta) after exposure group." ///
                    "Both control and exposure group at 30-49 years of
age at beginning of year =0." ///
                    "Vertical line indicates time of exposure group
concussion.")*/

graph export $highdef/FigureS2.png, replace width(3900)


graph twoway ///
        connect norm_mean_l count if control1== 1, ///
        lcolor(blue)  mcolor(blue) || ///
        connect norm_mean_l count if control1== 2, ///
        lcolor(green) mcolor(green) || ///
        connect norm_mean_l count if control1== 3, ///
        lcolor(purple) mcolor(purple) || ///
        connect norm_mean_l count if control1== 4, ///
        lcolor(red) mcolor(red) || ///
        connect norm_mean_l count if control1== 5, ///
        lcolor(orange) mcolor(orange) || ///
        connect mean_l count if control1== 0, ///
        lcolor(black) mcolor(black)  ///
        legend( ///
                    label(1 "Control {&Delta}=1" ///
                                "N=`C1'") ///
                    label(2 "Control {&Delta}=2" ///
                                "N=`C2'") ///
                    label(3 "Control {&Delta}=3" ///
                                "N=`C3'") ///
                    label(4 "Control {&Delta}=4" ///
                                "N=`C4'") ///
                    label(5 "Control {&Delta}=5" ///
                                "N=`C5'") ///
                    label(6 "Exposure" ///
                                "N=`C0'") ///
                    c(1) order(6 1 2 3 4 5) pos(3) size(small) ///
                    c(1) symx(4) region(lc(white))) ///
        ysc(range(26 31)) ylab(26(1)31) ///
        xsc(range(-5 5)) xlab(-5(1)5) ///
        xline(0, lcolor(red)) ysize(10) xsize(12)
graphr(c(white)) ///
```

```
///     title("Impact of Concussion on Salary") ///
        yti("Deflated Norm. Salaried Income in 1K EUR",
height(7)) ///
        xti("Years since exposure group's concussion") scale(.95)
        /*///
        note("Control groups suffer concussion 1, 2, 3, 4, and 5
years (&Delta) after exposure group." ///
                "Both control and exposure group at 30-49 years of
age at beginning of year =0." ///
                "Vertical line indicates time of exposure group
concussion." ///
                "Salary levels normalized with pre-concussion level
difference between" ///
                "each control group and the exposure group")*/

graph export $highdef/Figure2.png, replace width(3900)


graph twoway ///
        connect mean_no_l count if control1== 0, ///
        lcolor(black) mcolor(black) || ///
        connect mean_no_l count if control1== 1, ///
        lcolor(blue)  mcolor(blue) || ///
        connect mean_no_l count if control1== 2, ///
        lcolor(green) mcolor(green) || ///
        connect mean_no_l count if control1== 3, ///
        lcolor(purple) mcolor(purple) || ///
        connect mean_no_l count if control1== 4, ///
        lcolor(red) mcolor(red) || ///
        connect mean_no_l count if control1== 5, ///
        lcolor(orange) mcolor(orange) ///
        legend( ///
                label(1 "Exposure" ///
                                 "N=`C0'") ///
                label(2 "Control {&Delta}=1" ///
                                 "N=`C1'") ///
                label(3 "Control {&Delta}=2" ///
                                 "N=`C2'") ///
                label(4 "Control {&Delta}=3" ///
                                 "N=`C3'") ///
                label(5 "Control {&Delta}=4" ///
                                 "N=`C4'") ///
                label(6 "Control {&Delta}=5" ///
                                 "N=`C5'") ///
                c(1) order(1 2 3 4 5 6) pos(3) size(small) ///
                c(1) symx(4) region(lc(white))) ///
        ysc(range(.2 .325)) ylab(.2(.025).325) ///
        xsc(range(-5 5)) xlab(-5(1)5) ///
        xline(0, lcolor(red)) ysize(10) xsize(12)
graphr(c(white)) ///
///     title("Impact of Concussion on Prob(Salary=0)") ///
        yti("Prob(Salary=0)", height(7)) ///
        xti("Years since exposure group concussion") scale(.95) ///
/*      note("Control groups suffer concussion 1, 2, 3, 4, and 5
```

```
years (&Delta) after exposure group." ///
                  "Both control and exposure group at 30-49 years of
age at beginning of year =0." ///
                  "Vertical line indicates time of exposure group
concussion.")*/

graph export $highdef/FigureS3.png, replace width(3900)

graph twoway ///
        connect norm_mean_no_l count if control1== 1, ///
        lcolor(blue)  mcolor(blue) || ///
        connect norm_mean_no_l count if control1== 2, ///
        lcolor(green) mcolor(green) || ///
        connect norm_mean_no_l count if control1== 3, ///
        lcolor(purple) mcolor(purple) || ///
        connect norm_mean_no_l count if control1== 4, ///
        lcolor(red) mcolor(red) || ///
        connect norm_mean_no_l count if control1== 5, ///
        lcolor(orange) mcolor(orange) || ///
        connect mean_no_l count if control1== 0, ///
        lcolor(black) mcolor(black) ///
        legend( ///
                label(1 "Control {&Delta}=1" ///
                                 "N=`C1'") ///
                label(2 "Control {&Delta}=2" ///
                                 "N=`C2'") ///
                label(3 "Control {&Delta}=3" ///
                                 "N=`C3'") ///
                label(4 "Control {&Delta}=4" ///
                                 "N=`C4'") ///
                label(5 "Control {&Delta}=5" ///
                                 "N=`C5'") ///
                label(6 "Exposure" ///
                                 "N=`C0'") ///
                c(1) order(6 1 2 3 4 5) pos(3) size(small) ///
                c(1) symx(4) region(lc(white))) ///
        ysc(range(.2 .325)) ylab(.2(.025).325) ///
        xsc(range(-5 5)) xlab(-5(1)5) ///
        xline(0, lcolor(red)) ysize(10) xsize(12)
graphr(c(white)) ///
///     title("Impact of Concussion on Prob(Salary=0)") ///
        yti("Norm. Prob(Salary=0)", height(7)) ///
        xti("Years since exposure group concussion") scale(.95) ///
/*      note("Control groups suffer concussion 1, 2, 3, 4, and 5
years (&Delta) after exposure group." ///
                  "Both control and exposure group at 30-49 years of
age at beginning of year =0." ///
                  "Vertical line indicates time of exposure group
concussion." ///
                  "Probability levels normalized with pre-concussion
level difference between" ///
                  "each control group and the exposure group")*/

graph export $highdef/Figure2A.png, replace width(3900)
```

```
*************************************************************************
************
**
**      Effects across the salary distribution
**
**
**
*************************************************************************
************


forvalue control_time=5/5{
        local end = 2012 // last incident year in data

        **build dataset for joint estimate across years
        forvalue count=2003/`end'{
                if `count'==2003{
                        use $data/
sample_control_`count'_`control_time'.dta, clear
                        gen time = `count' //incident year
indicator
                }
                else append using $data/
sample_control_`count'_`control_time'.dta
                replace time = `count' if time ==.

                **exclude individuals in years where they do not
appear in data,
                **due to either death or migration, as well as
periods from when
                **the control group sufer their concussion
                drop if merge ==1 | count > `control_time'-1
        }

        gen female = koen==2
        qui{
                gen edu =0
                replace edu = 1 if inrange(hffsp,20000000,39000000)
| ///
                                                                (hffsp
>40000000 & hffsp!=.)
        }

        //build ident, so we can multivariate cluster for
individuals
        //who occur both as control and exposure during the period
(id)
```

```
        bysort pnr time: gen helpx = _n ==1
        gen id= sum(helpx)
        drop helpx


        **generate concussion variable
        gen treat = inrange(count,0,`control_time'-1) & treatment
==1
        replace treat = time_from_incident if count ==0 & treatment
==1

        **effect across salary distribution
        if `control_time'==5 matrix results = J(81,5,.) // matrix
to capture results

        local n = `control_time'
        gen post = count >=0
        replace count = count+6
        local v =0

        qui{
                forvalue t= 0(14932)1045240{
                        local top = 42
                        local v =1+`v'
                        if `n' ==5 matrix results[`v',1]= `t'
                        gen D = personindk <=`t'
                        reg D treat i.treatment i.post i.edu
i.alder i.female i.time, cl(pnr)
                        matrix V = e(V)
                        matrix b= e(b)
                        matrix results[`v',2]     = b[1,1]
                        margins, at(treat= (0 1) treatment=1
post=1)
                        matrix results[`v',5]     = V[1,1]^.5
                        matrix M = r(b)
                        matrix results[`v',4]     = M[1,2] //
                        matrix results[`v',3]     = M[1,1] //
                        drop D
                }
        }
}

svmat results

gen l5 = results2-1.96*results5
gen u5 = results2+1.96*results5


replace results1 = results1/7466

gen dif6 = results4-results3
gen udif6 = u5
gen ldif6 = l5
```

```
gr two rline udif6 ldif6 results1, ///
        yaxis(2) color(gray) lp(dash) ylab(0(.01).04, axis(2))
ysc(range(-.006 .04) axis(2))     || ///
        line dif6 results1, yaxis(2) yline(0, axis(2)) ///
        lcolor(black)  ylab(0(.01).04)  ysc(range(-.006 .04)
axis(2)) || ///
        line results4 results1, yaxis(1) lcolor(red) || ///
        line results3 results1, yaxis(1) lcolor(blue) ///
         ysc(range(0 1)) ylab(0(.1)1, nogrid) xlab(0(10)140)
xsc(range(0 140)) ///
        ysize(10) xsize(12) graphr(c(white)) ///
        xti("Total Income in 1K Euro") scale(1) ///
        yti("Effect of Concussion on Pr(Total Income < X)",
axis(2)) ///
        yti("{&Phi}(Total Income)",  axis(1)) ///
        legend( ///
        label(2 "Effect of concussion (left axis)") ///
        label(3 "Concussion income distribution (right axis)") ///
        label(4 "Counterfactual income distribution (right
axis)") ///
        c(1) order(2 3 4) pos(6) size(small) ///
                         symx(4) region(lc(white)))



graph export [home]\highdef\l5_income.png,  replace width(3900)

cap graph drop g1 g2

gr two ///
        line results4 results1, yaxis(1) lcolor(red) || ///
        line results3 results1, yaxis(1) lcolor(blue) ///
         ysc(range(.2 1)) ylab(0(.1)1) xlab(0(20)140,
labs(small)) ///
         xsc(range(0 140)) ///
        ysize(10) xsize(10) graphr(c(white)) ///
        xti("Total Income in 1K Euro") scale(1) ///
        yti("Cumulative Distribution of Total Income",
axis(1)) ///
        legend( ///
        label(1 "Observed Post-Concussion Total Income
Distribution") ///
        label(2 "Counterfactual No-Concussion Total Income
Distribution") ///
        c(1) order(1 2 4) pos(6) size(small) ///
                         symx(4) region(lc(white))) , name(g1)

gr two rline udif6 ldif6 results1, ///
        color(gray) lp(dash) ysc(range(-.002 .035)) || ///
        line dif6 results1,  yline(0) ///
        lcolor(black)  ylab(0(.01).0325)  ysc(range(-.002 .
035)) ///
        xlab(0(20)140, labs(small)) xsc(range(0 140))      ///
        ysize(10) xsize(10) graphr(c(white)) ///
```

```
          xti("Total Income in 1K Euro (X)") scale(1) ///
          yti("Effect of Concussion on Pr(Total Income < X)") ///
                    legend( label(1 "95% CI") ///
          label(2 "Effect of concussion") ///
          c(1) order(2 1) pos(6) size(small) ///
                               symx(4) region(lc(white))) , name(g2)

graph combine g1 g2      ,graphr(c(white))

graph export "[home]\highdef\figure s2.tif",  replace width(1000)



********************************************************************
************
**
**        Effects across the salary distribution
**
**
**
********************************************************************
************


forvalue control_time=5/5{
          local end = 2012 // last incident year in data

          **build dataset for joint estimate across years
          forvalue count=2003/`end'{
                    if `count'==2003{
                              use $data/
sample_control_`count'_`control_time'.dta, clear
                              gen time = `count' //incident year
indicator
                    }
                    else append using $data/
sample_control_`count'_`control_time'.dta
                    replace time = `count' if time ==.

                    **exclude individuals in years where they do not
appear in data,
                    **due to either death or migration, as well as
periods from when
                    **the control group sufer their concussion
                    drop if merge ==1 | count > `control_time'-1
          }

          gen female = koen==2
          qui{
                    gen edu =0
                    replace edu = 1 if inrange(hffsp,20000000,39000000)
| ///
                                                                   (hffsp
>40000000 & hffsp!=.)
```

```
        }

        //build ident, so we can multivariate cluster for
individuals
        //who occur both as control and exposure during the period
(id)

        bysort pnr time: gen helpx = _n ==1
        gen id= sum(helpx)
        drop helpx


        **generate concussion variable
        gen treat = inrange(count,0,`control_time'-1) & treatment
==1
        replace treat = time_from_incident if count ==0 & treatment
==1

        **effect across salary distribution
        if `control_time'==5 matrix results = J(81,5,.) // matrix
to capture results

        local n = `control_time'
        gen post = count >=0
        replace count = count+6
        local v =0

        **Estimate Pr(salary < X) across income distribution
        qui{
                forvalue t= 0(14932)895950{
                        local top = 42
                        local v =1+`v'
                        if `n' ==5 matrix results[`v',1]= `t'
                        gen D = loenmv <=`t'
                        reg D treat i.treatment i.post i.edu
i.alder i.female i.time, cl(pnr)
                        matrix V = e(V)
                        matrix b= e(b)
                        matrix results[`v',2]    = b[1,1]
                        margins, at(treat= (0 1) treatment=1
post=1)
                        matrix results[`v',5]    = V[1,1]^.5
                        matrix M = r(b)
                        matrix results[`v',4]    = M[1,2] //
                        matrix results[`v',3]    = M[1,1] //
                        drop D
                }
        }
}

svmat results

*Generate 95% confidence intervals
gen l5 = results2-1.96*results5
```

```
gen u5 = results2+1.96*results5

*Correct to €
replace results1 = results1/7466

*Obtain difference between observed and counterfactual wage
distribution
gen dif6 = results4-results3
gen udif6 = u5
gen ldif6 = l5

gr two rline udif6 ldif6 results1, ///
        yaxis(2) color(gray) lp(dash) ysc(range(-.006 .04) axis(2))
|| ///
        line dif6 results1, yaxis(2) yline(0, axis(2)) ///
        lcolor(black)  ylab(0(.01).0325)  ysc(range(-.006 .04)
axis(2)) || ///
        line results4 results1, yaxis(1) lcolor(red) || ///
        line results3 results1, yaxis(1) lcolor(blue) ///
         ysc(range(0 1)) ylab(0(.1)1, nogrid) xlab(0(10)120)
xsc(range(0 120)) ///
        ysize(10) xsize(12) graphr(c(white)) ///
        xti("Salary in 1K Euro") scale(1) ///
        yti("Effect of Concussion on Pr(Salary < X)", axis(2)) ///
        yti("{&Phi}(Salary)",  axis(1)) ///
        legend( ///
        label(2 "Effect of concussion (left axis)") ///
        label(3 "Concussion salary distribution (right axis)") ///
        label(4 "Counterfactual salary distribution (right
axis)") ///
        c(1) order(2 3 4) pos(6) size(small) ///
                         symx(4) region(lc(white)))

cap graph drop g1 g2

gr two ///
        line results4 results1, yaxis(1) lcolor(red) || ///
        line results3 results1, yaxis(1) lcolor(blue) ///
         ysc(range(.2 1)) ylab(0.2(.1)1) xlab(0(10)120,
labs(small)) ///
         xsc(range(0 120)) ///
        ysize(10) xsize(10) graphr(c(white)) ///
        xti("Salary in 1K Euro") scale(1) ///
        yti("Cumulative Distribution of Salary",  axis(1)) ///
        legend( ///
        label(1 "Observed Post-Concussion Salary Distribution") ///
        label(2 "Counterfactual No-Concussion Salary
Distribution") ///
        c(1) order(1 2 4) pos(6) size(small) ///
                         symx(4) region(lc(white))) , name(g1)

gr two rline udif6 ldif6 results1, ///
        color(gray) lp(dash) ysc(range(-.002 .035)) || ///
        line dif6 results1,  yline(0) ///
```

```
        lcolor(black)  ylab(0(.01).0325)  ysc(range(-.002 .
035)) ///
        xlab(0(10)120, labs(small)) xsc(range(0 120))      ///
        ysize(10) xsize(10) graphr(c(white)) ///
        xti("Salary in 1K Euro (X)") scale(1) ///
        yti("Effect of Concussion on Pr(Salary < X)") ///
                legend( label(1 "95% CI") ///
        label(2 "Effect of concussion") ///
        c(1) order(2 1) pos(6) size(small) ///
                        symx(4) region(lc(white))) , name(g2)

graph combine g1 g2      ,graphr(c(white))

graph export "[home]\highdef\figure 3.tif",  replace width(1000)




**ESTIMATE SICK LEAVE

forvalue control_time=1/5{
        local end = 2012 // last incident year in data
        if `control_time' ==1    eststo clear

        **build dataset for joint estimate across years
        forvalue count=2003/`end'{
                if `count'==2003{
                        use $data/
sample_control_`count'_`control_time'.dta, clear
                        gen time = `count' //incident year
indicator
                }
                else append using $data/
sample_control_`count'_`control_time'.dta
                replace time = `count' if time ==.

                **exclude individuals in years where they do not
appear in data,
                **due to either death or migration, as well as
periods from when
                **the control group sufer their concussion
                drop if merge ==1 | count > `control_time'-1
        }

        merge m:1 pnr t using $data/temp.dta, keep(1 3) nogen
        replace share = 0 if share==.

        gen female = koen==2

        //build ident, so we can multivariate cluster for
individuals
        //who occur both as control and exposure during the period
(id)
```

```
	bysort pnr time: gen helpx = _n ==1
	gen id= sum(helpx)
	drop helpx


	**Generate educational groups
	qui{
		gen edu =0
		replace edu = 1 if inrange(hffsp,20000000,39000000)
| ///
										(hffsp
>40000000 & hffsp!=.)
	}


	**Calculate number of observations for exposure and control
	count if count==0 & treatment ==1
	local Ntreated = r(N)
	count if count==0 & treatment ==0
	local Ncontrol = r(N)

	**generate concussion variable
	gen treat = inrange(count,0,`control_time'-1) & treatment
==1
	replace treat = time_from_incident if count ==0 & treatment
==1

	**Generate pre-concussion income difference	for
	**use in calculating marginal effects
	sum loenmv if count <0 & treatment ==0
	local control =r(mean)
	sum loenmv if count <0 & treatment ==1
	local treat =r(mean)
	sum loenmv if count>=0 & treatment ==0
	local control_post =r(mean)

	forvalue t=-4/4{
		local n = `t'*-1
		if `t' < -1 gen T_`n' = treatment ==1 & count ==`t'
		if `t' > -1 gen T`t' = treatment ==1 & count ==`t'

	}

	**estimate DiD model on salary
	reghdfe share T*, abs(alder female count time treatment
edu) cl(pnr  id)
	eststo est1_`control_time'
	if `control_time'==1 matrix results = J(5,5,.) // matrix to
capture results
	if `control_time'==1 matrix results_p = J(5,5,.) // matrix
to capture results
```

```
        matrix b = e(b)
        matrix V = e(V)
        local n = `control_time'

        matrix results[`n',1]    = b[1,1] / 7466  // capture beta
results as 1K Euro
        matrix results[`n',2]    = (V[1,1]^.5)/7466       //
capture standard error as 1K Euro
        matrix results[`n',3]    =       b[1,1]/(`control_post'-
(`control'-`treat'))
        matrix results[`n',4]    =       `n'

        gen no_share = share >0 //dummy for no salary


        **Generate pre-concussion probability difference   for
        **use in calculating marginal effects
        sum no_share if count <0 & treatment ==0
        local control =r(mean)
        sum no_share if count <0 & treatment ==1
        local treat =r(mean)
        sum no_share if count>=0 & treatment ==0
        local control_post =r(mean)

        **estimate DiD LP-model on P(salary=0)
        xi: reghdfe no_share T*, abs(alder female count time
treatment edu) cl(pnr  id)
        eststo est2_`control_time'
        matrix b =e(b)   //regression coefficient
        matrix V = e(V)  // standard error^2

        matrix results_p[`n',1]  =       b[1,1]
        matrix results_p[`n',2]  =       V[1,1]^.5
        matrix results_p[`n',3]  =       b[1,1]/(`control_post'-
(`control'-`treat'))
        matrix results_p[`n',4]  =       `n'

}

esttab est1_* using [home]/tables/dynamic_share1.rtf, ///
        replace se(3) b(3) compress nogap star(+ .1 * .05 ** .01
*** .001) ///
        keep(T*)

esttab est2_* using [home]/tables/dynamic_share2.rtf, ///
        replace se(3) b(3) compress nogap star(+ .1 * .05 ** .01
*** .001) ///
        keep(T*)

**ESTIMATE SICK LEAVE

forvalue control_time=1/5{
        local end = 2012 // last incident year in data
        if `control_time' ==1    eststo clear
```

```
	**build dataset for joint estimate across years
	forvalue count=2003/`end'{
		if `count'==2003{
			use $data/
sample_control_`count'_`control_time'.dta, clear
			gen time = `count' //incident year
indicator
		}
		else append using $data/
sample_control_`count'_`control_time'.dta
		replace time = `count' if time ==.

		**exclude individuals in years where they do not
appear in data,
		**due to either death or migration, as well as
periods from when
		**the control group sufer their concussion
		drop if merge ==1 | count > `control_time'-1
	}

	merge m:1 pnr t using $data/temp2.dta, keep(1 3) nogen
	replace syg_barsel_13= 0 if syg_barsel_13==.

	gen female = koen==2

	//build ident, so we can multivariate cluster for
individuals
	//who occur both as control and exposure during the period
(id)

	bysort pnr time: gen helpx = _n ==1
	gen id= sum(helpx)
	drop helpx


	**Generate educational groups
	qui{
		gen edu =0
		replace edu = 1 if inrange(hffsp,20000000,39000000)
| ///
									(hffsp
>40000000 & hffsp!=.)
	}


	**Calculate number of observations for exposure and control
	count if count==0 & treatment ==1
	local Ntreated = r(N)
	count if count==0 & treatment ==0
	local Ncontrol = r(N)

	**generate concussion variable
	gen treat = inrange(count,0,`control_time'-1) & treatment
```

```
==1
        replace treat = time_from_incident if count ==0 & treatment
==1

        **Generate pre-concussion income difference        for
        **use in calculating marginal effects
        sum loenmv if count <0 & treatment ==0
        local control =r(mean)
        sum loenmv if count <0 & treatment ==1
        local treat =r(mean)
        sum loenmv if count>=0 & treatment ==0
        local control_post =r(mean)

        forvalue t=-4/4{
                local n = `t'*-1
                if `t' < -1 gen T_`n' = treatment ==1 & count ==`t'
                if `t' > -1 gen T`t' = treatment ==1 & count ==`t'

        }

        **estimate DiD model on salary
        reghdfe syg_barsel_13 T*, abs(alder female count time
treatment edu) cl(pnr  id)
        eststo est1_`control_time'
        if `control_time'==1 matrix results = J(5,5,.) // matrix to
capture results
        if `control_time'==1 matrix results_p = J(5,5,.) // matrix
to capture results


        matrix b = e(b)
        matrix V = e(V)
        local n = `control_time'

        matrix results[`n',1]    = b[1,1] / 7466  // capture beta
results as 1K Euro
        matrix results[`n',2]    = (V[1,1]^.5)/7466       //
capture standard error as 1K Euro
        matrix results[`n',3]    =       b[1,1]/(`control_post'-
(`control'-`treat'))
        matrix results[`n',4]    =       `n'




        **Generate pre-concussion probability difference   for
        **use in calculating marginal effects

        **estimate DiD LP-model on P(salary=0)
        xi: reghdfe kont_dag T*, abs(alder female count time
treatment edu) cl(pnr  id)
        eststo est2_`control_time'
        matrix b =e(b)   //regression coefficient
        matrix V = e(V)  // standard error^2
```

```
        matrix results_p[`n',1]  =       b[1,1]
        matrix results_p[`n',2]  =       V[1,1]^.5
        *matrix results_p[`n',3] =       b[1,1]/(`control_post'-
(`control'-`treat'))
        matrix results_p[`n',4]  =       `n'

}

esttab est1_* using [home]/tables/dynamic_sickpay1.rtf, ///
        replace se(3) b(3) compress nogap star(+ .1 * .05 ** .01
*** .001) ///
        keep(T*)

esttab est2_* using [home]/tables/dynamic_welfare2.rtf, ///
        replace se(3) b(3) compress nogap star(+ .1 * .05 ** .01
*** .001) ///
        keep(T*)
```

For peer review only

Supplemental results

For peer review only

For peer review only - http://bmjopen.bmj.com/site/about/guidelines.xhtml

Table S1. **Test of parallel trends assumption pre-exposure incident against each control group separately using eq. S3 in supplementary methods. Separate exposure dummies for all time periods (except the year prior to exposure, which serves as reference period).**

Time to exposure group's concussion (exposure)	Δ=1 Est (S.E.) p-value	Δ=2 Est (S.E.) p-value	Δ=3 Est (S.E.) p-value	Δ=4 Est (S.E.) p-value	Δ=5 Est (S.E.) p-value
**Exposure-4y**	-0.368<br>(0.226)<br>p=.104	0.046<br>(0.363)<br>p=.900	0.120<br>(0.362)<br>p=.741	0.159<br>(0.313)<br>p=.612	0.042<br>(0.329)<br>p=.899
**Exposure-3y**	-0.094<br>(0.317)<br>p=.768	0.227<br>(0.510)<br>p=.656	0.167<br>(0.354)<br>p=.637	0.537<br>(0.372)<br>p=.148	0.113<br>(0.393)<br>p=.774
**Exposure-2y**	-0.548<br>(0.312)<br>p=.079	-0.082<br>(0.347)<br>p=.812	-0.163<br>(0.236)<br>p=.491	-0.124<br>(0.247)<br>p=.617	0.082<br>(0.250)<br>p=.744
**Exposure-1y**	Ref.	Ref.	Ref.	Ref.	Ref.
**N*T**	284115	273725	266120	260647	256337

Note: The table shows test for differences in pre-exposure trends between exposure and control group model using interactions between pre-exposure time dummies and the exposure indicator. There is no indication of substantial or significant pre-exposure differences in salary trajectories between exposure group and any of the control groups.

For peer review only

Table S2. **Effect of concussion on different labor market outcome parameters using separate exposure dummies for all time periods (except the year prior to exposure, which serves as reference period): In this exploratory analysis, the exposure group is compared to the control group Δ=5, which suffers a concussion five years after the exposure group. Outcomes include annual salaried income (annual salary), total annual income (total income), annual sick leave benefits received (sick leave benefits) as well as a binary indicator of employment (probability of employment). Monetary outcomes were measured at 2015-level in € 1,000.**

Time to exposure group's concussion (exposure)	Annual Salary Est. (S.E.) p-value	Total income Est. (S.E.) p-value	Sick leave benefits Est. (S.E.) p-value	Probability of employment Est. (S.E.) p-value
**Exposure-4y**	0.012	0.164	0.035	0.001
	(0.212)	(0.173)	(0.036)	(0.004)
	p=.954	p=.343	p=.320	p=.803
**Exposure-3y**	0.059	0.305	0.022	-0.001
	(0.252)	(0.233)	(0.034)	(0.003)
	p=.814	p=.190	p=.529	p=.739
**Exposure-2y**	0.043	0.122	0.002	0.001
	(0.160)	(0.147)	(0.029)	(0.003)
	p=.788	p=.405	p=.946	p=.739
**Exposure-1y**				
**Exposure**	-0.611	-0.338	0.166	-0.003
	(0.168)	(0.140)	(0.030)	(0.003)
	p<.001	0.016	p<.001	p=.317
**Exposure+1y**	-1.389	-0.608	0.288	-0.020
	(0.209)	(0.162)	(0.039)	(0.003)
	p<.001	p<.001	p<.001	p<.001
**Exposure+2y**	-1.568	-0.847	0.132	-0.023
	(0.261)	(0.231)	(0.039)	(0.004)
	p<.001	p<.001	p=.001	p<.001
**Exposure+3y**	-1.393	-0.497	0.031	-0.022
	(0.246)	(0.219)	(0.040)	(0.004)
	p<.001	p=.023	p=.432	p<.001
**Exposure+4y**	-1.319	-0.499	-0.076	-0.018
	(0.253)	(0.218)	(0.042)	(0.004)
	p<.001	p=.022	p=.075	p<.001
**N*T**	577762	577758	577872	577872

Note: Annual salary include all income from salary and employee fringe benefits, employee stock options, employer paid sick leave, net gains (including interests and capital gains) from own companies. Total income includes all income absent wealth. Sick leave includes only public health benefits (sick leave and paternity leave). Employment is a binary indicator measured last week of November for each year. Results obtained from estimations following Eq. (1). Models include controls for high school diploma, gender, age, and observation year. Results obtained using reghdfe in Stata. Total number of observations (N*T) differ slightly between outcomes because all income information is not available for all observation all years.

Source: Own calculations on data from Statistics Denmark.

For peer review only - http://bmjopen.bmj.com/site/about/guidelines.xhtml

Table S3. **Demographic factors for exposure group and control groups (Δ=1, 2, 3, 4, 5) averaged over the 5 years leading up to the concussion event in each of the groups. Factors include patient age (in years), share of sample female (1=100% female), and share of individuals with at least a high school degree (1=100%).**

		Exposure	Control, Δ=1	Control, Δ=2	Control, Δ=3	Control, Δ=4	Control, Δ=5
**Pr(Female=1)**	Mean	.430	.438	.447	.458	.464	.473
	S.D.	(.495)	(.496)	(.497)	(.498)	(.499)	(.499)
	p-value		.030	<.001	<.001	<.001	<.001
**Age**	Mean	36.899	37.354	37.754	38.065	38.343	38.592
	S.D.	(11.856)	(11.857)	(11.718)	(11.630)	(11.584)	(11.491)
	p-value		<.001	<.001	<.001	<.001	<.001
**Pr(High school=1)**	Mean	.624	.632	.640	.646	.653	.660
	S.D.	(.484)	(.482)	(.480)	(.478)	(.476)	(.474)
	p-value		.026	<.001	<.001	<.001	<.001
**Total individuals**		37848	34551	31851	29922	28580	27484

Note: S.D.: Standard deviation. P-values calculated using two-sided t-tests. All test performed between exposure group and each control group separately.

For peer review only

Figure S1. **Unnormalized Average Salary for Treatment and Control Groups Measured in 1K € at 2015-levels**

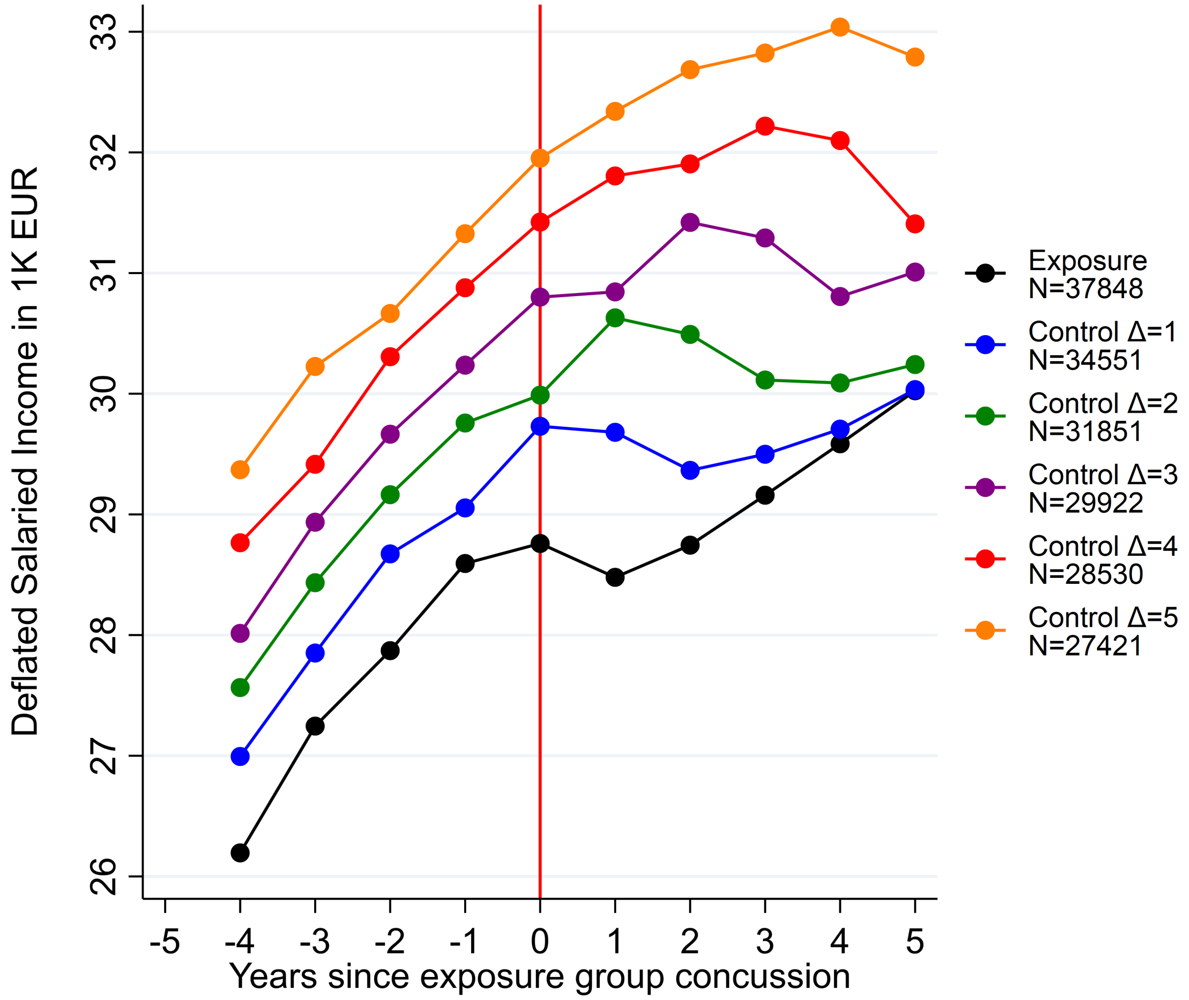

Note: Salary of the exposure group compared to salary of the 5 control groups, who experienced their concussions Δ={1, 2, 3, 4, and 5} years later than the exposure group. Salary progression is shown for the 5 years before and the 5 years after the exposure group suffered a concussion event. Table S1 demonstrates that the trends for salary progression pre-exposure incident are parallel between exposure group and each control group.

Figure S2. (Left Panel)The Cumulative Distribution for Total Income Post-Treatment among the Treatment Group and Their Counterfactual, and (Right Panel) the Difference between the Two CDFs Expressed as the Effect of Concussion on the Probability of Total Income Below that Income-Level Expressed on the X-Axis following Exposure Event.

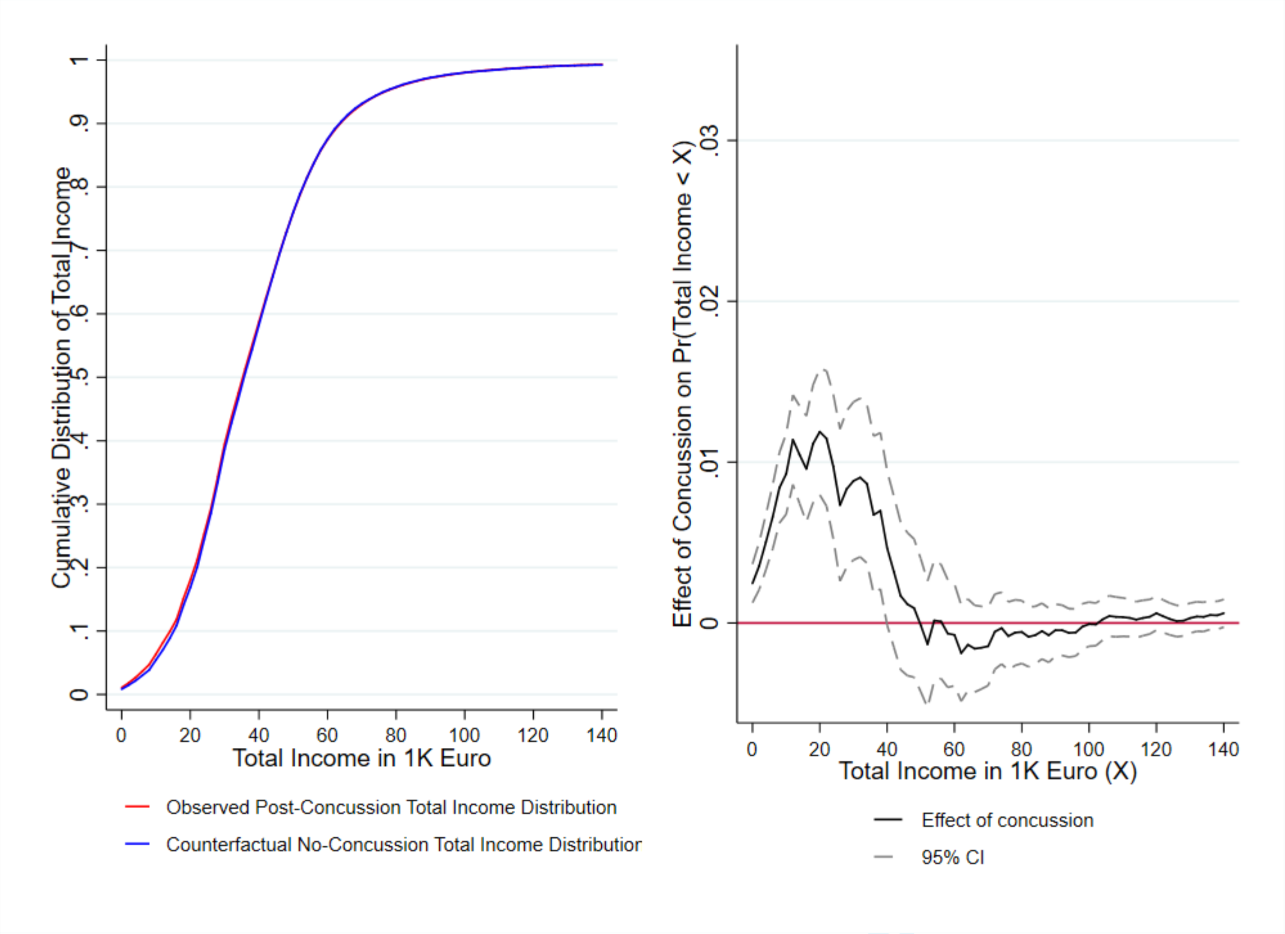

Note: The figure shows the observed cumulative salary distribution following a concussion (red) and the expected counterfactual salary distribution absent the concussion (blue). The black line shows the difference between the observed and the counterfactual distribution, and the grey dash lines show the 95 % confidence interval. The bell-shape of the difference between the two distributions as the total income increase from 0 to 40,000 € indicates that the main part of the effect of concussions on total incomes is driven by low-income people shifting total income downwards following concussion, but not going to total income equal to zero.

Figure S3. Percentage Effect of Concussion on Relative Salary Across Age Groups.

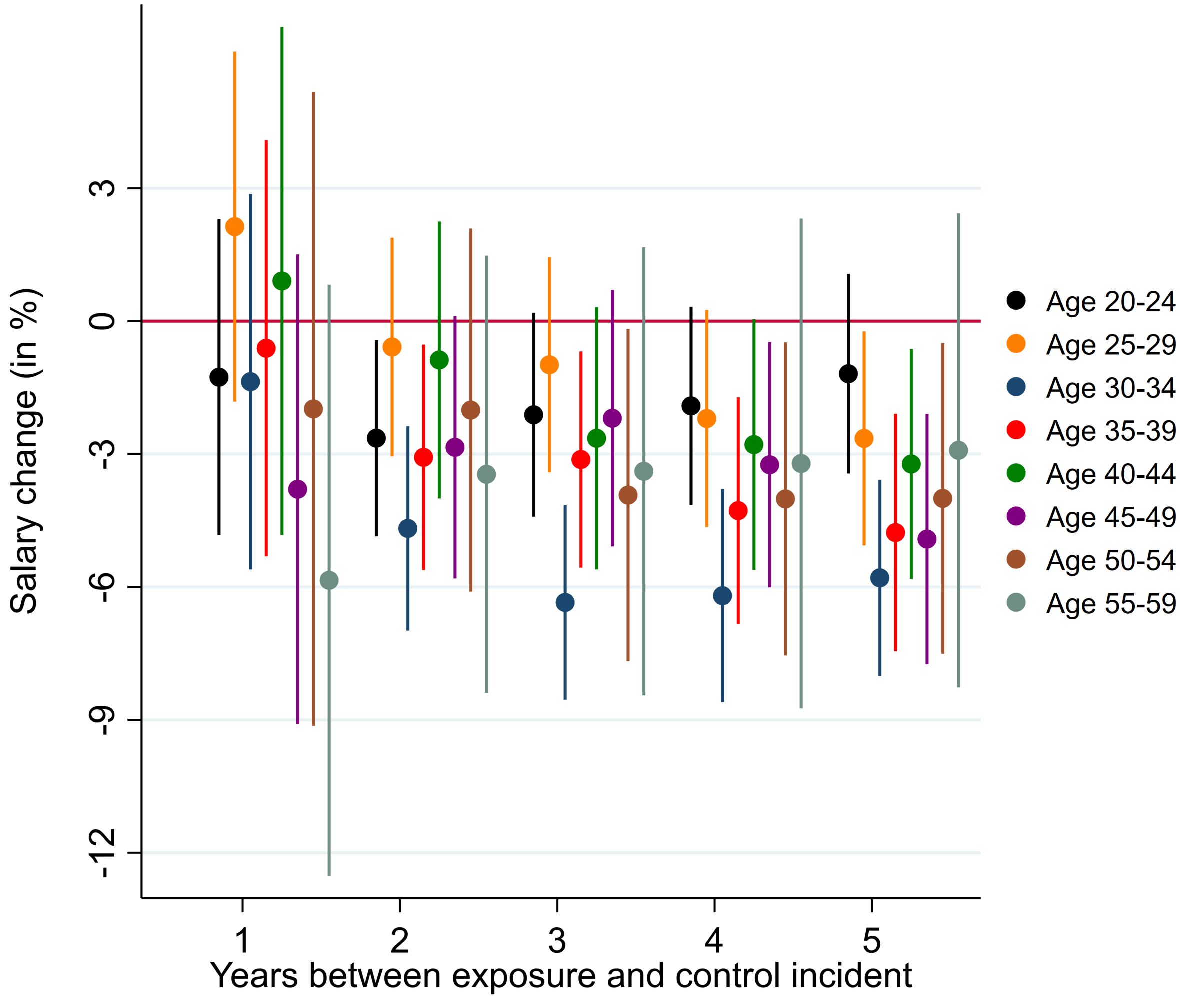

Note: Relative salary drop of the exposure group compared to the control groups, who suffer a concussion 1, 2, 3, 4, and 5 years after the exposure group. Relative salary changes are shown across different age groups. Graph shows parameter estimates and 95% CI.

For peer review only - http://bmjopen.bmj.com/site/about/guidelines.xhtml

Figure S4. **Percentage Effect of Concussion on Relative Salary Across High School Completion.**

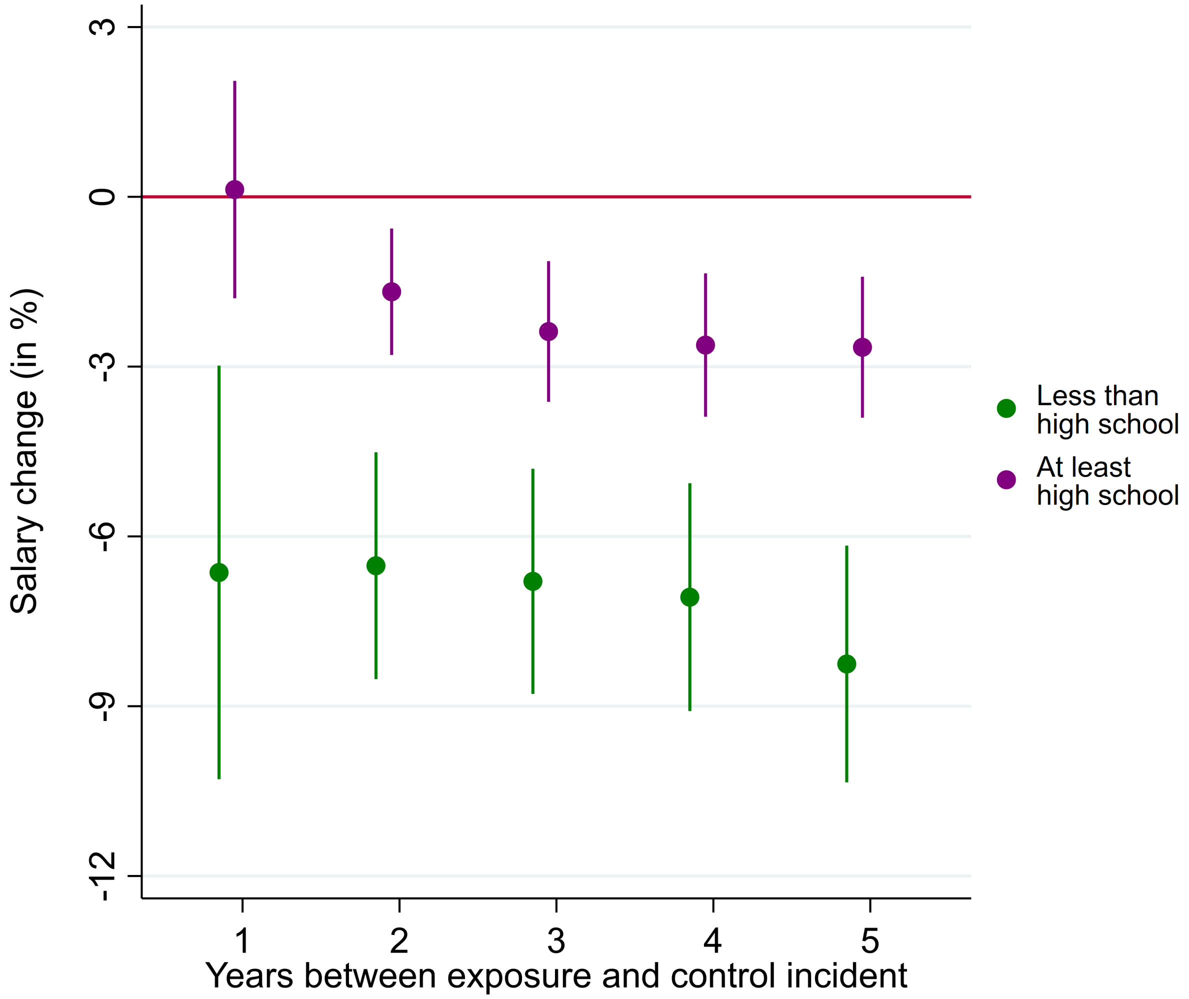

Note: Relative salary drop of the exposure group compared to the control groups, who suffer a concussion 1, 2, 3, 4, and 5 years after the exposure group. Relative salary changes are shown across whether individuals had obtained at least a high school diploma (ISCED > 2). Graph shows parameter estimates and 95% CI.

only

Figure S5. **Percentage Effect of Concussion on Relative Salary Across Gender.**

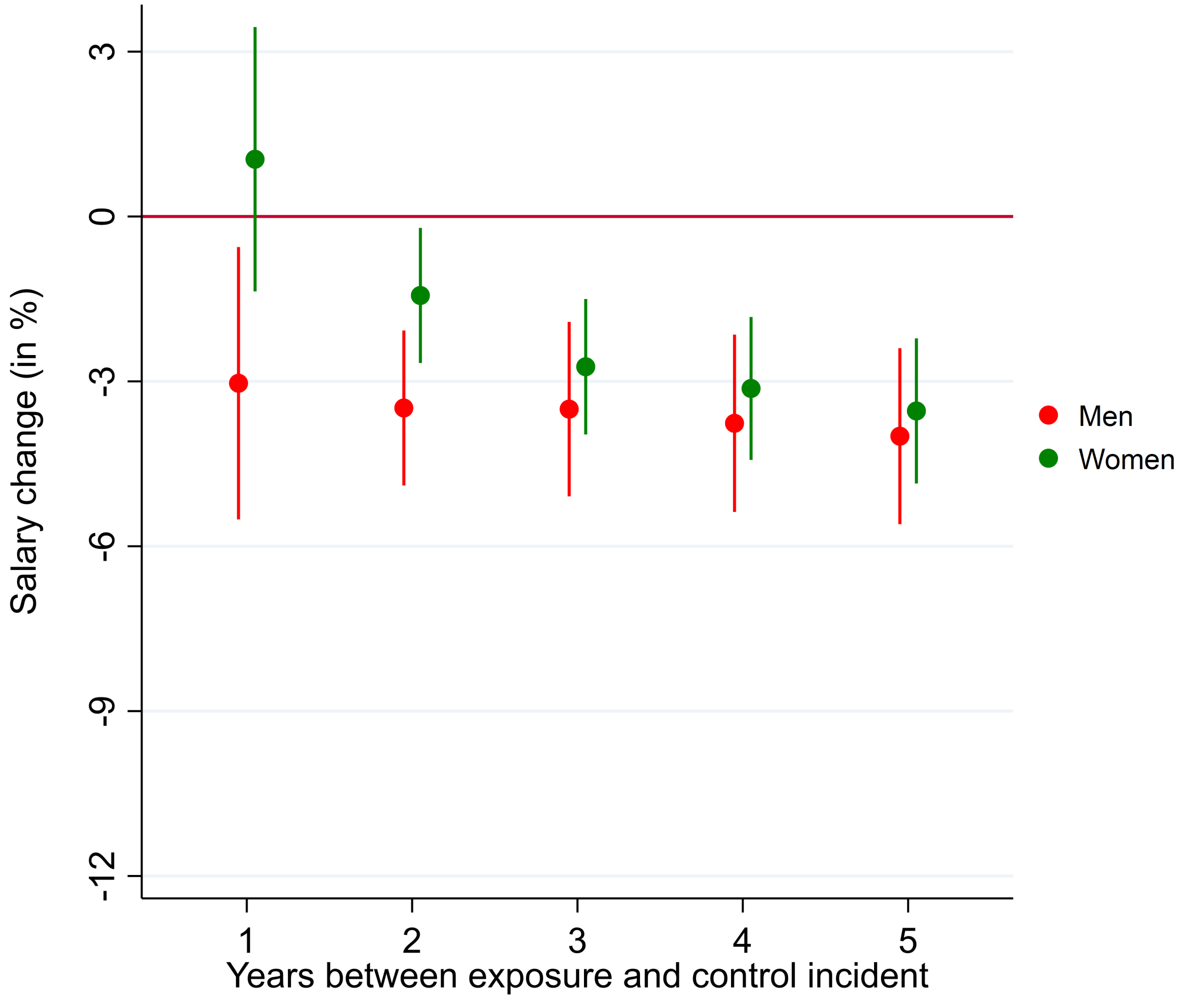

Note: Relative salary drop of the exposure group compared to the control groups, who suffer a concussion 1, 2, 3, 4, and 5 years after the exposure group. Relative salary changes are shown across gender. Graph shows parameter estimates and 95% CI.

For peer review only - http://bmjopen.bmj.com/site/about/guidelines.xhtml

Figure S6. **Effect of Concussion on Absolute Salary in 1K Euro Across Age groups.**

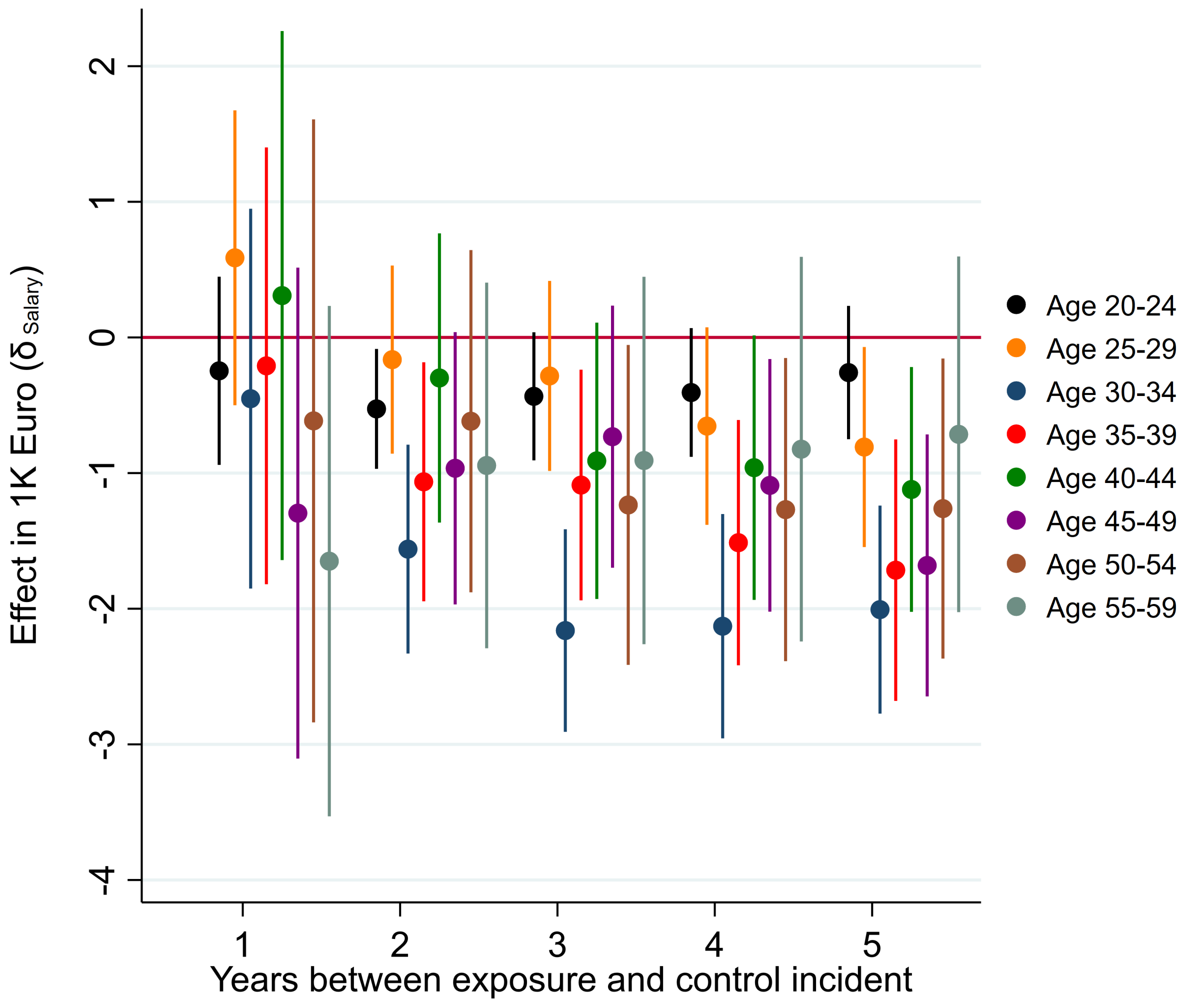

Note: Absolute salary drop of the exposure group compared to the control groups, who suffer a concussion 1, 2, 3, 4, and 5 years after the exposure group. Absolute salary changes are shown across different age groups. Graph shows parameter estimates and 95% CI.

For peer review only - http://bmjopen.bmj.com/site/about/guidelines.xhtml

Figure S7. **Effect of Concussion on Absolute Salary in 1K Euro Across Education.**

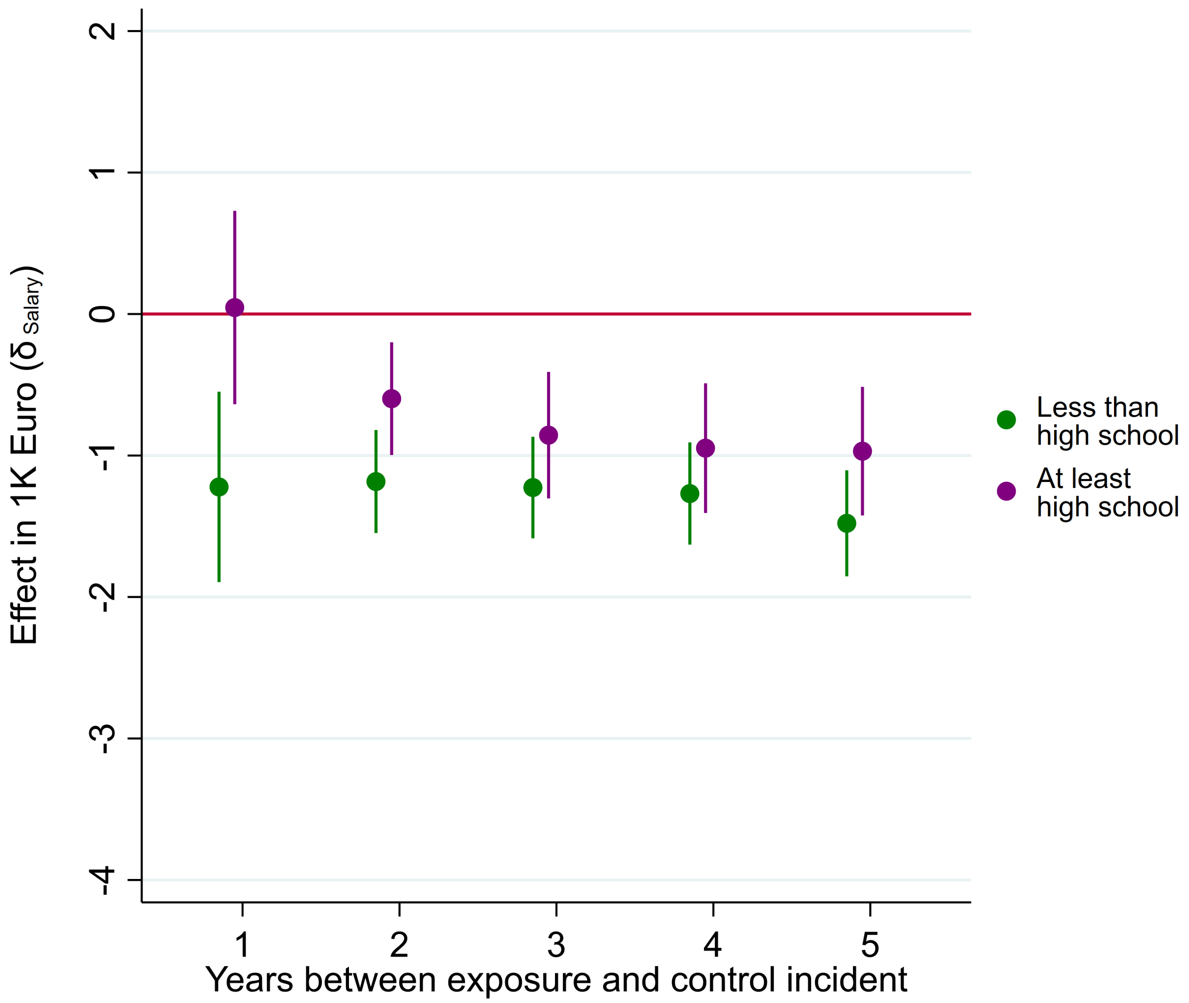

Note: Absolute salary drop of the exposure group compared to the control groups, who suffer a concussion 1, 2, 3, 4, and 5 years after the exposure group. Absolute changes in salary are shown across whether individuals had obtained at least a high school diploma (ISCED > 2). Graph shows parameter estimates and 95% CI.

Figure S8. **Effect of Concussion on Absolute Salary in 1K Euro Across Gender.**

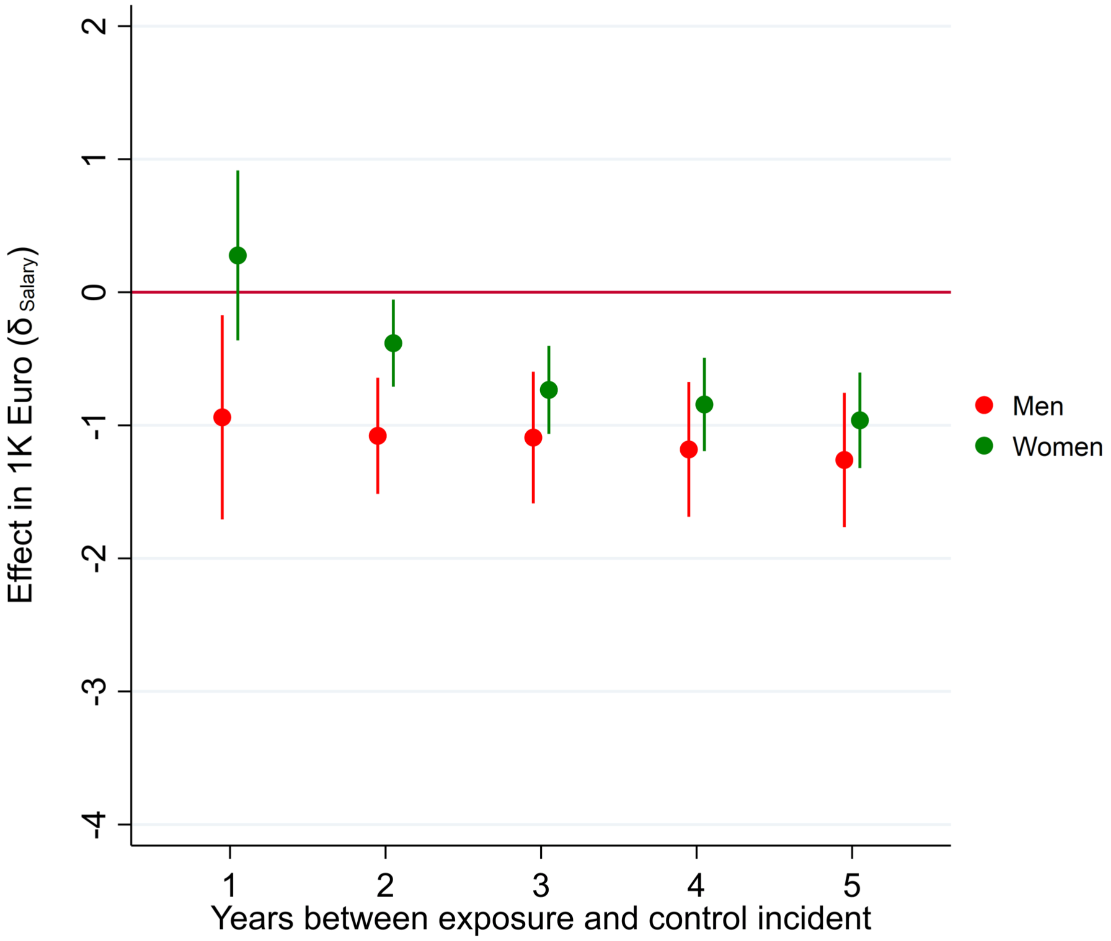

Note: Absolute salary drop of the exposure group compared to the control groups, who suffer a concussion 1, 2, 3, 4, and 5 years after the exposure group. Absolute changes in salary are shown across gender. Graph shows parameter estimates and 95% CI.

The RECORD statement – checklist of items, extended from the STROBE statement, that should be reported in observational studies using routinely collected health data.

	Item No.	STROBE items	Location in manuscript where items are reported	RECORD items	Location in manuscript where items are reported
**Title and abstract**					
	1	(a) Indicate the study's design with a commonly used term in the title or the abstract (b) Provide in the abstract an informative and balanced summary of what was done and what was found	title<br>abstract	RECORD 1.1: The type of data used should be specified in the title or abstract. When possible, the name of the databases used should be included.<br><br>RECORD 1.2: If applicable, the geographic region and timeframe within which the study took place should be reported in the title or abstract.<br><br>RECORD 1.3: If linkage between databases was conducted for the study, this should be clearly stated in the title or abstract.	title<br>abstract<br><br>title<br>abstract<br><br>abstract
**Introduction**					
Background rationale	2	Explain the scientific background and rationale for the investigation being reported	abstract<br>introduction		
Objectives	3	State specific objectives, including any prespecified hypotheses	introduction		
**Methods**					
Study Design	4	Present key elements of study design early in the paper	introduction		
Setting	5	Describe the setting, locations, and relevant dates, including periods of recruitment, exposure, follow-up, and data collection	materials and methods		

For peer review only - http://bmjopen.bmj.com/site/about/guidelines.xhtml

Participants	6	*(a) Cohort study* - Give the eligibility criteria, and the sources and methods of selection of participants. Describe methods of follow-up<br>*Case-control study* - Give the eligibility criteria, and the sources and methods of case ascertainment and control selection. Give the rationale for the choice of cases and controls<br>*Cross-sectional study* - Give the eligibility criteria, and the sources and methods of selection of participants<br><br>*(b) Cohort study* - For matched studies, give matching criteria and number of exposed and unexposed<br>*Case-control study* - For matched studies, give matching criteria and the number of controls per case	materials and methods	RECORD 6.1: The methods of study population selection (such as codes or algorithms used to identify subjects) should be listed in detail. If this is not possible, an explanation should be provided.<br><br>RECORD 6.2: Any validation studies of the codes or algorithms used to select the population should be referenced. If validation was conducted for this study and not published elsewhere, detailed methods and results should be provided.<br><br>RECORD 6.3: If the study involved linkage of databases, consider use of a flow diagram or other graphical display to demonstrate the data linkage process, including the number of individuals with linked data at each stage.	materials and methods<br><br>materials and methods<br><br>not included
Variables	7	Clearly define all outcomes, exposures, predictors, potential confounders, and effect modifiers. Give diagnostic criteria, if applicable.	materials and methods<br>main text	RECORD 7.1: A complete list of codes and algorithms used to classify exposures, outcomes, confounders, and effect modifiers should be provided. If these cannot be reported, an explanation should be provided.	materials and methods
Data sources/ measurement	8	For each variable of interest, give sources of data and details of methods of assessment (measurement).<br>Describe comparability of assessment methods if there is more than one group	materials and methods		

For peer review only

Bias	9	Describe any efforts to address potential sources of bias	materials and methods and results		
Study size	10	Explain how the study size was arrived at	materials and methods		
Quantitative variables	11	Explain how quantitative variables were handled in the analyses. If applicable, describe which groupings were chosen, and why	materials and methods		
Statistical methods	12	(a) Describe all statistical methods, including those used to control for confounding<br>(b) Describe any methods used to examine subgroups and interactions<br>(c) Explain how missing data were addressed<br>(d) *Cohort study* - If applicable, explain how loss to follow-up was addressed<br>*Case-control study* - If applicable, explain how matching of cases and controls was addressed<br>*Cross-sectional study* - If applicable, describe analytical methods taking account of sampling strategy<br>(e) Describe any sensitivity analyses	a) materials and methods<br>b) materials and methods<br>c) materials and methods<br>d-e) NA		
Data access and cleaning methods		..		RECORD 12.1: Authors should describe the extent to which the investigators had access to the database population used to create the study population.	materials and methods<br><br>materials and methods

				RECORD 12.2: Authors should provide information on the data cleaning methods used in the study.	
Linkage		..		RECORD 12.3: State whether the study included person-level, institutional-level, or other data linkage across two or more databases. The methods of linkage and methods of linkage quality evaluation should be provided.	materials and methods
**Results**					
Participants	13	(a) Report the numbers of individuals at each stage of the study (*e.g.*, numbers potentially eligible, examined for eligibility, confirmed eligible, included in the study, completing follow-up, and analysed)<br>(b) Give reasons for non-participation at each stage.<br>(c) Consider use of a flow diagram	(a-c) materials and methods	RECORD 13.1: Describe in detail the selection of the persons included in the study (*i.e.,* study population selection) including filtering based on data quality, data availability and linkage. The selection of included persons can be described in the text and/or by means of the study flow diagram.	materials and methods
Descriptive data	14	(a) Give characteristics of study participants (*e.g.*, demographic, clinical, social) and information on exposures and potential confounders<br>(b) Indicate the number of participants with missing data for each variable of interest<br>(c) *Cohort study* - summarise follow-up time (*e.g.*, average and total amount)	a) materials and methods and Supplementary Table S3<br><br>b) materials and methods,Table 1<br><br>c) materials and methods		
Outcome data	15	*Cohort study* - Report numbers of outcome events or summary measures over time<br>*Case-control study* - Report numbers in each exposure	materials and methodsand Supplementary Table S3, results		

For peer review only

		category, or summary measures of exposure<br>*Cross-sectional study* - Report numbers of outcome events or summary measures			
Main results	16	(a) Give unadjusted estimates and, if applicable, confounder-adjusted estimates and their precision (e.g., 95% confidence interval). Make clear which confounders were adjusted for and why they were included<br>(b) Report category boundaries when continuous variables were categorized<br>(c) If relevant, consider translating estimates of relative risk into absolute risk for a meaningful time period	a) results<br>b) results<br>c) results		
Other analyses	17	Report other analyses done—e.g., analyses of subgroups and interactions, and sensitivity analyses	results		
**Discussion**					
Key results	18	Summarise key results with reference to study objectives	results and discussion		
Limitations	19	Discuss limitations of the study, taking into account sources of potential bias or imprecision. Discuss both direction and magnitude of any potential bias	results and discussion	RECORD 19.1: Discuss the implications of using data that were not created or collected to answer the specific research question(s). Include discussion of misclassification bias, unmeasured confounding, missing data, and changing eligibility over time, as they pertain to the study being reported.	NA
Interpretation	20	Give a cautious overall interpretation of results considering objectives,	discussion		

For peer review only - http://bmjopen.bmj.com/site/about/guidelines.xhtml

		limitations, multiplicity of analyses, results from similar studies, and other relevant evidence			
Generalisability	21	Discuss the generalisability (external validity) of the study results	discussion		
**Other Information**					
Funding	22	Give the source of funding and the role of the funders for the present study and, if applicable, for the original study on which the present article is based	Title page		
Accessibility of protocol, raw data, and programming code		..	Reference to supplementary data throughout the text	RECORD 22.1: Authors should provide information on how to access any supplemental information such as the study protocol, raw data, or programming code.	materials and methods

*Reference: Benchimol EI, Smeeth L, Guttmann A, Harron K, Moher D, Petersen I, Sørensen HT, von Elm E, Langan SM, the RECORD Working Committee. The REporting of studies Conducted using Observational Routinely-collected health Data (RECORD) Statement. *PLoS Medicine* 2015; in press.

*Checklist is protected under Creative Commons Attribution (CC BY) license.

For peer review only

The Effect of Concussion on Salary and Employment-A Population-Based Event Time Study using a Quasi-Experimental Design

Journal:	*BMJ Open*
Manuscript ID	bmjopen-2020-038161.R2
Article Type:	Original research
Date Submitted by the Author:	10-Sep-2020
Complete List of Authors:	Fallesen, Peter; Stockholm University, Swedish Institute for Social Research<br>Campos, Benito; Heidelberg University Hospital Department of Neurosurgery; Technical University of Denmark, Department of Health Tech
**Primary Subject Heading**:	Public health
Secondary Subject Heading:	Neurology
Keywords:	HEALTH ECONOMICS, Neurosurgery < SURGERY, Trauma management < ORTHOPAEDIC & TRAUMA SURGERY

SCHOLARONE™
Manuscripts

I, the Submitting Author has the right to grant and does grant on behalf of all authors of the Work (as defined in the below author licence), an exclusive licence and/or a non-exclusive licence for contributions from authors who are: i) UK Crown employees; ii) where BMJ has agreed a CC-BY licence shall apply, and/or iii) in accordance with the terms applicable for US Federal Government officers or employees acting as part of their official duties; on a worldwide, perpetual, irrevocable, royalty-free basis to BMJ Publishing Group Ltd ("BMJ") its licensees and where the relevant Journal is co-owned by BMJ to the co-owners of the Journal, to publish the Work in this journal and any other BMJ products and to exploit all rights, as set out in our licence.

The Submitting Author accepts and understands that any supply made under these terms is made by BMJ to the Submitting Author unless you are acting as an employee on behalf of your employer or a postgraduate student of an affiliated institution which is paying any applicable article publishing charge ("APC") for Open Access articles. Where the Submitting Author wishes to make the Work available on an Open Access basis (and intends to pay the relevant APC), the terms of reuse of such Open Access shall be governed by a Creative Commons licence – details of these licences and which Creative Commons licence will apply to this Work are set out in our licence referred to above.

Other than as permitted in any relevant BMJ Author's Self Archiving Policies, I confirm this Work has not been accepted for publication elsewhere, is not being considered for publication elsewhere and does not duplicate material already published. I confirm all authors consent to publication of this Work and authorise the granting of this licence.

For peer review only

The Effect of Concussion on Salary and Employment-A Population-Based Event Time Study using a Quasi-Experimental Design

[1,2]Peter Fallesen, PhD, [3,4]Benito Campos, M.D.

[1]Swedish Institute for Social Research, Stockholm University, SE-106 91 Stockholm, Sweden
[2]ROCKWOOL Foundation, Ny Kongensgade 6, DK-1472 Copenhagen C, Denmark
[3]Department of Neurosurgery, University of Heidelberg, INF400, 69120 Heidelberg, Germany
[4]Department of Health Tech, Technical University of Denmark, DK-2800 Kongens Lyngby, Denmark

Address of Correspondence: Peter Fallesen, Swedish Institute for Social Research, Stockholm University, SE-106 91 Stockholm, Sweden, peter.fallesen@sofi.su.se, +4561333184

Keywords: administrative data; concussion; income; productivity

Abstract word count: 245

Text word count: 3989 (excluding references and figure/table legends)

Number of references: 21

Number of tables and/or figures: 2 tables and 4 figures

Number of videos: 0

For peer review only

Abstract

Objective: Concussions are the most frequent traumatic brain injuries. Yet, the socio-economic impact of concussions remains unclear. Socio-economic effects of concussions on working age adults were studied on a population scale.

Design: This population-based, event time study uses administrative data as well as hospital and emergency room records for the population of Denmark.

Setting: We study all Danish patients, aged 20-59 y, who were treated at a public hospital or at an emergency room between 2003-2017 after suffering a concussion without other intracranial or extracranial injuries (n=55,424 unique individuals). None of the patients had a prior diagnosis of intra- or extracranial injuries within the past ten years leading up to the incident.

Primary and Secondary Outcome Measures: As primary endpoint, we investigate the mean effect of concussion on annual salaried income within a five-year period after trauma. In an exploratory analysis, we study whether the potential impact of concussion on annual salaried income is driven by patient age, education, or economic cycle.

Results: Concussion was associated with an average change in annual salary income of -1,223€ (95% CI, -1,540€; -905€, p<.001) corresponding to a salary change of -4.2 % (95% CI, -5.2 %; -3.1 %). People between 30-39 y and those without high school degrees suffered the largest salary decreases. Affected individuals leaving the workforce drove the main part of the decrease. Absolute annual effect sizes were countercyclical to the unemployment rate.

Conclusions: Concussions have a large and long-lasting impact on salary and employment of working-age adults on a nationwide scale.

For peer review only

Strengths and limitations of this study

- Natural experiments used to obtain plausible causal effects between concussion and salary/employment.
- Large, population-based sample with multiple data layers.
- Analysis includes how economic cycles affect outcome measures.
- Data only captures concussions registered in ERs and hospitals.
- Because concussions do not occur at random, causal estimate relies on stronger assumptions than for a randomized control trial.

For peer review only

For peer review only - http://bmjopen.bmj.com/site/about/guidelines.xhtml

Introduction

Concussions are by far the most frequently occurring intracranial injuries affecting approximately 450 to 600 per 100,000 people every year[1] . Immediate symptoms may last for days or weeks. Further, Danish cohort data[2] indicates that 10-15% of patients diagnosed with concussion suffer from long-term symptoms such as headache, fatigue, and intolerance to stress, whereas other studies place the upper bound as high as 30%[3,4]. Clinical practice has encouraged patients to restrict social, mental, and physical activity in the weeks following a concussion (see[5] for review), although prolonged inactivity may prolong symptoms. Thus, symptoms, comorbidities, and suggested treatment are associated with short- to long-term absence from work and lower productivity.

Yet, the causal effect of concussion on economic burdens for individuals and society through decreased labour market activity has not been identified. First, concussion is a sudden incident and thus not amenable to prospective study nor randomization. Cohort and case-control studies[6–10] provide some valuable evidence on employment and labour market outcomes among those who suffered concussions but are prone to selection bias. Individuals at high risk of concussions may differ on unobserved characteristics (e.g., risk aversion, routine activities) from those at low risk. People who are more likely to suffer concussions may also, on average, have more precarious or unstable employment trajectories prior to the incident, which may further bias prospective studies. Given the high incidence rate of concussion, even small losses of productivity and discrete drops in employment would have a significant socioeconomic impact and thus, it would require large patient cohorts with suitable controls to grasp the full socioeconomic impact of concussions. Thus, absent the possibility of randomization, using a

For peer review only

natural or quasi-experimental design is the only likely option to parse out the *causal* effect of concussions on labour market activity.[11]

We examine how concussions affect salary and employment of working age individuals in Denmark, a representative north-European industrial nation with a strong welfare state and a flexible labour market. We use administrative longitudinal data linked to hospital and emergency room diagnostic data on all Danes, who received a primary diagnosis of concussion between 2003 and 2017. To address the problem of unmeasured bias between those that do and do not experience a concussion, we use a quasi-experimental event-study approach[12,13] where we compare similar individuals, who experienced their concussions at different time points. Under mild assumptions of parallel trends in wage progression prior to concussion and random timing of concussion event within a five-year time frame, the approach recovers a robust estimation of the effect of concussion on annual salary and employment status.

For peer review only

For peer review only - http://bmjopen.bmj.com/site/about/guidelines.xhtml

Material & Methods

Data Sources and Sample Construction

Concussion data originates from the Danish National Patient Registry (DNPR) (see[14] for description). DNPR is published annually and holds information on all hospitalizations at public hospitals in Denmark since 1977, on all emergency room visits and outpatient treatments at public hospitals since 1994, and almost all private hospital treatments since 2003. With one single, short-lived exception, private hospitals do not operate emergency rooms in Denmark. Since 2003, the data cover 95 % of all treatments at private hospitals[14], yet only 13 concussion were diagnosed in private hospital settings throughout the period covered by the data.

The combined exposure and control cohort includes all Danes aged 20-59 y, who received a primary diagnosis of concussion (ICD-10 code S06.0, ICD-8 code N850) between 2003 and 2017 and did not sustain any kind of additional intracranial or extracranial injury. Individuals who regularly engage in activities associated with a high risk of sustaining multiple concussions may differ from the average concussion patient and would likely be over-represented in the exposure sample. To avoid such potential bias, all individuals who were diagnosed with any kind of brain trauma during a ten-year period prior to the concussion event were excluded. Altogether, the study included a cohort of 55,424 individuals. Only attrition is through mortality and out-migration, and out-migrated or deceased patients with missing spells in the follow up period is excluded in those periods.

As a measure of productivity, a price-index deflated annual salaried income was used. Salary information comes from Statistics Denmark's Income Statistics database. The database

For peer review only - http://bmjopen.bmj.com/site/about/guidelines.xhtml

includes all declared annual incomes including income from self-employment. The Danish Tax Authorities supply the data to Statistics Denmark. Overall accuracy is considered very good.[15] Table 1 reports number of observations for the samples and number of observations with missing salary information. As evident, only between 0.01 to 0.02 percent of observations across exposure and control groups have missing salary information. These observations were disregarded in the main analysis. Through social security numbers, information on salaried income were linked to records on diagnosed concussions. Further, information on high school or equivalent level degree at time of concussion was obtained using the Danish Education Database. The Danish Population Database provided demographic information on age and gender for all respondents. Since the data used in the study come from de-identified administrative registers that Statistics Denmark makes available for research purposes for approved institutions, no approval from an ethics committee was needed to carry out the study. The research was carried out as part of project no. 706630 approved by Statistics Denmark. Statistical analysis was carried out using Stata MP 15.1.

Quasi-experimental design

The study used a quasi-experimental, difference-in-differences event time approach previously described in a health setting by Dobkin et al.[12] The approach compare two groups of individuals from the same cohort, where both groups experience concussions, but at two different time points (t_c,t_c+Δ). Specifically, the sample of 55,496 individuals was divided into six different subgroups: i) The *exposure group*, which includes all patients, who suffered their concussion during the period 2003-2012 (n=37,848) and ii) five *control groups*, which comprise

For peer review only - http://bmjopen.bmj.com/site/about/guidelines.xhtml

patients who experienced their concussions Δ={1 (n=34,551), 2 (n=31,851), 3 (n=29,922), 4 (n=28,530), and 5 (n=27,421)} years later than the exposure group and did not experience any kind of brain injury in the 10+Δ years before the concussion event (note that the design allows individuals to both be part of the exposure and control group). The model is built on the assumption that the exact timing of a concussion is random for small enough values of Δ, and on the additional assumption that the exposure and the control groups would have displayed parallel trends in salary if the control group had not suffered a concussion at t_c (i.e., assuming that control and exposure group(s) would have continued to show similar trends in salaried earnings had the exposure group not experienced concussions). Table 1 show the number of patients in the exposure group and the five control groups for each year relative to exposure group's concussion incident. Using multiple comparison groups makes it possible to gage the validity of the assumption that the exact timing of a concussion is random for small enough sizes of Δ

To estimate the impact of concussion on labour market outcomes, the analysis focuses on the change in annual salary as the primary outcome, and, in further exploratory analyses, studies additional outcomes such as income from health-related benefits, income from welfare benefits, and employment rates. The data are nested within a three-level structure: Exposure or control group *g*, which includes individuals *I*, at times to exposure-groups concussion incident *t*. First, a standard difference in differences model for each separate control group Δ={1, 2, 3, 4, and 5} is estimated using ordinary least squares:

$$Salary_{git} = \beta_0 + \gamma Exposure_g + \theta Post_t + \delta Post \times Exposure_{git} + \boldsymbol{X_i \beta} + \sum_{Age=20}^{59+\Delta} I(Age)\eta_{Age} + \sum_{Year=1999}^{2012} I(Year)\eta_{Year} + \epsilon_{git} \quad (1)$$

where $Salary_{git}$ measures annual salaried income deflated to 2015-level; $Exposure_g$ indicates whether the observation belongs to the exposure or control group; $Post_t$ captures the period after the exposure group's concussion occurred; $Post_t \times Exposure_{git}$ captures the effect concussion, measured as share of year t ≥ 0 affected by concussion (i.e., for year of incident exposure is expressed as share of year spent with post-exposure, for following years it is equal to 1); $\boldsymbol{X_i}$ is a set of covariates that includes a high school indicator and a gender dummy; ϵ_{git} is the error-term; and the two last sets of indicator variables $I(Age)$ and $I(Year)$ capture age and incident year (for control group, the year indexed against). Under an assumption of parallel trends in salaried earnings (i.e., assuming that control and exposure group(s) would have continued to show similar trends in salaried earnings had the exposure group not experienced concussions), δ then captures the annual causal effect of concussion on salary for people exposed to concussions (see Supplemental Methods for further details). For additional exploratory analyses, separate models across gender, educational level, and age, as well as across the salary distribution are also estimated (see Supplemental Methods, Supplemental Digital Content 1, for further details). The authors document and make available all code needed to reproduce the findings in the study (Supplemental Digital Content 2).

Standard Protocol Approvals, Registrations, and Patient Consents

Since the data used in the study come from de-identified administrative registers that Statistics Denmark makes available for research purposes for approved institutions, no approval from an ethics committee was needed to carry out the study. The research was carried out as part of project no. 706630 approved by Statistics Denmark.

For peer review only

Patient and Public Involvement

There was no involvement from patients or members of the public in the design, or conduct, or reporting, or dissemination plans of the research.

For peer review only

For peer review only - http://bmjopen.bmj.com/site/about/guidelines.xhtml

Results

Concussion leads to long-term loss in salaried income

Individuals who suffered a concussion (exposure group) had a lower salaried income compared to individuals who experienced their concussion 1, 2, 3, 4 and 5 years after the exposure group (control groups). Compared to patients who experienced a concussion one year after the exposure group, salaried income was €423/£380 (95% CI: -€9129/-£8208; €73/£66, p=.095; Table 2) lower, corresponding to a salary decrease of 1.5 % (95% CI: -0.3 %; 3.2 %; Figure 1). Compared to patients who experienced a concussion 5 years after the exposure group, however, salaried income in the exposure group was €1,243 (95% CI: -€1,564/-£1,406; -€922/-£829, p<.001) lower, corresponding to a salary decrease of 4.2 % (95% CI: 3.1 %; 5.3 %; Figure 1). Normalized wage progression for the control groups, who suffered a concussion 1, 2, 3, 4, and 5 years after the exposure group, showed similar trends and similar levels pre-exposure, indicating that the parallel wage trends assumption was met (Figure 2 and table S1, Figures S1 in Supplemental Digital Content 3).

We hypothesized that the salary decreases resulted from a combination of lower salary and exit from the labour market, either through short- or long-term absence/unemployment. In an exploratory analysis, we tested whether labour force exit drove the full effect of concussion on salary (Figure 3). By comparing the cumulative distribution of salary density for the exposure group with the cumulative distribution of salary density for the Δ=5 control group (Figure 3, left panel), we found that the impact of concussion on salary was significant for individuals in the lower quartile of the salary distribution (at a 95 % significance level). Specifically, below a threshold salaried income of 40,000€ (£36,000) the presumed impact of concussion on salary

For peer review only - http://bmjopen.bmj.com/site/about/guidelines.xhtml

increased towards the bottom of the earnings pyramid; Figure 3, right panel).

Comparing the exposure group to the control group Δ=5, which suffers a concussion five years after the exposure group, concussion was associated with a 2.6% (95% CI: 3.0 %; 2.2 %, p <.001) increase in the risk of receiving € 0 in annual salary.

Long-term loss in salaried income stems from exit from the labour market

To further examine whether exit from the labour market was caused either through short- or long-term absence/unemployment, we estimated a dynamic model using the control group Δ=5, which suffers a concussion five years after the exposure group. Sick leave benefits payments were higher in the exposure group compared to the control groups for the first two years following concussion. Sick leave benefits were no longer different from year 3 while the difference in annual salary between exposure and control groups persisted. Further, employment in the exposure group remained lower than in the control group Δ=5 and remained so for the entire post-exposure period (see table S2, Supplemental Digital Content 3 for further details). To assess whether some form of public benefits covered part of the salary loss, total income decline was compared to salary decline following concussion. Indeed, total income decline was lower than the salary decline through a five year period (see Figure S2, Supplemental Digital Content 3 for further details).

Younger patients without high school degree drove the effect of concussion on income loss

The exposure group and all control groups differed slightly in terms of average patient age, male to female ratio, and for control group Δ=5, in the frequency of individuals with at least a

For peer review only - http://bmjopen.bmj.com/site/about/guidelines.xhtml

high school degree (see Table S3, Supplemental Digital Content 3 for further details). To ensure that differences in gender, education, or age did not influence our results, we subdivided our exposure group into subgroups based on gender, education status, and age at time of concussion. We then estimated the impact of concussion on salary and employment across all values of Δ and for all subgroups (see, Figures S3-S8, Supplemental Digital Content 3 for further details). Patients between age 30-39 and those without a high school degree experienced the largest absolute and relative declines in salary.

Finally, we addressed the role of timing of concussion across different years. Given that per design our exposure group always suffered their concussion earlier than the control groups do, changing labour market conditions could moderate effects. Part of our sample suffered their concussion during or just prior to the Great Recession in 2009-2010, which arguably presented the largest shock to both the global and local economy since the Great Depression in the 1930s. In Denmark, the great recession was preceded by a series of years of economic growth, low unemployment, and increasing salaries (see Figure S8, Supplemental Digital Content 3 for salary development from 1994 to 2017). We estimated the impact of concussion on salary separately for each year from 2003-2012 and plotted the estimate against the percent of full-time unemployment in the Danish labour force (Figure 4). Suffering a concussion during an economic boom had a substantially higher impact on salary than doing so during a recession when comparing to control groups who suffered concussions two to five years later than exposure group.

For peer review only

For peer review only - http://bmjopen.bmj.com/site/about/guidelines.xhtml

Discussion

The impact of concussion on employment and salary remains understudied. In a systematic review of four studies on the association between mild TBI and return to work, Cancelliere and colleagues[9] found that most workers return to work within 3-6 months of suffering a mild TBI, but that the long-term impact (more than one year after concussion) was not studied. In addition, studies included small to medium sample sizes, varied measures of return to work, and employed both case-control and cohort designs. Using an inception cohort study design, Theadom and colleagues[7] collected follow up data four years after mild TBI incidents on 245 New Zealanders who were employed prior to incident. They found a 3.6 percent productivity decline among those who suffered a concussion, compared to a 2.3 population average decline. The group suffering mild TBI also reported more difficulties carrying out work-related tasks. In a related study,[7] Theadom and colleagues further found that the mild TBI group had persisting cognitive symptoms four years after suffering their concussion compared to an age-sex matched control group. Also using a case-control design and data from Taiwan, Chu and colleagues[6] found that one month after incident, 26 percent of patients had still not managed to return to work, and a large share of those who did return scored below full-time employment on a work quality index. Only one other study by Graff and colleagues[10] include a large patient cohort (n=19,732). Using case-control they found an association between concussion and failing to return to work of 1.54 odds ratio, but also found that exposed individuals had lower labour market attachment and was more likely to receive health related benefits pre-incident compared to the control group.

In the present study, we overcame some of the obstacles faced by previous work on the impact

For peer review only

271 of concussions on labour market outcomes by including a vast cohort of patients and exploiting a quasi-experimental design that allow us to plausibly account for unobserved difference between exposure and control group. In such a quasi-experimental setup exposure and control groups only differ in the timing of concussion. Since everyone in the control group experiences a concussion within five years after individuals in the exposure group, the groups are likely to be balanced on unobservable characteristics. This is particularly important given the number of potential factors that can influence employment after concussion[16,17]. Data from Donker-Cools et al.[17], for instance, suggests larger employers are more able to keep those who have sustained brain injuries in work compared to smaller employers. Furthermore, since our data did not include health-related data such as past psychiatric history, we cannot exclude that exposure and control groups differed in health-related aspects and that these differences biased our results, i.e. that an individual left the workforce for a concussion-unrelated cause like a psychiatric disease triggered by the stress of a concussion event. Thus, even if we believe that our quasi-experimental setup leaves us with exposure and control groups that only differ in the timing of concussion, especially given the reported sample sizes and the finding that exposure and control groups show similar pre-exposure trends on both primary and secondary outcomes (Figure 1 and Tables S1 and S2),, this aspect needs to be discussed as a potential limitation of our study.

In addition, salary and employment data reported here were compiled routinely through third-party reporting and were mandatory for all subjects, thus giving a complete and comprehensive picture of the economic impact of concussion on a nationwide scale. It should be mentioned that our study also included data from individuals diagnosed in private hospitals. However,

For peer review only - http://bmjopen.bmj.com/site/about/guidelines.xhtml

given the setup of the Danish health care system, i.e. private hospitals predominantly do selective and overflow surgery and have no ERs[18], only 13 patients were diagnosed at a private hospital from 2003 onwards.

Altogether, we showed that Danes between 20-59 year of age, who suffered a concussion during the period 2003-2012 experienced average salary losses of 4.2%. The impact of concussions on salary already materialized one year after the incident and remained sizeable for at least five years. This result is in line with a "burn-in" period in which the impact of concussion on wages fully develops. First, concussions occur at some point during the year, thereby not affecting already earned salary that year. Second, in Denmark, most employees are entitled to receive their salary during sickness absence for an open ended, but not indefinite period. The results further showed that both in absolute and relative terms, people with an educational level at less than a high school degree saw substantially larger negative impact to salaried earnings than did those with at least a high school degree. Also, the group with less than a high school degree also saw an immediate impact on salary from their concussion (cf. Figure S4), indicating that the burn-in period present for workers with at least high school education likely expressed differences in types of employment and job protection.

In addition, total income decline was lower than the salary decline through a five year period (see Figure S2, Supplemental Digital Content 3 for further details), suggesting that the impact of concussions on salary largely stems from affected individuals leaving the labour force completely, likely sustaining themselves through early retirement, disability pensions, self-sufficiency, or other income sources instead.

It is important to mention that our study was restricted to individuals diagnosed in ER and

For peer review only - http://bmjopen.bmj.com/site/about/guidelines.xhtml

hospital settings and individuals diagnosed by a GP might differ from the population studied here. Rowson et al., however, show that in concussed individuals, severity of the cranial injury is not strongly correlated with strength or length of subsequent symptoms[19]. Thus, individuals diagnosed by a GP might suffer concussion effects as much as individuals who initially sustained a more severe cranial injury and sought medical attention in an ER or hospital setting. If this holds true, our results may have validity beyond individuals diagnosed in an ER or hospital setting.

If we assume that people return to their expected salary levels after a five-year recovery period (a very conservative assumption that is not supported by our data), the mere net annual salary loss in our sample would be approximately €23,000,000 (£21,000,000) measured in 2015-value. That would neither include hospital charges, medical costs for the treatment of concussion, the foregone tax from income, and the increased need for welfare spending, nor would it account for the large group of individuals who never seeks treatment[20] or receive their diagnosis from their general practitioner rather than in a hospital or emergency room, and thus escape our study. Thus, total public costs are likely substantially higher.

In addition, both in absolute and relative terms, the early peak-working aged individuals (30-39 y) and the less-educated individuals in our cohort seemed to be most affected after suffering a concussion. These findings might have and additional and yet unmeasured social impact, especially if our results are transferrable to other nations with a less established welfare state and a less flexible labour market. In such countries, the impact on the young and less-educated individuals suffering a concussion and thus on society might be accentuated.

For peer review only

Comparing our hospital incidence rates to more complete canvases of incidences carried out by Cassidy et al.[21], it seems likely that the actual cost in the population is more than twice as large as what we estimate, assuming that individuals not diagnosed in a hospital setting on average suffer the same extent of concussion symptoms. If we were to consider the average concussion incidence rates for six other advanced European countries that are somewhat comparable to Denmark (Norway, Finland, Germany, Netherlands, England and France) and under the assumption that concussion have a similar impact on earnings in these countries, the net annual salary loss would be approximately €1,099,400,000 (£988,4780,000) measured in 2015-value. While our study likely underestimates the total socioeconomic impact of concussion, it suggests that concussions has a large economic impact on a nationwide scale and on productivity and income at the patient level.

Conclusion

Using timing of concussion as a natural experiment, we provide first plausible causal estimates of the effect of concussion on salary and employment among patients treated for concussion in an emergency room or hospital setting in Denmark, 2003-2017. Our results show that among this patient group concussion has a large and long-term negative causal impact on salary and employment. People between 30-39 y and those without high school degrees suffered the largest salary decreases.

For peer review only - http://bmjopen.bmj.com/site/about/guidelines.xhtml

Disclosures:

The authors report no conflict of interest.

Acknowledgements:

The authors thank Torben Heien Nielsen for helpful comments on an earlier draft of the manuscript.

Funding:

This work was supported by the Swedish Research Council for Health (PF), Working Life and Welfare (Grant no. 2016-07099) with additional funding from the ROCKWOOL Foundation (PF). The research was carried out independently of the funders.

Authors contributions: P.F. and B.C. conceived of the presented idea, P.F. performed the computations. P.F. and B.C. verified the statistical methods. P.F. and B.C. discussed the results and wrote the manuscript. The corresponding author confirms that he had full access to all the data in the study and had final responsibility for the decision to submit for publication.

Data Availability Statement

The data used in this study has been made available through a trusted third party, Statistics Denmark. Due to privacy concerns the data cannot be made available outside the hosted research servers at Statistics Denmark. University-based and private Danish scientific organizations can be authorized to work with data within Statistics Denmark. Such organization

For peer review only - http://bmjopen.bmj.com/site/about/guidelines.xhtml

can provide access to individual scientists inside and outside of Denmark. Requests for data may be sent to Statistics Denmark: http://www.dst.dk/en/OmDS/organisation/TelefonbogOrg.aspx?kontor=13&tlfbogsort=sektion or the Danish Data Protection Agency: https://www.datatilsynet.dk/english/the-danish-data-protection-agency/contact/. The authors document and make available all code needed to reproduce the findings in the study.

For peer review only

For peer review only - http://bmjopen.bmj.com/site/about/guidelines.xhtml

References

1 Cassidy JD, Carroll LJ, Peloso PM, *et al.* Incidence, risk factors and prevention of mild traumatic brain injury: results of the WHO Collaborating Centre Task Force on Mild Traumatic Brain Injury. *J Rehabil Med* 2004;:28–60. doi:10.1080/16501960410023732

2 Pinner M, Jensen R, Birket-Smith M, *et al.* Konsensusrapport om commotio cerebri (hjernerystelse). Videnscenter for Hjerneskade 2003.

3 Hou R, Moss-Morris R, Peveler R, *et al.* When a minor head injury results in enduring symptoms: a prospective investigation of risk factors for postconcussional syndrome after mild traumatic brain injury. *J Neurol Neurosurg Psychiatry* 2012;**83**:217–23. doi:10.1136/jnnp-2011-300767

4 Ruff R. Two decades of advances in understanding of mild traumatic brain injury. *J Head Trauma Rehabil* 2005;**20**:5–18. doi:10.1097/00001199-200501000-00003

5 Silverberg ND, Iverson GL. Is rest after concussion "the best medicine?": recommendations for activity resumption following concussion in athletes, civilians, and military service members. *The Journal of head trauma rehabilitation* 2013;**28**:250–9. doi:10.1097/HTR.0b013e31825ad658

6 Chu SY, Tsai YH, Xiao SH, *et al.* Quality of return to work in patients with mild traumatic brain injury: a prospective investigation of associations among post-concussion symptoms, neuropsychological functions, working status and stability. *Brain Injury* 2017;**31**:1674–82. doi:10.1080/02699052.2017.1332783

7 Theadom A, Barker-Collo S, Jones K, *et al.* Work Limitations 4 Years After Mild Traumatic Brain Injury: A Cohort Study. *Archives of Physical Medicine and Rehabilitation*

For peer review only

For peer review only - http://bmjopen.bmj.com/site/about/guidelines.xhtml

2017;**98**:1560–6. doi:10.1016/j.apmr.2017.01.010

8 Theadom A, Starkey N, Barker-Collo S, *et al.* Population-based cohort study of the impacts of mild traumatic brain injury in adults four years post-injury. *PLOS ONE* 2018;**13**:e0191655. doi:10.1371/journal.pone.0191655

9 Cancelliere C, Kristman VL, Cassidy JD, *et al.* Systematic Review of Return to Work After Mild Traumatic Brain Injury: Results of the International Collaboration on Mild Traumatic Brain Injury Prognosis. *Archives of Physical Medicine and Rehabilitation* 2014;**95**:S201–9. doi:10.1016/j.apmr.2013.10.010

10 Graff HJ, Siersma V, Møller A, *et al.* Labour market attachment after mild traumatic brain injury: nationwide cohort study with 5-year register follow-up in Denmark. *BMJ open* 2019;**9**:e026104. doi:10.1136/bmjopen-2018-026104

11 Ogilvie D, Adams J, Bauman A, *et al.* Using natural experimental studies to guide public health action: turning the evidence-based medicine paradigm on its head. *J Epidemiol Community Health* Published Online First: 19 November 2019. doi:10.1136/jech-2019-213085

12 Dobkin C, Finkelstein A, Kluender R, *et al.* The Economic Consequences of Hospital Admissions. *American Economic Review* 2018;**108**:308–52. doi:10.1257/aer.20161038

13 Fadlon I, Nielsen TH. Household Responses to Severe Health Shocks and the Design of Social Insurance. Cambridge, MA: 2015. doi:10.3386/w21352

14 Schmidt M, Schmidt SAJ, Sandegaard JL, *et al.* The Danish National Patient Registry: a review of content, data quality, and research potential. *Clinical epidemiology* 2015;**7**:449–90. doi:10.2147/CLEP.S91125

For peer review only

15 Statistics Denmark. Documentation of statistics: Income Statistics - Statistics Denmark. 2015.https://www.dst.dk/en/Statistik/dokumentation/documentationofstatistics/income-statistics (accessed 20 Aug 2018).

16 Buck PW, Laster RG, Sagrati JS, *et al.* Working with mild traumatic brain injury: voices from the field. *Rehabil Res Pract* 2012;**2012**:625621. doi:10.1155/2012/625621

17 Donker-Cools BHPM, Wind H, Frings-Dresen MHW. Prognostic factors of return to work after traumatic or non-traumatic acquired brain injury. *Disabil Rehabil* 2016;**38**:733–41. doi:10.3109/09638288.2015.1061608

18 Vrangbaek K, Østergren K, Birk HO, *et al.* Patient reactions to hospital choice in Norway, Denmark, and Sweden. *Health Econ Policy Law* 2007;**2**:125–52. doi:10.1017/S174413310700401X

19 Rowson S, Duma SM, Stemper BD, *et al.* Correlation of Concussion Symptom Profile with Head Impact Biomechanics: A Case for Individual-Specific Injury Tolerance. *J Neurotrauma* 2018;**35**:681–90. doi:10.1089/neu.2017.5169

20 Setnik L, Bazarian JJ. The characteristics of patients who do not seek medical treatment for traumatic brain injury. *Brain Injury* 2007;**21**:1–9. doi:10.1080/02699050601111419

21 Cassidy JD, Carroll LJ, Peloso PM, *et al.* Incidence, risk factors and prevention of mild traumatic brain injury: Results of the WHO Collaborating Centre Task Force on mild traumatic brain injury. *Journal of Rehabilitation Medicine* 2004;**36**:28–60. doi:10.1080/16501960410023732

For peer review only - http://bmjopen.bmj.com/site/about/guidelines.xhtml

For peer review only

Figure Legends

Figure 1. Estimated effect of concussions in percentage on salary for the exposure group measured against each control group

Note: Figure shows the percentage change in salary experienced by the exposure group following their concussions compared to the expected trajectory absent the concussion (calculated from the control groups) with 95 % confidence intervals. See table 1 for separate p-values for each estimate.

Figure 2. Salary development for exposure and control groups across time of exposure

Note: Figure shows the salary trajectories for the exposure group (black) who suffers concussion at year zero against normalized wage trajectories for the control groups who suffer their concussions one to five years later. Δ indicates the number of years between exposure and control incident. Table shows that there are no significant differences in the normalized salary levels for exposure and control group prior to exposure incident (see Figure S1, Supplemental Digital Content 3 for unnormalized salary trajectories).

Figure 3. (Left panel) The cumulative density function (cdf) for salary post-treatment among the treatment group and their counterfactual outcome had they not experienced their concussions, and (Right panel) the change in salary density for the exposure group compared to their counterfactual baseline expressed as the effect of concussion on the probability of earning below the salary-level expressed on the x-axis following exposure

For peer review only

For peer review only - http://bmjopen.bmj.com/site/about/guidelines.xhtml

event.

Note: The figure shows the observed cumulative salary distribution following concussion for the exposure group (red) and the expected counterfactual salary distribution absent suffering concussion in the exposure group (blue), when using the Δ=5 control group. The black line shows the difference between the observed and the counterfactual distribution, and the grey dash lines show the 95 % confidence interval. The close to constant decline of the difference between the two distributions as the salary increase indicates that the main part of the effect of concussions on salary are driven by people having a salary equal to zero.

Figure 4. Effect of concussion on salary across incident years and control groups together with the percentage fulltime unemployed of the labor force.

Note: Figure shows annual estimates of concussion against each control group separately mapped against the share of the labor force that is full time unemployed. 95 % confidence intervals. The estimates for the effect of concussion on salary almost uniformly increase in absolute magnitude when unemployment decreases, and decrease when unemployment increase, indicating that the effect of concussion on salary is countercyclical to the economic cycle.

Supplemental Digital Content titles & legends

Supplemental Digital Content [#1]. Text file. Supplemental materials and methods. This file contains further details on our quasi-experimental, difference-in-differences event time approach.

For peer review only

Supplemental Digital Content [#2]. File. Code used for the analyses.

Supplemental Digital Content [#3]. Table. Supplemental results Table S1: Test of parallel trends assumption pre-exposure incident against each control group separately using eq. S3 in supplementary methods. Separate exposure dummies for all time periods (except the year prior to exposure, which serves as reference period).

Supplemental Digital Content [#3]. Table. Supplemental results Table S2: Effect of concussion on different labor market outcome parameters using separate exposure dummies for all time periods (except the year prior to exposure, which serves as reference period): In this exploratory analysis, the exposure group is compared to the control group Δ=5, which suffers a concussion five years after the exposure group. Outcomes include annual salaried income (annual salary), total annual income (total income), annual sick leave benefits received (sick leave benefits) as well as a binary indicator of employment (probability of employment). Monetary outcomes were measured at 2015-level in € 1,000.

Supplemental Digital Content [#3]. Table. Supplemental results Table S3: Demographic factors for exposure group and control groups (Δ=1, 2, 3, 4, 5) averaged over the 5 years leading up to the concussion event in each of the groups. Factors include patient age (in years), share of sample female (1=100% female), and share of individuals with at least a high school degree (1=100%).

For peer review only - http://bmjopen.bmj.com/site/about/guidelines.xhtml

Supplemental Digital Content [#3]. Figure. Supplemental results Figure S1: Unnormalized Average Salary for Treatment and Control Groups Measured in 1K € at 2015-levels

Supplemental Digital Content [#3]. Figure. Supplemental results Figure S2: The Cumulative Density Function (CDF) for Total Income Post-Treatment among the Treatment Group and Their Counterfactual, and the Difference between the Two CDFs Expressed as the Effect of Concussion on the Probability of Total Income Below that Income-Level following Exposure Event.

Supplemental Digital Content [#3]. Figure. Supplemental results Figure S3: Percentage Effect of Concussion on Relative Salary Across Age Groups.

Supplemental Digital Content [#3]. Figure. Supplemental results Figure S4: Percentage Effect of Concussion on Relative Salary Across High School Completion.

Supplemental Digital Content [#3]. Figure. Supplemental results Figure S5: Percentage Effect of Concussion on Relative Salary Across Gender.

Supplemental Digital Content [#3]. Figure. Supplemental results Figure S6: Effect of Concussion on Absolute Salary in 1K Euro Across Age groups.

For peer review only

For peer review only - http://bmjopen.bmj.com/site/about/guidelines.xhtml

Supplemental Digital Content [#3]. Figure. Supplemental results Figure S7: Effect of Concussion on Absolute Salary in 1K Euro Across Education.

Supplemental Digital Content [#3]. Figure. Supplemental results Figure S8: Effect of Concussion on Absolute Salary in 1K Euro Across Gender.

Table 1. Number of observations for exposure and control groups across time since exposure and number of observations with missing salary information

Years until exposure	Exposure group	Control Δ=1	Control Δ=2	Control Δ=3	Control Δ=4	Control Δ=5
-4	36,804	33,681	31,112	29,190	27,859	26,794
-3	36,978	33,834	31,245	29,366	27,973	26,907
-2	37,195	34,003	31,407	29,501	28,146	27,031
-1	37,449	34,224	31,582	29,687	28,288	27,220
0	37,848	34,551	31,851	29,922	28,530	27,421
1	37,467		31,755	29,832	28,433	27,337
2	36,940			29,807	28,421	27,295
3	36,484				28,421	27,304
4	36,084					27,314
Total observations	333,249	170,293	188,952	207,305	226,071	244,623
Observations with missing salary	81	32	31	44	35	29

Control groups have not suffered a concussion in 10+Δ years before incident, exposure group has not suffered concussion the 10 years before exposure incident.

For peer review only

For peer review only - http://bmjopen.bmj.com/site/about/guidelines.xhtml

552 **Table 2. Estimated effect of concussion on salary of exposure group compared to**
553 **control groups that suffered their concussion Δ = 1, 2, 3, 4, 5 y after the exposure**
554 **group's concussion event, measured at 2015-level. $N_{Exposure}$: 37,848**

555

	Estimated salary effect (δ)	95 % CI	*p*	$N_{Control}$
Δ = 1 y	-423€	(-919€;73€)	.095	34,551
Δ = 2 y	-825€	(-1,108€; -543€)	<.001	31,851
Δ = 3 y	-1,019€	(-1,331€; -707€)	<.001	29,922
Δ = 4 y	-1,126€	(-1,446€; -805€)	<.001	28,530
Δ = 5 y	-1,243€	(-1,564€; -922€)	<.001	27,421

556

557 Results obtained from estimations following Eq. (1). Models include controls for high school
558 diploma, gender, age, and observation year. Results obtained using reghdfe in Stata.

559

For peer review only

For peer review only - http://bmjopen.bmj.com/site/about/guidelines.xhtml

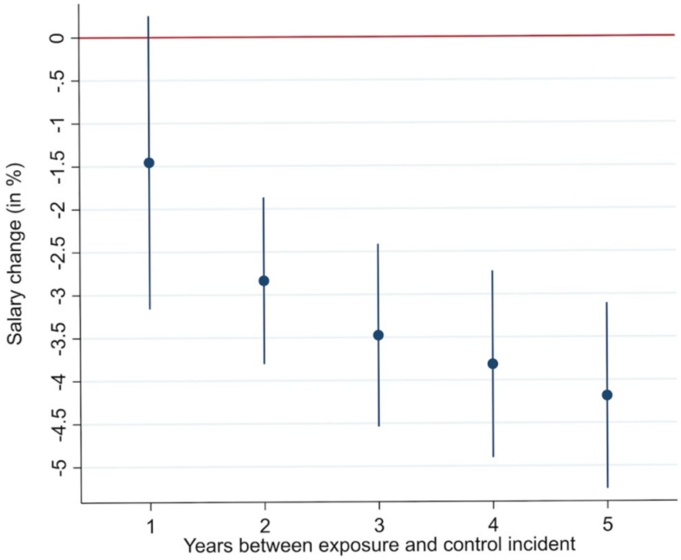

Figure 1

338x190mm (108 x 108 DPI)

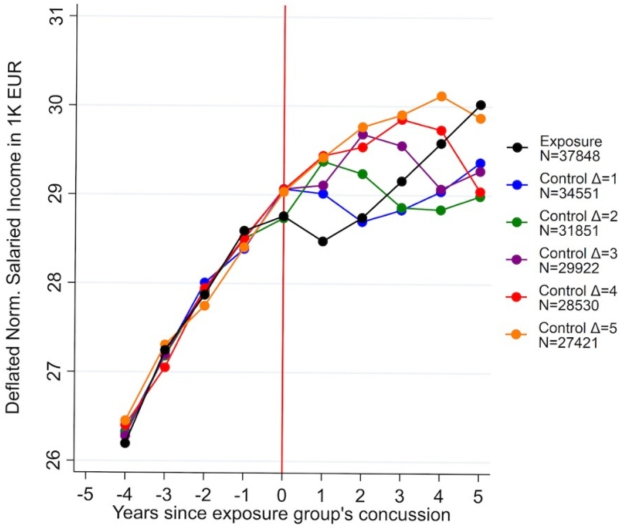

Figure 2

338x190mm (108 x 108 DPI)

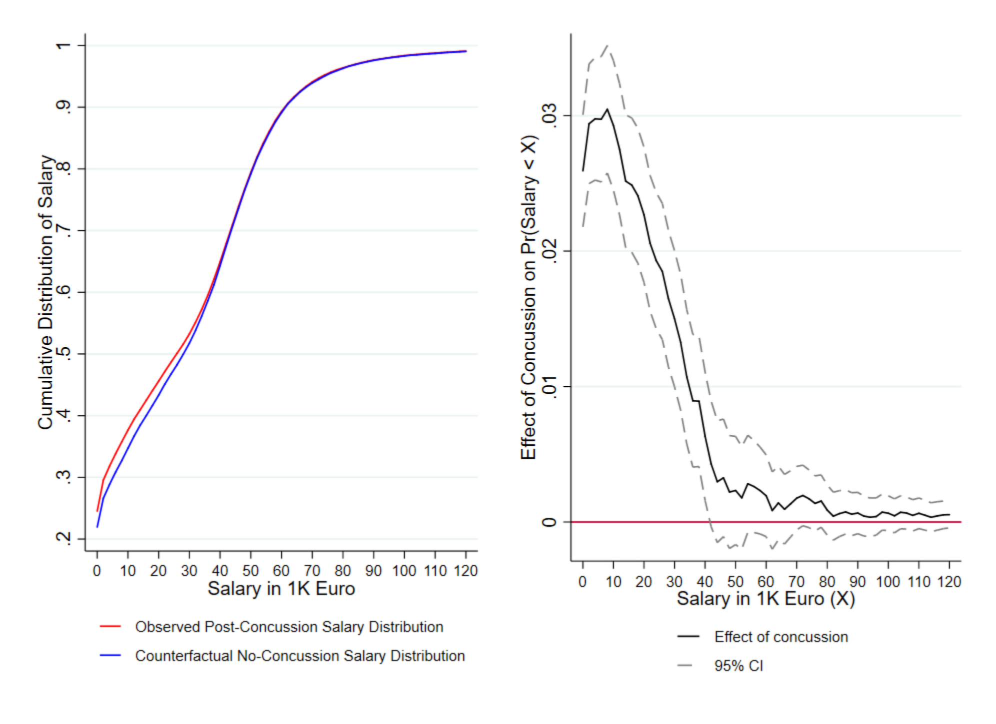

Figure 3

352x256mm (72 x 72 DPI)

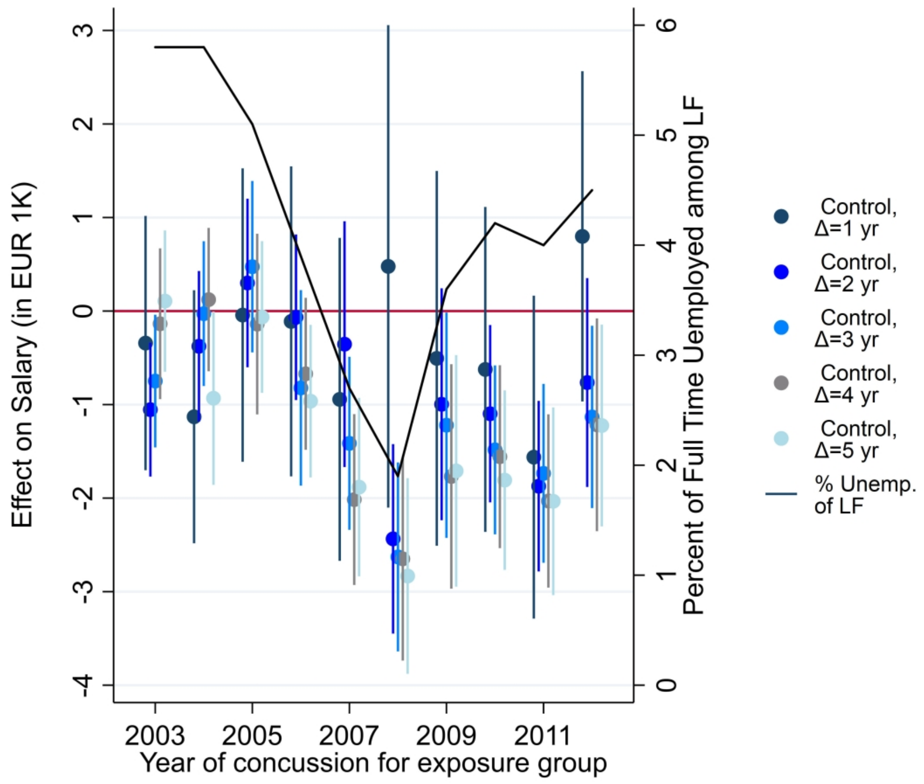

Effect on Salary (in EUR 1K)
3
2
1
0
-1
-2
-3
-4
Percent of Full Time Uemployed among LF
6
5
4
3
2
1
0
2003
2005
2007
2009
2011
Year of concussion for exposure group
Control, Δ=1 yr
Control, Δ=2 yr
Control, Δ=3 yr
Control, Δ=4 yr
Control, Δ=5 yr
% Unemp. of LF

Figure 4

Supplemental materials and methods

Our quasi-experimental, difference-in-differences event time approach compares two groups of individuals from the same cohort, where both groups experience concussions, but at two different time points (t_c,$t_c+\Delta$). For the simple situation where we have three periods (t=0,1,2) and the exposure group (T) experiences their concussion at the start of period 1 (t_c=1), and the control group (C) at the start of period 2 ($t_c+\Delta=2$), the effect of concussion on salary (Y) is:

$$\Delta = \left(Y_1^T - Y_1^C\right) - \left(Y_0^T - Y_0^C\right)$$

The effect of concussion on salary in t=1 is estimated by comparing the average difference in salary between exposure and control groups for the post-concussion period t=1 $\left(Y_1^T - Y_1^C\right)$ to the average difference in salary for the pre-concussion, or baseline, interval t=0 $(Y_0^T - Y_0^C)$. Assuming the exact timing of a concussion is random for small enough sizes of Δ, and under the additional assumption that the exposure group would have had parallel trends in salary as the control group absent suffering concussion at t_c, δ captures the causal effect of concussion among those who suffer concussions – also known as the average effect on the treated (AT). The AT does not capture how concussions would affect a random person. The AT captures how concussions causally affect those who suffer concussions.

For our study, the parallel trends assumption states that exposure and control groups have parallel developments in salary leading up to the exposure group's concussion and the exposure and control groups would have further exhibited parallel salary trajectories if the concussion had not occurred. To test the parallel trends assumption, we estimate a dynamic version of the model specification (shown in supplementary table S1), which explicitly allows us to test whether the parallel trend assumption for our sample is probable.

To validate that the timing of concussion is random with our study period, we present estimates for effect of exposure across different periods between exposure and control incident (Δ). Most recorded concussions outside contact sports and military engagements stem from unforeseen events, such as falls or striking/being struck by an object[25,26], so assuming random timing is likely valid. People who regular engage in activities that result in high risk of multiple concussions may be different than the average concussion patient and would be more likely to end up in the exposure sample than in the control sample, which could induce bias. To avoid such potential bias, we restrict our sample to individuals without prior diagnoses for intracranial injuries ten years prior to exposure.

At t=-1, i.e. one year before the exposure group suffered a concussion, the control groups were slightly smaller than the exposure group, and two control groups (Δ=4 and 5) differed slightly but significantly in terms of average patient age ($p <. 001$; supplementary table S2), male to female ratio ($p <. 001$), and for control group Δ=5, in the frequency of individuals with at least a high school degree ($p < .001$). However, the differences are numerically small. To test that composition differences between exposure and control do not drive our results, we provide separate results for individuals with and without high school degree, for males and females, and for different age groups across all different values of Δ.

Further, our design inherently leads to the possibility of timing issues—our exposure group always suffers their concussion earlier (in terms of calendar time and age) than the control groups do. If the labor market is constantly improving or worsening during the period we consider, this could substantially influence our results. Therefore, we also estimate separate models across exposure incident year and control group. Estimating separate models allow us the added benefit of being able to examine whether the business cycle influences the effect of concussions on salary.

Statistical model

To estimate the impact of concussion on salary, we define the following variables: Exposure or control group g, which includes individuals i, at times to exposure-groups concussion incident t. First, we estimate a standard difference in differences model for each separate control group Δ={1, 2, 3, 4, and 5} using ordinary least squares:

$$Salary_{git} = \beta_0 + \gamma exposure_g + \theta post_t + \delta post \times exposure_{git} + \boldsymbol{X_i \beta} + \sum_{Age=26}^{48+\Delta} I(Age)\eta_{age} + \sum_{year=1999}^{2012} I(year)\,\eta_{year} + \epsilon_{git} \quad \text{(S1)}$$

where $Salary_{git}$ measures annual salaried income deflated to 2015-level, $exposure_g$ indicates whether the observation belongs to the exposure or control group, $post_t$ captures the period after the exposure group's concussion occurred, and $post_t \times exposure_{git}$ captures the effect concussion, measured as share of year $t \geq 0$ affected by concussion. In this way, someone who suffers a concussion July 1 has $post_t \times exposure_{git} = 0.5$ for $t = 0$ and $post_t \times exposure_{git} = 1$ for $t > 0$. $\boldsymbol{X_i}$ is a set of covariates that includes a high school indicator and a gender dummy, ϵ_{git} is the error-term, and the two last sets of indicator variables $I(Age)$ and $I(Year)$ capture age and incident year levels (control group indexed against incident year). Under the parallel trends assumption, δ then captures the annual effect of concussion on salary. In eq. 1, $exposure_g$ normalizes any pre-exposure differences between the exposure and control group, thereby creating a joint baseline pre-exposure.

We estimate robust individual-level clustered standard errors to account for the possibility that individuals enter the data twice both as control (0) and exposure (1) individuals (g={0,1}), and that they are observed for multiple periods (t={-4,…, Δ-1}). To calculate the relative salary decrease after concussion, we exploit the parallel trends assumption to generate the expected counterfactual salary level, i.e. had the concussion not occurred, and calculate the decline expressed in percentage as: $\%\,\text{change} = \delta \,/\, E(\widehat{Salary}_{git}|g = 1, post_t = 1, post_t \times exposure_{git} = 0)$. In this way, we provide both absolute estimates measured in 1K Euro, as well as percentage change.

We expect δ from eq. (1) to likely be negative. Yet, a decrease in annual salary can arrive through two different channels. Concussions may affect salary through either decreasing income among those employed or by reducing the number of individuals who are employed and earning any salary at. To parse out which of the two channels is driving the results, we examine how concussion affects the salary distribution among the exposure group following. Following Chernozhukov et al.[27] we estimate a series of regressions across the whole salary distribution, where, for a finite set of points, we predict how concussion affects the likelihood of having earnings on the left side of each finite point, as follows:

$$\sum_{j=0}^{\max(Salary)} p_j = \beta_{0j} + \delta_j post_t \times exposure_{git} + \theta post_t + \gamma_j exposure_g + \boldsymbol{X_i \beta} + \sum_{Age=26}^{48+\Delta} I(Age)\eta_{age,j} + \sum_{year=1999}^{2012} I(year)\,\eta_{year,j} + \epsilon_{git,j} \quad \text{(S2)}$$

where $p_j = \Pr\,(Salary_{git} \le j)$ and j is the interval from 0 to $max(Salary)$. Across the salary distribution, we can now predict the probability of earning less than j for those with and without concussions. From equation 2, we predict $p_j^1 = E(p_j|post_t \times exposure_{git} = 1, exposure_g = 1, t \ge 0)$ and the counterfactual $p_j^0 = E(p_j|post_t \times exposure_{git} = 0, exposure_g = 1, t \ge 0)$. Plotting p_j^1 and p_j^0 over each value of salary j, and assuming rank stability, gives the cumulative density function of salary for the treated (p_j^1) and the counterfactual observation of the treated had they not suffered concussions (p_j^0). The difference between p_j^1 and p_j^0 is simply δ_j. If the value of δ_j monotonically moves towards zero as j increases until $p_j^1 \approx p_j^0 \approx 1$ it indicates that exit from employment fully drives the effect of concussion on salary. If instead the value of δ_j is constant or increasing across parts of the distribution, it instead indicates that a decrease in salary among those still receiving salary drives at least part of the effect.

Eq. 1 and eq. 2 are based on the parallel trends assumption. The assumption states that exposure and control groups follow parallel salary trajectories until individuals in the exposure group experiences a concussion, and that the parallel trends would have continued had the concussion not occurred. Whereas we cannot verify the counterfactual situation of parallel trends after exposure, we can use a dynamic model to test for systematic differences in salary trends between exposure and control group in the years leading up to the exposure group's concussion event. To do so, we estimate the following dynamic model:

$$Salary_{git} = \beta_0 + \sum_{t\neq-1,t=-4}^{\Delta-1} \delta_t \times I(t_g) \times exposure_g + \sum_{t=-4}^{\Delta-1} I(t_g)\eta_t + \gamma exposure_g + \boldsymbol{X_i \beta} + \sum_{Age=26}^{48+\Delta} I(Age)\eta_{age} + \sum_{year=1999}^{2012} I(year)\,\eta_{year} + \epsilon_{git} \quad \text{(S3)}$$

Where we interact exposure group status (*exposure*$_g$) with indicators $I(t_g)$ capturing time from concussion. If the parallel trends assumption holds, then it must be the case {δ_{-4}, δ_{-3}, δ_{-2}}=0, whereas the size and sign of {$\delta_0 \ldots \delta_{\Delta-1}$} captures the dynamic effect of a concussion from the year of incidence and Δ-1 years onward. By estimating the effect of concussion on salary among different years of the study period, we are also able to capture how the impact of concussion on salary evolves year to year after the concussion has occurred. We further estimate eq. 3 for a series of related labor market outcomes (annual total income, annual amount of sickness benefits received, annual probability of being employed), to generate a more thorough understanding on how concussions affect labor market outcomes—i.e., if people experience a decrease in salary due to a concussion, are they then compensated through different types of welfare state services.

25 Faul M, Coronado V. Epidemiology of traumatic brain injury. *Handbook of clinical neurology* **127**:3-13, 2015

26 Taylor CA, Bell JM, Breiding MJ, Xu L. Traumatic Brain Injury–Related Emergency Department Visits, Hospitalizations, and Deaths — United States, 2007 and 2013. *MMWR Surveillance Summaries* **66**:1-16, 2017

27 Chernozhukov V, Fernández-Val I, Melly B. Inference on Counterfactual Distributions. *Econometrica* **81**:2205-2268, 2013

For peer review only

```
********************************************************************
*************************************************
********************************************************************
*************************************************
********************************************************************
*************************************************
*****
***** This study relies on restricted individual level
administrative data obtained
***** from Statistics Denmark.
*****
***** Only Danish research environments are granted authorization to
access data from Statistics Denmark. ***** Foreign
***** researchers can, however, get access to micro data through an
affiliation to a Danish authorized
***** environment.
***** Access is given to anonymized micro data, i.e. data at an
individual personal or corporate level. ***** Access takes
***** place through researcherís own pc over the Internet.
*****
***** See https://www.dst.dk/en/TilSalg/Forskningsservice for detail
*****
***** For the replication of present study, contact the ROCKWOOL
Foundation for access.
***** http://www.rockwoolfonden.dk/en/
*****
********************************************************************
*************************************************
********************************************************************
*************************************************
********************************************************************
*************************************************


********************************************************************
*************************************************
********************************************************************
*************************************************
********************************************************************
*************************************************
*****
***** This is the master data do-file for the full package of do-
files that generates the results for
***** Fallesen and Campos (2020). Execute files in the order they
are listed. For each file, specify a ***** home registry in place of
[home]. Further, generate subfolders [home]/data, [home]/tables, and
***** [home]/highdef to capture auxiliary data sets, tables, and
figures.
*****
********************************************************************
*************************************************
********************************************************************
*************************************************
```

```
***************************************************************************
*************************************************

***Install user-written reghdfe command for faster computation of
regressions
ssc install reghdfe

***Generate concussion samples and merges on covariates and outcome
do 01generate_sample.do

***Generates auxilary data sets the includes social benefit
recipiency indicator and levels of benefits received
do 02generate_benefits.do

***Generates auxilary data sets the includes social benefit
recipiency indicator and levels of benefits received
do 02generate_benefits.do

***Generate results
do 03generate_figures_and_results

exit, clear
```

```
clear all

*********************************************************************
***********
**
**              This program builds data for Fallesen & Campos
(2020)
**              study of concussion's impact on productivity
measured
**              through annual salary
**
*********************************************************************
***********


**Global for path to registry data
global dorg "E:/data/rawdata/706630"

*Global for processed data
global data "[home]/data"

/*globals for price index to calculate income at 2015-level across
years*/
/*Price index obtained from www.dst.dk/en/statistik/emner/priser-og-
forbrug/forbrugeriser/nettoprisindeks */

{
global price1980 = .358
global price1981 = .398
global price1982 = .439
global price1983 = .466
global price1984 = .494
global price1985 = .517
global price1986 = .521
global price1987 = .537
global price1988 = .564
global price1989 = .594
global price1990 = .612
global price1991 = .628
global price1992 = .642
global price1993 = .651
global price1994 = .662
global price1995 = .674
global price1996 = .688
global price1997 = .703
global price1998 = .713
global price1999 = .728
global price2000 = .751
global price2001 = .769
global price2002 = .788
global price2003 = .806
global price2004 = .817
global price2005 = .833
global price2006 = .850
```

```
global price2007 = .867
global price2008 = .899
global price2009 = .917
global price2010 = .936
global price2011 = .960
global price2012 = .978
global price2013 = .986
global price2014 = .994
global price2015 =1.00
global price2016 =1.005
global price2017 =1.017
}


/*Locate concussions and other TBIs from the Danish National Patient
Registry */
/**/
forvalue t = 1977/2017{
        if `t' < 1994 use $dorg/lpr_diag`t'.dta /// **uses ICD-8
codes until Dec. 31, 1993
                if substr(c_diag,1,2)=="85"
        if `t' > 1993 use $dorg/lpr_diag`t'.dta /// **uses ICD-10
codes from Jan. 1, 1994
                if substr(c_diag,1,4)=="DS06"
        **recovers encrypted social security number and admittance
date
        merge m:m recnum using $dorg/lpr_adm`t', keepus(pnr d_ind*)
keep(3)
        drop _merge recnum


        **Keeps diagnosis, diagnosis type, and individual id (pnr)
        keep pnr c_diag  c_diagtype pnr d_ind

        **generate year variable
        gen year = year(d_ind)

        **geenrate share of year with concussion
         gen time_from_incident = 1-((d_ind-mdy(1,1,year(d_ind)))/
365)
        drop d_ind

        *save as one dataset
        if `t' >  1977 append using $data/concussion.dta
        if `t' == 2017 sort pnr year
        save $data/concussion.dta, replace


}
*/
/*******************************************
Sets up datasets for treatment group (x = 0)
and the control groups who suffer concussion
1, 2, 3, 4, 5 years later (x = 1 2 3 4 5).
```

```
Treatment group are not allowed to have suffered
any type of TBI the last 10 years before concussion
, control group are not allowed to have suffered any TBI
concussion the last 10 + x years.

******************************************/
/**/
forvalue x = 0/5{
        use $data/concussion.dta, clear
        sort pnr year

        **generate measure of length between registered TBIs
        by pnr: gen help = year-year[_n-1]

        drop if help < 10+`x'

        **Keep only concussion, and only when it was primary
diagnosis
        keep if c_diagtype== "A" & ///    Primary daignosis
                (c_diag == "85099" | /// ICD-8 code for concussion
                substr(c_diag,1,5) == "DS060") // ICD-10 code for
concussion

        *generate treatment and control datasets
        save $data/concussion_`x'.dta, replace

}



*/
/***************************************************************
Generate datasets for analysis. First incident year is allowed
to be 1992, because it is the first year where we have full record
for the five year plus control group (1977+10+5 = 1992).

We generate seperate datasets for each incident year for treatment
group and different control groups.

***************************************************************/

forvalue time = 1/5{             //for different control groups

        local post_period = 5    // local for number of years
observed post
                                                          //
concussion for treatment group

        local endtime = 2017-`post_period'        /*last year where
we allow for treatment event
                                                          to
occur, in order to have long enough control
                                                          period.
```

```
Defined by latest year available data*/

        forvalue count = 1992(1)`endtime'{
                local t = `count'                         // for
ease of coding
                local n = `t'-4                           // first
pre-treatment event period
                local c = `t'+`post_period'       //last post-event
period
                local w = `t'+`time'              //time of
concussion for control

                use pnr alder using $dorg/bef`t'          ///
Bring in all 30-49 yr olds
                        if inrange(alder`t',20,59), clear // from
the population register

                **year variable
                gen year = `t'

                **limit sample to those who suffer a concussion in
`t'
                merge 1:1 pnr year using $data/
concussion_0.dta, ///
                        keep(3) nogen

                forvalue x=`n'/`c'{       //add longitudinal data
                        merge 1:1 pnr using $dorg/bef`x', ///
                                keep(1 3) keepus(efalle alder
koen)   //add information on spouse,

                                        //age, and gender
                        rename _merge merge`x' //indicator for
whether in DK that year

                        **Add salary information and ses
information
                        if `x' < 2017{
                                merge 1:m pnr using $dorg/
ind`x', ///      m:1 to account for duplicates
                                        nogen keep(1 3)
keepus(erhvervsindk_13 pre_socio personindk dispon_13
aekvivadisp_13)  // in data on non-important variables

                                bysort _all: keep if _n ==1
                                        //drop perfect duplicates
                                **Align variable names and account
for inflation

                                rename erhvervsindk_13 loenmv
                                rename pre_socio pre_socio`x'

                                foreach kk in personindk dispon_13
aekvivadisp_13 loenmv{
```

```
                                                            rename `kk'
        `kk'`x'
                                        }

                                        foreach kk in personindk dispon_13
        aekvivadisp_13 loenmv{
                                                            replace `kk'`x' =
                `kk'`x'/${price`x'}
                                        }

                                        **Bring in educational information
                                        merge 1:1 pnr using $dorg/
        uddany`x', ///
                                        nogen keep(1 3) keepus(hffsp)
                                }
                                if `x' == 2017{
                                        merge 1:m pnr using $dorg/
        ind`x', ///      m:1 to account for duplicates
                                                nogen keep(1 3)
        keepus(erhvervsindk_13 pre_socio personindk)       // in data on
        non-important variables

                                        bysort _all: keep if _n ==1
                                                //drop perfect duplicates
                                        **Align variable names and account
        for inflation

                                        rename erhvervsindk_13 loenmv
                                        rename pre_socio pre_socio`x'

                                        foreach kk in personindk loenmv{
                                                            rename `kk'
        `kk'`x'
                                        }

                                        foreach kk in personindk loenmv{

                                                            replace `kk'`x' =
                `kk'`x'/${price`x'}
                                        }

                                        **Bring in educational information
                                        merge 1:1 pnr using $dorg/
        udda`x', ///
                                                nogen keep(1 3)
        keepus(hfaudd)
                                        rename hfaudd hfaudd`x'
                                }
                        }

                        **Reshape data to panel structure
                        if `count'>= 2012 reshape long efalle alder koen
        loenmv pre_socio personindk dispon_13 aekvivadisp_13 hffsp merge
        hfaudd , i(pnr) j(t)
```

```
                if `count' < 2012 reshape long efalle alder koen
loenmv pre_socio personindk dispon_13 aekvivadisp_13 hffsp merge  ,
i(pnr) j(t)

                gen count = t-year       //variable for time to
concussion
                gen treatment =1 //treatment group indicator

                save $data/sample_temp.dta, replace //temporary
dataset

                /
********************************************************
                Now build control sample for time `time' and year
`count'

*********************************************************/



                use pnr alder using $dorg/bef`t'          ///
Bring in all 30-49 yr olds
                        if inrange(alder`t',20,59), clear // from
the population register

                **year variable
                gen year = `w'   //time for concussion for control
group `time'

                **limit sample to those who suffer a concussion in
`t'
                merge 1:1 pnr year using $data/
concussion_`time'.dta, ///
                        keep(3) nogen

                        forvalue x=`n'/`c'{      //add
longitudinal data
                                merge 1:1 pnr using $dorg/
bef`x', ///
                                        keep(1 3) keepus(efalle
alder koen)      //add information on spouse,

                                                //age, and gender
                                rename _merge merge`x' //indicator
for whether in DK that year
                                if `x' < 2017{
                                        **Add salary and SES
information
                                        merge 1:m pnr using
$dorg/ind`x', ///        1:m to account for duplicates
                                                nogen keep(1 3)
keepus(erhvervsindk_13 pre_socio personindk dispon_13
aekvivadisp_13) // in data on non-important variables
```

```
                    bysort _all: keep if _n
==1                         //drop perfect
duplicates

                    **Align variable names
and account for inflation

                    rename erhvervsindk_13
loenmv
                    rename pre_socio
pre_socio`x'

                    foreach kk in personindk
dispon_13 aekvivadisp_13 loenmv{
                            rename `kk'
`kk'`x'
                    }

                    foreach kk in personindk
dispon_13 aekvivadisp_13 loenmv{
                            replace `kk'`x' =
        `kk'`x'/${price`x'}
                    }

                    **Bring in educational
information
                    merge 1:1 pnr using
$dorg/uddany`x', ///
                    nogen keep(1 3)
keepus(hffsp)
            }
        if `x' == 2017{
            merge 1:m pnr using $dorg/
ind`x', ///      m:1 to account for duplicates
                    nogen keep(1 3)
keepus(erhvervsindk_13 pre_socio personindk)      // in data on
non-important variables

            bysort _all: keep if _n ==1
                    //drop perfect duplicates
            **Align variable names and account
for inflation

            rename erhvervsindk_13 loenmv
            rename pre_socio pre_socio`x'

            foreach kk in personindk loenmv{
                            rename `kk'
`kk'`x'
            }

            foreach kk in personindk loenmv{

                            replace `kk'`x' =
```

```
        `kk'`x'/${price`x'}
                                }

                                **Bring in educational information
                                merge 1:1 pnr using $dorg/
udda`x', ///
                                        nogen keep(1 3)
keepus(hfaudd)
                                rename hfaudd hfaudd`x'
                        }
                }

                **Reshape data to panel structure
                if `count'>= 2012 reshape long efalle alder koen
loenmv pre_socio personindk dispon_13 aekvivadisp_13 hffsp merge
hfaudd , i(pnr) j(t)
                if `count' < 2012 reshape long efalle alder koen
loenmv pre_socio personindk dispon_13 aekvivadisp_13 hffsp merge  ,
i(pnr) j(t)
                gen count = t-`t'                //variable for
time to concussion for treatment
                gen control`time' =1     //control indicator
                save $data/control_temp, replace

                **Build sample with treatment and control `time'
for year `count'
                use $data/sample_temp
                append using $data/control_temp

                **fixes control and treatment indicators
                replace control`time' = 0 if control`time'==.
                replace treatment = 0 if treatment==.

                **Picks up changes to education variable
                if `count' >=2012{
                        tostring hfaudd, replace
                        rename hfaudd start
                        merge m:1 start using "\
\srvfsenas1\data\Formater\SAS formater i Danmarks
Statistik\STATA_datasaet\Disced\c_udd_niveau_l1l2_k.dta" , nogen
keep(1 3)
                        destring UDD, replace force
                        **Replace all with high scho0l degree or
higher in HFAUDD to have HFFSP = 40000001
                        replace hffsp = 40000001 if t == 2017 &
inrange(UDD,30,80)
                        replace hffsp = 0 if t == 2017 & !
inrange(UDD,30,80)
                        drop UDD start
                }

                sort pnr t
```

```
                    save $data/sample_control_`count'_`time'.dta,
replace

          }
}


forvalue time = 1/5{
          forvalue count =2003/2012{
                    if `time' ==1 & `count' ==2003 use $data/
sample_control_`count'_`time'.dta, clear
                    else append using $data/
sample_control_`count'_`time'.dta

                    if `time' ==5 & `count' ==2012 bysort pnr: keep if
_n ==1
                    if `time' ==5 & `count' ==2012  count
          }
}
```

For peer review only

```
clear

************************************************************************************
************************************************************************************
**
**              Calculate share of year on public benefits and size of benefit
**              payments for Fallesen and Campos (2020)
**
**
************************************************************************************
************************************************************************************
/*globals for price index to calculate income at 2015-level across years*/
/*Price index obtained from www.dst.dk/en/statistik/emner/priser-og-forbrug/forbrugeriser/nettoprisindeks */

global price1980 = .358
global price1981 = .398
global price1982 = .439
global price1983 = .466
global price1984 = .494
global price1985 = .517
global price1986 = .521
global price1987 = .537
global price1988 = .564
global price1989 = .594
global price1990 = .612
global price1991 = .628
global price1992 = .642
global price1993 = .651
global price1994 = .662
global price1995 = .674
global price1996 = .688
global price1997 = .703
global price1998 = .713
global price1999 = .728
global price2000 = .751
global price2001 = .769
global price2002 = .788
global price2003 = .806
global price2004 = .817
global price2005 = .833
global price2006 = .850
global price2007 = .867
global price2008 = .899
global price2009 = .917
global price2010 = .936
global price2011 = .960
global price2012 = .978
```

```
global price2013 = .986
global price2014 = .994
global price2015 =1.00
global price2016 =1.005
global price2017 =1.017


**Global for path to registry data
global dorg "e:/data/rawdata/706630"
*Global for processed data
global data "E:/data/workdata/706630/pf/FallesenCampos/data"

forvalue t=1996/2017{

        ** Read in data on social benefits recipiency share of
weeks
        ** from the DREAM database
        use $dorg/dream`t'
        gen share =0
        forvalue y = 1/52{
                        if `y' < 10 replace share = share+1 if
y_0`y' !=.
                        if `y' >  9 replace share = share+1 if
y_`y' !=.
                        if `y' < 10 drop y_0`y'
                        if `y' >  9 drop y_`y'

        }
        **Generate annual measure of share of year receiving social
benefits
        replace share = share/52
        keep pnr share
        gen t = `t'
        if `t' > 1996 append using $data/temp.dta
        save $data/temp.dta, replace

}


forvalue t=1998/2017{

        **Read in information on size of different types of social
benefits

        if `t' < 2002{
                use pnr syg_barsel_13 konthj arblhum pre_socio
using $dorg/ind`t'.dta, clear
                replace syg_barsel_13 = syg_barsel_13/${price`t'}
                replace konthj  = konthj /${price`t'}
                replace arblhum = arblhum/${price`t'}
                gen kont_dag = konthj+arblhum
                drop konthj arblhum
        }
        if `t' >= 2002 & `t' < 2013{
```

```
                use pnr syg_barsel_13 adagpagn konthj arblhum
pre_socio using $dorg/ind`t'.dta, clear
                replace syg_barsel_13 = syg_barsel_13/${price`t'}
                replace adagpagn = adagpagn/${price`t'}
                replace konthj  = konthj /${price`t'}
                replace arblhum = arblhum/${price`t'}
                gen kont_dag = konthj+arblhum
                drop konthj arblhum
        }
        if `t' >= 2013{
                use pnr syg_barsel_13 adagpagn dagpenge_kontant_13
pre_socio using $dorg/ind`t'.dta, clear
                replace syg_barsel_13 = syg_barsel_13/${price`t'}
                replace adagpagn = adagpagn/${price`t'}
                replace dagpenge_kontant_13  =
dagpenge_kontant_13 /${price`t'}
                gen kont_dag = dagpenge_kontant_13-syg_barsel_13
                drop dagpenge_kontant_13
        }
        gen t = `t'
        compress
        bysort pnr: keep if _n ==1
        if `t' > 1998 append using $data/temp2.dta
        if `t' == 2017{
                sort pnr t
        }
        save $data/temp2.dta, replace
}
```

iew only

```
clear all

*******************************************************************
***********
**
**              This program geenrates figerus and results for
Fallesen & Campos (2020)
**              study of concussion's impact on productivity
measured through
**              annual salary
**
*******************************************************************
***********

**Global for path to registry data
global dorg "E:/data/rawdata/706630"

*Global for processed data
global data "[home]/data"

*Global on figures
global highdef "[home]\highdef"



forvalue time = 1/5{              //for different control groups

        local post_period = 5     // local for number of years
observed post
                                                            //
concussion for exposure group

        local endtime = 2017-`post_period'        /*last year where
we allow for exposure event
                                                            to
occur, in order to have long enough control
                                                            period.
Defined by latest year available data*/

        **Matrixes to capture estimates
        matrix results   = J(15,6,.)      // For salary estimates
        matrix results_p = J(15,6,.)      // For Pr(salary=0)
estimates
        matrix t         = J(15,6,.)      // For time indicators

        forvalue count = 2003(1)`endtime'{
                use $data/sample_control_`count'_`time', clear

                gen female = koen==2

                qui{
                        gen edu =0
                        replace edu = 1 if inrange(hffsp,
20000000,39000000) | ///
```

```
(hffsp >40000000 & hffsp!=.)
                 }

                 **exclude individuals in years where they do not
appear in data,
                 **due to either death or migration, as well as
periods from when
                 **the control group sufer their concussion
                 drop if merge ==1 | count > `time'-1

                 **generate concussion variable
                 gen treat = inrange(count,0,`time'-1) & treatment
==1
                 replace treat = time_from_incident if count ==0 &
treatment ==1

                 **Generate pre-concussion income difference
for
                 **use in calculating marginal effects
                 sum loenmv if count <0 & treatment ==0
                 local control =r(mean)
                 sum loenmv if count <0 & treatment ==1
                 local treat =r(mean)
                 sum loenmv if count>=0 & treatment ==0
                 local control_post =r(mean)
                 gen post = count >=0

                 **estimate DiD model on salary
                 reghdfe loenmv treat, abs(alder female post
treatment edu year) cl(pnr)
                 matrix b =e(b)   //regression coefficient
                 matrix V = e(V)  // standard error^2
                 local n = `count'-2002   //time

                 matrix results[`n',1]    =      b[1,1]
                 matrix results[`n',2]    =      V[1,1]^.5
                 matrix results[`n',3]    =      b[1,1]/
(`control_post'-(`control'-`treat'))
                 matrix results[`n',4]    =      `n'


        }
        svmat results

        rename results1 est
        rename results2 se
        rename results3 marg
        rename results4 time

        replace time = time+(`time'-3)*.1 //jitter estimates for
graph
```

```
        keep est* se* marg* time
        keep if est !=.

        replace est = est/7446    //estimate measured as 1000 Euro
        replace se = se/7446      //S.E. measured as 1000 Euro

        gen upper = est+se*1.96 // Upper CI
        gen lower = est-se*1.96 // Lower CI


        gen control = `time'              //indicate control group

        if `time' >1 append using $data/results.dta
        save $data/results.dta, replace
}

use $data/results.dta, clear

replace time = 2002+time


*reads in unemployment statistcis obtained from statistikbanken.dk/
en/

gen unemp = 5.8 if time ==2003
replace unemp = 5.8 if time ==2004
replace unemp = 5.1 if time ==2005
replace unemp = 3.9 if time ==2006
replace unemp = 2.7 if time ==2007
replace unemp = 1.9 if time ==2008
replace unemp = 3.6 if time ==2009
replace unemp = 4.2 if time ==2010
replace unemp = 4.0 if time ==2011
replace unemp = 4.5 if time ==2012

scatter est time if control ==1, mcolor(navy) yaxis(1) ysc(range(-4
3) axis(1)) ylab(-4(1)3)  || ///
scatter est time if control ==2, mcolor(blue) || ///
scatter est time if control ==3, mcolor(midblue) || ///
scatter est time if control ==4, mcolor(gray) || ///
scatter est time if control ==5, mcolor(ltblue) || ///
rspike upper lower time if control ==1, lcolor(navy) || ///
rspike upper lower time if control ==2, lcolor(blue) || ///
rspike upper lower time if control ==3, lcolor(midblue) || ///
rspike upper lower time if control ==4, lcolor(gray) || ///
rspike upper lower time if control ==5, lcolor(ltblue) || ///
line unemp time , lcolor(black) yaxis(2) ysc(range(0 6) axis(2))
ylab(0(1)6, axis(2))  ///
xsc(range(2002.5 2012.5)) xlab(2003(2)2012) ///
yline(0) ysize(10) xsize(12) graphr(c(white)) ///
xti("Year of concussion for exposure group") scale(.95) ///
legend(label(1 " Control," "{&Delta}=1 yr") ///
label(2 " Control," "{&Delta}=2 yr") ///
label(3 " Control," "{&Delta}=3 yr") ///
```

```
label(4 " Control," "{&Delta}=4 yr") ///
label(5 " Control," "{&Delta}=5 yr") ///
label(6 "% Unemp." "of LF") ///
c(1) order(1 2 3 4 5 6) pos(3) size(small) ///
c(1) symx(4) region(lc(white))) ///
yti("Effect on Salary (in EUR 1K)", height(7) axis(1)) ///
yti("Percent of Full Time Uemployed among LF", height(7) axis(2))


graph export $highdef/marg_est.png, replace width(3900)


forvalue control_time=1/5{
        local end = 2012 // last incident year in data
        if `control_time' ==1    eststo clear

        **build dataset for joint estimate across years
        forvalue count=2003/`end'{
                if `count'==2003{
                        use $data/
sample_control_`count'_`control_time'.dta, clear
                        gen time = `count' //incident year
indicator
                }
                else append using $data/
sample_control_`count'_`control_time'.dta
                replace time = `count' if time ==.

                **exclude individuals in years where they do not
appear in data,
                **due to either death or migration, as well as
periods from when
                **the control group sufer their concussion
                drop if merge ==1 | count > `control_time'-1
        }

        gen female = koen==2

        //build ident, so we can multivariate cluster for
individuals
        //who occur both as control and exposure during the period
(id)

        bysort pnr time: gen helpx = _n ==1
        gen id= sum(helpx)
        drop helpx

        **Generate educational groups
        qui{
                gen edu =0
                replace edu = 1 if inrange(hffsp,20000000,39000000)
| ///
                                                                  (hffsp
>40000000 & hffsp!=.)
```

```
        }

        **Calculate number of observations for exposure and control
        count if count==0 & treatment ==1
        local Ntreated = r(N)
        count if count==0 & treatment ==0
        local Ncontrol = r(N)

        **generate concussion variable
        gen treat = inrange(count,0,`control_time'-1) & treatment
==1
        replace treat = time_from_incident if count ==0 & treatment
==1

        **Generate pre-concussion income difference        for
        **use in calculating marginal effects
        sum loenmv if count <0 & treatment ==0
        local control =r(mean)
        sum loenmv if count <0 & treatment ==1
        local treat =r(mean)
        sum loenmv if count>=0 & treatment ==0
        local control_post =r(mean)

        forvalue t=-4/4{
                local n = `t'*-1
                if `t' < -1 gen T_`n' = treatment ==1 & count ==`t'
                if `t' > -1 gen T`t' = treatment ==1 & count ==`t'

        }

        **estimate DiD model on salary
        reghdfe loenmv T*, abs(alder female count time treatment
edu) cl(pnr  id)
        eststo est1_`control_time'
        if `control_time'==1 matrix results = J(5,5,.) // matrix to
capture results
        if `control_time'==1 matrix results_p = J(5,5,.) // matrix
to capture results
        if `control_time'==1 matrix results_pre = J(5,5,.) //
matrix to capture results

        matrix b = e(b)
        matrix V = e(V)
        local n = `control_time'

        matrix results[`n',1]    = b[1,1] / 7466  // capture beta
results as 1K Euro
        matrix results[`n',2]    = (V[1,1]^.5)/7466       //
capture standard error as 1K Euro
        matrix results[`n',3]    =       b[1,1]/(`control_post'-
(`control'-`treat'))
        matrix results[`n',4]    =       `n'

        gen no_lon = loenmv<1 //dummy for no salary
```

```
		**estimate DiD LP-model for pre-trends
		xi: reghdfe loenmv T*, abs(alder female count time 
	treatment edu) cl(pnr  id), if count <0
		eststo est3_`control_time'
		matrix b =e(b)   //regression coefficient
		matrix V = e(V)  // standard error^2

		matrix results_pre[`n',1] = 	b[1,1]
		matrix results_pre[`n',2] = 	V[1,1]^.5
		matrix results_pre[`n',3] = 	b[1,1]/(`control_post'-
	(`control'-`treat'))
		matrix results_pre[`n',4] = 	`n'

	}

	esttab est1_* using [home]/tables/dynamic1.rtf, ///
		replace se(1) b(1) compress nogap star(+ .1 * .05 ** .01 
	*** .001) ///
		keep(T*)

	esttab est2_* using [home]/tables/dynamic2.rtf, ///
		replace se(3) b(3) compress nogap star(+ .1 * .05 ** .01 
	*** .001) ///
		keep(T*)

	esttab est3_* using [home]/tables/pre_trends.rtf, ///
		replace se(3) b(3) compress nogap star(+ .1 * .05 ** .01 
	*** .001) ///
		keep(T*)

	forvalue control_time=1/5{
		local end = 2012 // last incident year in data
		if `control_time' ==1	eststo clear
		qui{
			**build dataset for joint estimate across years
			forvalue count=2003/`end'{
				if `count'==2003{
					use $data/
	sample_control_`count'_`control_time'.dta, clear
					gen time = `count' //incident year 
	indicator
				}
				else append using $data/
	sample_control_`count'_`control_time'.dta
				replace time = `count' if time ==.

				**exclude individuals in years where they 
	do not appear in data,
				**due to either death or migration, as 
	well as periods from when
				**the control group sufer their concussion
```

```
                    drop if merge ==1 | count >
`control_time'-1
            }

            gen female = koen==2

                    **Generate educational groups
            gen edu =0
            replace edu = 1 if inrange(hffsp,20000000,39000000)
| ///

(hffsp >40000000 & hffsp!=.)


            //build ident, so we can multivariate cluster for
individuals
            //who occur both as control and exposure during the
period (id)

            bysort pnr time: gen helpx = _n ==1
            gen id= sum(helpx)
            drop helpx

            **Calculate number of observations for exposure and
control
            count if count==0 & treatment ==1
            local Ntreated = r(N)
            count if count==0 & treatment ==0
            local Ncontrol = r(N)

            **generate concussion variable
            gen treat = inrange(count,0,`control_time'-1) &
treatment ==1
            replace treat = time_from_incident if count ==0 &
treatment ==1

            **Generate pre-concussion income difference
for
            **use in calculating marginal effects
            sum loenmv if count <0 & treatment ==0
            local control =r(mean)
            sum loenmv if count <0 & treatment ==1
            local treat =r(mean)
            sum loenmv if count>=0 & treatment ==0
            local control_post =r(mean)

            forvalue t=-4/4{
                    local n = `t'*-1
                    if `t' < -1 gen T_`n' = treatment ==1 &
count ==`t'
                    if `t' > -1 gen T`t' = treatment ==1 &
count ==`t'

            }
```

```
                    gen post = count > -1

                    **estimate DiD model on salary
                    reghdfe loenmv treat, abs(alder female post time
treatment edu) cl(pnr id)
                    eststo est1_`control_time'
                    if `control_time'==1 matrix results = J(5,5,.) //
matrix to capture results
                    if `control_time'==1 matrix results_p = J(5,5,.) //
matrix to capture results


                    matrix b = e(b)
                    matrix V = e(V)
                    local n = `control_time'

                    matrix results[`n',1]     = b[1,1] / 7466  //
capture beta results as 1K Euro
                    matrix results[`n',2]     = (V[1,1]^.5)/
7466    // capture standard error as 1K Euro
                    matrix results[`n',3]     =       b[1,1]/
(`control_post'-(`control'-`treat'))
                    matrix results[`n',4]     =       `n'


          }
          *examining balance of samples
          di in ye `control_time'
          bysort treatment: sum female alder edu if count ==0

}



svmat results

gen upper = results1+results2*1.96
gen lower = results1-results2*1.96

scatter results1 results4 if results4 ==1, mcolor(navy) || ///
scatter results1 results4 if results4 ==2, mcolor(navy) || ///
scatter results1 results4 if results4 ==3, mcolor(navy) || ///
scatter results1 results4 if results4 ==4, mcolor(navy) || ///
scatter results1 results4 if results4 ==5, mcolor(navy) || ///
rspike upper lower results4 if results4 ==1, lcolor(navy) || ///
rspike upper lower results4 if results4 ==2, lcolor(navy) || ///
rspike upper lower results4 if results4 ==3, lcolor(navy) || ///
rspike upper lower results4 if results4 ==4, lcolor(navy) || ///
rspike upper lower results4 if results4 ==5, lcolor(navy)  ///
ysc(range(-2 1)) ylab(-2(.5)1) ///
xsc(range(.5 5.5)) xlab(1(1)5) ///
yline(0) ysize(10) xsize(12) graphr(c(white)) ///
xti("Years between exposure and control incident") scale(.95) ///
```

```
legend(label(1 " Control," "{&Delta}=1 yr") ///
label(2 " Control," "{&Delta}=2 yr") ///
label(3 " Control," "{&Delta}=3 yr") ///
label(4 " Control," "{&Delta}=4 yr") ///
label(5 " Control," "{&Delta}=5 yr") ///
c(1) order(1 2 3 4 5) pos(3) size(small) ///
c(1) symx(4) region(lc(white))) ///
yti("Effect in 1K Euro ({&delta}{subscript: Salary})",
height(7)) ///
legend(off)

graph export $highdef/est2003_2011.png, replace width(3900)


**Reports marginal effects for period 2003-2011 in percent

gen upper2 = (results3+results2/(results1/results3)*1.96)*100
gen lower2 = (results3-results2/(results1/results3)*1.96)*100
replace results3 = results3*100

scatter results3 results4 , mcolor(navy) || ///
rspike upper2 lower2 results4, lcolor(navy) ///
ysc(range(-5 0)) ylab(-5(.5)0) ///
xsc(range(.5 5.5)) xlab(1(1)5) ///
yline(0) ysize(10) xsize(12) graphr(c(white)) ///
/*title("Percentage change in salary, 2003-10")*/ ///
yti("Salary change (in %)", height(7)) ///
xti("Years between exposure and control incident") scale(.95) ///
legend(label(1 " Control," "{&Delta}=1 yr") ///
label(2 " Control," "{&Delta}=2 yr") ///
label(3 " Control," "{&Delta}=3 yr") ///
label(4 " Control," "{&Delta}=4 yr") ///
label(5 " Control," "{&Delta}=5 yr") ///
c(1) order(1 2 3 4 5) pos(3) size(small) ///
c(1) symx(4) region(lc(white)))  legend(off)
/*
note("Marginal effects for exposure dummy across spacing of control
groups. Decrease " ///
"calculated by dividing {&delta} with the normalized control groups'
average salary " ///
"post-concussion. Control groups suffer concussions 1, 2, 3, 4, and
5 years (&Delta) after" ///
"the exposure group. Both control and exposure group are 30-49 years
of age when" ///
"exposure group suffers concussion. 95% confidence intervals.")*/

graph export $highdef/marginal2003_2011.png, replace width(3900)




*******************************************************************
***********
**
```

```
**               Results for individuals with at least highschool
**
**
********************************************************************
***********


forvalue control_time=1/5{
        local end = 2012 // last incident year in data

        **build dataset for joint estimate across years
        forvalue count=2003/`end'{
                if `count'==2003{
                        use $data/
sample_control_`count'_`control_time'.dta, clear
                        gen time = `count' //incident year
indicator
                }
                else append using $data/
sample_control_`count'_`control_time'.dta
                replace time = `count' if time ==.

                **exclude individuals in years where they do not
appear in data,
                **due to either death or migration, as well as
periods from when
                **the control group sufer their concussion
                drop if merge ==1 | count > `control_time'-1


        }

        gen female = koen==2

        **Generate educational groups
        qui{
                gen edu =0
                replace edu = 1 if inrange(hffsp,20000000,39000000)
| ///
                                                                (hffsp
>40000000 & hffsp!=.)
        }

        keep if edu==1

        //build ident, so we can multivariate cluster for
individuals
        //who occur both as control and exposure during the period
(id)

        bysort pnr time: gen helpx = _n ==1
        gen id= sum(helpx)
        drop helpx help
```

```
        **Calculate number of observations for exposure and control
        count if count==0 & treatment ==1
        local Ntreated = r(N)
        count if count==0 & treatment ==0
        local Ncontrol = r(N)

        **generate concussion variable
        gen treat = inrange(count,0,`control_time'-1) & treatment
==1
        replace treat = time_from_incident if count ==0 & treatment
==1

        **Generate pre-concussion income difference        for
        **use in calculating marginal effects
        sum loenmv if count <0 & treatment ==0
        local control =r(mean)
        sum loenmv if count <0 & treatment ==1
        local treat =r(mean)
        sum loenmv if count>=0 & treatment ==0
        local control_post =r(mean)

        gen post = count >=0

        **estimate DiD model on salary
        xi: reghdfe loenmv treat, abs(alder female post time
treatment) cl(id pnr)

        if `control_time'==1 matrix results_edu = J(5,5,.) //
matrix to capture results
        if `control_time'==1 matrix results_p_edu = J(5,5,.) //
matrix to capture results


        matrix b = e(b)
        matrix V = e(V)
        local n = `control_time'

        matrix results_edu[`n',1]         = b[1,1] / 7466  //
capture beta results as 1K Euro
        matrix results_edu[`n',2]         = (V[1,1]^.5)/
7466    // capture standard error as 1K Euro
        matrix results_edu[`n',3] =       b[1,1]/(`control_post'-
(`control'-`treat'))
        matrix results_edu[`n',4] =       `n'


}

*********************************************************************
***********
**
**               Results for individuals with no high school+
**
**
```

```
****************************************************************************
***********


forvalue control_time=1/5{
        local end = 2012 // last incident year in data

        **build dataset for joint estimate across years
        forvalue count=2003/`end'{
                if `count'==2003{
                        use $data/
sample_control_`count'_`control_time'.dta, clear
                        gen time = `count' //incident year
indicator
                }
                else append using $data/
sample_control_`count'_`control_time'.dta
                replace time = `count' if time ==.

                **exclude individuals in years where they do not
appear in data,
                **due to either death or migration, as well as
periods from when
                **the control group sufer their concussion
                drop if merge ==1 | count > `control_time'-1


        }

        gen female = koen==2

        //build ident, so we can multivariate cluster for
individuals
        //who occur both as control and exposure during the period
(id)

        bysort pnr time: gen helpx = _n ==1
        gen id= sum(helpx)
        drop helpx help

        **Generate educational groups
        qui{
                gen edu =0
                replace edu = 1 if inrange(hffsp,20000000,39000000)
| ///
                                                                    (hffsp
>40000000 & hffsp!=.)
        }

        keep if edu==0



        **Calculate number of observations for exposure and control
```

```
        count if count==0 & treatment ==1
        local Ntreated = r(N)
        count if count==0 & treatment ==0
        local Ncontrol = r(N)

        **generate concussion variable
        gen treat = inrange(count,0,`control_time'-1) & treatment
==1
        replace treat = time_from_incident if count ==0 & treatment
==1

        **Generate pre-concussion income difference       for
        **use in calculating marginal effects
        sum loenmv if count <0 & treatment ==0
        local control =r(mean)
        sum loenmv if count <0 & treatment ==1
        local treat =r(mean)
        sum loenmv if count>=0 & treatment ==0
        local control_post =r(mean)
        gen post = count >=0

        **estimate DiD model on salary
        xi: reghdfe loenmv treat, abs(alder female post time
treatment) cl(id pnr)

        if `control_time'==1 matrix results_noedu = J(5,5,.) //
matrix to capture results
        if `control_time'==1 matrix results_p_noedu = J(5,5,.) //
matrix to capture results


        matrix b = e(b)
        matrix V = e(V)
        local n = `control_time'

        matrix results_noedu[`n',1]      = b[1,1] / 7466  //
capture beta results as 1K Euro
        matrix results_noedu[`n',2]      = (V[1,1]^.5)/
7466    // capture standard error as 1K Euro
        matrix results_noedu[`n',3]      =       b[1,1]/
(`control_post'-(`control'-`treat'))
        matrix results_noedu[`n',4]      =       `n'



}
**********************************************************************
***********
**
**      Draw figure for subgroups
**
**
**********************************************************************
***********
```

```
local t= -.15

foreach x in noedu edu{
        svmat results_`x'
        replace results_`x'4= results_`x'4+`t'
        svmat results_p_`x'
        replace results_p_`x'4= results_p_`x'4+`t'

        gen upper`x' = results_`x'1+results_`x'2*1.96
        gen lower`x' = results_`x'1-results_`x'2*1.96

        gen upper2`x' = (results_`x'3+results_`x'2/(results_`x'1/
results_`x'3)*1.96)*100
        gen lower2`x' = (results_`x'3-results_`x'2/(results_`x'1/
results_`x'3)*1.96)*100
        replace results_`x'3 = results_`x'3*100

        gen upper_p_`x' = results_p_`x'1+results_p_`x'2*1.96
        gen lower_p_`x' = results_p_`x'1-results_p_`x'2*1.96

        local t = `t'+.1

}


keep results* upper* lower*
keep if _n <=5

**generate locals for figure

foreach x in noedu edu{
        if "`x'" == "nopay" local color = "navy"
        if "`x'" == "pay"        local color = "red"
        if "`x'" == "noedu" local color = "green"
        if "`x'" == "edu"        local color = "purple"

        local figure_`x' "scatter results_`x'1 results_`x'4,
mcolor(`color') || rspike upper`x' lower`x' results_`x'4,
lcolor(`color') vertical"
        if "`x'" == "nopay" local figure2_`x' "scatter results_`x'3
results_`x'4, mcolor(`color') || rspike upper2`x' lower2`x'
results_`x'4, lcolor(`color' )  vertical "
        else local figure2_`x' "scatter results_`x'3 results_`x'4,
mcolor(`color') || rspike upper2`x' lower2`x' results_`x'4,
lcolor(`color')  vertical "
        local figure_p_`x' "scatter results_p_`x'1 results_p_`x'4,
mcolor(`color') || rspike upper_p_`x' lower_p_`x' results_p_`x'4,
lcolor(`color')  vertical "
}


`figure_noedu' || `figure_edu'  ///
legend( ///
```

```
label(1 "Less than" "high school") ///
label(3 "At least" "high school") ///
c(1) order(1 3 ) pos(3) size(small) ///
c(1) symx(4) region(lc(white))) ///
ysc(range(-4 2)) ylab(-4(1)2) ///
xsc(range(.5 5.5)) xlab(1(1)5) ///
yline(0) ysize(10) xsize(12) graphr(c(white)) ///
///title("Parameter estimates across control group, 2003-10") ///
xti("Years between exposure and control incident") scale(.95) ///
yti("Effect in 1K Euro ({&delta}{subscript: Salary})",
height(7)) ///
/*note("Parameter estimates for exposure dummy across spacing of
control groups." ///
"Control groups suffer concussions 1, 2, 3, 4, and 5 years (&Delta)
after the exposure group." ///
"Both control and exposure group are 30-49 years of age when
exposure group suffers " ///
"concussion. 95% confidence intervals.")
*/

graph export $highdef/grouped_est2003_2011.png, replace width(3900)

preserve



`figure2_noedu' || `figure2_edu'  ///
legend( ///
label(1 "Less than" "high school") ///
label(3 "At least" "high school") ///
c(1) order(1 3 5) pos(3) size(small) ///
c(1) symx(4) region(lc(white))) ///
ysc(range(-12 3)) ylab(-12(3)3) ///
xsc(range(.5 5.5)) xlab(1(1)5) ///
yline(0) ysize(10) xsize(12) graphr(c(white)) ///
/// title("Percentage change in salary, 2003-10") ///
yti("Salary change (in %)", height(7)) ///
xti("Years between exposure and control incident") scale(.95) ///
/*note("Marginal effects for exposure dummy across spacing of
control groups." ///
"change calculated by {&delta} with the normalized control groups'
average" ///
"salary post-concussion. Control groups suffer concussions 1, 2, 3,
4, and 5 years (&Delta) after" ///
"the exposure group. Both control and exposure group are 30-49 years
of age when" ///
"exposure group suffers concussion.  95% confidence intervals.")*/

graph export $highdef/grouped_marginal2003_2011.png, replace
width(3900)

restore
```

```
**********************************************************************
***********
**
**              Results different age-groups
**
**
**********************************************************************
***********

forvalue y=20(5)55{
        forvalue control_time=1/5{
                local end = 2012 // last incident year in data

                **build dataset for joint estimate across years
                forvalue count=2003/`end'{
                        if `count'==2003{
                                use $data/
sample_control_`count'_`control_time'.dta, clear
                                gen time = `count' //incident year
indicator
                        }
                        else append using $data/
sample_control_`count'_`control_time'.dta
                        replace time = `count' if time ==.

                        **exclude individuals in years where they
do not appear in data,
                        **due to either death or migration, as
well as periods from when
                        **the control group sufer their concussion
                        drop if merge ==1 | count >
`control_time'-1


                }

                gen female = koen==2

                //build ident, so we can multivariate cluster for
individuals
                //who occur both as control and exposure during the
period (id)

                bysort pnr time: gen helpx = _n ==1
                gen id= sum(helpx)
                drop helpx help

                gen nopay = loenmv <1

                **Generate age group
                local z = `y'+4
                gen help = count == 0 & inrange(alder,`y',`z')
                bysort id: egen helpx =max(help)
```

```
                    keep if helpx == 1
                    drop helpx help




                    **Calculate number of observations for exposure and
control
                    count if count==0 & treatment ==1
                    local Ntreated = r(N)
                    count if count==0 & treatment ==0
                    local Ncontrol = r(N)

                    **generate concussion variable
                    gen treat = inrange(count,0,`control_time'-1) &
treatment ==1
                    replace treat = time_from_incident if count ==0 &
treatment ==1

                    **Generate pre-concussion income difference
for
                    **use in calculating marginal effects
                    sum loenmv if count <0 & treatment ==0
                    local control =r(mean)
                    sum loenmv if count <0 & treatment ==1
                    local treat =r(mean)
                    sum loenmv if count>=0 & treatment ==0
                    local control_post =r(mean)
                    gen post = count >=0

                    **estimate DiD model on salary
                    xi: reghdfe loenmv treat, abs(alder female post
time treatment) cl(id pnr)

                    if `control_time'==1 matrix results_`y' =
J(5,5,.) // matrix to capture results
                    if `control_time'==1 matrix results_p_`y' =
J(5,5,.) // matrix to capture results


                    matrix b = e(b)
                    matrix V = e(V)
                    local n = `control_time'

                    matrix results_`y'[`n',1]        = b[1,1] /
7466    // capture beta results as 1K Euro
                    matrix results_`y'[`n',2]        = (V[1,1]^.5)/
7466    // capture standard error as 1K Euro
                    matrix results_`y'[`n',3] =      b[1,1]/
(`control_post'-(`control'-`treat'))
                    matrix results_`y'[`n',4] =      `n'


          }
```

```
}


local t = -.15   //Jitter estimates along x-axis
forvalue x =20(5)55{
        svmat results_`x'
        replace results_`x'4= results_`x'4+`t'
        svmat results_p_`x'
        replace results_p_`x'4= results_p_`x'4+`t'

        gen upper`x' = results_`x'1+results_`x'2*1.96
        gen lower`x' = results_`x'1-results_`x'2*1.96

        gen upper2`x' = (results_`x'3+results_`x'2/(results_`x'1/
results_`x'3)*1.96)*100
        gen lower2`x' = (results_`x'3-results_`x'2/(results_`x'1/
results_`x'3)*1.96)*100
        replace results_`x'3 = results_`x'3*100

        gen upper_p_`x' = results_p_`x'1+results_p_`x'2*1.96
        gen lower_p_`x' = results_p_`x'1-results_p_`x'2*1.96

        local t = `t'+.1

}

keep results* upper* lower*
keep if _n <=5

**generate locals for figure

forvalue x =20(5)55{
        if `x' == 20 local color = "black"
        if `x' == 25 local color = "orange"
        if `x' == 30 local color = "navy"
        if `x' == 35 local color = "red"
        if `x' == 40 local color = "green"
        if `x' == 45 local color = "purple"
        if `x' == 50 local color = "sienna"
        if `x' == 55 local color = "teal"

        local figure_`x' "scatter results_`x'1 results_`x'4,
mcolor(`color') || rspike upper`x' lower`x' results_`x'4,
lcolor(`color') vertical"
        local figure2_`x' "scatter results_`x'3 results_`x'4,
mcolor(`color') || rspike upper2`x' lower2`x' results_`x'4,
lcolor(`color')  vertical "
        local figure_p_`x' "scatter results_p_`x'1 results_p_`x'4,
mcolor(`color') || rspike upper_p_`x' lower_p_`x' results_p_`x'4,
lcolor(`color')  vertical "
}

/**/
```

```
`figure_20' || `figure_25' || `figure_30' || `figure_35' ///
        || `figure_40' || `figure_45' || `figure_50' ||
`figure_55'  ///
legend( ///
label(1 "Age 20-24") ///
label(3 "Age 25-29") ///
label(5 "Age 30-34") ///
label(7 "Age 35-39") ///
label(9 "Age 40-44") ///
label(11 "Age 45-49") ///
label(13 "Age 50-54") ///
label(15 "Age 55-59") ///
c(1) order(1 3 5 7 9 11 13 15) pos(3) size(small) ///
c(1) symx(4) region(lc(white))) ///
ysc(range(-4 2)) ylab(-4(1)2) ///
xsc(range(.5 5.5)) xlab(1(1)5) ///
yline(0) ysize(10) xsize(12) graphr(c(white)) ///
/// title("Parameter estimates across control group, 2003-10") ///
xti("Years between exposure and control incident") scale(.95) ///
yti("Effect in 1K Euro ({&delta}{subscript: Salary})",
height(7)) ///
/*note("Parameter estimates for exposure dummy across spacing of
control groups." ///
"Control groups suffer concussions 1, 2, 3, 4, and 5 years (&Delta)
after the exposure group." ///
"Age group described age at time of exposure incident. 95%
confidence intervals.")*/

graph export $highdef/age_est2003_2011.png, replace width(3900)
*/

`figure2_20' || `figure2_25' || `figure2_30' || `figure2_35' ///
        || `figure2_40' || `figure2_45' || `figure2_50' ||
`figure2_55'  ///
legend( ///
label(1 "Age 20-24") ///
label(3 "Age 25-29") ///
label(5 "Age 30-34") ///
label(7 "Age 35-39") ///
label(9 "Age 40-44") ///
label(11 "Age 45-49") ///
label(13 "Age 50-54") ///
label(15 "Age 55-59") ///
c(1) order(1 3 5 7 9 11 13 15) pos(3) size(small) ///
c(1) symx(4) region(lc(white))) ///
ysc(range(-12 3)) ylab(-12(3)3) ///
xsc(range(.5 5.5)) xlab(1(1)5) ///
yline(0) ysize(10) xsize(12) graphr(c(white)) ///
yti("Salary change (in %)", height(7)) ///
xti("Years between exposure and control incident") scale(.95)


graph export $highdef/age_marginal2003_2011.png, replace width(3900)
```

```
`figure_p_30' || `figure_p_35' || `figure_p_40' ||
`figure_p_45'  ///  ///
legend(label(1 "Age 30-34") ///
label(3 "Age 35-39") ///
label(5 "Age 40-44") ///
label(7 "Age 45-49") ///
c(1) order(1 3 5 7) pos(3) size(small) ///
c(1) symx(4) region(lc(white))) ///
ysc(range(-.02 .050)) ylab(-0.02(.01)0.05) ///
xsc(range(.5 5.5)) xlab(1(1)5) ///
yline(0, lcolor(black)) ysize(10) xsize(12) graphr(c(white)) ///
title("Parameter estimates across control group, 2003-10") ///
yti("Effect on Pr(Salary=0)", height(7)) ///
xti("Years between exposure and control incident ({&Delta})")
scale(.95) ///
note("Parameter estimates for exposure dummy across spacing of
control groups." ///
"Control groups suffer concussions 1, 2, 3, 4, and 5 (&Delta) years
after the exposure group." ///
"Age group described age at time of exposure incident. 95%
confidence intervals.")

graph export $highdef/age_nopay2003_2011.png, replace width(3900)


********************************************************************
***********
**
**                Results accross gender
**
**
********************************************************************
***********

forvalue y=0/1{
        forvalue control_time=1/5{
                local end = 2012 // last incident year in data

                **build dataset for joint estimate across years
                forvalue count=2003/`end'{
                        if `count'==2003{
                                use $data/
sample_control_`count'_`control_time'.dta, clear
                                gen time = `count' //incident year
indicator
                        }
                        else append using $data/
sample_control_`count'_`control_time'.dta
                        replace time = `count' if time ==.

                        **exclude individuals in years where they
do not appear in data,
                        **due to either death or migration, as
well as periods from when
```

```
                **the control group sufer their concussion
                drop if merge ==1 | count >
`control_time'-1


        }

        gen female = koen==2

        //build ident, so we can multivariate cluster for
individuals
        //who occur both as control and exposure during the
period (id)

        bysort pnr time: gen helpx = _n ==1
        gen id= sum(helpx)
        drop helpx help

        gen nopay = loenmv <1

        **Generate age group
        local z = `y'+4
        gen help = count == 0 & female==`y'
        bysort id: egen helpx =max(help)
        keep if helpx == 1
        drop helpx help




        **Calculate number of observations for exposure and
control
        count if count==0 & treatment ==1
        local Ntreated = r(N)
        count if count==0 & treatment ==0
        local Ncontrol = r(N)

        **generate concussion variable
        gen treat = inrange(count,0,`control_time'-1) &
treatment ==1
        replace treat = time_from_incident if count ==0 &
treatment ==1

        **Generate pre-concussion income difference
for
        **use in calculating marginal effects
        sum loenmv if count <0 & treatment ==0
        local control =r(mean)
        sum loenmv if count <0 & treatment ==1
        local treat =r(mean)
        sum loenmv if count>=0 & treatment ==0
        local control_post =r(mean)
        gen post = count >=0
```

```
                **estimate DiD model on salary
                xi: reghdfe loenmv treat, abs(alder post time
treatment) cl(id pnr)

                if `control_time'==1 matrix results_`y' =
J(5,5,.) // matrix to capture results
                if `control_time'==1 matrix results_p_`y' =
J(5,5,.) // matrix to capture results


                matrix b = e(b)
                matrix V = e(V)
                local n = `control_time'

                matrix results_`y'[`n',1]         = b[1,1] /
7466    // capture beta results as 1K Euro
                matrix results_`y'[`n',2]         = (V[1,1]^.5)/
7466    // capture standard error as 1K Euro
                matrix results_`y'[`n',3] =       b[1,1]/
(`control_post'-(`control'-`treat'))
                matrix results_`y'[`n',4] =       `n'


        }
}



local t = -.05   //Jitter estimates along x-axis
foreach x in 0 1{
        svmat results_`x'
        replace results_`x'4= results_`x'4+`t'
        svmat results_p_`x'
        replace results_p_`x'4= results_p_`x'4+`t'

        gen upper`x' = results_`x'1+results_`x'2*1.96
        gen lower`x' = results_`x'1-results_`x'2*1.96

        gen upper2`x' = (results_`x'3+results_`x'2/(results_`x'1/
results_`x'3)*1.96)*100
        gen lower2`x' = (results_`x'3-results_`x'2/(results_`x'1/
results_`x'3)*1.96)*100
        replace results_`x'3 = results_`x'3*100

        gen upper_p_`x' = results_p_`x'1+results_p_`x'2*1.96
        gen lower_p_`x' = results_p_`x'1-results_p_`x'2*1.96

        local t = `t'+.1

}

keep results* upper* lower*
keep if _n <=5
```

```
**generate locals for figure

foreach x in 0 1{
        if `x' == 0 local color = "red"
        if `x' == 1 local color = "green"

        local figure_`x' "scatter results_`x'1 results_`x'4,
mcolor(`color') || rspike upper`x' lower`x' results_`x'4,
lcolor(`color') vertical"
        local figure2_`x' "scatter results_`x'3 results_`x'4,
mcolor(`color') || rspike upper2`x' lower2`x' results_`x'4,
lcolor(`color')  vertical "
        local figure_p_`x' "scatter results_p_`x'1 results_p_`x'4,
mcolor(`color') || rspike upper_p_`x' lower_p_`x' results_p_`x'4,
lcolor(`color')  vertical "
}


`figure_0' || `figure_1'  ///
legend(label(1 "Men") ///
label(3 "Women") ///
c(1) order(1 3) pos(3) size(small) ///
c(1) symx(4) region(lc(white))) ///
ysc(range(-4 2)) ylab(-4(1)2) ///
xsc(range(.5 5.5)) xlab(1(1)5) ///
yline(0) ysize(10) xsize(12) graphr(c(white)) ///
/// title("Parameter estimates across control group, 2003-10") ///
xti("Years between exposure and control incident") scale(.95) ///
yti("Effect in 1K Euro ({&delta}{subscript: Salary})",
height(7)) ///
/*note("Parameter estimates for exposure dummy across spacing of
control groups." ///
"Control groups suffer concussions 1, 2, 3, 4, and 5 years (&Delta)
after the exposure group." ///
"95% confidence intervals.")*/

graph export $highdef/gender_est2003_2011.png, replace width(3900)

`figure2_0' || `figure2_1'   ///
legend( ///
label(1 "Men") ///
label(3 "Women") ///
c(1) order(1 3 5 7) pos(3) size(small) ///
c(1) symx(4) region(lc(white))) ///
ysc(range(-12 3)) ylab(-12(3)3) ///
xsc(range(.5 5.5)) xlab(1(1)5) ///
yline(0) ysize(10) xsize(12) graphr(c(white)) ///
/// title("Percentage change in salary, 2003-10") ///
yti("Salary change (in %)", height(7)) ///
xti("Years between exposure and control incident") scale(.95) ///
/* note("Parameter estimates for exposure dummy across spacing of
control groups." ///
"Control groups suffer concussions 1, 2, 3, 4, and 5 years (&Delta)
after the exposure group." ///
```

```
"95% confidence intervals.")*/

graph export $highdef/gender_marginal2003_2011.png, replace
width(3900)

`figure_p_0' || `figure_p_1'   ///
legend( ///
label(1 "Men") ///
label(3 "Women") ///
c(1) order(1 3 5 7) pos(3) size(small) ///
c(1) symx(4) region(lc(white))) ///
ysc(range(-.02 .050)) ylab(-0.02(.01)0.05) ///
xsc(range(.5 5.5)) xlab(1(1)5) ///
yline(0, lcolor(black)) ysize(10) xsize(12) graphr(c(white)) ///
/// title("Parameter estimates across control group, 2003-10") ///
yti("Effect on Pr(Salary=0)", height(7)) ///
xti("Years between exposure and control incident ({&Delta})")
scale(.95) ///
/*note("Parameter estimates for exposure dummy across spacing of
control groups." ///
"Control groups suffer concussions 1, 2, 3, 4, and 5 years (&Delta)
after the exposure group." ///
"95% confidence intervals.")*/

graph export $highdef/gender_nopay2003_2011.png, replace width(3900)


*********************************************************************
************
**
**                         Generate descriptive figures of wage
development for exposure
**                          and control group
**
**
*********************************************************************
************


 forvalue control_time=1/5{
        local end = 2012 // last incident year in data

        **build dataset for joint estimate across years
        forvalue count=2003/`end'{
                if `control_time' ==1 & `count'==2003{
                        use $data/
sample_control_`count'_`control_time'.dta, clear
                        gen time = `count' //incident year
indicator
                }
                else append using $data/
sample_control_`count'_`control_time'.dta

                **Drops exposure-group already in the data
```

```
                if `control_time' > 1 drop if time ==. & treatment
==1
                replace time = `count' if time ==.
                replace control1 = `control_time' if
control`control_time'==1
                if `control_time' > 1 drop control`control_time'

                **exclude individuals in years where they do not
appear in data,
                **due to either death or migration, as well as
periods from when
                **the control group suffer their concussion

        }

}

**Generate mean salary for each period relative to exposure groups
concussion
**separately for exposure and each control group

bysort control1 count: egen mean_loen = mean(loenmv)

**Generate mean Pr(sal= for each period relative to exposure groups
concussion
**separately for exposure and each control group

gen no_lon = loenmv < 1

bysort control1 count: egen mean_no_lon = mean(no_lon)

**generate group size
bysort control1 count: gen Ncount=_N if count ==0

**generate pre-exposure mean levels for normalization
gen pre = count <0
bysort control1 pre: egen pre_mean_loen = mean(loenmv) if pre==1
bysort control1 pre: egen pre_mean_no_lon = mean(no_lon) if pre==1


**reduce data set size
bysort control1 count: keep if _n ==1

keep count mean* pre_* control1 Ncount

**standardize to 1k euro
replace mean_loen = mean_loen/7466
replace pre_mean_loen = pre_mean_loen/7466

sort control1 count


**Pre-tratment normalization of salary
```

```
gen norm_mean_lon =mean_loen
gen norm_mean_no_lon =mean_no_lon

forvalue t = 1/5{
        qui sum pre_mean_loen if control == 0
        local treat = r(mean)
        qui sum pre_mean_loen if control == `t'
        local control = r(mean)

        **normalize with pre-concussion difference
        qui replace norm_mean_lon = mean_loen - (`control'-`treat')
if control ==`t'

        qui sum pre_mean_no_lon if control == 0
        local treat = r(mean)
        qui sum pre_mean_no_lon if control == `t'
        local control = r(mean)

        **normalize with pre-concussion difference
        qui replace norm_mean_no_lon = mean_no_lon - (`control'-
`treat') if control ==`t'

}

**local indicators of group sizes
forvalue t=0/5{
        qui sum Ncount if control == `t'
        local C`t' = r(mean)
}


graph twoway ///
        connect mean_l count if control1== 0, ///
        lcolor(black) mcolor(black) || ///
        connect mean_l count if control1== 1, ///
        lcolor(blue)  mcolor(blue) || ///
        connect mean_l count if control1== 2, ///
        lcolor(green) mcolor(green) || ///
        connect mean_l count if control1== 3, ///
        lcolor(purple) mcolor(purple) || ///
        connect mean_l count if control1== 4, ///
        lcolor(red) mcolor(red) || ///
        connect mean_l count if control1== 5, ///
        lcolor(orange) mcolor(orange) ///
        legend( ///
                label(1 "Exposure" ///
                                "N=`C0'") ///
                label(2 "Control {&Delta}=1" ///
                                "N=`C1'") ///
                label(3 "Control {&Delta}=2" ///
                                "N=`C2'") ///
                label(4 "Control {&Delta}=3" ///
                                "N=`C3'") ///
                label(5 "Control {&Delta}=4" ///
```

```
                                "N=`C4'") ///
                    label(6 "Control {&Delta}=5" ///
                                "N=`C5'") ///
                    c(1) order(1 2 3 4 5 6) pos(3) size(small) ///
                    c(1) symx(4) region(lc(white))) ///
            ysc(range(26 33)) ylab(26(1)33) ///
            xsc(range(-5 5)) xlab(-5(1)5) ///
            xline(0, lcolor(red)) ysize(10) xsize(12)
graphr(c(white)) ///
///         title("Impact of Concussion on Salary") ///
            yti("Deflated Salaried Income in 1K EUR", height(7)) ///
            xti("Years since exposure group concussion") scale(.95) ///
/*          note("Control groups suffer concussion 1, 2, 3, 4, and 5
years (&Delta) after exposure group." ///
                    "Both control and exposure group at 30-49 years of
age at beginning of year =0." ///
                    "Vertical line indicates time of exposure group
concussion.")*/

graph export $highdef/FigureS2.png, replace width(3900)


graph twoway ///
            connect norm_mean_l count if control1== 1, ///
            lcolor(blue)  mcolor(blue) || ///
            connect norm_mean_l count if control1== 2, ///
            lcolor(green) mcolor(green) || ///
            connect norm_mean_l count if control1== 3, ///
            lcolor(purple) mcolor(purple) || ///
            connect norm_mean_l count if control1== 4, ///
            lcolor(red) mcolor(red) || ///
            connect norm_mean_l count if control1== 5, ///
            lcolor(orange) mcolor(orange) || ///
            connect mean_l count if control1== 0, ///
            lcolor(black) mcolor(black)  ///
            legend( ///
                    label(1 "Control {&Delta}=1" ///
                                "N=`C1'") ///
                    label(2 "Control {&Delta}=2" ///
                                "N=`C2'") ///
                    label(3 "Control {&Delta}=3" ///
                                "N=`C3'") ///
                    label(4 "Control {&Delta}=4" ///
                                "N=`C4'") ///
                    label(5 "Control {&Delta}=5" ///
                                "N=`C5'") ///
                    label(6 "Exposure" ///
                                "N=`C0'") ///
                    c(1) order(6 1 2 3 4 5) pos(3) size(small) ///
                    c(1) symx(4) region(lc(white))) ///
            ysc(range(26 31)) ylab(26(1)31) ///
            xsc(range(-5 5)) xlab(-5(1)5) ///
            xline(0, lcolor(red)) ysize(10) xsize(12)
graphr(c(white)) ///
```

```
///     title("Impact of Concussion on Salary") ///
        yti("Deflated Norm. Salaried Income in 1K EUR",
height(7)) ///
        xti("Years since exposure group's concussion") scale(.95)
        /*///
        note("Control groups suffer concussion 1, 2, 3, 4, and 5
years (&Delta) after exposure group." ///
                "Both control and exposure group at 30-49 years of
age at beginning of year =0." ///
                "Vertical line indicates time of exposure group
concussion." ///
                "Salary levels normalized with pre-concussion level
difference between" ///
                "each control group and the exposure group")*/

graph export $highdef/Figure2.png, replace width(3900)


graph twoway ///
        connect mean_no_l count if control1== 0, ///
        lcolor(black) mcolor(black) || ///
        connect mean_no_l count if control1== 1, ///
        lcolor(blue)  mcolor(blue) || ///
        connect mean_no_l count if control1== 2, ///
        lcolor(green) mcolor(green) || ///
        connect mean_no_l count if control1== 3, ///
        lcolor(purple) mcolor(purple) || ///
        connect mean_no_l count if control1== 4, ///
        lcolor(red) mcolor(red) || ///
        connect mean_no_l count if control1== 5, ///
        lcolor(orange) mcolor(orange) ///
        legend( ///
                label(1 "Exposure" ///
                                 "N=`C0'") ///
                label(2 "Control {&Delta}=1" ///
                                 "N=`C1'") ///
                label(3 "Control {&Delta}=2" ///
                                 "N=`C2'") ///
                label(4 "Control {&Delta}=3" ///
                                 "N=`C3'") ///
                label(5 "Control {&Delta}=4" ///
                                 "N=`C4'") ///
                label(6 "Control {&Delta}=5" ///
                                 "N=`C5'") ///
                c(1) order(1 2 3 4 5 6) pos(3) size(small) ///
                c(1) symx(4) region(lc(white))) ///
        ysc(range(.2 .325)) ylab(.2(.025).325) ///
        xsc(range(-5 5)) xlab(-5(1)5) ///
        xline(0, lcolor(red)) ysize(10) xsize(12)
graphr(c(white)) ///
///     title("Impact of Concussion on Prob(Salary=0)") ///
        yti("Prob(Salary=0)", height(7)) ///
        xti("Years since exposure group concussion") scale(.95) ///
/*      note("Control groups suffer concussion 1, 2, 3, 4, and 5
```

```
years (&Delta) after exposure group." ///
                  "Both control and exposure group at 30-49 years of
age at beginning of year =0." ///
                  "Vertical line indicates time of exposure group
concussion.")*/

graph export $highdef/FigureS3.png, replace width(3900)

graph twoway ///
        connect norm_mean_no_l count if control1== 1, ///
        lcolor(blue)  mcolor(blue) || ///
        connect norm_mean_no_l count if control1== 2, ///
        lcolor(green) mcolor(green) || ///
        connect norm_mean_no_l count if control1== 3, ///
        lcolor(purple) mcolor(purple) || ///
        connect norm_mean_no_l count if control1== 4, ///
        lcolor(red) mcolor(red) || ///
        connect norm_mean_no_l count if control1== 5, ///
        lcolor(orange) mcolor(orange) || ///
        connect mean_no_l count if control1== 0, ///
        lcolor(black) mcolor(black) ///
        legend( ///
                label(1 "Control {&Delta}=1" ///
                                 "N=`C1'") ///
                label(2 "Control {&Delta}=2" ///
                                 "N=`C2'") ///
                label(3 "Control {&Delta}=3" ///
                                 "N=`C3'") ///
                label(4 "Control {&Delta}=4" ///
                                 "N=`C4'") ///
                label(5 "Control {&Delta}=5" ///
                                 "N=`C5'") ///
                label(6 "Exposure" ///
                                 "N=`C0'") ///
                c(1) order(6 1 2 3 4 5) pos(3) size(small) ///
                c(1) symx(4) region(lc(white))) ///
        ysc(range(.2 .325)) ylab(.2(.025).325) ///
        xsc(range(-5 5)) xlab(-5(1)5) ///
        xline(0, lcolor(red)) ysize(10) xsize(12)
graphr(c(white)) ///
///     title("Impact of Concussion on Prob(Salary=0)") ///
        yti("Norm. Prob(Salary=0)", height(7)) ///
        xti("Years since exposure group concussion") scale(.95) ///
/*      note("Control groups suffer concussion 1, 2, 3, 4, and 5
years (&Delta) after exposure group." ///
                  "Both control and exposure group at 30-49 years of
age at beginning of year =0." ///
                  "Vertical line indicates time of exposure group
concussion." ///
                  "Probability levels normalized with pre-concussion
level difference between" ///
                  "each control group and the exposure group")*/

graph export $highdef/Figure2A.png, replace width(3900)
```

```
*********************************************************************
************
**
**      Effects across the salary distribution
**
**
**
*********************************************************************
************


forvalue control_time=5/5{
        local end = 2012 // last incident year in data

        **build dataset for joint estimate across years
        forvalue count=2003/`end'{
                if `count'==2003{
                        use $data/
sample_control_`count'_`control_time'.dta, clear
                        gen time = `count' //incident year
indicator
                }
                else append using $data/
sample_control_`count'_`control_time'.dta
                replace time = `count' if time ==.

                **exclude individuals in years where they do not
appear in data,
                **due to either death or migration, as well as
periods from when
                **the control group sufer their concussion
                drop if merge ==1 | count > `control_time'-1
        }

        gen female = koen==2
        qui{
                gen edu =0
                replace edu = 1 if inrange(hffsp,20000000,39000000)
| ///
                                                                (hffsp
>40000000 & hffsp!=.)
        }

        //build ident, so we can multivariate cluster for
individuals
        //who occur both as control and exposure during the period
(id)
```

```
        bysort pnr time: gen helpx = _n ==1
        gen id= sum(helpx)
        drop helpx


        **generate concussion variable
        gen treat = inrange(count,0,`control_time'-1) & treatment
==1
        replace treat = time_from_incident if count ==0 & treatment
==1

        **effect across salary distribution
        if `control_time'==5 matrix results = J(81,5,.) // matrix
to capture results

        local n = `control_time'
        gen post = count >=0
        replace count = count+6
        local v =0

        qui{
                forvalue t= 0(14932)1045240{
                        local top = 42
                        local v =1+`v'
                        if `n' ==5 matrix results[`v',1]= `t'
                        gen D = personindk <=`t'
                        reg D treat i.treatment i.post i.edu
i.alder i.female i.time, cl(pnr)
                        matrix V = e(V)
                        matrix b= e(b)
                        matrix results[`v',2]    = b[1,1]
                        margins, at(treat= (0 1) treatment=1
post=1)
                        matrix results[`v',5]    = V[1,1]^.5
                        matrix M = r(b)
                        matrix results[`v',4]    = M[1,2] //
                        matrix results[`v',3]    = M[1,1] //
                        drop D
                }
        }
}

svmat results

gen l5 = results2-1.96*results5
gen u5 = results2+1.96*results5


replace results1 = results1/7466

gen dif6 = results4-results3
gen udif6 = u5
gen ldif6 = l5
```

```
gr two rline udif6 ldif6 results1, ///
        yaxis(2) color(gray) lp(dash) ylab(0(.01).04, axis(2))
ysc(range(-.006 .04) axis(2))     || ///
        line dif6 results1, yaxis(2) yline(0, axis(2)) ///
        lcolor(black)  ylab(0(.01).04)  ysc(range(-.006 .04)
axis(2)) || ///
        line results4 results1, yaxis(1) lcolor(red) || ///
        line results3 results1, yaxis(1) lcolor(blue) ///
         ysc(range(0 1)) ylab(0(.1)1, nogrid) xlab(0(10)140)
xsc(range(0 140)) ///
        ysize(10) xsize(12) graphr(c(white)) ///
        xti("Total Income in 1K Euro") scale(1) ///
        yti("Effect of Concussion on Pr(Total Income < X)",
axis(2)) ///
        yti("{&Phi}(Total Income)",  axis(1)) ///
        legend( ///
        label(2 "Effect of concussion (left axis)") ///
        label(3 "Concussion income distribution (right axis)") ///
        label(4 "Counterfactual income distribution (right
axis)") ///
        c(1) order(2 3 4) pos(6) size(small) ///
                         symx(4) region(lc(white)))



graph export [home]\highdef\l5_income.png,  replace width(3900)

cap graph drop g1 g2

gr two ///
        line results4 results1, yaxis(1) lcolor(red) || ///
        line results3 results1, yaxis(1) lcolor(blue) ///
         ysc(range(.2 1)) ylab(0(.1)1) xlab(0(20)140,
labs(small)) ///
         xsc(range(0 140)) ///
        ysize(10) xsize(10) graphr(c(white)) ///
        xti("Total Income in 1K Euro") scale(1) ///
        yti("Cumulative Distribution of Total Income",
axis(1)) ///
        legend( ///
        label(1 "Observed Post-Concussion Total Income
Distribution") ///
        label(2 "Counterfactual No-Concussion Total Income
Distribution") ///
        c(1) order(1 2 4) pos(6) size(small) ///
                         symx(4) region(lc(white))) , name(g1)

gr two rline udif6 ldif6 results1, ///
        color(gray) lp(dash) ysc(range(-.002 .035)) || ///
        line dif6 results1,  yline(0) ///
        lcolor(black)  ylab(0(.01).0325)  ysc(range(-.002 .
035)) ///
        xlab(0(20)140, labs(small)) xsc(range(0 140))      ///
        ysize(10) xsize(10) graphr(c(white)) ///
```

```
        xti("Total Income in 1K Euro (X)") scale(1) ///
        yti("Effect of Concussion on Pr(Total Income < X)") ///
                legend( label(1 "95% CI") ///
        label(2 "Effect of concussion") ///
        c(1) order(2 1) pos(6) size(small) ///
                        symx(4) region(lc(white))) , name(g2)

graph combine g1 g2      ,graphr(c(white))

graph export "[home]\highdef\figure s2.tif",  replace width(1000)



********************************************************************
************
**
**      Effects across the salary distribution
**
**
**
********************************************************************
************


forvalue control_time=5/5{
        local end = 2012 // last incident year in data

        **build dataset for joint estimate across years
        forvalue count=2003/`end'{
                if `count'==2003{
                        use $data/
sample_control_`count'_`control_time'.dta, clear
                        gen time = `count' //incident year
indicator
                }
                else append using $data/
sample_control_`count'_`control_time'.dta
                replace time = `count' if time ==.

                **exclude individuals in years where they do not
appear in data,
                **due to either death or migration, as well as
periods from when
                **the control group sufer their concussion
                drop if merge ==1 | count > `control_time'-1
        }

        gen female = koen==2
        qui{
                gen edu =0
                replace edu = 1 if inrange(hffsp,20000000,39000000)
| ///
                                                                (hffsp
>40000000 & hffsp!=.)
```

```
        }

        //build ident, so we can multivariate cluster for
individuals
        //who occur both as control and exposure during the period
(id)

        bysort pnr time: gen helpx = _n ==1
        gen id= sum(helpx)
        drop helpx


        **generate concussion variable
        gen treat = inrange(count,0,`control_time'-1) & treatment
==1
        replace treat = time_from_incident if count ==0 & treatment
==1

        **effect across salary distribution
        if `control_time'==5 matrix results = J(81,5,.) // matrix
to capture results

        local n = `control_time'
        gen post = count >=0
        replace count = count+6
        local v =0

        **Estimate Pr(salary < X) across income distribution
        qui{
                forvalue t= 0(14932)895950{
                        local top = 42
                        local v =1+`v'
                        if `n' ==5 matrix results[`v',1]= `t'
                        gen D = loenmv <=`t'
                        reg D treat i.treatment i.post i.edu
i.alder i.female i.time, cl(pnr)
                        matrix V = e(V)
                        matrix b= e(b)
                        matrix results[`v',2]    = b[1,1]
                        margins, at(treat= (0 1) treatment=1
post=1)
                        matrix results[`v',5]    = V[1,1]^.5
                        matrix M = r(b)
                        matrix results[`v',4]    = M[1,2] //
                        matrix results[`v',3]    = M[1,1] //
                        drop D
                }
        }
}

svmat results

*Generate 95% confidence intervals
gen l5 = results2-1.96*results5
```

```
gen u5 = results2+1.96*results5

*Correct to €
replace results1 = results1/7466

*Obtain difference between observed and counterfactual wage
distribution
gen dif6 = results4-results3
gen udif6 = u5
gen ldif6 = l5

gr two rline udif6 ldif6 results1, ///
        yaxis(2) color(gray) lp(dash) ysc(range(-.006 .04) axis(2))
|| ///
        line dif6 results1, yaxis(2) yline(0, axis(2)) ///
        lcolor(black)  ylab(0(.01).0325)  ysc(range(-.006 .04)
axis(2)) || ///
        line results4 results1, yaxis(1) lcolor(red) || ///
        line results3 results1, yaxis(1) lcolor(blue) ///
         ysc(range(0 1)) ylab(0(.1)1, nogrid) xlab(0(10)120)
xsc(range(0 120)) ///
        ysize(10) xsize(12) graphr(c(white)) ///
        xti("Salary in 1K Euro") scale(1) ///
        yti("Effect of Concussion on Pr(Salary < X)", axis(2)) ///
        yti("{&Phi}(Salary)",  axis(1)) ///
        legend( ///
        label(2 "Effect of concussion (left axis)") ///
        label(3 "Concussion salary distribution (right axis)") ///
        label(4 "Counterfactual salary distribution (right
axis)") ///
        c(1) order(2 3 4) pos(6) size(small) ///
                         symx(4) region(lc(white)))

cap graph drop g1 g2

gr two ///
        line results4 results1, yaxis(1) lcolor(red) || ///
        line results3 results1, yaxis(1) lcolor(blue) ///
         ysc(range(.2 1)) ylab(0.2(.1)1) xlab(0(10)120,
labs(small)) ///
         xsc(range(0 120)) ///
        ysize(10) xsize(10) graphr(c(white)) ///
        xti("Salary in 1K Euro") scale(1) ///
        yti("Cumulative Distribution of Salary",  axis(1)) ///
        legend( ///
        label(1 "Observed Post-Concussion Salary Distribution") ///
        label(2 "Counterfactual No-Concussion Salary
Distribution") ///
        c(1) order(1 2 4) pos(6) size(small) ///
                         symx(4) region(lc(white))) , name(g1)

gr two rline udif6 ldif6 results1, ///
        color(gray) lp(dash) ysc(range(-.002 .035)) || ///
        line dif6 results1,  yline(0) ///
```

```
        lcolor(black)  ylab(0(.01).0325)  ysc(range(-.002 .
035)) ///
        xlab(0(10)120, labs(small)) xsc(range(0 120))      ///
        ysize(10) xsize(10) graphr(c(white)) ///
        xti("Salary in 1K Euro (X)") scale(1) ///
        yti("Effect of Concussion on Pr(Salary < X)") ///
                legend( label(1 "95% CI") ///
        label(2 "Effect of concussion") ///
        c(1) order(2 1) pos(6) size(small) ///
                         symx(4) region(lc(white))) , name(g2)

graph combine g1 g2      ,graphr(c(white))

graph export "[home]\highdef\figure 3.tif",  replace width(1000)




**ESTIMATE SICK LEAVE

forvalue control_time=1/5{
        local end = 2012 // last incident year in data
        if `control_time' ==1    eststo clear

        **build dataset for joint estimate across years
        forvalue count=2003/`end'{
                if `count'==2003{
                        use $data/
sample_control_`count'_`control_time'.dta, clear
                        gen time = `count' //incident year
indicator
                }
                else append using $data/
sample_control_`count'_`control_time'.dta
                replace time = `count' if time ==.

                **exclude individuals in years where they do not
appear in data,
                **due to either death or migration, as well as
periods from when
                **the control group sufer their concussion
                drop if merge ==1 | count > `control_time'-1
        }

        merge m:1 pnr t using $data/temp.dta, keep(1 3) nogen
        replace share = 0 if share==.

        gen female = koen==2

        //build ident, so we can multivariate cluster for
individuals
        //who occur both as control and exposure during the period
(id)
```

```
        bysort pnr time: gen helpx = _n ==1
        gen id= sum(helpx)
        drop helpx


        **Generate educational groups
        qui{
                gen edu =0
                replace edu = 1 if inrange(hffsp,20000000,39000000)
| ///
                                                                (hffsp
>40000000 & hffsp!=.)
        }


        **Calculate number of observations for exposure and control
        count if count==0 & treatment ==1
        local Ntreated = r(N)
        count if count==0 & treatment ==0
        local Ncontrol = r(N)

        **generate concussion variable
        gen treat = inrange(count,0,`control_time'-1) & treatment
==1
        replace treat = time_from_incident if count ==0 & treatment
==1

        **Generate pre-concussion income difference        for
        **use in calculating marginal effects
        sum loenmv if count <0 & treatment ==0
        local control =r(mean)
        sum loenmv if count <0 & treatment ==1
        local treat =r(mean)
        sum loenmv if count>=0 & treatment ==0
        local control_post =r(mean)

        forvalue t=-4/4{
                local n = `t'*-1
                if `t' < -1 gen T_`n' = treatment ==1 & count ==`t'
                if `t' > -1 gen T`t' = treatment ==1 & count ==`t'

        }

        **estimate DiD model on salary
        reghdfe share T*, abs(alder female count time treatment
edu) cl(pnr  id)
        eststo est1_`control_time'
        if `control_time'==1 matrix results = J(5,5,.) // matrix to
capture results
        if `control_time'==1 matrix results_p = J(5,5,.) // matrix
to capture results
```

```
        matrix b = e(b)
        matrix V = e(V)
        local n = `control_time'

        matrix results[`n',1]    = b[1,1] / 7466  // capture beta
results as 1K Euro
        matrix results[`n',2]    = (V[1,1]^.5)/7466       //
capture standard error as 1K Euro
        matrix results[`n',3]    =       b[1,1]/(`control_post'-
(`control'-`treat'))
        matrix results[`n',4]    =       `n'

        gen no_share = share >0 //dummy for no salary


        **Generate pre-concussion probability difference   for
        **use in calculating marginal effects
        sum no_share if count <0 & treatment ==0
        local control =r(mean)
        sum no_share if count <0 & treatment ==1
        local treat =r(mean)
        sum no_share if count>=0 & treatment ==0
        local control_post =r(mean)

        **estimate DiD LP-model on P(salary=0)
        xi: reghdfe no_share T*, abs(alder female count time
treatment edu) cl(pnr  id)
        eststo est2_`control_time'
        matrix b =e(b)   //regression coefficient
        matrix V = e(V)  // standard error^2

        matrix results_p[`n',1]  =       b[1,1]
        matrix results_p[`n',2]  =       V[1,1]^.5
        matrix results_p[`n',3]  =       b[1,1]/(`control_post'-
(`control'-`treat'))
        matrix results_p[`n',4]  =       `n'

}

esttab est1_* using [home]/tables/dynamic_share1.rtf, ///
        replace se(3) b(3) compress nogap star(+ .1 * .05 ** .01
*** .001) ///
        keep(T*)

esttab est2_* using [home]/tables/dynamic_share2.rtf, ///
        replace se(3) b(3) compress nogap star(+ .1 * .05 ** .01
*** .001) ///
        keep(T*)

**ESTIMATE SICK LEAVE

forvalue control_time=1/5{
        local end = 2012 // last incident year in data
        if `control_time' ==1    eststo clear
```

```
        **build dataset for joint estimate across years
        forvalue count=2003/`end'{
                if `count'==2003{
                        use $data/
sample_control_`count'_`control_time'.dta, clear
                        gen time = `count' //incident year
indicator
                }
                else append using $data/
sample_control_`count'_`control_time'.dta
                replace time = `count' if time ==.

                **exclude individuals in years where they do not
appear in data,
                **due to either death or migration, as well as
periods from when
                **the control group sufer their concussion
                drop if merge ==1 | count > `control_time'-1
        }

        merge m:1 pnr t using $data/temp2.dta, keep(1 3) nogen
        replace syg_barsel_13= 0 if syg_barsel_13==.

        gen female = koen==2

        //build ident, so we can multivariate cluster for
individuals
        //who occur both as control and exposure during the period
(id)

        bysort pnr time: gen helpx = _n ==1
        gen id= sum(helpx)
        drop helpx


        **Generate educational groups
        qui{
                gen edu =0
                replace edu = 1 if inrange(hffsp,20000000,39000000)
| ///
                                                                (hffsp
>40000000 & hffsp!=.)
        }


        **Calculate number of observations for exposure and control
        count if count==0 & treatment ==1
        local Ntreated = r(N)
        count if count==0 & treatment ==0
        local Ncontrol = r(N)

        **generate concussion variable
        gen treat = inrange(count,0,`control_time'-1) & treatment
```

```
==1
        replace treat = time_from_incident if count ==0 & treatment
==1

        **Generate pre-concussion income difference        for
        **use in calculating marginal effects
        sum loenmv if count <0 & treatment ==0
        local control =r(mean)
        sum loenmv if count <0 & treatment ==1
        local treat =r(mean)
        sum loenmv if count>=0 & treatment ==0
        local control_post =r(mean)

        forvalue t=-4/4{
                local n = `t'*-1
                if `t' < -1 gen T_`n' = treatment ==1 & count ==`t'
                if `t' > -1 gen T`t' = treatment ==1 & count ==`t'

        }

        **estimate DiD model on salary
        reghdfe syg_barsel_13 T*, abs(alder female count time
treatment edu) cl(pnr  id)
        eststo est1_`control_time'
        if `control_time'==1 matrix results = J(5,5,.) // matrix to
capture results
        if `control_time'==1 matrix results_p = J(5,5,.) // matrix
to capture results


        matrix b = e(b)
        matrix V = e(V)
        local n = `control_time'

        matrix results[`n',1]    = b[1,1] / 7466  // capture beta
results as 1K Euro
        matrix results[`n',2]    = (V[1,1]^.5)/7466       //
capture standard error as 1K Euro
        matrix results[`n',3]    =       b[1,1]/(`control_post'-
(`control'-`treat'))
        matrix results[`n',4]    =       `n'




        **Generate pre-concussion probability difference   for
        **use in calculating marginal effects

        **estimate DiD LP-model on P(salary=0)
        xi: reghdfe kont_dag T*, abs(alder female count time
treatment edu) cl(pnr  id)
        eststo est2_`control_time'
        matrix b =e(b)   //regression coefficient
        matrix V = e(V)  // standard error^2
```

```
		matrix results_p[`n',1]	=	b[1,1]
		matrix results_p[`n',2]	=	V[1,1]^.5
		*matrix results_p[`n',3]	=	b[1,1]/(`control_post'-
	(`control'-`treat'))
		matrix results_p[`n',4]	=	`n'

	}

	esttab est1_* using [home]/tables/dynamic_sickpay1.rtf, ///
		replace se(3) b(3) compress nogap star(+ .1 * .05 ** .01
	*** .001) ///
		keep(T*)

	esttab est2_* using [home]/tables/dynamic_welfare2.rtf, ///
		replace se(3) b(3) compress nogap star(+ .1 * .05 ** .01
	*** .001) ///
		keep(T*)
```

Supplemental results

For peer review only

For peer review only - http://bmjopen.bmj.com/site/about/guidelines.xhtml

Table S1. **Test of parallel trends assumption pre-exposure incident against each control group separately using eq. S3 in supplementary methods. Separate exposure dummies for all time periods (except the year prior to exposure, which serves as reference period).**

**Time to exposure group's concussion (exposure)**	**Δ=1 Est (S.E.) p-value**	**Δ=2 Est (S.E.) p-value**	**Δ=3 Est (S.E.) p-value**	**Δ=4 Est (S.E.) p-value**	**Δ=5 Est (S.E.) p-value**
**Exposure-4y**	-0.368 (0.226) p=.104	0.046 (0.363) p=.900	0.120 (0.362) p=.741	0.159 (0.313) p=.612	0.042 (0.329) p=.899
**Exposure-3y**	-0.094 (0.317) p=.768	0.227 (0.510) p=.656	0.167 (0.354) p=.637	0.537 (0.372) p=.148	0.113 (0.393) p=.774
**Exposure-2y**	-0.548 (0.312) p=.079	-0.082 (0.347) p=.812	-0.163 (0.236) p=.491	-0.124 (0.247) p=.617	0.082 (0.250) p=.744
**Exposure-1y**	Ref.	Ref.	Ref.	Ref.	Ref.
**N*T**	284115	273725	266120	260647	256337

Note: The table shows test for differences in pre-exposure trends between exposure and control group model using interactions between pre-exposure time dummies and the exposure indicator. There is no indication of substantial or significant pre-exposure differences in salary trajectories between exposure group and any of the control groups.

For peer review only - http://bmjopen.bmj.com/site/about/guidelines.xhtml

Table S2. **Effect of concussion on different labor market outcome parameters using separate exposure dummies for all time periods (except the year prior to exposure, which serves as reference period): In this exploratory analysis, the exposure group is compared to the control group Δ=5, which suffers a concussion five years after the exposure group. Outcomes include annual salaried income (annual salary), total annual income (total income), annual sick leave benefits received (sick leave benefits) as well as a binary indicator of employment (probability of employment). Monetary outcomes were measured at 2015-level in € 1,000.**

Time to exposure group's concussion (exposure)	Annual Salary Est. (S.E.) p-value	Total income Est. (S.E.) p-value	Sick leave benefits Est. (S.E.) p-value	Probability of employment Est. (S.E.) p-value
**Exposure-4y**	0.012	0.164	0.035	0.001
	(0.212)	(0.173)	(0.036)	(0.004)
	p=.954	p=.343	p=.320	p=.803
**Exposure-3y**	0.059	0.305	0.022	-0.001
	(0.252)	(0.233)	(0.034)	(0.003)
	p=.814	p=.190	p=.529	p=.739
**Exposure-2y**	0.043	0.122	0.002	0.001
	(0.160)	(0.147)	(0.029)	(0.003)
	p=.788	p=.405	p=.946	p=.739
**Exposure-1y**				
**Exposure**	-0.611	-0.338	0.166	-0.003
	(0.168)	(0.140)	(0.030)	(0.003)
	p<.001	0.016	p<.001	p=.317
**Exposure+1y**	-1.389	-0.608	0.288	-0.020
	(0.209)	(0.162)	(0.039)	(0.003)
	p<.001	p<.001	p<.001	p<.001
**Exposure+2y**	-1.568	-0.847	0.132	-0.023
	(0.261)	(0.231)	(0.039)	(0.004)
	p<.001	p<.001	p=.001	p<.001
**Exposure+3y**	-1.393	-0.497	0.031	-0.022
	(0.246)	(0.219)	(0.040)	(0.004)
	p<.001	p=.023	p=.432	p<.001
**Exposure+4y**	-1.319	-0.499	-0.076	-0.018
	(0.253)	(0.218)	(0.042)	(0.004)
	p<.001	p=.022	p=.075	p<.001
**N*T**	577762	577758	577872	577872

Note: Annual salary include all income from salary and employee fringe benefits, employee stock options, employer paid sick leave, net gains (including interests and capital gains) from own companies. Total income includes all income absent wealth. Sick leave includes only public health benefits (sick leave and paternity leave). Employment is a binary indicator measured last week of November for each year. Results obtained from estimations following Eq. (1). Models include controls for high school diploma, gender, age, and observation year. Results obtained using reghdfe in Stata. Total number of observations (N*T) differ slightly between outcomes because all income information is not available for all observation all years.

Source: Own calculations on data from Statistics Denmark.

For peer review only - http://bmjopen.bmj.com/site/about/guidelines.xhtml

Table S3. **Demographic factors for exposure group and control groups (Δ=1, 2, 3, 4, 5) averaged over the 5 years leading up to the concussion event in each of the groups. Factors include patient age (in years), share of sample female (1=100% female), and share of individuals with at least a high school degree (1=100%).**

		Exposure	Control, Δ=1	Control, Δ=2	Control, Δ=3	Control, Δ=4	Control, Δ=5
**Pr(Female=1)**	Mean	.430	.438	.447	.458	.464	.473
	S.D.	(.495)	(.496)	(.497)	(.498)	(.499)	(.499)
	p-value		.030	<.001	<.001	<.001	<.001
**Age**	Mean	36.899	37.354	37.754	38.065	38.343	38.592
	S.D.	(11.856)	(11.857)	(11.718)	(11.630)	(11.584)	(11.491)
	p-value		<.001	<.001	<.001	<.001	<.001
**Pr(High school=1)**	Mean	.624	.632	.640	.646	.653	.660
	S.D.	(.484)	(.482)	(.480)	(.478)	(.476)	(.474)
	p-value		.026	<.001	<.001	<.001	<.001
**Total individuals**		37848	34551	31851	29922	28580	27484

Note: S.D.: Standard deviation. P-values calculated using two-sided t-tests. All test performed between exposure group and each control group separately.

For peer review only

Figure S1. **Unnormalized Average Salary for Treatment and Control Groups Measured in 1K € at 2015-levels**

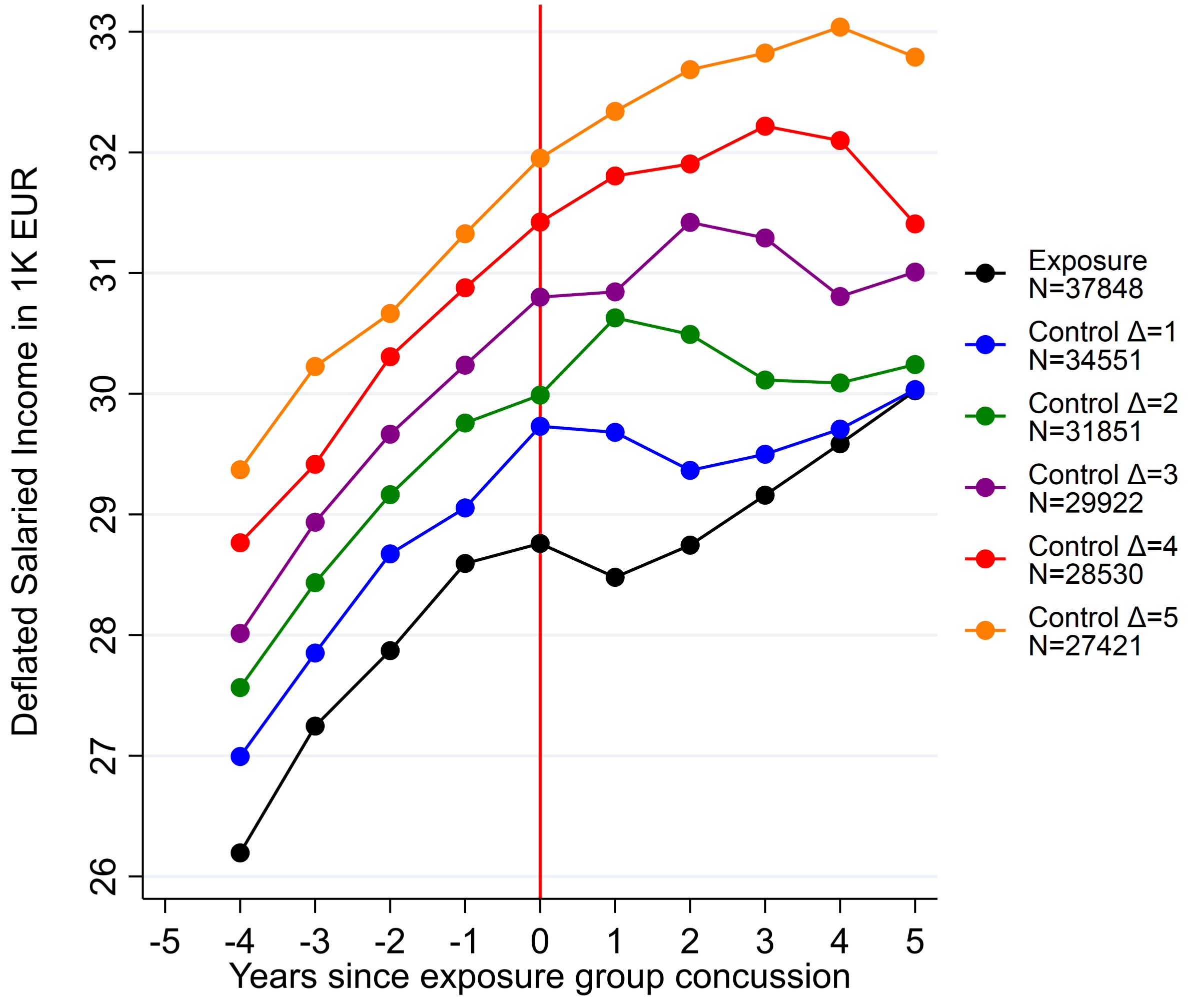

Note: Salary of the exposure group compared to salary of the 5 control groups, who experienced their concussions Δ={1, 2, 3, 4, and 5} years later than the exposure group. Salary progression is shown for the 5 years before and the 5 years after the exposure group suffered a concussion event. Table S1 demonstrates that the trends for salary progression pre-exposure incident are parallel between exposure group and each control group.

Figure S2. (Left Panel)The Cumulative Distribution for Total Income Post-Treatment among the Treatment Group and Their Counterfactual, and (Right Panel) the Difference between the Two CDFs Expressed as the Effect of Concussion on the Probability of Total Income Below that Income-Level Expressed on the X-Axis following Exposure Event.

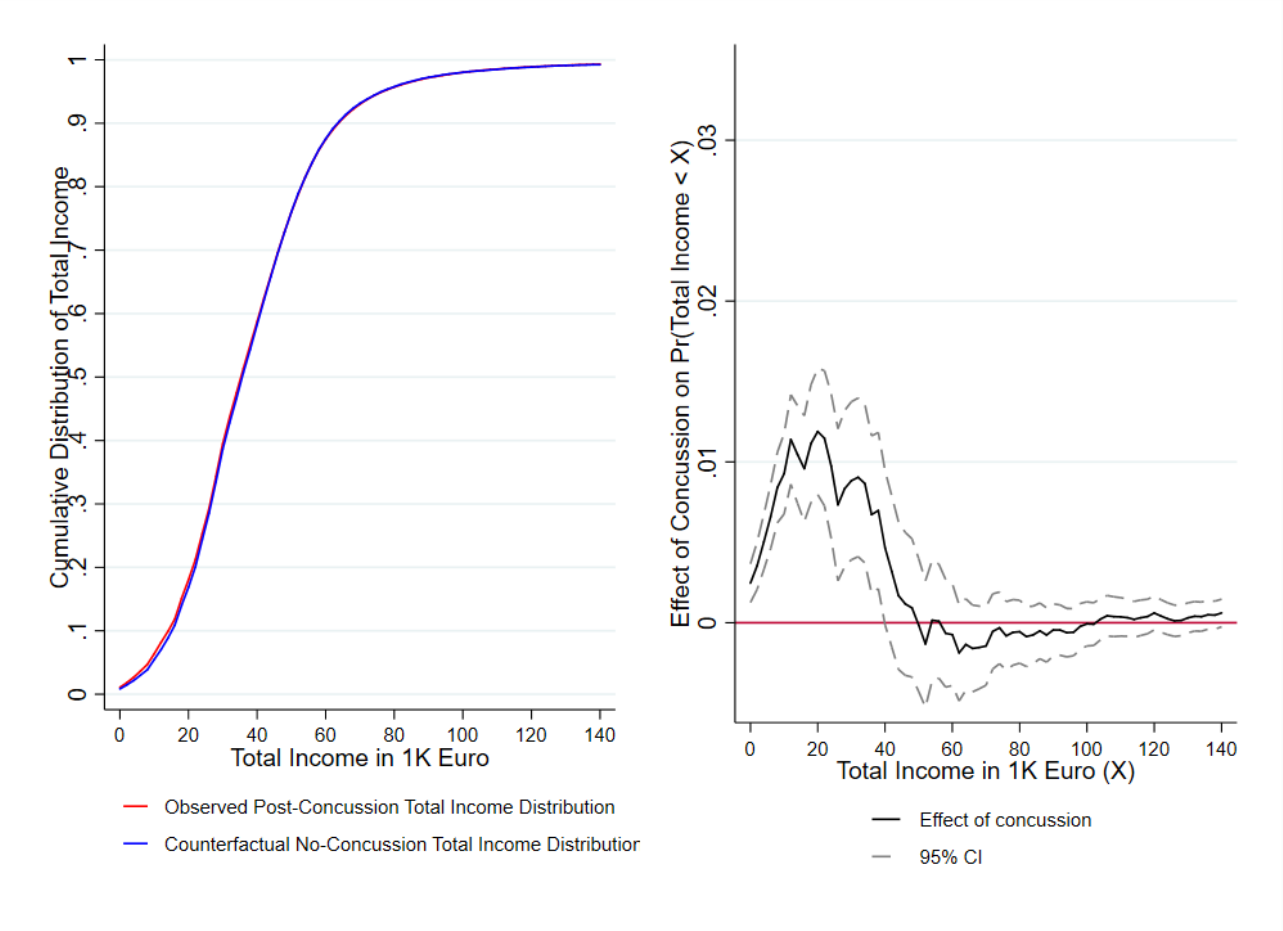

Note: The figure shows the observed cumulative salary distribution following a concussion (red) and the expected counterfactual salary distribution absent the concussion (blue). The black line shows the difference between the observed and the counterfactual distribution, and the grey dash lines show the 95 % confidence interval. The bell-shape of the difference between the two distributions as the total income increase from 0 to 40,000 € indicates that the main part of the effect of concussions on total incomes is driven by low-income people shifting total income downwards following concussion, but not going to total income equal to zero.

Figure S3. Percentage Effect of Concussion on Relative Salary Across Age Groups.

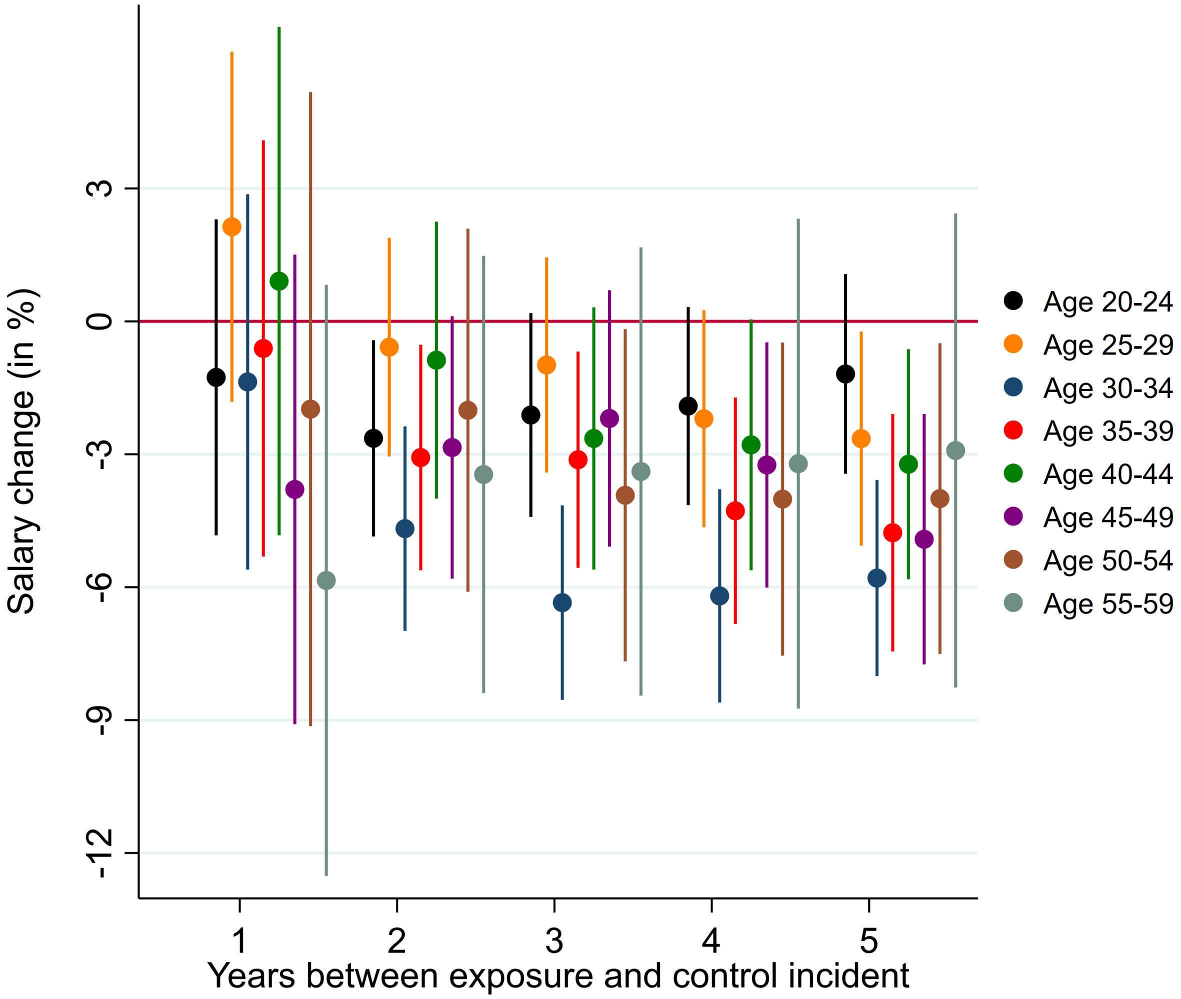

Note: Relative salary drop of the exposure group compared to the control groups, who suffer a concussion 1, 2, 3, 4, and 5 years after the exposure group. Relative salary changes are shown across different age groups. Graph shows parameter estimates and 95% CI.

For peer review only - http://bmjopen.bmj.com/site/about/guidelines.xhtml

Figure S4. **Percentage Effect of Concussion on Relative Salary Across High School Completion.**

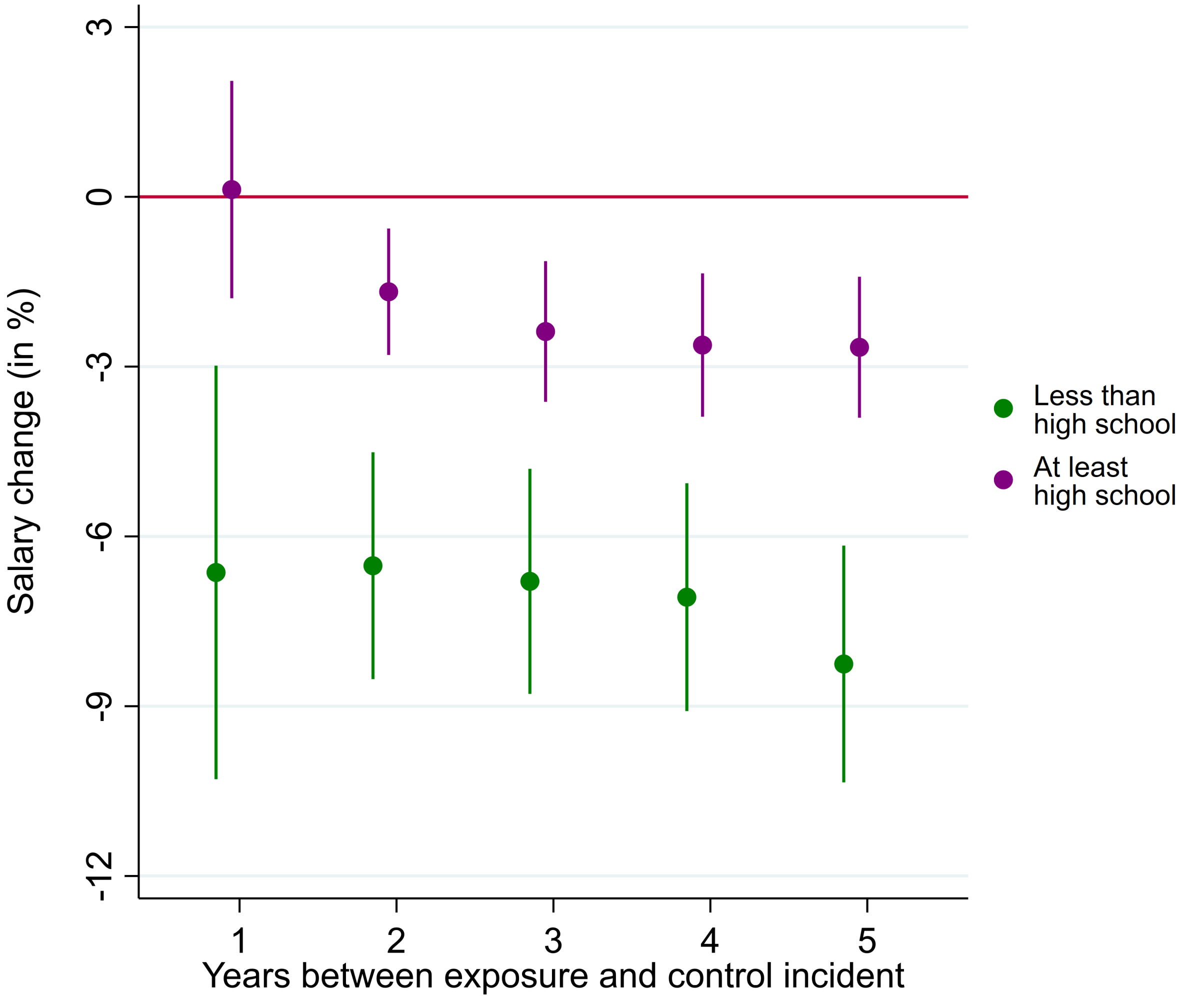

Note: Relative salary drop of the exposure group compared to the control groups, who suffer a concussion 1, 2, 3, 4, and 5 years after the exposure group. Relative salary changes are shown across whether individuals had obtained at least a high school diploma (ISCED > 2). Graph shows parameter estimates and 95% CI.

For peer review only - http://bmjopen.bmj.com/site/about/guidelines.xhtml

Figure S5. **Percentage Effect of Concussion on Relative Salary Across Gender.**

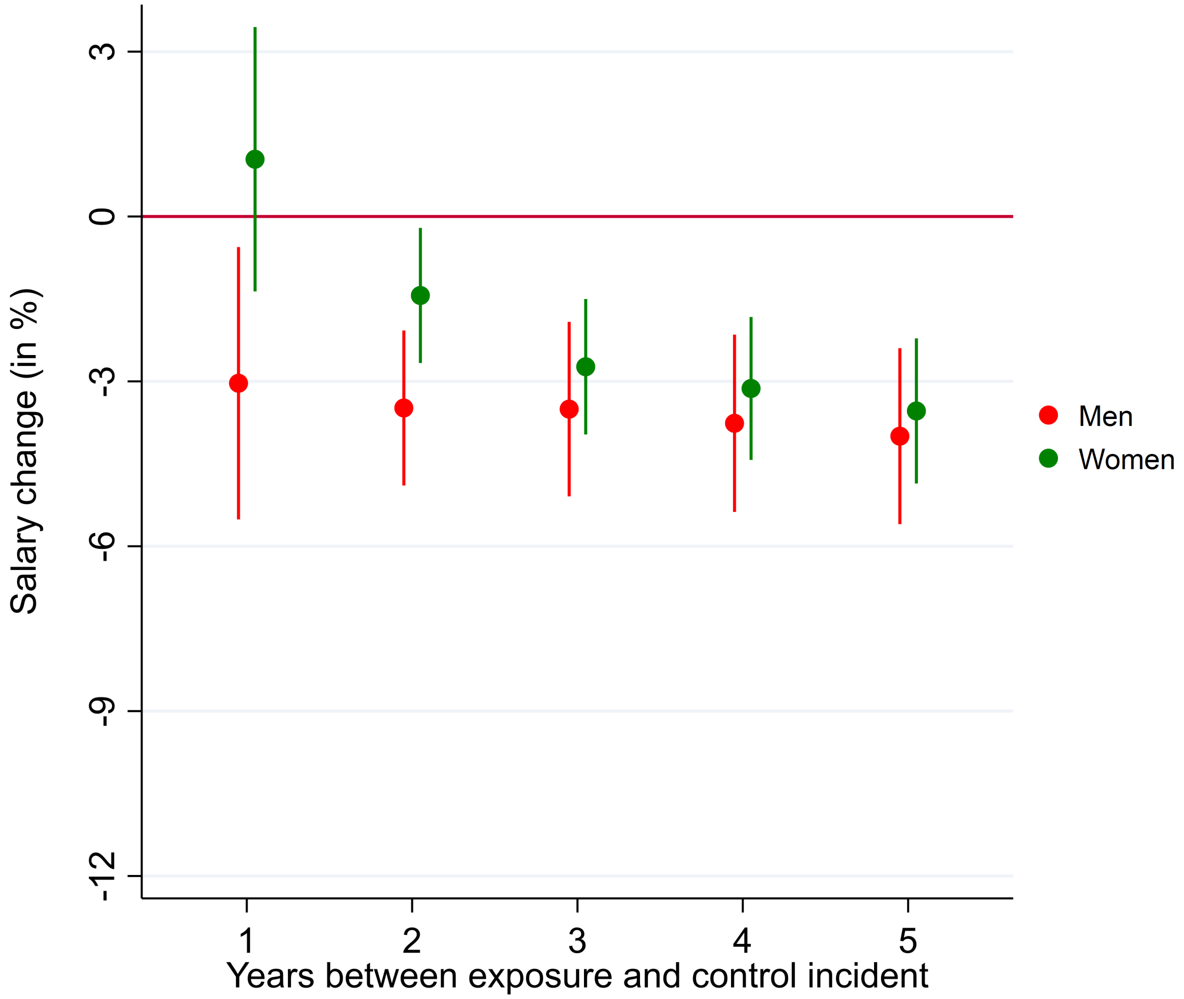

Note: Relative salary drop of the exposure group compared to the control groups, who suffer a concussion 1, 2, 3, 4, and 5 years after the exposure group. Relative salary changes are shown across gender. Graph shows parameter estimates and 95% CI.

only

For peer review only - http://bmjopen.bmj.com/site/about/guidelines.xhtml

Figure S6. **Effect of Concussion on Absolute Salary in 1K Euro Across Age groups.**

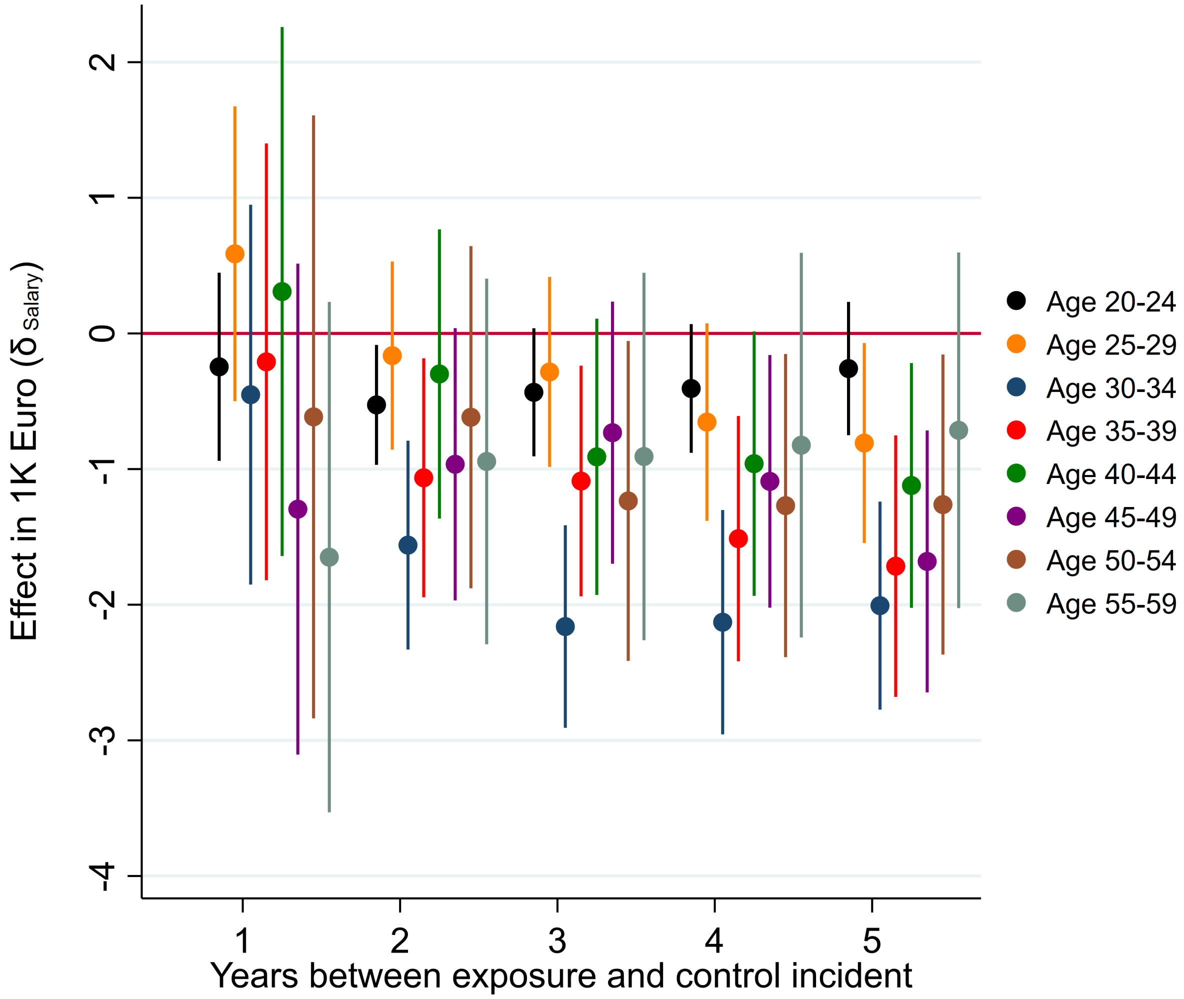

Note: Absolute salary drop of the exposure group compared to the control groups, who suffer a concussion 1, 2, 3, 4, and 5 years after the exposure group. Absolute salary changes are shown across different age groups. Graph shows parameter estimates and 95% CI.

y only

Figure S7. **Effect of Concussion on Absolute Salary in 1K Euro Across Education.**

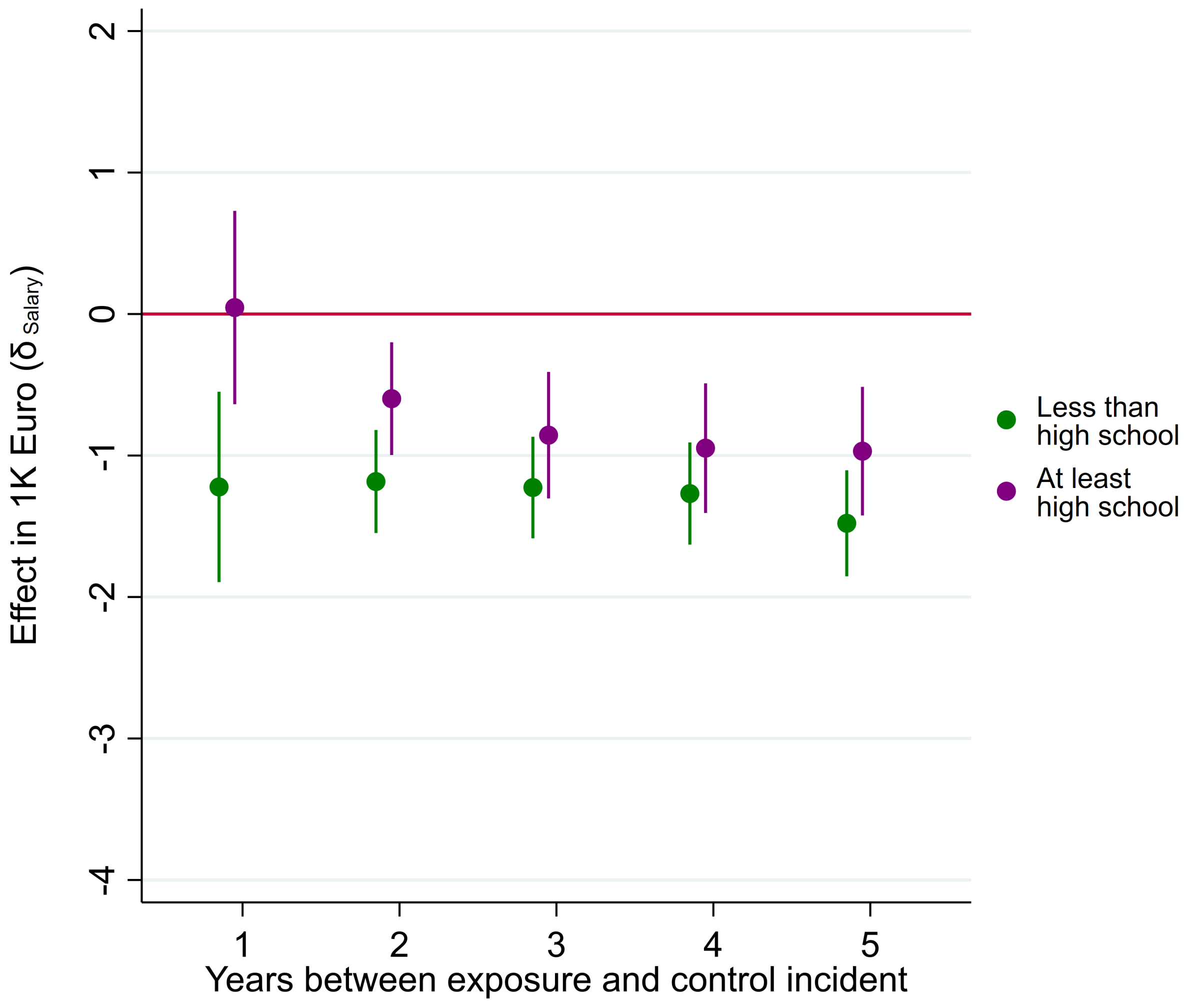

Note: Absolute salary drop of the exposure group compared to the control groups, who suffer a concussion 1, 2, 3, 4, and 5 years after the exposure group. Absolute changes in salary are shown across whether individuals had obtained at least a high school diploma (ISCED > 2). Graph shows parameter estimates and 95% CI.

Figure S8. **Effect of Concussion on Absolute Salary in 1K Euro Across Gender.**

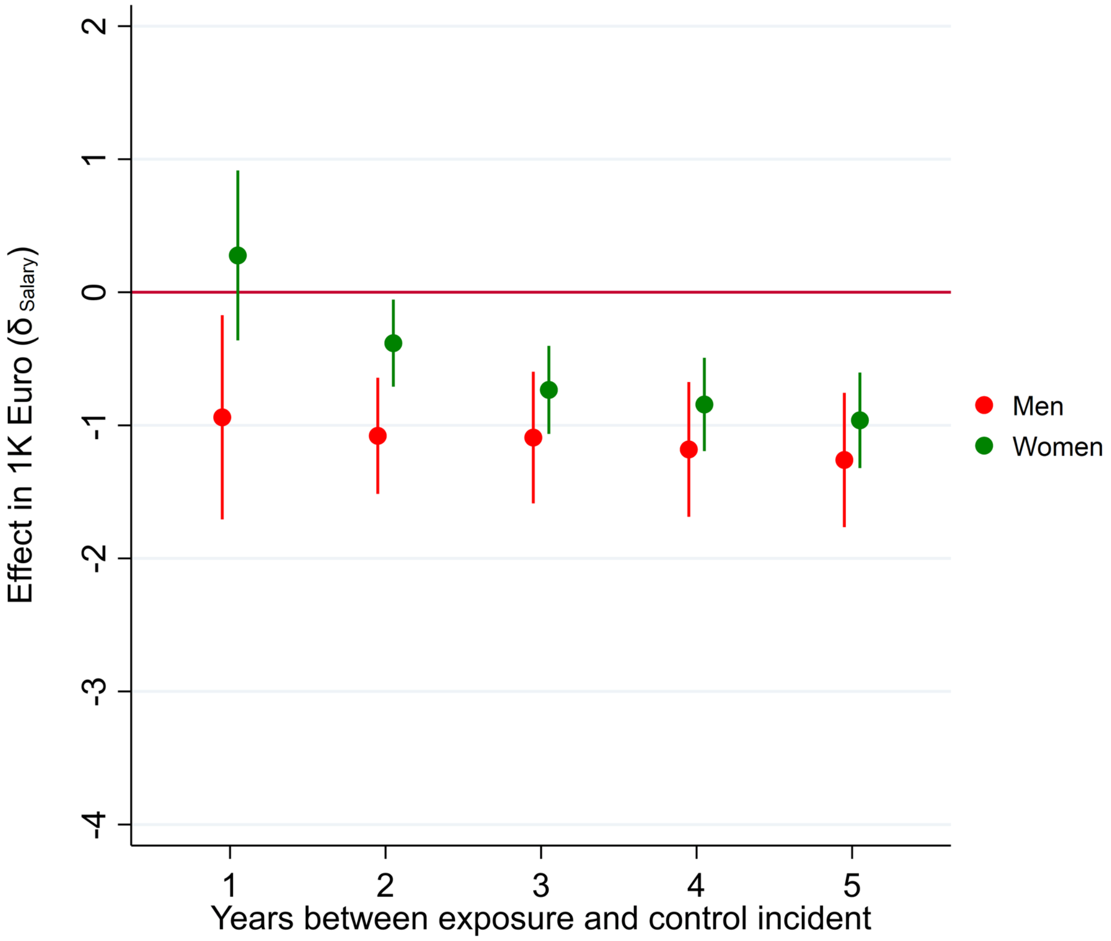

Note: Absolute salary drop of the exposure group compared to the control groups, who suffer a concussion 1, 2, 3, 4, and 5 years after the exposure group. Absolute changes in salary are shown across gender. Graph shows parameter estimates and 95% CI.

For peer review only - http://bmjopen.bmj.com/site/about/guidelines.xhtml

The RECORD statement – checklist of items, extended from the STROBE statement, that should be reported in observational studies using routinely collected health data.

	Item No.	STROBE items	Location in manuscript where items are reported	RECORD items	Location in manuscript where items are reported
**Title and abstract**					
	1	(a) Indicate the study's design with a commonly used term in the title or the abstract (b) Provide in the abstract an informative and balanced summary of what was done and what was found	title<br>abstract	RECORD 1.1: The type of data used should be specified in the title or abstract. When possible, the name of the databases used should be included.<br><br>RECORD 1.2: If applicable, the geographic region and timeframe within which the study took place should be reported in the title or abstract.<br><br>RECORD 1.3: If linkage between databases was conducted for the study, this should be clearly stated in the title or abstract.	title<br>abstract<br><br>title<br>abstract<br><br>abstract
**Introduction**					
Background rationale	2	Explain the scientific background and rationale for the investigation being reported	abstract<br>introduction		
Objectives	3	State specific objectives, including any prespecified hypotheses	introduction		
**Methods**					
Study Design	4	Present key elements of study design early in the paper	introduction		
Setting	5	Describe the setting, locations, and relevant dates, including periods of recruitment, exposure, follow-up, and data collection	materials and methods		

For peer review only

Participants	6	*(a) Cohort study* - Give the eligibility criteria, and the sources and methods of selection of participants. Describe methods of follow-up<br>*Case-control study* - Give the eligibility criteria, and the sources and methods of case ascertainment and control selection. Give the rationale for the choice of cases and controls<br>*Cross-sectional study* - Give the eligibility criteria, and the sources and methods of selection of participants<br><br>*(b) Cohort study* - For matched studies, give matching criteria and number of exposed and unexposed<br>*Case-control study* - For matched studies, give matching criteria and the number of controls per case	materials and methods	RECORD 6.1: The methods of study population selection (such as codes or algorithms used to identify subjects) should be listed in detail. If this is not possible, an explanation should be provided.<br><br>RECORD 6.2: Any validation studies of the codes or algorithms used to select the population should be referenced. If validation was conducted for this study and not published elsewhere, detailed methods and results should be provided.<br><br>RECORD 6.3: If the study involved linkage of databases, consider use of a flow diagram or other graphical display to demonstrate the data linkage process, including the number of individuals with linked data at each stage.	materials and methods<br><br>materials and methods<br><br>not included
Variables	7	Clearly define all outcomes, exposures, predictors, potential confounders, and effect modifiers. Give diagnostic criteria, if applicable.	materials and methods<br>main text	RECORD 7.1: A complete list of codes and algorithms used to classify exposures, outcomes, confounders, and effect modifiers should be provided. If these cannot be reported, an explanation should be provided.	materials and methods
Data sources/ measurement	8	For each variable of interest, give sources of data and details of methods of assessment (measurement).<br>Describe comparability of assessment methods if there is more than one group	materials and methods		

For peer review only

Bias	9	Describe any efforts to address potential sources of bias	materials and methods and results		
Study size	10	Explain how the study size was arrived at	materials and methods		
Quantitative variables	11	Explain how quantitative variables were handled in the analyses. If applicable, describe which groupings were chosen, and why	materials and methods		
Statistical methods	12	(a) Describe all statistical methods, including those used to control for confounding<br>(b) Describe any methods used to examine subgroups and interactions<br>(c) Explain how missing data were addressed<br>(d) *Cohort study* - If applicable, explain how loss to follow-up was addressed<br>*Case-control study* - If applicable, explain how matching of cases and controls was addressed<br>*Cross-sectional study* - If applicable, describe analytical methods taking account of sampling strategy<br>(e) Describe any sensitivity analyses	a) materials and methods<br>b) materials and methods<br>c) materials and methods<br>d-e) NA		
Data access and cleaning methods		..		RECORD 12.1: Authors should describe the extent to which the investigators had access to the database population used to create the study population.	materials and methods<br><br>materials and methods

For peer review only

				RECORD 12.2: Authors should provide information on the data cleaning methods used in the study.	
Linkage		..		RECORD 12.3: State whether the study included person-level, institutional-level, or other data linkage across two or more databases. The methods of linkage and methods of linkage quality evaluation should be provided.	materials and methods
**Results**					
Participants	13	(a) Report the numbers of individuals at each stage of the study (*e.g.*, numbers potentially eligible, examined for eligibility, confirmed eligible, included in the study, completing follow-up, and analysed)<br>(b) Give reasons for non-participation at each stage.<br>(c) Consider use of a flow diagram	(a-c) materials and methods	RECORD 13.1: Describe in detail the selection of the persons included in the study (*i.e.,* study population selection) including filtering based on data quality, data availability and linkage. The selection of included persons can be described in the text and/or by means of the study flow diagram.	materials and methods
Descriptive data	14	(a) Give characteristics of study participants (*e.g.*, demographic, clinical, social) and information on exposures and potential confounders<br>(b) Indicate the number of participants with missing data for each variable of interest<br>(c) *Cohort study* - summarise follow-up time (*e.g.*, average and total amount)	a) materials and methods and Supplementary Table S3<br><br>b) materials and methods,Table 1<br><br>c) materials and methods		
Outcome data	15	*Cohort study* - Report numbers of outcome events or summary measures over time<br>*Case-control study* - Report numbers in each exposure	materials and methodsand Supplementary Table S3, results		

For peer review only

		category, or summary measures of exposure *Cross-sectional study* - Report numbers of outcome events or summary measures			
Main results	16	(a) Give unadjusted estimates and, if applicable, confounder-adjusted estimates and their precision (e.g., 95% confidence interval). Make clear which confounders were adjusted for and why they were included (b) Report category boundaries when continuous variables were categorized (c) If relevant, consider translating estimates of relative risk into absolute risk for a meaningful time period	a) results b) results c) results		
Other analyses	17	Report other analyses done—e.g., analyses of subgroups and interactions, and sensitivity analyses	results		
**Discussion**					
Key results	18	Summarise key results with reference to study objectives	results and discussion		
Limitations	19	Discuss limitations of the study, taking into account sources of potential bias or imprecision. Discuss both direction and magnitude of any potential bias	results and discussion	RECORD 19.1: Discuss the implications of using data that were not created or collected to answer the specific research question(s). Include discussion of misclassification bias, unmeasured confounding, missing data, and changing eligibility over time, as they pertain to the study being reported.	NA
Interpretation	20	Give a cautious overall interpretation of results considering objectives,	discussion		

For peer review only

		limitations, multiplicity of analyses, results from similar studies, and other relevant evidence			
Generalisability	21	Discuss the generalisability (external validity) of the study results	discussion		
**Other Information**					
Funding	22	Give the source of funding and the role of the funders for the present study and, if applicable, for the original study on which the present article is based	Title page		
Accessibility of protocol, raw data, and programming code		..	Reference to supplementary data throughout the text	RECORD 22.1: Authors should provide information on how to access any supplemental information such as the study protocol, raw data, or programming code.	materials and methods

*Reference: Benchimol EI, Smeeth L, Guttmann A, Harron K, Moher D, Petersen I, Sørensen HT, von Elm E, Langan SM, the RECORD Working Committee. The REporting of studies Conducted using Observational Routinely-collected health Data (RECORD) Statement. *PLoS Medicine* 2015; in press.

*Checklist is protected under Creative Commons Attribution (CC BY) license.

For peer review only
